# Iron homeostasis and ferroptosis in human diseases: mechanisms and therapeutic prospects

**DOI:** 10.1038/s41392-024-01969-z

**Published:** 2024-10-14

**Authors:** Qin Ru, Yusheng Li, Lin Chen, Yuxiang Wu, Junxia Min, Fudi Wang

**Affiliations:** 1 Institute of Intelligent Sport and Proactive Health, Department of Health and Physical Education, Jianghan University, Wuhan, China; 2 Department of Orthopedics, Xiangya Hospital, Central South University, Changsha, China; 3 National Clinical Research Center for Geriatric Disorders, Xiangya Hospital, Central South University, Changsha, China; 4 The First Affiliated Hospital, Institute of Translational Medicine, Zhejiang University School of Medicine, Hangzhou, China; 5 The Second Affiliated Hospital, School of Public Health, State Key Laboratory of Experimental Hematology, Zhejiang University School of Medicine, Hangzhou, China

**Keywords:** Cell biology, Molecular biology, Molecular medicine

## Abstract

Iron, an essential mineral in the body, is involved in numerous physiological processes, making the maintenance of iron homeostasis crucial for overall health. Both iron overload and deficiency can cause various disorders and human diseases. Ferroptosis, a form of cell death dependent on iron, is characterized by the extensive peroxidation of lipids. Unlike other kinds of classical unprogrammed cell death, ferroptosis is primarily linked to disruptions in iron metabolism, lipid peroxidation, and antioxidant system imbalance. Ferroptosis is regulated through transcription, translation, and post-translational modifications, which affect cellular sensitivity to ferroptosis. Over the past decade or so, numerous diseases have been linked to ferroptosis as part of their etiology, including cancers, metabolic disorders, autoimmune diseases, central nervous system diseases, cardiovascular diseases, and musculoskeletal diseases. Ferroptosis-related proteins have become attractive targets for many major human diseases that are currently incurable, and some ferroptosis regulators have shown therapeutic effects in clinical trials although further validation of their clinical potential is needed. Therefore, in-depth analysis of ferroptosis and its potential molecular mechanisms in human diseases may offer additional strategies for clinical prevention and treatment. In this review, we discuss the physiological significance of iron homeostasis in the body, the potential contribution of ferroptosis to the etiology and development of human diseases, along with the evidence supporting targeting ferroptosis as a therapeutic approach. Importantly, we evaluate recent potential therapeutic targets and promising interventions, providing guidance for future targeted treatment therapies against human diseases.

## Introduction

Ferroptosis is a type of cell death that depends on iron, involving free radicals and lipid metabolism, leading to iron-dependent lipid peroxidation and ultimately cell death.^1,2^ Ferroptosis exhibits unique morphological and biochemical characteristics that distinguish it from other types of cell death,^3,4^ it is primarily influenced by iron homeostasis, redox balance, as well as lipid metabolism in mammalian cells.^5^ Iron is a vital mineral for the human body, and iron metabolism-related molecules including transferrin, metal transporter, and iron response element binding protein 2, can induce ferroptosis by affecting intracellular and systemic iron homeostasis.^6^ Exogenous iron sources, like ferric ammonium citrate (FAC), promotes ferroptosis, while iron chelators like deferoxamine (DFO) inhibit iron overload.^7^ Excess iron stimulates the formation of reactive oxygen species (ROS) via the Fenton reaction, oxidizing phospholipids in unsaturated fatty acid acyl tails, thereby initiating lipid peroxidation. The degree of lipid unsaturation determines the sensitivity to ferroptosis.^8^ Cells primarily catalyze the reduction of lipid peroxides through two antioxidant systems including the glutathione (GSH)/glutathione peroxidase 4 (GPX4) system and the coenzyme Q10 (CoQ10)/ferroptosis suppressor protein 1 (FSP1) system.^9,10^ Detecting changes in the expression and activity of these molecules is essential for further investigation of ferroptosis.

**Fig. 1:**
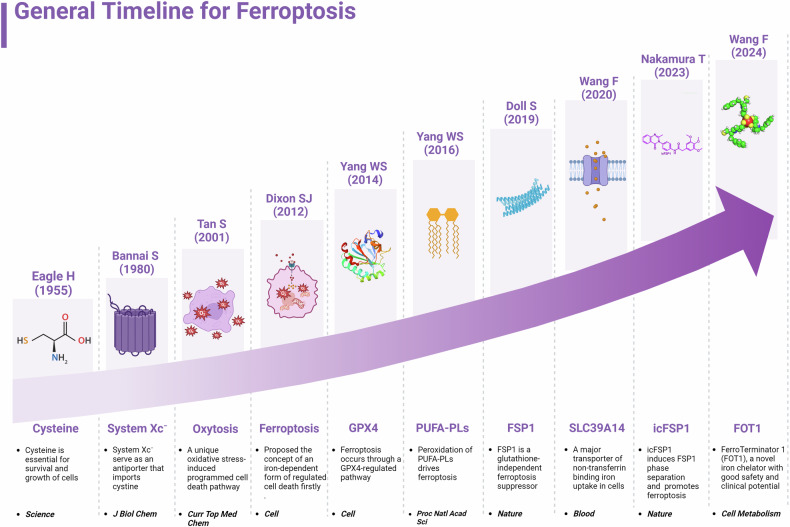
The history and development of ferroptosis. In the figure, we outline the major events in the field of ferroptosis from 1955 to 2024, including the identification of the important role of cysteine in cell survival, the formal definition of ferroptosis, the discovery of system Xc^−^, oxytosis, PUFA-phospholipids (PUFA-PLs), and the proposal of antioxidant systems GPX4 and FSP1. This figure was created with BioRender (https://biorender.com/)

Since ferroptosis was initially documented more than ten years ago,^11^ accumulating data suggests that it is involved in a variety of biological processes, including tumor growth, immune escape, neuron loss, muscle atrophy, and ischemia-reperfusion. This indicates that ferroptosis is essential for health maintenance via regulating metabolic and redox balance, as well as being implicated in the development and treatment of multiple human diseases^12–14^ (Fig. 1). In recent years, rapid advancements in ferroptosis research have led to the discovery of many effective ferroptosis regulators for clinical applications, and these discoveries have opened new avenues for treating ferroptosis-related diseases, with some showing promising clinical prospects.^15^ This review comprehensively introduces the biological role, molecular mechanisms, and progress of ferroptosis in various human diseases. Notably, we explore prospective targets and candidate intervention agents for ferroptosis, paving the way for novel ferroptosis regulators and approaches to intervention in the treatment of multiple human diseases.

## Biological functions of iron homeostasis

Iron is a crucial minor element that is useful for human health. It is involved in a variety of physiological processes, including the production of hemoglobin and myoglobin, the synthesis of cytochrome enzymes and DNA, and mitochondrial function.^16,17^

### Systemic iron metabolism

Usually, the internal iron metabolism remains in relative equilibrium. The body needs to absorb 1 to 2 mg of iron daily to maintain iron homeostasis, primarily from dietary intake. Dietary iron exists as divalent iron (Fe^2+^) and trivalent iron (Fe^3+^). Iron metabolism in the human body involves absorption, transport, utilization, circulation, regulation, and storage. Iron homeostasis is strictly regulated to ensure sufficient iron for essential biological processes while limiting the toxicity of excess iron.^18^ Iron is primarily absorbed in the duodenum and upper jejunum. The oxidation state of iron affects its absorption mechanism in the gastrointestinal tract; iron must be in a divalent (Fe^2+^) state or combine with a transporter protein to be absorbed by intestinal epithelial cells. Trivalent iron (Fe^3+^) in food must first be reduced to divalent iron (Fe^2+^) before passing via divalent metal transporter 1 (DMT1) into epithelial cells. Heme iron (iron-protoporphyrin IX) can be directly absorbed into intestinal epithelial cells via heme carrier protein 1.^19^ After being absorbed into intestinal epithelial cells, some iron is stored as ferritin, while the rest enters the blood circulation through membrane ferroportin (FPN). Divalent iron is reoxidized to trivalent iron by membrane iron transporter auxiliary proteins, then binds to transferrin (TF) and is transported to various organs to perform physiological roles. After reaching target organs through blood circulation, the iron bound TF then binds up to the transferrin receptor 1 (TFR1) and is internalized under the clathrin-dependent endocytosis. While within cells, iron separates from TF and then is reduced to divalent iron, which enters the cytoplasm via DMT1 and is utilized or stored as ferritin.^18,19^ Ferrous ions can normally enter cells via the DMT1 pathway. However, in the case of iron overload, the uptake of non-transferrin-bound iron (NTBI) increases, mediated by metal transport proteins such as SLC39A14.^20^

FPN is the only known mammalian iron export protein that is subsequently oxidized and, in association with TF, distributed to other organs. Under iron-limited conditions, ferritin is mobilized by nuclear receptor coactivator 4 (NCOA4) to lysosomes to degrade and release stored iron. Within the cell, iron storage proteins, transporters, and other factors are regulated to ensure adequate but nontoxic iron levels in cellular, as well as enough storage in the event of iron over capacity.^21^ The body has no active iron removal mechanism and passively loses 1 to 2 mg of iron per day due to the desquamation of skin and mucosal cells, and blood loss. Typically, iron homeostasis is maintained by regulating iron absorption. Excess iron (including heme and non-heme iron) is mainly stored as ferritin and hemosiderin in the liver parenchyma and reticuloendothelial system, especially in the reticuloendothelial cells of the bone marrow, spleen, and liver, along with free ferrous ions, forming a dynamic iron pool cycle in the body.^19^

Regulation of iron balance within the body is primarily performed by hepcidin, which is produced in the liver.^22^ Hepcidin is encoded by the HAMP gene, and is upregulated by a lot of influence factors, such as bone morphogenetic protein (BMP), hemojuvelin (HJV) as well as human hemochromatosis protein (HFE), which regulating both the TFR and the BMP receptor on hepatocytes.^23,24^ Hepcidin and FPN jointly regulate iron circulation from intestinal epithelial cells. When the iron load increases (such as high iron reserve level, high serum iron)^25^ or in cases of infection and chronic inflammation,^26^ elevated hepcidin directly binds to membrane FPN, promoting its internalization as well as degradation in epithelial cells of intestinal, thereby inhibiting iron absorption into the blood.^27^ Under hypoxic conditions, intervened by hypoxia-inducible factor (HIF), there is a reduction in hepcidin expression, which promotes iron release.^28^

At the cellular level, NTBI, which is accepted as the unstable iron pool, is composed of ferrous iron bound by low-affinity iron chelators. Intracellular iron flux of the labile iron pool is regulated by the interaction between iron-responsive elements (IRE) with iron-responsive proteins (IRP)and by controlling the translation of mRNA encoding for FPN, ferritin, TFR1, and DMT1. Moreover, the TFR1, FPN, and DMT1 transcriptions are controlled by HIF via binding to their hypoxia-responsive elements, serving as a potent transcription factor. Prolyl-4-hydroxylase (PHD), which regulates the degradation of HIF, is highly Fe^2+^-dependent, causing HIF became iron metabolism pathologies treatment target.^21,27^ In conclusion, iron is one of the most crucial metal elements in all organisms and maintaining iron homeostasis is essential for regulating the uptake of iron, preserving normal physiological activity, and ensuring proper organ function in the body.^17^

The maintenance of iron homeostasis plays a key role in vital processes such as erythropoiesis, energy metabolism of muscle, cell cycle regulation, hormone production, immune system function, heme synthesis, DNA replication and repair, brain development and aging, and the formation of cytochromes. Iron deficiency or iron overload can severely disrupt physiological functions.^18,21^

### Iron deficiency

Iron is an active metabolically micronutrient, serving as an important enzyme cofactor and key structural protein component. Its most critical roles include oxygen storage (such as myoglobin), transport (such as hemoglobin), and cellular utilization (such as oxidases and the electron transport chain).^29^ Iron deficiency is a familiar nutritional deficiency, typically caused by deficient dietary iron, inadequate iron absorption or loss. Severe deficiency of iron can lead to hypochromic anemia, characterized by reduced heme production.^30^ Heme (a tetrapyrrole containing iron) is critical for various biological functions. It plays essential roles in oxygen transport, gas sensing, oxidative metabolism, xenobiotic detoxification, and microRNA processing.^21^ Iron deficiency-induced heme reduction directly affects oxygen transport and tissue oxygen utilization. The most typical manifestation is impaired aerobic endurance exercise performance, with adolescent and female athletes being particularly prone to iron deficiency.^31^ Heme can also adjust the target gene transcriptions in various pathways, such as circadian rhythm, cell proliferation, apoptosis, antioxidant stress response, ion channel activity, and mitochondrial respiration.^21,32^

In addition, iron is important for normal mitochondrial function, ER stress, DNA impairment and repair, and other cell survival-related enzymatic reactions. Iron imperfection may also induce cognitive function defects and inadequate physical performance.^30,33^ In chronic inflammation patients, the effects of iron deficiency may be particularly severe, exacerbating the underlying disease state and leading to accelerated clinical deterioration. Recent studies have found that iron deficiency exists in about 50% of heart failure patients and is associated with impaired functional capacity and poorer prognosis.^29^ Chronic kidney disease and inflammatory bowel disease are also closely associated with iron deficiency.^34^ Iron also plays a crucial role in vitamin D metabolism and collagen synthesis. The reduction of intracellular iron may disturb activity and homeostasis between osteoclasts and osteoblasts, leading to dysregulation of bone balance and ultimately bone loss. In fact, iron deficiency, whether accompanied by anemia or not, can lead to osteopenia or osteoporosis.^35^ It is also shown that iron deficiency promotes glycolysis by limiting oxidative metabolism, affecting carbohydrate and fat catabolic processes, and leading to skeletal muscle energy disorders. This is an essential factor owing to the loss of muscle oxidative capacity and mass in patients with type 2 diabetes, chronic obstructive pulmonary disease, and heart failure.^36^ Cohort studies have established that iron deficiency is related to reduced muscle mass in individual community residents and impairs the proliferation of myoblasts.^37^ During cancer development, iron metabolism is usually changed at both the cellular and systemic levels. In general, cancer is accompanied by chronic anemia, mediated by high concentrations of hepcidin and exacerbated by therapeutic interventions such as chemotherapy.^27^ TFR1 is overexpressed in cancer cells, increasing the iron levels of intracellular. DMT1 is crucial for intestinal iron absorption and endosomal transport and its expression is notably increased in colorectal cancer. Ferritin, typically found in the cytosol can also be secreted, providing an additional iron source. TFR1, which binds and internalizes ferritin, exhibits altered expression in cancer cells. FPN, which facilitates iron release, is downregulated in breast and prostate cancer. Studies based on these iron-regulated proteins will provide a deeper understanding of the relationship between iron deficiency and cancer treatment.^27^

In addition, the uptake, recycling, and clearance of non-heme iron are crucial for regulating iron’s stable state. The absence or dysfunction of key proteins in these regulatory pathways and their receptors can cause dysregulation of nonheme iron uptake, leading to serious consequences.^21^ For instance, systemic iron is bound primarily to the TF and cellular iron uptake is mediated by TF and its receptor TFR1.^22^ Systemic TF knockout mice die within one day of birth.^20^ Mice lacking TFR1 develop fatal cardiomyopathy with failed oxidative phosphorylation and impaired mitophagy, but iron supplementation prevents these complications.^38^

### Iron overload

Although iron is a helpful component of the human body and the major micronutrient in the human diet, involved in crucial cellular functions and metabolic processes, excessive iron increases the production of ROS, leading to cell dysfunction or death, tissue damage, and organ disease.^39^ Many physiological processes require iron, but iron overload produces toxic effects because excessive free iron can easily change between trivalent and bivalent forms. These act as electron carriers, catalyzing biochemical reactions to produce ROS, which destroy macromolecules such as DNA, proteins, lipids, even and organelles like mitochondria and lysosomes. Therefore, iron overload is toxic, and excess unbound form iron causes severe oxidative stress. When the serum transferrin saturation is about 60%-70%, exceeding the transferrin binding capacity, it forms the NTBI, also known as the unstable iron pool. NTBI can cross the plasma membrane and especially easily be absorbed by various cells, such as cardiomyocytes through calcium channels, causing cascade damage.^39,40^

Iron overload diseases encompass a wide variety of genetic and acquired conditions. Numerous “iron genes” have been identified in hereditary iron overload syndromes, with hemochromatosis (deregulation of intestinal iron absorption) being the most common.^41^ Given that iron overload is caused by long-term transfusion therapy or congenital disorders of iron metabolism, its diagnosis and evaluation are mostly based on clinical presentation, biochemical analysis, and gene mutation analysis.^40^

Iron overload can be classified based on various criteria: the entry route of iron into the body, the primary accumulated tissue, and the overload cause. Excess iron can enter the body through enteral and parenteral pathways. Currently, the adjuvant diagnosis of iron overload is supported by clinical data, high transferrin saturation, and/or elevated serum ferritin levels.^42^ Additionally, liver iron concentration correlates linearly with the total body iron stores, making it a widely accepted surrogate for assessing total iron levels. Magnetic resonance imaging (MRI), highly sensitive to tissue iron, is used as a non-invasive method for severity grading and therapeutic monitoring in patients with known or suspected iron overload.^43^

Disorders in the hepcidin/FPN regulatory system can lead to diseases associated with iron overload. The most common form of hereditary hemochromatosis (HH) is caused by mutations in the HFE, TFR1, hemojuvelin, hepcidin, or FPN genes.^40^ Hepcidin deficiency resulting from these mutations ultimately leads to excessive absorption of dietary iron.^41^

The consequences of iron overload may be diverse beyond hemochromatosis.^39^ Previous reports have found that long-term liver iron overload leads to liver fibrosis, cirrhosis, and even the development of hepatocellular carcinoma (HCC).^44,45^ Myocardial iron overload can cause mitochondrial dysfunction and impaired mitochondrial dynamics, leading to heart failure and arrhythmia.^46^ Iron overload is also associated with cardiovascular diseases. Macrophages of the vascular wall can collect iron, and iron overload in turn causes macrophages to transition to an anti-inflammatory phenotype, which leads to inflammation, oxidative stress, and increased plaque formation.^47^ Iron has also participated in the oxidation of low-density lipoprotein (LDL). The impact of iron metabolism, especially elevated of free iron, on atherosclerosis and other vascular diseases remains controversial. Although iron deposition has been found in atherosclerotic plaques, it is still uncertain whether iron accumulation is a cause or a consequence of these plaques.^48^

Neurodegenerative diseases, for example, Huntington’s disease (HD), Parkinson’s disease (PD), and Alzheimer’s disease (AD), and autism are all associated with iron overload in the developing cerebrum regions.^49^ Iron chelators may alleviate the effects of iron stasis and neurotoxicity on neurons.^50^ After cerebral parenchymal hemorrhage caused by cerebrovascular rupture, iron overload, and toxicity occur in the brain. Given the rich lipid content of the nervous system, hemoglobin-derived iron works with lipid peroxidation to induce ferroptosis, which eventually leads to neuronal mitochondrial damage, edema, and apoptosis of neurons and astrocytes. In the transformation of hemorrhage after cerebral ischemia, iron overload significantly decreases oxidation metabolism, lactic acid generation, and brain pH value, further increasing iron release. This leads to the production of free radicals, destroying mitochondria, affecting energy metabolism, and forming a vicious cycle.^51^

Iron overload can also affect the body’s immunity.^27^ Microorganisms rely on iron for their growth and survival.^52^ High iron content in the body is linked to an increased risk of infection, and iron overload may promote pathogen growth, leading to infection.^53^ Hyperferritinemia and hemoglobinopathy have been reported to accelerate the progression of COVID-19 infection.^54^

At the early stages of the cell life cycle, excessive iron can cause DNA damage and mutations, cell cycle disorders, and tumorigenesis. Clinical studies have also found a 200-fold increased risk of HCC in people with iron overload, such as HH patients. Evidence for a rising incidence of other cancers besides HCC is inconclusive. Some studies have found that iron overload is related to an increased incidence of cancers, while others have reported that iron overload does not affect cancer development. In summary, there is still much to learn about how iron influences the development, progression, and treatment of cancer. Future comprehensive studies are required to clarify iron’s involvement in various types and stages of malignancies.^55^

Iron overload is a risk factor for diabetes mellitus. High dietary iron can confer diabetes risk. Iron plays an important role in the pathogenesis of diabetes, causing β cell failure. Also, iron overload affects energy balance and metabolism, especially in adipocytes. The basic molecular mechanisms involved in these damaging effects include oxidative stress and inflammation-associated intracellular signal transduction pathways.^56,57^

Iron content in skeletal muscle increases with age. This unwanted iron elevation leads to mitochondrial dysfunction and age-related muscle atrophy. Oxidative stress caused by iron overload further induces lipid peroxidation, destroys mitochondrial membrane and function, and causes redox imbalance. Iron overload can also increase the risk of ferroptosis and damage muscle stem cells, affecting skeletal muscle repair and regeneration. Chronic inflammation may interact with skeletal muscle iron overload, aggravating muscle atrophy.^58^

Earlier studies found that iron overload in mice increases oxidative stress in bone, leading to changes in bone microstructure and mineral deposition properties, resulting in osteoporosis.^59^ In recent years, it has been demonstrated that iron has become an independent factor affecting bone metabolic disorders, particularly in the context of iron overload in osteoporosis.^60,61^

## Overview of ferroptosis

**Fig. 2:**
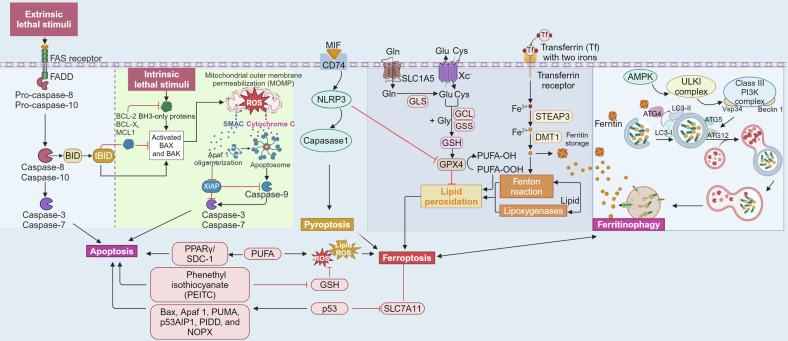
The crosstalk of ferroptosis with apoptosis, autophagy and pyroptosis. In the figure, we illustrate that ferritin produced in the ferroptosis can be digested and degraded by the autophagic mechanism, releasing excessive ferric ions that participate in the Fenton reaction, thereby inducing a vicious cycle of ferroptosis. Moreover, inflammasome NACHT, LRR and PYD domains-containing protein 3 (NLRP3), besides inducing pyroptosis, can promote ferroptosis by inhibiting GPX4. Excessive oxygen free radicals and lipid peroxidation products in the apoptosis signal pathway also induced ferroptosis. This figure was created with BioRender (https://biorender.com/)

Ferroptosis is a cellular response to oxidative stress that depends on excess iron, phospholipids (PLs) with polyunsaturated fatty acid chains (PUFAs), and ROS.^3,15^ Distinct from apoptosis, autophagy, and pyroptosis, ferroptosis is a unique form of iron-dependent regulated cell death. It is marked by massive iron-induced lipid oxidation, enhanced oxidative stress, and depletion of antioxidant defenses.^11,15^ Additionally, there is cellular interaction between ferroptosis and other cell death modes (Fig. 2). Ferroptosis is usually caused by the oxidative stress damage induced by iron overload, which exceeds the antioxidant capacity.^61^ This unique cell death pattern, triggered by iron-dependent peroxidation of phospholipids, is governed by many cellular metabolic pathways, including the redox balance, mitochondrial function, amino acids metabolism, blood lipids, and glucose metabolism.^3^ Thus, in general, ferroptosis is regulated mostly by lipid metabolism, iron homeostasis, and redox system homeostasis.

### Abnormal iron metabolism and ferroptosis

Usually, iron exists in different oxidative states. Ferric iron (Fe^3+^) is stable but insoluble in water. By binding to proteins, such as transferrin, Fe^3+^ can be transported in a redox-inactive state. In contrast, ferrous iron (Fe^2+^) is soluble in water, which makes it potentially harmful. An excess of Fe^2+^ leads to the production of ROS through the Fenton reaction, which induces cell damage or death and potentially impairs organ function eventually.^27^

Iron overload appears to be the main driver of ferroptosis and has been shown to make many cell types more susceptible to this form of cell death. Thus, maintaining iron balance is crucial to safeguard cells against ferroptosis. Iron absorption, storage, and utilization processes are all integral to the regulation of ferroptosis.^15^ Hepcidin and FPN jointly regulate iron circulation from intestinal epithelial cells. Hepcidin can directly bind to the membrane-bound FPN, promoting its internalization and degradation in intestinal epithelial cells, thus inhibiting iron absorption into the blood.^27^ Under hypoxia, HIFs mediate a reduction in hepcidin expression, which promotes iron release.^28^ When cellular iron is sufficient, transferrin-bound iron is reduced to limit excess iron accumulation. In the case of iron excess, the transferrin becomes saturated, and the excess iron circulates, a toxic iron is known as NTBI.^62,63^ Excess iron is stored in unstable iron pools, which can increase due to metabolic imbalance, leading to increased ROS production through Fenton reactions. When iron limitation in cells, ferritin is mobilized to the lysosome via NCOA4 to degrade and liberate the stored iron.^21^ Thus, therapeutic approaches for iron overload include direct or indirect modulation of hepcidin expression to reduce iron absorption.^64,65^ Alternatively, the use of iron chelators, such as DFO, can prevent ferroptosis.^11^ Effectively reducing NTBI uptake mediated by DMT1 has been shown to reduce iron-induced damage, suggesting that DMT1 may be a promising target for alleviation of ferroptosis.^62,63^ Transferrin can protect the body from liver damage caused by the increased intracellular NTBI accumulation mediated by the SLC39A14.^66^ Transferrin also protects the cells from ferroptosis.^20,67^ It is also suggested that TRF1-mediated intracellular iron uptake is required for ferroptosis, and using RNA interference with TRF1 can effectively prevent ferroptosis.^66^ FPN has also been shown to modulate cell sensitivity to ferroptosis in vitro,^68^ and FPN inhibitors can be used to modulate ferroptosis.^69^

### Lipid metabolism and ferroptosis

Excess ROS produced by the Fenton reaction further causes lipid peroxidation, eventually leading to cell death.^70^ Thus, lipid and lipid peroxidation play a critical role in the progression of ferroptosis. British chemist Henry Fenton discovered that the mixture of hydrogen peroxide and ferric ions has strong oxidative properties, with the main oxidative component being the hydroxyl radical (OH•). Iron overload in the body increases the levels of NTBI, which reacts with hydrogen peroxide through the Fenton reaction to generate many hydroxyl radicals (OH•) and ROS. These ROS oxidize PUFAs in cell membranes into lipid peroxides (PUFA-OOH), thereby destroying cell membrane structural stability, attacking cellular DNA and proteins, and causing ferroptosis.^71^

Acyl-CoA synthetase long-chain family member 4 (ACSL4) and lysophosphatidylcholine acyltransferase 3 (LPCAT3) were firstly identified as key enzymes which promote PUFAs into PLs for forming PL-PUFAs and induce ferroptosis.^15,72^ ACSL4 facilitates the transformation of free PUFAs into acyl-CoA derivatives known as PUFA-CoAs. These derivatives can then be further catalyzed by LPCAT3 to form PL-PUFAs, which participate in cellular membrane biosynthesis and are highly prone to peroxidation.^72^ In contrast, ACSL3 shifts monounsaturated fatty acids (MUFAs) into acyl-CoA esters, which are then integrated into membrane phospholipids, a process that also requires the involvement of LPCAT3,^73^ and is closely associated with phospholipid remodeling.^74^ Interestingly, exogenous MUFAs have been found to effectively inhibit ferroptosis. This protective mechanism is likely linked to the prevention of lipid ROS accumulation in plasma membranes and the reduction of phospholipids containing oxidizable PUFAs, an effect that requires ACSL3 activation.^73^ Increased expression or activity of ACSL4 may trigger ferroptosis in multifarious pathophysiological settings. Inhibition of ACSL4 expression or knockdown of LPCAT3 may be key mechanisms for ultimately regulating ferroptosis through different signaling pathways.^3,73^

Additionally, lipoxygenases (LOXs) are a family of iron-containing enzymes that directly oxidize PUFAs in cell membranes and PUFA-containing lipids, mediating lipid peroxidation to produce hydrogen peroxide.^75^

### Redox reaction imbalance and ferroptosis

Ferroptosis is characterized by the interplay and equilibrium between the antioxidant defense system and intracellular oxidative stress. Antioxidant systems play a crucial role in preventing peroxidative damage by scavenging free radicals or indirectly consuming compounds that induce free radical generation, thus preventing ferroptosis.

With consideration of oxidative and antioxidative defense, several antioxidant systems are involved in defending against ferroptosis. GSH (the most abundant reducing agent), is crucial for the iron-sulfur cluster biogenesis and serves as a cofactor for various enzymes, such as glutathione peroxidase and transferase. System Xc^−^, GSH synthesis, and GPX4 have been shown to protect against cell death triggered by multiple oxidative stress responses.^3^ System Xc^−^ consists of a solute carrier family 7 members 11 (SLC7A11, or xCT) and solute carrier family 3 members 2. Cystine enters cells through the cystine/glutamate reverse transporter (system Xc^−^) and is then reduced via the glutathione or thioredoxin reductase 1-dependent cysteine reduction pathway to generate cysteine and promote GSH synthesis.^3^ GSH acts as a cofactor of GPX4, promoting the cellular reduction of phospholipid peroxides (PLOOHs) to non-toxic phospholipid alcohols (PLOHs), thereby alleviating cellular oxidative damage induced by ferroptosis.^3,76^ Glutathione-disulfide reductase then uses electron-catalyzed oxidized glutathione (GSSG) to regenerate GSH.^3^ Therefore, decreased GSH content in both intracellular and mitochondrial compartments may contribute to the occurrence of ferroptosis.^77,78^ The extracellular and intracellular cystine and glutamate concentrations keep the function of system Xc^‒^ to protect cells in an antioxidant state. Increasing extracellular glutamate levels limits the uptake of cystine, gradually inducing ferroptosis.^11,78^ Moreover, suppressing either component of system Xc^–^ induces ferroptosis by disturbing cystine uptake, then limiting GSH synthesis.^11^ In the mitochondria, carrier proteins such as oxoglutarate carrier (also known as SLC25A11) and dicarboxylate carrier (SLC25A10) play roles in the mitochondrial transport of GSH,^79^ and are therefore also regulatory targets of ferroptosis. In this pathway, GPX4 also serves as a crucial regulator of ferroptosis.^78^ It was found that reduced GPX4 expression leads to increased lipid ROS levels and activation of ferroptosis.^76^ Conditional knockout of GPX4 causes death of mouse embryonic fibroblasts in a lipid peroxide-dependent, non-apoptotic form and causes neurodegenerative disease in the cortical regions and hippocampus of the mouse brain.^80^

However, the knockout of GPX4 alone is insufficient to induce ferroptosis, indicating the presence of compensatory pathways. One such pathway, identified later, is the FSP1 pathway.^72,81^ FSP1 is an oxidoreductase which reduces CoQ10, and generating a lipophilic antioxidant and quits the progress of lipid peroxides. FSP1 has been reported to trap lipid peroxyl radicals, thereby protecting against lipid peroxidation and ultimately suppressing ferroptosis at the plasma membrane.^81,82^

Recently, the PLs-modifying enzymes lysophospholipid acyltransferase 1 (MBOAT1) and MBOAT2 were identified as novel sex hormone-dependent inhibitors of ferroptosis. MBOAT1 and MBOAT2 remodel PLs to protect cells from ferroptosis independent of GPX4.^83^ Additionally, GTP cyclohydrolase 1 prevents ferroptosis through its metabolites dihydrobiopterin and tetrahydrobiopterin (BH4). BH4 has been shown to protect phospholipids containing two PUFA tails from oxidative degradation.^84^

Alternatively, accumulating evidence suggests that multiple other signaling pathways can influence cellular sensitivity to ferroptosis in specific biological settings. Nuclear factor erythroid 2-related factor 2 (Nrf2) can reduce ferroptosis through stimulating the typical ferroptosis target genes expressions, such as ferritin heavy chain 1 (FTH1), quinone oxidoreductase 1 (NQO1), and heme oxygenase-1 (HO-1).^85^ The electron transport chain regulates ferroptosis, where most ROS are generated during electron transport. ROS generation increases when any key enzyme deficiency or inhibited, while high levels of cumulative ROS cause ferroptosis.^86^ Excessive ROS accumulation in mitochondria can cause ferroptosis. Mitochondrial respiratory chain complexes I (NADH-CoQ reductase complex), II (succinate dehydrogenase complex), III (cytochrome bc-1 complex) can, under different metabolic stress, produce ROS that damage the mitochondrial membrane, resulting in lipid peroxidation and ultimately leading to ferroptosis. This process is inhibited by mitochondria-targeting antioxidants.^87^ Studies have also shown that hypoxia promotes the development of ferroptosis by activating HIFs, which induce the increased expression of lipid-related proteins and drive the enrichment of polyunsaturated lipids to induce ferroptosis.^88^ In terms of immune regulation, it has been documented that interleukin-4 (IL-4) and IL-13 inhibit the expression of GPX4 in some tissues (including heart, spleen, lung, and kidney), while the expression of ALOX15 increases, resulting in the massive production of arachidonic acid metabolites and inducing lipid peroxidation.^89^

## Role of ferroptosis in human diseases

**Fig. 3:**
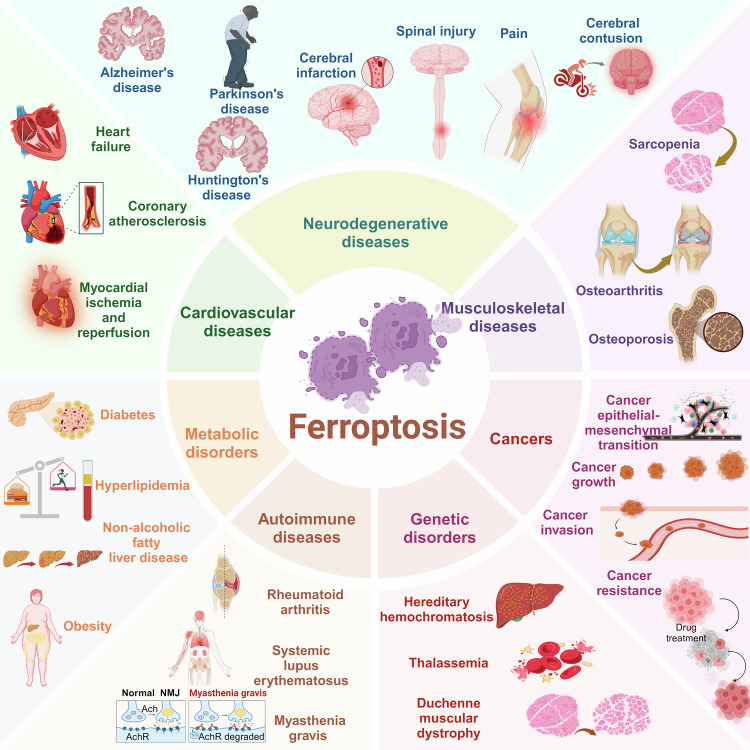
The role of ferroptosis in human diseases. The figure illustrates that ferroptosis plays a significant role in many human diseases. In neurodegenerative conditions, it is involved in the pathological process of Alzheimer’s disease (AD), Parkinson’s disease (PD), and Huntington’s disease (HD), as well as in cerebral ischemia-reperfusion (I/R) injury, brain and spinal cord injury, and pain. In the circulatory system, ferroptosis contributes to heart failure, coronary heart disease, and myocardial I/R injury. In the musculoskeletal system, it leads to functional decline in skeletal muscle, bones, and joints. In chronic metabolic diseases, ferroptosis can trigger the occurrence and development of diabetes mellitus, hyperlipidemia, fatty liver disease, and obesity. In cancer, ferroptosis mainly affects tumor growth, metastasis, invasion, and chemoresistance. In autoimmune diseases, it is mainly involved in the pathogenesis of rheumatoid arthritis, systemic lupus erythematosus, and myasthenia gravis. In genetic diseases, ferroptosis predominantly affects thalassemia and progressive muscular dystrophy. This figure was created with BioRender (https://biorender.com/)

Increasing evidence has shown that ferroptosis has an important role in many biological and pathological processes and is involved in the onset and progression of multiple human diseases (Fig. 3). Here, we discuss recent breakthroughs in understanding the significance of ferroptosis in cancers, metabolic disorders, autoimmune disorders, genetic disorders, cardiovascular diseases, neurodegenerative diseases, and musculoskeletal diseases. We also describe the current roles of ferroptosis in the preclinical treatment of these diseases.

### Ferroptosis in cancers

#### Ferroptosis and the epithelial-mesenchymal transition

In vertebrates, the two basic cell types are epithelial cells and mesenchymal cells, which can transform into one another through a reversible process known as epithelial–mesenchymal transition (EMT).^90^ In this process, epithelial cells lose their characteristic structure and functions, including cell polarity, tight junctions, and intercellular adhesion, and gain the morphology and functions of mesenchymal cells, including cell migration, invasion, and resistance to apoptosis.^91^ Under physiological conditions, this process plays an important role in embryonic development and tissue repair. EMT is a primary driver of cancer progression, from early to later aggressive and metastatic stages, and the aggressive growth of many malignancies depends on EMT activation inside tumor cells. E-cadherin is an epithelial cell surface marker that creates adhesive structures between epithelial cells, resulting in strong and enduring intercellular attachment. N-cadherin forms weakly adhesive structures between mesenchymal cells. During EMT, E-cadherin expression decreases, while N-cadherin expression increases.^92,93^ Ferroptosis and EMT have a complicated interaction, which is mediated by multiple signaling pathways.^94^ Cancer cells undergoing EMT may have greater sensitivity to ferroptosis, and cancer cells with mesenchymal characteristics are generally more vulnerable to ferroptosis than cancer cells with epithelial properties.^90,95^

Melanoma is a highly aggressive skin cancer known for its early metastasis, significant malignancy, and high mortality rate. Transforming growth factor-β1 (TGF-β1) can induce the EMT phenotype in melanoma cells and upregulate the levels of GPX4 and SLC7A11, while gambogenic acid, a xanthone derived from traditional Chinese medicine Gamboge, can promote ferroptosis by inhibiting the SLC7A11/GPX4 pathway in melanoma cells stimulated by TGF-β1.^96^ Specifically, this compound decreases the levels of GSH and superoxide dismutase (SOD), increases malondialdehyde (MDA) levels, promotes E-cadherin expression, and lowers N-cadherin expression,^96^ indicating that gambogenic acid can induce ferroptosis to inhibit TGF-β1-induced EMT by disrupting the oxidative stress balance of melanocytes. TGF-β1 induced EMT increases the intracellular levels of unstable iron and ROS, and the elevated iron content and oxidative stress in cells undergoing EMT make them more susceptible to ferroptosis. Erastin was shown to induce ferroptosis in lung cancer A549 cells, accompanied by increased oxidative stress, lipid peroxidation, and decreased expression of E-cadherin and SLC7A11. The ferroptosis inhibitor ferrostatin-1 (Fer-1) effectively inhibits erastin-induced ferroptosis in A549 cells.^97^ Notably, GPX4 expression did not significantly affect TGF-β1-induced EMT in A549 cells, nor did it reverse erastin-induced ferroptosis.^97^

The incidence of renal cell carcinoma (RCC) has been increasing annually. Hsa_circ_0057105 has potential carcinogenicity and is an independent prognostic factor in patients with RCC.^98^ COL1A1 (induction of EMT activation) and VDAC2 (regulation of ferroptosis sensitivity) are direct target genes of miR-577. Hsa_circ_0057105 can inhibit the expression of miR-577,^98^ thereby promoting the activation of EMT in RCC.

Mitochondrial pyruvate carrier 1 (MPC1), an inner membrane protein that transfers pyruvate into mitochondria, is differentially expressed in normal and cancer cells, and inhibition of MPC1 expression can prompt a shift towards EMT in cancer cells.^99^ The expression of MPC1 is regulated by histone lysine demethylase 5A (KDM5A), also referred to as RBP2 or JARID1A. The KDM5A-MPC1 signaling axis may enhance their sensitivity to ferroptosis inducers by regulating mitochondrial metabolism and EMT in cancer cells.^100^ Analysis of The Cancer Genome Atlas data showed that low MPC1 expression in head and neck cancer (HNC) tissues was associated with poor overall survival.^101^ KDM5A inhibition increases MPC1 expression and reduces the susceptibility of erlotinib-tolerant persistent head and neck cancer cells (erPCCs) to ferroptosis inducers. Conversely, MPC1 inhibition increases cell susceptibility to ferroptosis by preserving the mesenchymal properties of erPCCs,^101^ indicating that MPC1 regulates ferroptosis in erPCCs through EMT.

Zinc finger E-box binding homeobox 1 (ZEB1) is a crucial molecule involved in the EMT.^102^ ZEB1 silencing induces E-cadherin expression and decreases susceptibility to ferroptosis in HNC cells, while ZEB1 overexpression increases susceptibility to ferroptosis.^90^ Histone demethylase KDM3B is another potential epigenetic regulator of ferroptosis, as it upregulates the expression of SLC7A11, indicating that ferroptosis is influenced by epigenetic mechanisms.^103^ Activation of histone deacetylase Sirtuin1 (SIRT1) promotes ferroptosis in HNC cells, increases ZEB1 expression, and decreases E-cadherin levels. Silencing SIRT1 gene or inhibiting it pharmacologically with Ex-527 can reduce cell ferroptosis and inhibit EMT,^90^ suggesting that epigenetic reprogramming of EMT helps to promote ferroptosis in HNC cells.

The expression level of SIRT3 in patients with gallbladder cancer (GBC) is notably lower compared to adjacent normal tissues, and low expression of SIRT3 is positively associated with poor overall survival. SIRT3 knockdown induces energy metabolism and mitochondrial respiration in GBC cells, but reduces ROS production and ACSL4 expression, and inhibits AKT-dependent ferroptosis.^104,105^ Meanwhile, SIRT3 knockdown results in reduced E-cadherin expression and increased levels of N-cadherin and vimentin. Conversely, SIRT3 overexpression has the opposite effects,^105^ suggesting that SIRT3 inhibits AKT-dependent mitochondrial metabolism and EMT, thereby promoting ferroptosis.

BACH1 is a hemoglobin-binding transcription factor that plays a role in regulation of oxidative stress and iron metabolism.^106,107^ BACH1 directly affects EMT by regulating intercellular adhesion genes and transcription factors, including Snail2 and FOXA1. Pancreatic ductal adenocarcinoma (PDAC) has the worst prognosis among malignant tumors. FOXA1 can promote the expression of E-cadherin, while BACH1 inhibits the expression of FOXA1 and genes critical for epithelial cell adhesion, including claudin3 and claudin4, thus promoting the EMT.^108^ Further research has shown that BACH1 inhibits the Nrf2 pathway by binding to Nrf2, and promotes the increase of labile iron in PDAC cells, leading to ferroptosis.^109^ Therefore, BACH1 is possible to link ferroptosis with cancer EMT, and studying the function and regulation of BACH1 could enhance our understanding of the interaction between ferroptosis and EMT, providing guidance for developing new treatment strategies.

Colorectal cancer (CRC) is linked to significant morbidity and mortality. The combination of cetuximab and β-elemene not only promotes ferroptosis in CRC cells, but also promotes the expression of E-cadherin (epithelial marker) while reducing the expression of mesenchymal markers such as snail, vimentin, N-cadherin, slug, and MMP-9. These effects can be reversed by ferroptosis inhibitors DFO and Fer-1,^110^ indicating that the combination of cetuximab and β-elemene inhibits EMT by promoting ferroptosis.

The global incidence of lung cancer is increasing annually, with lung adenocarcinoma being the most common pathological type.^111,112^ lncRNA LINC00641 is primarily localized in the nucleus, modified by N6-methyladenosine (m6A), and associated with various cancers. lncRNA-LINC00641 is significantly downregulated in lung cancer tissue than that in the neighboring normal lung tissue, and its downregulation is associated with poor prognosis in lung adenocarcinoma.^113^ Knockdown of LINC00641 increases N-cadherin levels by upregulating Human antigen R (HuR) protein, ultimately promoting EMT.^114^ Notably, LINC00641 knockdown in lung cancer cells increases ferroptosis sensitivity by promoting arachidonic acid metabolism.^114^ This is inconsistent with findings that inhibiting ferroptosis promotes EMT in other tumors. 2,2′-di-pyridylketone hydrazone dithiocarbamate s-butyric acid (DpdtbA) exhibits interesting antitumor activities against esophageal and gastric cancer. DpdtbA inhibits EMT by downregulating the proline hydroxylase 2/HIF-1α pathway and inducing ROS production through ferritinophagy.^115^ ARNTL2 is a circadian transcription factor, which has been shown to be involved in the pathogenesis of various tumors in recent years.^116,117^ ARNTL2 is highly expressed in patients with lung adenocarcinoma and has been shown to be an independent predictor of poor prognosis in these patients. Knockdown of ARNTL2 significantly decreases the expression levels of Nrf2, SLC7A11, and CDGSH iron-sulfur domain protein 1 of lung adenocarcinoma cells, and increases the sensitivity to ferroptosis. In addition, ARNTL2 knockdown decreases N-cadherin expression and increases the expression of β-catenin and E-cadherin in lung adenocarcinoma cells, while the opposite effects are observed in ARNTL2-overexpressed cells,^118^ suggesting that ARNTL2 overexpression may promote EMT by inhibiting ferroptosis.

#### Ferroptosis in cancer growth

Ovarian cancer is a gynecological tumor with high morbidity and mortality.^119^ Agrimonolide dose-dependently increases intracellular ROS and iron levels, while decreasing SLC7A11 and GPX4 levels.^120^ Sterol CoA desaturase (SCD1) is a lipid regulatory enzyme that catalyzes the desaturation of saturated fatty acids to their Δ9-monounsaturated counterparts.^121^ Agrimonolide induces ferroptosis in ovarian cancer cells by inhibiting SCD1 protein translation and stability. Additionally, agrimonolide significantly downregulates SCD1 expression in tumor tissues in SKOV-3 xenotransplantation models, reducing tumor growth of ovarian cancer.^120^

CRC is a prevalent malignancy within the digestive system and is one of the most pressing health issues today. Therefore, there is an urgent need for the scientific community to find effective ways to inhibit the proliferation and metastasis of CRC cells. Chemical modification of RNA plays a crucial role in regulating gene expression programs in a temporal and spatial manner during development. RNA methylation, introduced at highly conserved sites by specific modification enzymes, is closely associated with the pathogenesis of cancer.^122,123^ METTL17 is a member of the methyltransferase family.^124^ METTL17 is upregulated in colorectal cancer, and the absence of METTL17 increases the sensitivity of colon cancer cells to ferroptosis, and promotes cell proliferation and the growth of transplanted tumors. In addition, inhibiting METTL17 significantly reduces mitochondrial RNA methylation, impairing the translation of mitochondrial protein-coding genes, affecting mitochondrial energy metabolism, and enhancing mitochondrial and intracellular ROS and lipid peroxidation levels. This reveals that the METTL17-mediated defense mechanism against ferroptosis may serve as a potential therapeutic target for colon cancer.^125^ Tagitinin C, a sesquiterpene lactone derived from gentian, induces oxidative cellular microenvironment that triggers ferroptosis in colon cancer cells and inhibits cancer cell growth.^126^ The ferroptosis induced by Tagitinin C is characterized by a decrease in GSH and an increase in lipid peroxidation. Mechanistically, Tagitinin C induces oxidative stress and endoplasmic reticulum (ER) stress, leading to Nrf2 nuclear translocation and subsequent expression of its downstream target, HO-1. This results in increased unstable iron pools and promoted lipid peroxidation.^126^

Endometrial cancer, one of the most prevalent malignancies of the female reproductive system, occurs mainly in perimenopausal and postmenopausal women.^127,128^ RBM3 is an important RNA-binding protein with a dual role in tumors.^129,130^ By promoting the expression of RBM3, sodium butyrate indirectly inhibits the expression of SLC7A11,^131^ thus aggravating ferroptosis of endometrial cancer cells and inhibiting cell clonal formation and cell proliferation.

Despite the availability of traditional treatment strategies, gastric cancer remains a major global health challenge.^132^ Ubiquitin-specific protease 7 (USP7) plays a key role in the development and resistance of gastric cancer and is a promising target.^133^ USP7 regulates SCD1 through deubiquitination, inhibiting SCD1 degradation and ferroptosis in gastric cancer.^134^ DHPO, as an effective inhibitor of USP7, can directly bind to USP7 to induce ferroptosis of gastric cancer cells.^134^ By inhibiting cell proliferation and cladogenesis, DHPO exhibits significant antitumor activity in vitro and in vivo.^134^

Leukemia is characterized by the uncontrolled proliferation of undifferentiated myeloid or lymphoid progenitors, impeding the normal function of hematopoietic cells and affecting the development of non-hematopoietic cells.^135^ Dihydroartemisinin can significantly inhibit the viability of leukemia cells, causing cell cycle G0/G1 phase arrest and ferroptosis, and the mechanism may be related to inducing autophagy and accelerating ferritin degradation by regulating AMP-activated kinase (AMPK)/mTOR/p70S6k signaling pathway.^136^ Overexpression of the iron-sulfur cluster assembly enzyme can prevent dihydroartemisinin-induced ferroptosis by controlling iron metabolism, raising GSH levels, and preserving mitochondrial function.^136^ Inducing ferroptosis may be a promising therapeutic approach for targeting leukemia cells.

#### Ferroptosis in cancer metastasis

Metastasis is a key factor leading to poor prognosis of cancer patients. The invasion and metastasis of colorectal, breast and lung cancers has been associated with the overexpression of cysteine protease inhibitor SN (Cystatin SN, CST1).^137^ Clinical data indicate that CST1 levels are elevated in both peripheral blood and ascites in gastric cancer patients with metastasis, and CST1 expression is significantly elevated in metastatic gastric cancer tissues. According to a multi-factor Cox regression model analysis, CST1 is identified as an independent risk factor for prognosis in gastric cancer patients.^138^ The interaction between CST1 and GPX4 improves the stability of GPX4 protein and reduces the intracellular ROS, thus inhibiting ferroptosis. The invasion and migration of stomach cancer cells, as well as their peritoneal, lung, and liver metastases, are markedly enhanced by overexpression of CST1.^138^ USP7 regulates SCD1 through deubiquitination, inhibiting ROS, MDA accumulation, and iron overload, thereby inhibiting ferroptosis in gastric cancer cells. DHPO can inhibit USP7 activity to induce ferroptosis, preventing the invasion and migration of gastric cancer cells. In vivo studies of mouse models of orthotopic gastric tumor confirm the effectiveness of DHPO in inhibiting gastric cancer metastasis without obvious toxicity.^134^ These results highlight the potential for treating gastric cancer by modulating ferroptosis.

Breast cancer is the most prevalent malignancy among women, with the highest incidence among female malignancies, and is a leading cause of cancer-related mortality.^111^ Erastin increases Fe^2+^ levels and ROS production in breast cancer cells, promoting ferroptosis. Macrophages, which are crucial immune cells within the tumor microenvironment, play a significant role in cancer progression. Inhibition of the polarization of M2 macrophage can inhibit cancer cell metastasis.^139^ Exosomes derived from ferroptosis-induced breast cancer cells (Fe-Exos) markedly reduce the expression of macrophages M2 markers Arg-1 and CD206. Fe-Exos cultured macrophages, in turn, inhibit the invasion and migration of breast cancer cells.^140^

Wilms tumor 1-associated protein (WTAP), as an important component of the m6A methyltransferase classical complex, promotes the progression of various human malignant tumors by regulating RNA methylation levels.^141,142^ WTAP is highly expressed in triple-negative breast cancer (TNBC) tissue, suggesting that WTAP may be a risk factor for promoting the development of TNBC.^143^ Transcription factor nuclear protein 1(NUPR1) has been reported to transfer lipocalin 2 (LCN2) and block ferroptosis by reducing iron accumulation.^144^ In TNBC tissue, WTAP, NUPR1, and LCN2 are significantly overexpressed, and WTAP upregulates LCN2 expression by regulating NUPR1 m6A modification.^145^ WTAP knockdown promotes ferroptosis and inhibits the migration and invasion of TNBC cells, which is eliminated by NUPR1 overexpression. NUPR1 silencing also inhibits the invasion and migration of TNBC cells by inducing ferroptosis.^145^

The clinical metastasis rate of osteosarcoma is high, with 80% of osteosarcoma cases showing local infiltration and distant metastasis.^146^ Osteosarcoma cell lines and tissues show low expression of miR-144-3p and high expression of its target gene ZIB1, and miR-144-3p level is negatively correlated with the invasion and migration ability of osteosarcoma cells.^147^ Overexpression of miR-144-3p could downregulate the GSH/GSSG ratio, increase the levels of ROS, Fe^2+^, and ACSL4, promote ferroptosis and thus reduce the metastasis of osteosarcoma cells, while high expression of ZIB1 plays the opposite role,^147^ indicating that miR-144-3p and its downstream ZIB1 are involved in the development and metastasis of osteosarcoma mainly by interfering with iron metabolism and redox homeostasis.

Long-distance metastasis is a major obstacle to clinical treatment of RCC.^148^ Kruppel-like factor 2 (KLF2) is a transcription factor containing conserved zinc finger domains.^149^ Bioinformatic analysis and immunohistochemical results of clinical samples show that KLF2 expression is reduced in patients with metastatic RCC, and overall survival and metastasis-free survival are shortened in patients with low KLF2 expression. Mechanistically, there is a negative correlation between KLF2 and GPX4, and KLF2 deficiency increases the level of GPX4 and inhibits ferroptosis, promoting the invasion and migration of renal cancer cells, which can be reversed by overexpression of GPX4,^150^ indicating that promoting ferroptosis of cancer cells may be a promising strategy for the clinical treatment of advanced RCC.

Ovarian cancer has the highest mortality rate among gynecological malignancies, posing a serious threat to women’s health and life. Due to the absence of typical clinical symptoms and specific biomarkers in the early stages, and more than 70% of ovarian cancer cases have progressed to an advanced stage by the time they are diagnosed.^151^ CCAAT/enhancer binding protein gamma (CEBPG) is crucial in various biological processes, such as cell proliferation, cell differentiation, and energy metabolism.^152^ Compared with benign ovarian tissue, CEBPG expression is significantly increased in ovarian cancer tissue, and high CEBPG expression is closely associated with the poor prognosis in ovarian cancer patients.^153^ Knockout of CEBPG inhibits the proliferation, invasion and metastasis of ovarian cancer cells and the progression of transplanted tumor in mice.^153^ Importantly, dual luciferase reporter gene experiments reveal that CEBPG, as a transcription factor, regulates ferroptosis of ovarian cancer cells primarily through transcriptional control of SLC7A11, affecting the metastasis of ovarian cancer cells.

CRC is the third cause of cancer death worldwide. Approximately 40% of colon cancer patients have KRAS mutations, and patients with KRAS^G13D^ have a poor prognosis, including susceptibility to metastasis and short survival time, while anti-EGFR therapy offers little benefit.^154^ Natural product erianin increases the accumulation of ROS and Fe^2+^ in KRAS^G13D^ colon cancer cells, causing cellular lipid peroxidation and mitochondrial morphological changes, thereby inhibiting their migration and invasion. Interestingly, erianin-induced ferroptosis is accompanied by autophagy and can be reversed by autophagy inhibitors and ATG5 knockdown,^155^ implying that it is autophagy-dependent. Results from animal models have also shown that erianin can inhibit spleen-liver metastasis of colon cancer cells. Acyl-coenzyme A dehydrogenase, short/branched-chain (ACADSB, also called SBCAD) belongs to the acyl-CoA dehydrogenase family and plays a role in the metabolism of fatty acids and branched-chain amino acids.^156^ ACADSB expression is low in CRC tissues, negatively correlated with the pathological grade of colorectal cancer, and positively associated with the overall survival rate of CRC patients.^157^ ACADSB negatively regulates the expression of GPX4 and glutathione reductase in colon cancer cells, and overexpression of ACADSB increases the concentration of MDA, Fe^2+^ and lipid peroxides, inducing cell ferroptosis, and inhibiting the migration and invasion of CRC cells.^157^

PDAC is one of the deadliest cancers, with 75% of patients developing liver metastases within 1–2 years following original tumor resection.^158^ Mitochondria play a crucial role in the occurrence and development of pancreatic cancer.^159^ The mitochondrial calcium uniporter (MCU) regulates Ca^2+^ in mitochondria, and Ca^2+^ uptake by MCU buffers the increase of cytoplasmic Ca^2+^ and regulates mitochondrial oxidative phosphorylation.^160–162^ MCU stimulates PDAC cell invasion, migration, and metastasis via activating the Kelch-like ECH-associated protein 1 (Keap1)-Nrf2 antioxidant pathway. Pharmacological inhibition of SLC7A11 significantly causes tumor regression of PDAC and eliminates MCU-driven metastasis, suggesting that SLC7A11 may be a downstream target of the MCU-Nrf2 axis.^162,163^ Paradoxically, despite enhanced cystine uptake, MCU-overexpressed PDAC exhibited typical features of ferroptosis caused by cystine deprivation. In both in vivo patient-derived xenograft models and in vitro patient-derived organoid models, high-MCU PDAC show increased sensitivity to SLC7A11 inhibition compared to low-MCU tumors,^163^ suggesting that MCU-mediated ferroptosis could serve as a therapeutic strategy to prevent PDAC tumor metastasis.

Bladder cancer has become the most common malignancy of the urinary system, and there is currently no effective treatment strategy for invasive bladder cancer.^127,164,165^ Heat shock protein family A (HSP70) member 5 (HSPA5) is abnormally expressed in various tumors, closely related to tumor progression and prognosis.^166–168^ The expression of HSPA5 increases in bladder cancer and correlates with patient prognosis. Knockdown of HSPA5 inhibits the migration and invasion of bladder cancer cells by inhibiting the vascular endothelial growth factor A (VEGFA)/vascular endothelial growth factor receptor 2 signaling pathway, and overexpression of VEGFA alleviates the effects of HSPA5 downregulation.^169^ Additionally, overexpression of HSPA5 inhibits ferroptosis through the P53/SLC7A11/GPX4 pathway and may serve as a novel biomarker and potential therapeutic target.

Head and neck squamous cell carcinoma (HNSCC) is a highly aggressive tumor, with high morbidity and mortality. CAV1 is a membrane protein involved in cell signaling and lipid metabolism.^170^ Compared with normal tissues, caveolin-1 (CAV1) expression is upregulated and associated with poor prognosis in HNSCC patients, and negatively correlated with ROS level. Downregulation of CAV1 reduces the levels of GPX4 and FTH1, increases ROS and Fe^2+^ concentrations, promotes ferroptosis, and reduces cancer cell migration and invasion,^171^ suggesting that the regulatory pathways of CAV1 and ferroptosis may be potential targets for diagnosis and combination therapy strategies in patients with HNSCC.

Prominin-2 (PROM2) is a glycoprotein physiologically expressed in many normal tissues, mainly in epithelial cells.^172^ PROM2 regulates endocytosis, and its overexpression leads to significant changes in plasma membrane organization and function, including increased cellular pseudopodia, which has invasive potential and is a potential biomarker for predicting distant metastasis and decreased survival.^173,174^ PROM2 overexpression is strongly associated with the increased metastatic potential of melanoma, with similar results found in patient-derived xenotransplantation models of RCC and TNBC.^175^ Using oligonucleotide antisense anti-PROM2 to reduce PROM2 expression promotes ferroptosis of melanoma cells and prevents metastasis in melanoma xenotransplantation.^175^ These findings support further use of PROM2-targeted intervention strategies in treating metastatic melanoma and other cancers.

#### Ferroptosis in drug resistance in cancer

Despite significant advances in cancer treatment, drug resistance remains a major clinical challenge. Recent studies have confirmed that ferroptosis is associated with resistance to cancer treatment, and dysregulation of ferroptosis often leads to drug resistance and treatment failure.^176^

Glioblastoma multiforme (GBM) patients resistant to temozolomide have a poor prognosis. The androgen dehydroepiandrosterone enhances GBM resistance to temozolomide by activating the androgen receptor, thereby attenuating DNA damage. Curcumin analog ALZ003 can induce ubiquitination degradation of androgen receptor and significantly increase the sensitivity of GBM cells to temozolomide both in vitro and in vivo, and the mechanism may be related to ROS accumulation, lipid peroxidation and inhibition of GPX4, which are major features of ferroptosis.^177^ This indicates that inducing ferroptosis in GBM can reverse temozolomide resistance. de Souza et al. found that GBM cells with high Nrf2 expression were more resistant to temozolomide chemotherapy. The expression of Nrf2 and its downstream ABCC1 in glioma patients’ tissues is positively correlated, which may be related to drug resistance and poor overall survival rate in GBM patients.^178^ Inducing ferroptosis may be an important therapeutic strategy to reverse drug resistance in GBM with high expression of Nrf2 and ABCC1. Blocking the synthesis of GSH can indirectly trigger ferroptosis. Ginkgetin,^179^ PRLX93936^180^ and falnidamol^181^ can reverse chemotherapy resistance of non-small cell lung cancer cells by inducing ferroptosis via Nrf2-GSH axis or DUSP26 pathway.

HCC is highly malignant, with a poor 5-year survival rate for patients with aggressive forms of the disease.^182,183^ Oxaliplatin is a third-generation platinum-based anticancer drug widely used in various cancers; however, resistance to oxaliplatin remains a significant clinical challenge in HCC treatment.^184,185^ Upregulated USP20 expression in oxaliplatin-resistant HCC cells is associated with poor prognosis, while genetic knockdown or pharmacological inhibition of USP20 can trigger ferroptosis and increase the sensitivity of HCC cells to oxaliplatin both in vitro and in vivo.^186^ Further results showed that the UCH domain of USP20 interacts with the N-terminal of SLC7A11, stabilizing SLC7A11 levels by removing the polyubiquitination.^186^ Most importantly, DNA damage-induced activation of ataxia-telangiectasia mutated- and Rad3-related protein (ATR) promotes Ser132 and Ser368 phosphorylation of USP20, enhancing its stability and conferring oxaliplatin- and ferroptosis-resistance in HCC cells.^186^ This suggests that targeting USP20 may mitigate chemotherapy resistance and promote ferroptosis in HCC patients, and explains why DNA damage therapy often leads to treatment resistance.

Cisplatin, as a main chemotherapy drug, is used in combination with other drugs or radiotherapy for PDAC treatment. However, the serious side effects and drug resistance of cisplatin also affect the clinical application. Dihydroartemisinin can induce ferroptosis in PDAC cells, and dihydroartemisinin combined with cisplatin can cause catastrophic accumulation of free iron and mitochondria-derived ROS in PDAC cells and lipid peroxidation, and impaired mitochondrial homeostasis,^187^ indicating ferroptosis can increase the cytotoxicity of cisplatin to PDAC cells and overcome PDAC cisplatin resistance. Cysteine is the rate-limiting amino acid for GSH biosynthesis. SLC7A11 mediates the uptake of cystine and influences GSH synthesis, therefore, suppression of the SLC7A11 has also been shown to trigger ferroptosis. Genetic and pharmacological inhibition of SLC7A11 induces ferroptosis in HNC cells by accumulation of lipid ROS, and enhances cisplatin cytotoxicity of cisplatin-resistant cells.^188^

LCN2 inhibits ferroptosis by enhancing GPX4 and SLC7A11 expression and reducing intracellular iron levels, and LCN2 overexpression in colon cancer cells leads to resistance to 5-fluorouracil (5-FU) by inhibiting ferroptosis. Inhibition of LCN2 function by monoclonal antibodies reduces chemotherapy resistance in colon cancer cells and tumors in xenografted mouse models.^189^ Additionally, the absence of N6-methyladenosine modification results in high expression of cyclin-dependent kinase 1 (CDK1) in oxaliplatin-resistant colon cancer cells and tissues. CDK1 directly binds and phosphorylates the S447 site of ACSL4, promoting the ubiquitination degradation of ACSL4, while genetic and pharmacological blocking of CDK1 enhances ACSL4 activity, promoting ferroptosis of colon cancer cells and increasing their susceptibility to oxaliplatin.^190^ These studies suggest that promoting ferroptosis is a promising strategy to overcome drug resistance in colon cancer patients.

Cancer stem cells (CSCs) are a subpopulation within solid tumors that exhibit resistance to conventional chemotherapy. DMT1 is a key protein regulating iron balance, and DMT1 inhibitors have been reported to selectively target CSCs in primary cancer cells and circulating cancer cells, causing ferroptosis in breast cancer CSCs and reversing multidrug resistance through lysosomal iron accumulation and ROS production.^191^

The resistance of gastric cancer to chemotherapy agents like 5-FU limits their clinical application, and the regulation of ferroptosis is closely related to gastric cancer progression and chemotherapy resistance. Signal transducer and activator of transcription 3 (STAT3) binds to DNA response elements in the promoters of ferroptosis-related genes (FTH1, SLC7A11, and GPX4) and regulates their expression. FTH1, SLC7A11, GPX4 and STAT3 are upregulated in 5-FU-resistant cells. Knockdown of STAT3 or use of the STAT3 inhibitor W1131 can induce ferroptosis through Fe^2+^ accumulation and lipid peroxidation in gastric cancer cells. This increases the sensitivity to chemotherapy in gastric cancer cells, and offers a novel treatment strategy for advanced gastric cancer and chemotherapy resistance.^192^ Similarly, inhibiting Nrf2/Keap1/SLC7A11 pathway to promote ferroptosis in gastric cancer cells also increases the sensitivity of gastric cancer to cisplatin.^193^

Cisplatin-based chemotherapy remains the primary treatment for advanced gastric cancer, however, chemotherapy resistance remains a significant challenge to clinical efficacy.^194,195^ Dysregulation of the Wnt/β-catenin pathway is closely linked to the development of gastric cancer and the emergence of chemotherapy resistance.^196^ When the Wnt/β-catenin pathway is activated, it can reduce lipid ROS production in gastric cancer cells, and the β-catenin/TCF4 transcription complex can directly bind to the GPX4 promoter region, enhancing its expression and thereby inhibiting cell ferroptosis. TCF4 knockdown can promote ferroptosis and increase the cisplatin sensitivity of gastric cancer cells,^197^ suggesting that abnormal activation of Wnt/β-catenin pathway increases ferroptosis resistance in gastric cancer cells, offering a possible therapeutic strategy for enhancing chemotherapy sensitivity in patients with advanced gastric cancer.

Cancer-associated fibroblasts (CAFs) interact with cancer cells through the secretion of exosomes, cytokines, and growth factors. LncRNA secreted by CAFs can enter tumor cells and influence tumor cell proliferation, metastasis, and drug resistance.^198,199^ Downregulated expression of LncRNA Dact3-As1 can promote the proliferation, invasion, and migration of gastric cancer cells,^200^ and is associated with poor prognosis in patients.^200^ Sequencing results indicate that Dact3-As1 in gastric cancer tissue might originate from fibroblasts, and Dact3-As1 enters gastric cancer cells via exosomes secreted by fibroblasts and induces ferroptosis by decreasing the levels of GPX4 and SLC7A11, thereby increasing the sensitivity of gastric cancer cells to oxaliplatin.^200^ ALOX15 is intricately linked to the generation of lipid ROS in gastric cancer cells, and miR-522, found in exosomes derived from CAFs within the tumor microenvironment, acts as an upstream inhibitor of ALOX15.^201^ The packaging of miR-522 into exosomes is mediated by heterogeneous nuclear ribonucleoprotein A1 (hnRNPA1) within CAFs, while USP7 regulates the stability of hnRNPA1 by preventing its ubiquitination. Notably, the administration of cisplatin and paclitaxel can enhance the secretion of exosomal miR-522 by activating the USP7/hnRNPA1 axis in CAFs. This process leads to the suppression of ALOX15 and a consequent decrease in lipid ROS accumulation in gastric cancer cells, potentially contributing to drug resistance.^201^

Patients with advanced pancreatic cancer who are resistant to chemotherapy often have a poor prognosis. Exosomes derived from CAFs in PDAC do not exhibit gemcitabine resistance, however, CAFs promote chemotherapy resistance in PDAC cells by secreting exosomes and maintaining signal exchange with PDAC cells after gemcitabine treatment.^202^ The mechanism may be related to miR-3173-5p in exosomes secreted by CAFs, which is taken up by PDAC cells and inhibits ferroptosis by downregulating ACSL4 expression.^202^

Reprogramming lipid metabolism can regulate the cellular antioxidant system and is associated with cancer cell metastasis.^203^ The formation of ovarian cancer spheres aids in overcoming the harsh microenvironment of nutrient deficiency and low oxygen in the peritoneal cavity.^204^ The ferroptosis inhibitor Fer-1 significantly enhances spheroids formation. Clinical data show that ACSL1 exhibits a positive correlation with FSP1, while it shows a negative correlation with 4-hydroxynonenal (4-HNE) and prostaglandin-endoperoxide synthase 2 (PTGS2).^205^ ACSL1 promotes peritoneal metastasis of ovarian cancer cells by reducing lipid peroxidation by lipid reprogramming. Mechanistically, ACSL1 increases N-myristoylation of FSP1, inhibiting its degradation and promoting its membrane transfer, thereby counteracting oxidative stress-induced cellular ferroptosis.^205^ Following carboplatin treatment, ACSL1/FSP1 activation inhibits ferroptosis and reduces the sensitivity of cancer cells to platinum chemotherapy, promoting cell survival and metastasis.^205^ Shikonin is one of the main natural naphthoquinone compounds in dried roots of *Lithospermum erythrorhizon*.^206^ Combined administration of shikonin and cisplatin increases Fe^2+^, ROS, and lipid peroxidation (LPO) levels and downregulates GPX4 in ovarian cancer cells, reducing cisplatin resistance by inducing ferroptosis.^207^

Platinum-resistant ovarian cancer cells and tissues are more likely to form spheroids and have increased level of the Wnt receptor frzzled 7 (FZD7). Overexpressing of FZD7 activates the oncogenic factor tumor protein 63 (TP63), leading to increased levels of glutathione metabolic pathways, including GPX4, which protects cells against chemotherapy-induced oxidative stress and ferroptosis.^208^ FZD7 knockdown improves the sensitivity to platinum, reduces the formation of spheroids, and inhibits tumor growth.^208^ GPX4 inhibitors also induce ferroptosis and increase chemotherapy sensitivity in FZD7^+^ platinum-resistant ovarian cancer cells. High expression of FZD7, TP63, and genes related to and GSH metabolism was also found in the ovarian cancer and residual human ovarian cancer samples after chemotherapy.^208^ The platinum-tolerant cell population may exhibit characteristics of cancer stem cells, and platinum-resistance may be related to the FZD7-β-catenin-Tp63-GPX4 pathway. Targeting ferroptosis offers a novel therapeutic strategy and insight for the treating platinum-tolerant cancer cells and potentially “persistent cancer cells”.^208^

Cholangiocarcinoma (CCA) is a malignant tumor originating in the epithelium of the biliary tract, characterized by a high propensity for metastasis and recurrence.^209^ F-box only protein 31 (FBXO31) functions as a substrate recognition protein in the proteome system, and the loss of FBXO31 promotes the occurrence and development of cancer.^210,211^ FBXO31 expression is downregulated in cholangiocarcinoma tissues, and its deficiency is associated with the pathological grade of CCA.^212^ FBXO31 enhances cisplatin-induced ferroptosis in cholangiocarcinoma cells and stem cell-like cells, and the mechanism may be related to increased GPX4 ubiquitination and proteasome degradation, which could be reversed by GPX4 overexpression.^212^ This suggests that FBXO31 functions as a tumor suppressor in CCA and modulates the sensitivity to cisplatin by promoting ferroptosis in bile duct cancer cells.

RAS mutations reduce the efficacy of anti-epidermal growth factor receptor (EGFR) monoclonal antibodies combined with chemotherapy in metastatic colorectal cancer patients.^213^ β-elemene, a bioactive compound extracted from the Chinese herb turmeric, has been shown to work synergistically with cetuximab in KRAS mutated CRC cells.^110^ Mechanistic studies have shown that combination therapy induces increased ROS and lipid peroxides and depletion of GSH, and downregulates ferroptosis inhibitory proteins, including FTH1, SLC7A11, GPX4, and FPN,^110^ suggesting that β-elemene is a novel inducer of ferroptosis. By triggering ferroptosis, β-elemene improves the susceptibility of KRAS-mutant CRC cells to cetuximab, suggesting a potential therapeutic option for RAS-mutated CRC patients. A member of the RAS superfamily, ADP ribosylation factor 6 (ARF6), acts as a downstream of the KRAS/extracellular signal-regulated kinase (ERK) pathway to increase the proliferation and Warburg effect of pancreatic cancer cells.^214^ ARF6 negatively regulates ACSL4 expression and regulates gemcitabine resistance by influencing the sensitivity of pancreatic cancer cells to oxidative stress and ferroptosis.^214^

Sulfapyridine is a commonly used drug with SLC7A11 inhibitory activity, can inhibit the proliferation of primary acute myeloid leukemia cells. Sulfapyridine significantly enhances the cytotoxicity of daunorubicin-cytarabine to leukemia cells, as confirmed in patient-derived xenograft models. This suggests that cystine import is a drug target in acute myeloid leukemia, and inhibition of cystine uptake can increase the sensitivity of leukemia to chemotherapy drugs.^215^

Oral squamous cell carcinoma (OSCC) has a high incidence and poor prognosis.^111^ Carnosic acid dose-dependently inhibits OSCC cell viability without obvious cytotoxicity to normal oral keratinocytes. Cisplatin-resistant OSCC cells have higher GSH levels, lower ROS, and lipid peroxidation, and exhibit certain resistance to ferroptosis.^216^ Carnosic acid enhances the sensitivity to cisplatin by decreasing cell viability and increasing ferroptosis, and this effect can be reversed by the ferroptosis inhibitor liproxstatin-1, suggesting that ferroptosis is involved in carnosic acid-mediated cisplatin resistance.^216^

### Ferroptosis in metabolic disorders

As a key regulation mechanism in metabolic diseases, the imbalance of glucose and fatty acid metabolism can directly or indirectly participate in ferroptosis and influence its occurrence and development. Common metabolic pathways involved in ferroptosis, such as (seleno) thiol metabolism, mitochondrial respiration iron handling, mevalonate pathway, and fatty acid metabolism, directly impact cellular sensitivity to lipid peroxidation and ferroptosis.^217^ Among these metabolic pathways, mitochondria, as the major organelles in iron utilization, involved in anabolic and catabolic pathways, play a crucial role in iron and energy metabolism. Ferroptosis is characterized by smaller mitochondria volume and condensed membrane densities compared to normal ones, diminished mitochondria crista and ruptured outer membrane.^218^ Mitochondria are pivotal in regulating signal transduction, cellular metabolism, and apoptotic signals. Their primary function is to generate energy through oxidative phosphorylation, but they are also essential for the metabolism off fatty acids, amino acids, iron and carbon.^219^ Mitochondrial dysfunction and altered metabolism driven by both intracellular and extracellular signals determine cell fate. A large body of evidence indicates that various cellular metabolic pathways such as amino acid, lipid, glucose, and iron metabolism, can promote ferroptosis. Ferroptosis-related molecules metabolic pathways can regulate glutathione state, cysteine exploitation, lipid peroxidation, nicotinamide adenine dinucleotide phosphate function and iron homeostasis.^220^ Therefore, it is highly important to explore the relationships among lipid and glucose metabolism, mitochondria metabolic function and ferroptosis in metabolic diseases such as diabetes, hyperlipidemia, and obesity.

#### Ferroptosis in diabetes

Since iron plays a key role in metabolic disease processes (such as glucose oxidation) and metabolic regulation (such as hypoxia sensing), it is involved in determining key energy metabolic links such as metabolic rate, gluconeogenesis, fuel selection, and insulin action. High iron levels are a risk factor for type 2 diabetes mellitus (T2DM) and impacts the cardinal features: such as increased insulin resistance, decreased insulin secretion and elevated hepatic gluconeogenesis. The risk of T2DM related to iron affects various cellular components, with beta cells, adipocytes, muscle, and liver playing central roles in determining diabetes phenotypes.^57^

The absolute or relative insufficiency of insulin secretion is a characteristic feature of all common forms of diabetes mellitus. One of the main causes of decreased insulin secretion capacity in diabetes is oxidative stress. Normal insulin secretion depends on the delicate redox balance of pancreatic islet β cells, which are particularly sensitive to ROS.^221,222^ ROS directly impair insulin synthesis and secretion.^223^ The classical pathway of cellular damage caused by iron overload is the production of lipid peroxides, but in islets, the process of ferroptosis is more complex and subtle.^222^ GPX4 is one of the main antioxidant protective enzymes in pancreatic β cells and plays an important role in reducing lipid peroxides and protecting β cells from ferroptosis in T2DM.^224^ The decrease in GSH and GPX4 leads to increased tissue damage in animal models of ferroptosis and diabetes.^225^ Meanwhile, this is also an important mechanism for the body to eliminate senescent β cells in type 1 diabetes.^226^

Although most uptake of cellular iron is mediated by TFR1, iron can be directly taken up by DMT1, which is required for β cells to package insulin during secretory granule formation.^227^ In pathological iron overload, this DMT1 iron transport process is aggravated by increased TFR1 saturation, leading to the generation of toxic NTBI, which then causes β cell damage through ferroptosis.^228^ There is other evidence shown that the coupling of insulin secretion to reduced glucose levels is also influenced by iron.^229^

Adipose tissue acts as an endocrine organ,^230^ producing key hormones such as leptin and adiponectin,^231,232^ and plays a crucial role in the pathogenesis of T2DM. Iron overload is a key negative regulator of leptin and adiponectin.^233,234^ Iron overload causes a loss of adipocyte-specific FPN, further leading to iron overload in adipocytes, reduced adiponectin levels, and insulin resistance.^233^ The association between, low adiponectin levels, insulin resistance and adipocyte iron overload were also been proved in a mitochondrial ferritin (FtMt) overexpression model.^235^ These findings provide evidence of increased ROS, decreased adiponectin, insulin resistance, and glucose intolerance due to adipocyte iron overload.

Iron overload exacerbates insulin resistance and increases hepatic gluconeogenesis.^236^ In addition, high dietary iron regulates the circadian rhythm of hepatic glycogen production through blood-mediated interactions between nuclear receptor subunit 1 group D member 1 and nuclear receptor auxiliary inhibitor 1.^237^ Hepatic glycogen synthesis is usually suppressed during normal feeding and enhanced during fasting. In experimental animal models and in humans, this rhythm disturbance is associated with T2DM, increasing the risk of diabetes in night workers.^238^

Due to the high energy requirements, both cardiac and skeletal muscles are rich in mitochondria but also influenced by oxidant stress and high rates of fuel oxidation. Mitochondrial oxidative stress caused by iron overload in muscle, which is a hallmark of insulin resistance. Fe-S clusters regulate iron homeostasis in mitochondria.^239^ Mitochondrial iron accumulation lead by Fe-S cluster deficiency can further induce iron-mediated ROS increasing and subsequent ferroptosis that due to lipid peroxides accumulation.^240^ Then ferroptosis causes β cells death and accelerates the development of T2DM.^241^ Because skeletal muscle is the key site of insulin-dependent glucose disposal, the iron homeostasis on glycemia might be vitally important.^242^ High iron levels can impair mitochondrial function and are related to heart failure, poor exercise capacity, and respiratory compromise.^243^

Dysregulated blood glucose levels may lead to complications in almost every tissue, especially the optical system, cardiovascular system and kidneys.^244^ In diabetic complications research, the activation of p53/SLC7A11/GSH axis causes endothelial cell ferroptosis under hyperglycemia, suggesting the important role of ferroptosis of endothelial dysfunction caused by diabetes.^245^ Ferroptosis is involved in diabetes-induced cognitive damage by altering FPN.^246^ After ischemia in diabetic patients, ferroptosis leads to cell death of cerebral microvascular endothelial cells.^247^ Ferroptosis combined with ER stress is also associated with diabetic myocardial ischemia/reperfusion (I/R) injury.^248^ Diabetic rats show significantly increased ROS content, decreased GPX4 expression, a decreased GSH/GSSG ratio, and significant retinal tissue damage and ferroptosis, which could be inhibited by ferroptosis inhibitor Fer-1.^249^

It has been shown that insulin secretion and sensitivity are improved and blood glucose is better controlled after reducing iron storage levels in the body.^250^ Iron chelation has been demonstrated to improve insulin action, to adjust hypoxia pathway signaling and to reduce inflammatory mediators levels in the adipocytes of obese diabetic mouse models.^251^ Similarly, iron chelation has been shown to enhance insulin sensitivity in the mouse liver.^252^ Diabetic model mice are protected by iron limitation, showing improved mitochondrial function and insulin secretion.^253^ DFO treatment indicates that iron inhibition prevents cognitive impairment and vascular remodeling after diabetic stroke.^254^ Cryptochlorogenic acid compounds derived from mulberry leaves have antidiabetic properties by inhibiting ferroptosis.^255^ During myocardial I/R in diabetic rats, Fer-1 inhibits ferroptosis and relieves ER stress and myocardial injury.^248^

A small number of clinical studies have found that antioxidant vitamin E, CoQ10, and alpha-lipoic acid combined with polarized light are effective in controlling T2DM complications, including diabetic foot.^256,257^ Oral vitamin C can promote diabetic foot ulcer wound healing by enhancing the reducing oxidative stress.^258^

Some studies have shown that natural herbal compounds, such as berberine, puerarin, keratotin, artemisinin, astragalus and others, have anti-diabetic clinical effects.^259^ However, evidence of these active components acting through the ferroptosis pathway is still limited to cell or animal experiments.

Although there is limited information on the role of ferroptosis in clinic,^249,260,261^ it is accepted that ferroptosis inhibition may prevent diabetes and its complications. Almost all antioxidant genes involved in ferroptosis, such as NADPH regeneration, glutathione-regulated and lipid peroxidation, may be potential therapeutic targets.^260,262^

#### Ferroptosis in hyperlipidemia

Lower GPX4 activity and the massive lipid peroxides accumulation are the main reasons of ferroptosis. Lipids, as essential components of cell membranes play central roles as signaling molecules and regulators of most cellular functions.^263^ Fatty acid metabolism maintains normal energy metabolism and life activities by enhancing lipid synthesis, storage, and catabolism. Therefore, studies on cellular fatty acid metabolism, membrane fatty acid composition, and ferroptosis, while limiting lipotoxicity, are gaining increasing attention from researchers.^264^ The execution of ferroptosis is mainly driven by iron-dependent phospholipid peroxidation. Thus, ferroptosis and other cellular processes involving phospholipid peroxidation can be regulated by controlling phospholipid peroxidation.^8^ Lipid peroxidation associated with ferroptosis initiates on internal membranes and subsequently appears at the plasma membrane, leading to ion imbalances and increased membrane permeability. The vulnerability to ferroptosis is controlled by the oxidation of polyunsaturated lipids and the activity of related lipid metabolism enzymes. Diverse enzyme networks and endogenous metabolites inhibit lipid peroxidation by disrupting its onset or progression. This understanding provides insight into disease treatment by regulating lipid metabolism to either enhance or inhibit ferroptosis.^265^

Iron overload has been found to decrease lipoprotein lipase activity, thus promoting the development of hypertriglyceridemia.^266,267^ Meanwhile, serum ferritin is a major determinant of the lipid phenotype in both familial hyperlipidemia and familial hypertriglyceridemia. The positive correlation coefficient between ferritin and triglycerides is the highest among all the factors investigated.^268–270^ Hyperlipidemia, caused by high-fat diets and other secondary factors, is one of the metabolic syndromes. It mainly includes increased concentrations of total cholesterol and/or triacylglycerol, as well as decreased concentrations of high-density lipoprotein cholesterol, which is one of the independent risk factors for cardiovascular and cerebrovascular diseases.^271^ Therefore, more studies have focused on the progression and related mechanisms of ferroptosis in various tissues and cells under the high-fat conditions.

Free fatty acids (FFAs) serve as the body’s lipid fuel, and excess FFAs can cause insulin resistance, endothelial dysfunction, and inflammation. Elevated plasma FFA levels are strongly linked to cardiometabolic risk factors in metabolic syndrome, obesity, and diabetes, which also involve the regulation of key targets in the ferroptosis pathway. Lipid accumulation in L02 cells after FFA treatment could cause increased ROS, mitochondrial damage, and ferroptosis. Di Dang Decoction, a traditional Chinese prescription for treating hyperlipidemia, has been found to upregulate HIF-1α and GPX4 expression, thereby alleviating ferroptosis.^272^ The increase in FFAs contributes to cellular lipotoxicity. In diabetic patients, increased expression of peroxisome proliferator-activated receptor α (PPARα) is related to higher uptake of FFAs. Cluster differentiation protein 36 (CD36) also induces FFA absorption, leading to increased cardiac lipotoxicity in cardiomyocytes. Insulin resistance induces CD36 upregulation, promoting the accumulation of inflammation and lipids, worsening myocardial metabolic disorders. Insulin resistance stimulates AMPK (an energy-sensing enzyme in low energy), increases FFAs intake in the heart via CD36. It then inhibits adipocyte lipolysis and increases FFAs in cells, further stimulating the LDLs accumulation and secretion, inducing lipid metabolism disorders, lipotoxicity, and even cell necrosis, finally resulting in diabetic myocardial damage.^273^

Using the lipid overload oxidative stress-related calcification model, it was found that the downregulation of p53 expression suppressed GPX4 and SLC7A11 expression and decreased the GSH/GSSG ratio, thereby promoting lipid peroxidation and ferroptosis. Overexpression of p53 counteracts ferroptosis and induces a rebound in GPX4 and SLC7A11 expression. Energy stress-mediated AMPK activation and the activation of the Nrf2 antioxidant pathway also play important roles in regulating ferroptosis, through which metformin could alleviate ferroptosis and vascular calcification in high-lipid model mice.^274^ Additionally, using the above model, it was found that palmitic acid (PA) induced ferroptosis through mitochondrial DNA damage activation, resulting in calcification damage of vascular smooth muscle cells. Overexpression of PPARα contributes to reducing PA-induced ferroptosis; oleoylethanolamide inhibits ferroptosis induced by hyperlipidemia and mitochondrial DNA damage, improving vascular sclerosis.^275^ Oxidized LDL was found to induce ferroptosis by inhibiting the activity of GPX4, and anti-ferroptosis treatment showed good therapeutic effects.^276^

In addition, lipid metabolism disorders caused by high lipid levels lead to the dysregulation of GSH and ferroptosis of vascular smooth muscle cells. Echinatin significantly promotes the expression of glutamate cysteine ligase, maintaining the balance of GSH metabolism.^277^ Scavenger receptor class B type I (SRBI) plays a crucial role in promoting cholesterol efflux from cells and clearing plasma cholesterol, while SRBI deficiency results in disrupted cholesterol metabolism, hyperlipidemia, and subsequent ferroptosis via the HIF-1α/TFR1 pathway.^278^ The hepcidin-FPN axis is essential for maintaining systemic iron homeostasis Increased serum hepcidin levels are associated with ferroptosis. Hyperlipidemia increases serum hepcidin, which can be inhibited by the intervention of natural compound tetramethylpyrazine (TMP), and TMP may lower hepcidin expression by inhibiting the STAT3 signaling pathway.^279^

These pathways and targets, among others, involve intracellular lipid accumulation and excessive oxidation, eventually leading to cell decline and even death via ferroptosis. These evidence, while observed in cardiomyocytes and vascular smooth muscle cell metabolism, is also applicable to explain other important system functional imbalance in the lipotoxic state of high FFAs. For instance, hypertriglyceridemia was induced under high-fat diet conditions and then cerulein was then injected to establish hypertriglyceridemic pancreatitis (HTGP) models. Using this model, it was found that a high-fat diet could aggravate the ferroptosis process and, together with ER stress, worsen the development of pancreatitis. Liprostatin-1 treatment can inhibit ferroptosis through the lipid metabolism pathway and further resist the activation of ER stress-related proteins, thus reducing pancreatic damage. It was also demonstrated that lipid metabolism promoted ferroptosis in the HTGP process by regulating ACSL4/LPCAT3 levels, and that ER stress may be involved in ferroptosis through the BIP/p-EIF2α/CHOP pathway.^280^ Puerarin inhibits liver ferroptosis and inflammation in hyperlipidemic mice induced by the combination of a high-fat diet and PA, and improves insulin sensitivity and glucose tolerance in test mice, and alleviates liver dysfunction and hyperlipidemia.^281^ Grape seed extract can exert antioxidant action against ferroptosis and protect lung tissue in high fat-fed mice.^282^

#### Ferroptosis in obesity

Obesity is widely accepted as a global health problem which caused by a long-term energy imbalance due to excessive caloric intake and inadequate energy expenditure. Excessive energy intake with lack of physical activity are traditional risk factors for obesity. Recent studies have shown a strong association between ferroptosis and obesity.^283^

Obesity is closely related to iron metabolism disorders. Common mechanisms of ferroptosis including iron overload, lipid peroxidation, GPX4 inhibition, and system Xc^−^ inhibition in obesity, which lead to decrease of GSH, increased ROS, and even ferroptosis. These changes induce insulin resistance and mitochondrial dysfunction, eventually leading to metabolic disorders and obesity. Increasing evidence suggests that ferroptosis plays a key role in obesity and its complications. Ferroptosis directly affects adipose tissue and indirectly promotes the development of obesity through inflammation and insulin resistance caused by regulatory disorders in the liver and immune system. Indeed, ferroptosis forms a complex regulatory network with obesity. Key proteins involved in iron absorption, transport, storage, and regulation of iron balance are also potential targets for intervention.^284^

Previous reports have found that obese adult women are more likely to have low iron than nonobese ones.^285^ Obese patients who have chronic inflammation are more likely to suffer hypoferritinemia.^286^ The reason may be related to the hepcidin imbalance levels in obese women. Adipose tissue can directly express hepcidin and can also indirectly regulate the expression of hepcidin through the levels of leptin and proinflammatory cytokines to affect iron balance.^287^ Recent studies have shown that in obese individuals, increased fat is associated with iron homeostatic imbalance manifested as elevated ferritin, elevated serum hepcidin, and increased risk of iron overload. Reduced serum ferritin has been proven to improve symptoms of obese patients.^288^ The mRNA levels of hepcidin in obese mice^289^ and patients^290^ were shown significantly increased in adipose, and this increasing was related to IL-6 and other inflammatory factors. It is generally believed that TNF-α, IL-6, and leptin are involved in iron metabolism by regulating the hepcidin expression in obesity.^287^ The identification of genes associated with ferroptosis has also shown that obesity has higher expressions of ferroptosis genes in adipose tissue. Remarkable differences in nuclear factor kappa-B (NF-κB) activity, cytokine production, leukocyte migration that involved in the inflammatory response, and other biological targets (such as NCOA4, glutamate-cysteine ligase catalytic subunit and Aldo-keto reductase family 1-member C1) are also observed when compared to those in controls.^291^ Hypoxia can also cause ferroptosis, redox dysregulation, and inflammation in adipose tissue, partly due to iron overload.^292^

Deficiency of skeletal muscle cystathionine γ-lyase leads to insulin resistance, ferroptosis, and obesity, resulting in hyperglycemia and skeletal muscle injury in high-fat diet mice.^293^ In obese mice, there is a marked increase in VEGF, and mammalian target of rapamycin (mTOR), oxidative stress, and ferroptosis within primordial follicles.^294^

Cellular and animal experiments have found that long-chain fatty acids cause excessive oxidation of adipocytes, producing inflammatory cytokines, facilitating macrophage infiltration, and leading to systemic low-grade inflammation. GPX4 protects adipocytes from lipid peroxidation, oxygen-specific epitope accumulation, and ferroptosis.^295^ Alpha-linolenic acid reduces fat accumulation and ferroptosis by regulating 1-acetylglycerol-3-phosphate-acetyltransferase 2, thereby reducing cognitive impairment and liver damage in obese mice fed a high-fat diet.^296^ 5-hydroxytryptamine reduces the weight of high-fat diet-fed zebrafish, showing a significant correlation with the gene expression of ferroptosis in skeletal muscle of zebrafish.^297^ Nuciferine, an active ingredient derived from lotus leaf, shows potential for the treatment of obesity and related diseases. The mechanism involves multiple complex pathways, including ferroptosis signaling.^298^

Age-related changes in the metabolism of energy substances are thought to cause obesity. Aging and changes in substance metabolism associated with obesity may aggravate ferroptosis. For example, abnormal iron contents, significantly reduced expression of branch-chain aminotransferases during aging, increased plasma triglycerides, and disturbed metabolism of fat, amino acids, and iron all contribute to cytotoxicity, energy disorders, and ultimately ferroptosis. Aerobic training can improve fatty acid oxidation, enhance muscle metabolism, regulate insulin sensitivity, and improve amino acid and iron homeostasis, thereby mitigating the adverse effects of ferroptosis.^299^ Electroacupuncture has effects on weight loss via activating the Nrf2 pathway, inhibiting inflammation and ferroptosis in intestine caused by high-fat diet-induced obesity.^300^

#### Ferroptosis in metabolic dysfunction-associated fatty liver disease

Metabolic dysfunction-associated fatty liver disease (MAFLD), previously known as non-alcoholic fatty liver disease (NAFLD), represents a significant global health concern, associated with high incidence and high mortality rates. MAFLD is defined as hepatic steatosis with metabolic dysregulation, type 2 diabetes mellitus, overweight or obesity, and is characterized by extensive liver lesions, which can progress from isolated steatosis to metabolic dysfunction-associated steatohepatitis (MASH), liver fibrosis, and even HCC.^301^

Emerging evidence suggests a link between ferroptosis and MAFLD progression.^302^ Elevated hepatic iron levels may lead to MAFLD and promote the progression of the disease, with excessive iron content may increase hepatocyte swelling, inflammation, and fibrosis, potentially converting isolated steatosis to MASH.^303^ Moreover, hepatic iron accumulation can induce the transcription expression of ACSL4 by activating the transcription factor c-Myc, and further aggravate the development of MASH through ferroptosis. Therefore, the lipids accumulation, infiltration of immune cells, and activation of hepatic stellate cells in hepatocytes can induce ferroptosis, thus promoting MAFLD progression.

Bioinformatics analysis have identified associations between hepatic steatosis and ferroptosis-related genes such as AKR1C1, AKR1C2, ACSL3, ACSL4, CS, GSS, FADS2, and PGD.^304^ In MASH patients, ferroptosis-related genes like SLC40A1, ACSL5, CP, and SLC11A2 are downregulated, while ACSL4, ACSL6, ferritin light chain (FTL), and FTH1 are upregulated.^302^ This suggests that MAFLD progression involves iron overload, an imbalance in antioxidant defenses, and lipid peroxidation within in the ferroptosis signaling pathway. SLC7A11 is involved in the uptake of cystine, and the expression level of SLC7A11 in MAFLD patients is directly correlated with clinical grade. Both SLC7A11 knockdown or overexpression can induce ferroptosis in hepatocytes of MAFLD model mice, but the mechanisms are different. SLC7A11 deficiency accelerates the progression of high-fat diet-induced MASLD through ferroptosis induced by classical cystine/cysteine deficiency, while serine deficiency and consequent obstruction of de novo synthesis of cysteine are responsible for the progression of ferroptosis induced MAFLD in hepatic SLC7A11 overexpressing mice.^305^ These results suggest that liver cells require a narrow window of SLC7A11 activity to maintain healthy liver function when exposed to a high-fat environment.

Studies have found that secondary lipid peroxidation products, such as MDA and 4-HNE, are significantly higher in MASH patients compared to those with simple hepatic steatosis.^306^ Ferroptosis has also been implicated in liver fibrosis, with ferroptosis in hepatic cells promoting liver fibrosis.^307^

The interaction between hepcidin and FPN is also an important mechanism for iron homeostasis. In the case of TFR1 deficiency, SLC39A14 mediates NTBI entry into hepatocytes. Recent studies have found that SLC39A14 promotes hepatocyte ferroptosis through the delivery of NTBI.^20,287^ Deleting SLC39A14 in TFR knockout mice significantly reduces hepatic iron accumulation, and inhibits ferroptosis-mediated liver fibrosis in high iron-diet mice. Conversely, upregulation of SLC39A14 in hepatocyte-specific TFR knockout mice leads to ferroptosis due to excess iron, making them more prone to liver fibrosis.^20^

Fatty acid binding protein 5 (FABP5) has been identified as a driver of obesity-induced HCC. Genetic ablation and/or pharmacological inhibition of FABP5 ameliorate the HCC burden in mice.^308^ The absence of EF-hand domain family member D2 (EFHD2), improves hepatic steatosis, reduces immune cell infiltration, suppresses ferroptosis caused by lipid peroxidation, and ultimately reduces MASH. A stapled α-helical peptide target to EFHD2 has been shown have effect on protecting MASH in mice.^309^

Many ferroptosis inhibitors and inducers have been identified for studying ferroptosis in MAFLD. Iron chelators like deferasirox (DFX) and deferiprone (DFP) are used to treat iron overload-related diseases.^310–312^ DFO and artesunate also act as iron chelators to inhibit ferroptosis.^313,314^ DFP, with high membrane permeability, greatly inhibit ferroptosis and improves liver injury.^315^ A novel iron chelator, FerroTerminator1(FOT1), has demonstrated potent iron scavenging capacity and a good safety profile, effectively reversing liver injury in multiple MASH models without notable toxic side effects.^316^

Ferroptosis inducers such as erastin and RSL-3 exacerbate apoptosis and hepatic steatosis in MAFLD mice. While the ferroptosis inhibitors liprostatin-1 and Fer-1 can reduce MASH severity by inhibiting ferroptosis.^317,318^ Fer-1 is a putative drug that suppresses ferroptosis, reducing oxidative damage to the hepatocyte membrane mediated by MAFLD by inhibiting hepatocyte ferroptosis.^317^ Researchers have found that Fer-1 binds to 15LOX/phosphatidylethanolamine binding protein-1 (PEBP1) complex, suppressing the generation of peroxidized eicosatetraenoyl-phosphatidylethanolamine (ETE-PE) and protecting against ferroptosis.^319^

Thymosin β4 alleviates MAFLD by inhibiting ferroptosis through GPX4 upregulation.^320^ Additionally, targeting a novel inducible GPX4 isoform could also mitigate ferroptosis and may be used to treat metabolic-associated fatty liver diseases.^321^ Some existing drugs are being repurposed to treat MAFLD by promoting or inhibiting ferroptosis. Vitamin E, a lipid-soluble basic antioxidant, reduces oxidation levels of membrane proteins and significantly lowers the number of necrotic cells and the levels of serum markers of liver injury in early-stage MASH.^315^ Vitamin E diet enhances survive rate of hepatocyte-specific GPX4^−/−^ mice, which is associated with the improvement of hepatocellular degeneration by inhibition of ferroptosis.^322^ Vitamin E improves hepatic inflammation and steatosis but not fibrosis in MAFLD patients.^323,324^

Rosiglitazone has been shown to enhance liver function in individuals with MAFLD irrespective of the presence of diabetes.^325,326^ This improvement is achieved through the inhibition of ferroptosis by downregulating ACSL4 expression via a PPAR-γ-independent pathway. Knocking down ACSL4 also remarkably reduces the levels of 5-hydroxyeicosatetraenoic acid (5-HETE) thereby reducing arsenic-induced MASH.^302,327^ Another study demonstrated that troglitazone inhibits ferroptosis induced by erastin.^317^ However, no clinical trials have been conducted to evaluate the therapeutic effects of troglitazone and/or rosiglitazone on MAFLD through inhibiting ferroptosis.^302^

Diethyl fumarate (DMF), an activator of Nrf2, has been reported to reduce lipid peroxidation and inhibit hepatocyte ferroptosis, thereby ameliorating alcoholic hepatitis.^328^ Dehydroabietic acid, a tricyclic divalent resin acid isolated from coniferous plants, offers many benefits for the human body, including anti-tumor, antibacterial, anti-aging, and anti-inflammatory.^329,330^ It has been reported that dehydroabietic acid binding to Keap1 in hydroxide-induced MAFLD mice, promoting the expression of GSH, GPX4, and HO-1, thereby reducing ROS and MDA accumulation. Moreover, dehydroabietic acid increases the expression of key ferroptosis genes such as FSP1.^331^ Ginkgo biloba B, a principal component of Ginkgo biloba extract, reduces hepatic lipid accumulation and ameliorates MAFLD in obese mice. Ginkgo biloba B treatment has an inhibitory effect on lipid accumulation and ferroptosis caused by oxidative stress in MAFLD, possibly through the activation of the Nrf2 pathway, exerting an antioxidant effect.^332^

In addition, puerarin is known to improve MAFLD by inhibiting inflammation and ferroptosis.^281^ Melatonin inhibits the stress effect of ER through the melatonin receptors type 1B (MT2)/cAMP/protein kinase A (PKA)/inositol-requiring enzyme 1 (IRE1) pathway, reducing hepatic ferroptosis in MAFLD.^333^ Liraglutide alleviates type 2 diabetes mellitus-associated MAFLD by activating AMPK/acetyl-CoA carboxylase (ACC) pathway and inhibiting ferroptosis.^334^ Supplementation with Icariin reduces ferroptosis markers and attenuates the progression of MASH in mice fed a methionine choline-deficient diet.^335^ Zeaxanthin suppresses ferroptosis by inhibiting the p53 pathway and enhancing mitochondrial function in FFA-induced HepG2 cells.^336^ Enoyl coenzyme A hydratase 1 helps alleviates MASH in mice by suppressing hepatic ferroptosis.^337^ Additionally, fibroblast growth factor 21 mitigates liver fibrosis and injury induced by iron overload by preventing ferroptosis in hepatocytes.^338^

### Ferroptosis in autoimmune diseases

Autoimmune diseases, such as rheumatoid arthritis (RA), systemic lupus erythematosus (SLE), and myasthenia gravis (MG), are caused by disorders in autoimmune tolerance and the body’s immune response to the self-antigen. Influenced by hormones, immunomodulators and metabolic factors, these autoimmune diseases exhibit features of ferroptosis features, including iron metabolism disorder and lipid peroxide accumulation at different stages of development, suggesting that ferroptosis plays an important regulatory role in the onset and progress of autoimmune diseases.

#### Ferroptosis in rheumatoid arthritis

RA is a multifactorial autoimmune disorder which characterized by the bone and cartilage destruction and synovial hyperplasia, thereby leading to joint pain, swelling and stiffness. In the pathogenesis of RA, activated fibroblast-like synoviocytes (FLSs) display proliferative properties similar to those of tumor cells, causing cartilage erosion and ultimately joint destruction. The initial symptoms of RA often include pain in the finger and wrist, while with the disease develops, it can affect large joints including the shoulder, knee and so on, resulting in joint mobility limited, joint deformity, and even disability. RA may also cause systemic multisystem injury and extra-articular manifestations.^339,340^

Ferroptosis is a crucial regulator of the inflammatory response, and it has been implicated in the onset and development of various inflammatory arthritis, such as RA.^1^ Disordered iron metabolism contributes to RA development through mechanisms such as inducing oxidative stress, induction of inflammatory responses, and impairment of immune cell function. Therefore, the ferroptosis fundamental characteristics, that is, GPX4 inactivation, GSH depletion, iron deposition, and lipid peroxidation, have garnered attention in the context of RA pathogenesis.^341^ Gene regulatory network analysis based on FLSs, a key target of damage in RA disease, has identified major transcription factors involved in fatty acid metabolism and ferroptosis.^342^

Iron is avital regulator of the immune response, and its metabolism is important in autoimmune diseases, including RA.^1^ Iron overload profoundly affects the immune system by inhibiting the phagocytosis of macrophages and monocytes, increasing the number and viability of inhibitory T cells, limiting the proliferative ability of T helper cells, and changing the distribution of lymphocytes within the immune system.^343^ Moreover, excessive iron accumulation sustains inflammation.^344^ Iron overload may trigger an inflammatory response through activating the NF-κB pathway and inducing the secretion of inflammatory factors such as IL-1β, TNF-α, and IL-6.^345^ Patients with RA usually have elevated iron metabolism markers, such as serum ferritin and iron levels, which correlate with the severity of joint inflammation.^346,347^ Moreover, animal experiments have also found that synovial inflammation in RA can be aggravated by intravenous iron.^348^

In RA, inflammatory activation with excessive ROS further damage the synovium, vessels, and joints. Moreover, it promotes the secretion of macrophage colony-stimulating factor (MCSF) and NF-κB receptor activator (RANKL), which contribute to bone destruction.^349^

Meanwhile, RA patients typically have lower antioxidants, such as GSH and GSH peroxidase, and treatment with methotrexate significantly changes oxidative stress indicators in RA patients.^350^ The Nrf2-associated antioxidative stress is also closely associated with ferroptosis inhibition. Activation of Nrf2 can prevent inflammatory damage and oxidative stress in the synovium via transcribing antioxidant enzymes, such as HO-1, superoxide dismutase, and GSH.^351^ Nrf2 activation not only inhibits ROS, but also inhibits inflammation-related signaling molecules and the proliferation and migration of FLSs, thus alleviating RA synovitis effectively.^352^

Macrophages, which have antigen-presenting and phagocytic properties, play a key role in RA synovitis. Iron overload promotes M1 macrophage polarization and increases the M1 macrophage markers levels (IL-6, IL-1, and TNF-α), leading to synovial damage.^45^

Ferroptosis modulates the activity of cytotoxic T cells (CD8) and helper T cells (CD4). In vitro experiments have shown that GPX4 or FSP1 overexpression protects CD8^+^ T cells from ferroptosis, while application of GPX4 inhibitor significantly increases the sensitivity of T cells to ferroptosis.^353^ Interestingly, synovial inflammation is a key event in the RA pathogenesis and a critical trigger for destructions of bone and cartilage. The synovium primarily consists of FLSs and immune cells. Under oxidative stress conditions, FLSs exhibit abnormal proliferative capacity and invasive potential resembling tumor-like growth.^354^

Given these factors, targeting ferroptosis could be a potential strategy for the prevention and treatment of RA. Both ferroptosis inducers and inhibitors may reduce inflammation and then inhibit joint damage. However, due to the bidirectional regulation mechanism of ferroptosis, targeted therapy for ferroptosis is still in its infancy.^355^

The use of iron chelators in mice has been shown to reduce iron accumulation and ameliorate oxidative damage and immune dysfunction caused by iron overload effectively.^356^ Targeting ferroptosis in RA-FLSs can improve RA outcomes. Glycine induces ferroptosis in RA-FLSs via S-adenosylmethionine (SAM)-associated methylation of GPX4 promoter, further enhancing ferroptosis efficacy by downregulating FTH1 expression.^357^ P53, one of the frequent mutated tumor-suppressing genes, is increased in activated RA-FLSs.^358^ In addition, ferroptosis caused by sulfasalazine has a dual role. On one side, sulfasalazine inhibits cystine uptake and decreases GSH and GPX4 levels by inhibiting the system Xc^−^. On the other side, sulfasalazine induces a Fenton response, producing excessive ROS in lipid and promoting ferroptosis.^359^

Antioxidants also modulate ferroptosis in RA. The use of antioxidants including selenium, vitamin C, and vitamin E, has shown prospective effects when used to alleviate the symptoms of RA.^360^ CoQ10 can regulate IL-17 and TH17 via the STAT3 pathway, then inhibit ferroptosis and improve inflammation in joint of RA mice.^361^ Moreover, anti-inflammatory and the antioxidant effects of natural polyphenols (e. g. quercetin, curcumin, rutin, and resveratrol) are well-documented. SIRT1, a target in the inflammatory response of articular chondrocytes is activated by curcumin, which protects chondrocytes by inhibiting MMP-13 and NF-κB expression.^362^ Pomegranate-derived polyphenols alleviate RA by inhibiting the activation of MMP-13, simultaneously blocking the anti-inflammatory signaling pathway, and activating the antioxidant defense system in chondrocytes.^363^ Furthermore, icariin has been found to inhibit ferroptosis in synovial cells and exert a protective effect through activation of the Xc^−^/GPX4 axis.^364^

Emodin can relieve collagen-induced joint inflammation in rats by inhibiting ferroptosis and degrading MMP.^365^ Large leukotrienes relieve RA via the Nrf2/HO-1/GPX4 pathway.^366^ Haloperidol affects T lymphocytes in RA patients by altering the DNA replication program, DNA damage response, and ferroptosis.^367^ Moxibustion improves the inflammatory response in synovial tissue of RA model rats, potentially through its regulation of ferroptosis-related factors. Moxibustion can downregulate p53 and ROS, upregulate SLC7A11, GPX4 expression, and reduce ferroptosis.^368^

Given the dual-edged role of ferroptosis in RA, further scientific exploration is required to the understand the underlying mechanisms of ferroptosis in RA and their pharmacological interventions.

#### Ferroptosis in systemic lupus erythematosus

SLE is a unique autoimmune disease characterized by the excessive production of multiple autoantibodies against the cellular components of the nucleus, cytoplasm, and cell membrane. These autoantibodies form immune complexes that deposit in various tissues and organs, leading to organ damage. The induction and intensification of cell death, along with barriers to macrophage clearance and absorption of dead cells, increase the exposure of intracellular contents to the immune system, triggering an immune response. A decrease in immune cells (e. g., neutrophils, lymphocytes, red blood cells, and platelets) is common in SLE patients. Notably, neutrophil death is often accompanied by the release of neutrophil extracellular traps (NETs), which activate Toll-like receptors and induce ROS production. In addition, this process can also stimulate the release of interferon from plasmacytoid dendritic cells, causing a severe inflammatory response and further cell death.^369^ Recent studies have demonstrated that neutrophil death in SLE involves ferroptosis, which is characterized by morphological changes such as vacuole formation, disappearance of mitochondrial cristae, and increased membrane density in mitochondria.^370^

In SLE patients, GPX4 expression is notably diminished in neutrophils but remains unchanged in other immune cells. Myeloid-specific GPX4-haploinsufficient mice develop a lupus-like disease spontaneously, but complete GPX4 ablation in neutrophils results in severe neutropenia without inducing a lupus-like disease.^370^ This evidence highlights the critical role of neutrophils in the pathogenesis of SLE. Treatment with liproxstatin-1, a ferroptosis inhibitor, significantly reduces the progression of SLE.^371^

In addition to neutrophils, studies have shown that inhibitors of ferroptosis can regulate disease progression in SLE mouse models by modulating the TH_1_/TH_2_ ratio.^372^ Ferroptosis also occurs in peripheral blood monocytes in SLE, with electron microscopy revealing characteristic ferroptosis features, such as decreased mitochondrial volume, increased mitochondrial membrane density and the disappearance of mitochondria.^373^

Erucic acid has been found to improve pregnancy outcomes in SLE patients by inhibiting the function of CD8^+^ T cells. Mechanistically, erucic acid orchestrates this inhibition by impeding STAT3 phosphorylation and promoting ferroptosis.^374^

#### Ferroptosis in Myasthenia gravis

MG is a rare, chronic autoimmune disorder characterized by autoantibodies targeting receptors at the postsynaptic membrane of the neuromuscular junction. These autoantibodies target LDL receptor-associated protein 4, muscle-specific kinases, and the acetylcholine receptor (AchR). The binding of these antibodies to postsynaptic receptors, subsequently, complement activation and membrane attack complex formation, results in reduced expression of AchR. These alterations hinder normal neuromuscular signaling, ultimately leading to the onset and development of MG.^375^ The clinical features of MG include muscle weakness and fatigue such as ptosis, diplopia, and systemic muscle damage. Recent evidence suggests that ferroptosis may play a role in the pathogenesis of MG.^376^

Ferroptosis has been associated with the development of muscular dystrophy.^377^ Indicators of iron metabolism might be useful for assessing disease severity and monitoring clinical efficacy in MG patients.^378^ Some studies have shown decreased serum iron levels in MG patients, with an inverse correlation between serum iron content and IL-6 levels and anti-AchR antibodies. The negative correlation of serum iron content with muscle mass provides new perspectives on preventing and treating muscle loss.^379^ Elevated iron content promotes ROS production, reduces protective autophagy, and causes skeletal muscle death.^380^ Deficiency of TFR1 in skeletal muscle results in abnormal blood iron and lipid metabolism, impairing muscle function.^381^ Iron chelation therapy with intravenous DFO has been shown to be an effective treatment for MG.^382^

Iron imbalance not only promotes oxidation reaction, but also leads to mitochondrial dysfunction.^383,384^ Ferroptosis inhibitors targeting various pathways can reduce mitochondrial morphological changes and lipid peroxidation in muscle cells. Research has shown that enhancing mitochondrial function in muscle can be beneficial for treating muscle-related diseases. Drugs targeting mitochondria have shown high efficacy in managing skeletal muscle-related diseases. Mitoquinone (MitoQ), a mitochondria-targeted antioxidant, helps regulated energy metabolism in skeletal muscles.^385^ Mito-TEMPO (MT), another mitochondrial superoxide scavenger, also prevents muscle atrophy via alleviating mitochondrial dysfunction, oxidative stress and inflammatory.^386^ Increasing levels of nicotinamide adenine dinucleotide (NAD^+^) precursors can improve symptoms of acquired muscle dysfunction by activating mitochondrial metabolism.^387^

Recent studies show that nanodrug delivery systems can enhance the bioavailability and targeting of anti-ferroptosis drugs. By carrying antioxidants or iron chelators, nanodrug systems targeting mitochondrial ferroptosis can be used as promising agents for treating MG.^376^ For example, extracellular vesicles inhibit the Th17 and germinal center response, improving experimental autoimmune MG by targeting macrophages.^388^ Anti-inflammatory nanoparticles have been shown to enhance muscle function in a mouse model of advanced muscular dystrophy.^389^ Liposomal steroid nanomedicine reduces macrophage infiltration and attenuates serum TGF levels in a mouse Duchenne muscular dystrophy model.^390^ The introduction of nanogolds can assist in the recovery of muscle function in M2 macrophage-polarized mice.^391^ However, more research and direct evidence are needed for MG intervention.

### Ferroptosis in genetic disorders

Genetic diseases are caused by changes in genetic material, such as genes mutations or chromosomal abnormalities. Approximately 4000 genetic diseases have been identified, and over 100 kinds of genetic diseases are newly discovered every year, posing serious threats to human health. Iron overload is a common complication of genetic diseases such as thalassemia and Wilson Disease.

#### Ferroptosis in hemochromatosis

HH is an iron overload disorder resulting from mutations in genes including HFE, SLC40A1 (FPN), TFR2, and HJV. It is among the most widespread genetic diseases in Europe.^392,393^ Iron accumulates in the parenchymal cells of various organs in HH patients, generating ROS through the Fenton reaction, resulting in ferroptosis and several serious chronic complications, including heart disease, diabetes, and cirrhosis.^394^ Mice fed a high iron diet as well as those with inherited hemochromatosis (Hjv^−/−^ and Smad4^Alb/Alb^ mice) all developed systemic iron overload due to decreased hepcidin expression in liver.^27,395^ In these mice, liver NADPH contents drop significantly while MDA and mRNA levels of Ptgs2 increase. These changes can be reversed by Fer-1, indicating that iron overload induced ferroptosis in HH mice, and suggesting targeting ferroptosis could be a promising strategy for treating hemochromatosis-related injury.^396^

Hepcidin, a hormone produced by the liver, controls iron absorption and distribution in tissues, and lack of hepcidin is the primary cause of iron overload in almost all types of hereditary hemochromatosis.^397,398^ Minihepcidins are small engineered peptides that produce iron-limiting effects like the hepcidin hormone. Compared to natural hepcidin, Minihepcidin PR65 has advantages such as low cost, high titer, and long half-life. Subcutaneous injection of PR65 in iron-depleted hepcidin knockout mice blocks iron overload in the liver, reduces iron levels in the heart, and causes iron retention in the spleen and duodenum. In iron-loaded hepcidin knockout mice, PR65 injections have a milder effect, resulting in partial redistribution of iron from the liver to the spleen.^399^ Preza et al. used computer modeling and molecular docking to find that the 7-9 N-terminal amino acids of hepcidin, including a single mercaptocysteine, constitute the minimum structure required to retain the activity of hepcidin. Further improvements to enhance resistance to proteolytic enzymes and oral bioavailability of minihepcidins reduce serum iron and liver iron concentrations in mice after parenteral or oral administration, and the effect is comparable to that of natural hepcidin.^400^ These findings suggest that minihepcidins could be used alone to prevent iron overload disease or as an adjunct therapy with iron chelating agents.

Promoting hepcidin synthesis is also a novel therapeutic approach for regulating iron metabolism abnormalities, which can be achieved by inhibiting the expression of transmembrane protease serine 6 (TMPRSS6). TMPRSS6, also known as matriptase-2, negatively regulates the production of hepcidin and is a membrane-bound serine protease expressed in hepatocytes.^401^ In Hfe^−/−^ mice, treatment with antisense oligonucleotides targeting TMPRSS6 significantly decrease serum transferrin saturation, iron levels, and liver iron accumulation.^401^

#### Ferroptosis in thalassemia

Thalassemia is a common monogenic inherited disorder primarily caused by a reduction in globin synthesis due to deletion or point mutations of globin gene clusters. Anemia caused by a defect on chromosome 16, which codes for α-globin, is called α-thalassemia and β-thalassemia is caused by a defect on chromosome 11, which codes for β-globin.^402–404^ Approximately 1–5% of the global population carries the thalassemia gene mutation.^405^ Most patients with severe thalassemia experience complications of iron overload due to blood transfusions, which promote oxidative damage to various organs, leading to endocrine and ultimately organ dysfunction.^406,407^ Chronic iron deposits in the liver can lead to liver fibrosis, cirrhosis, and eventually HCC.^408^ Iron deposition in the thyroid, parathyroid gland, and pancreas causes endocrine gland dysfunction.^409^ Kidney function is also affected by iron overload, putting patients at risk for end-stage renal disease.^410^ The clinical manifestations of β-thalassemia are associated with oxidative stress and ferroptosis caused by iron overload. Clinical trial results showed blood iron overload, elevated lipid peroxidation, and significantly reduced GSH levels in β-thalassemia patients compared to healthy controls.^411^ Additionally, mononuclear cell proteins in the blood of β-thalassemia patients have increased binding activity with oligonucleotide probes targeting Nrf2-associated antioxidant response elements (ARE),^411^ suggesting an adaptive survival response to oxidative stress in these patients.

In addition to commonly used iron chelating therapy, regulating hepcidin levels may also help alleviate iron overload in β-thalassemia patients, due to their typically low hepcidin levels. In β-thalassemia mice, a modest increase in hepcidin expression limited iron overload, reduced the formation of insoluble membrane-bound globin and ROS, and improved anemia. Mice with increased hepcidin expression showed an increase in total hemoglobin levels and a corresponding increase in red blood cell lifespan, suggesting that hepcidin agonists may help treat abnormal iron absorption in patients with β-thalassemia and related diseases.^412^

Minihepcidins, which are hepcidin agonists, improve anemia and iron overload in young β-thalassemia model Hbb^th3/+^ mice, and enhance erythropoiesis without altering the beneficial effect of the iron chelating agent DFO on iron overload in older β-thalassemia mice.^413,414^ These findings suggest that minihepcidins have the potential to be a future treatment for β-thalassemia.

Inducing hepcidin synthesis and secretion, or utilizing hepcidin mimics, can improve β-thalassemia by correcting the imbalance in iron absorption and recycling. However, current hepcidin or minihepcidins alternative strategies require parenteral administration. VIT-2763, an oral small molecule inhibitor of FPN, regulates the internalization of FPN. In Hbb^th3/+^ mouse models, VIT-2763 limits iron availability and improves β-thalassemia and iron homeostasis.^415^ VIT-2763 is under development as an oral medication targeting FPN for β-thalassemia treatment.

#### Ferroptosis in Friedreich’s Ataxia

Friedreich’s Ataxia is a group of chronic, progressive, autosomal recessive neurodegenerative diseases caused by decreased frataxin expression due to the amplification of GAA triplet repeats within the first intron of the frataxin gene.^416–418^

Frataxin activates persulfide transfer, which is essential for the assembly of iron–sulfur clusters (Fe–S) in the mitochondria and the activity of the mitochondrial respiratory chain complex and other mitochondrial enzymes.^419,420^ Mitochondrial iron accumulation, energy imbalance, increased ROS, and lipid peroxidation are all related to the pathogenesis of this disease, suggesting that ferroptosis may be closely related to Friedreich’s Ataxia.^421–424^

Several studies have shown abnormal intracellular iron distribution in Friedreich’s Ataxia, with elevated serum transferrin receptor levels in patients.^425,426^ In the heart tissue of conditional frataxin knockout mice, increased mRNA levels of MFRN2, SEC15L1, and TFR1 and decreased mRNA levels of FPN1 indicate an activated response to cytoplasmic iron deficiency and increased mitochondrial iron input. The mRNA levels of multiple enzymes involved in the biosynthesis pathway using mitochondrial iron (heme and iron–sulfur clusters) are downregulated.^427^ In addition, the mRNA level of HO-1 in the tissues was increased, while the mRNA amount of FtMt was decreased.^427^

FtMt is responsible for transporting and storing iron from the cytoplasm to the mitochondria, thereby reducing the production of cytoplasmic ROS.^428^ Campanella found that frataxin knockout in yeast could maintain mitochondrial DNA integrity and restore mitochondrial respiratory ability, and FtMt overexpression protected cells from oxidative stress caused by H_2_O_2_ treatment.^429^ Subsequent studies found that FtMt overexpression reduced ROS and the contents of unstable iron in cytoplasm and mitochondria. After FtMt overexpression in fibroblasts of patients with Friedreich’s Ataxia, ROS production decreased and Fe-S mitochondrial enzyme activity partially recovered. These data support the idea that increased FtMt may be a possible treatment to counteract frataxin deficiency in patients with Friedreich’s Ataxia. Desmyter et al. found that overexpression of FTL extended the lifespan of Frataxin-deficient yeast by preventing oxidative stress and iron accumulation.^430^ These findings suggest that iron metabolism is abnormal in the cells of Friedreich’s Ataxia models, and regulation of iron metabolism may have some ameliorative effects on Friedreich’s Ataxia.

Patients with Friedreich’s ataxia have impaired antioxidant enzymes and increased sensitivity to oxidative stress.^431^ Primary human fibroblasts from Friedreich’s Ataxia patients are highly sensitive to erastin, a small molecule inhibitor of system Xc^−^, and the ferroptosis inhibitor Fer-1 protects fibroblast cells from damage caused by ferroptosis.^432^ Frataxin deficiency in yeast affects the GSH-dependent redox state of cells, resulting in a fivefold reduction in total glutathione (GSH + GSSG) content in frataxin-deficient yeast models.^433^ The adipocytes of Friedreich’s ataxia mice showed significantly reduced GPX4 expression, increased lipid peroxides, and significantly increased sensitivity to ferroptosis.^434^ Selenium supplementation can effectively improve GPX4 activity and increase the viability of fibroblasts. The small molecule idebenone also has potential to treat Friedreich’s ataxia.^435^ Treatment with ferroptosis inhibitors such as SRS11-92 also inhibit Frataxin-knockdown induced human fibroblast death.^432^ High p53 activity reduces the transcription of SLC7A11 and the antioxidant potential of frataxin-deficient cells, therefore, inhibiting P53 activity to improve system Xc^−^ and GSH content may be a potential therapeutic strategy to combat oxidative stress and possible ferroptosis in Friedreich’s Ataxia. These data confirm the central role of GPX4 in the regulation of intracellular redox states and ferroptosis in Friedreich’s Ataxia. Frataxin-knockout mouse myoblasts, heart, and skin fibroblasts all showed characteristics of ferroptosis, and Nrf2 was downregulated in Friedreich’s Ataxia patients and frataxin-knockout mice, while Nrf2 activators EPI-743 or sulforaphane regulated redox imbalances and rescued ferroptosis in frataxin knockout cells.^436^

Friedreich’s ataxia is characterized by alterations in lipid metabolism, resulting in the accumulation of intracellular lipids in the form of lipid droplets in fibroblasts from patients.^437^ Lipid droplet accumulation was observed in brown adipose tissue and heart in a mouse model of Friedreich’s ataxia, as well as in Frataxin-deficient rat cardiomyocytes and IPSC-derived cardiomyocytes.^434,438^ It is worth noting that Friedreich’s ataxia models are characterized by a significant increase in lipid peroxidation. High plasma MDA concentrations have been observed in patients with Friedreich’s Ataxia.^439^ These data suggest that lipid peroxidation is a major factor in the progression of Friedreich’s Ataxia; however, there are currently no effective treatment strategies. In other diseases, inhibition of ACSL4 to reduce lipid peroxides and ferroptosis is a possible therapeutic strategy, but there is currently no similar study in preclinical studies of Friedreich’s Ataxia.

#### Ferroptosis in Wilson Disease

Wilson disease, also known as hepatolenticular degeneration, is an autosomal recessive disorder characterized by liver cirrhosis and basal ganglia damage due to copper metabolic abnormalities.^440^ ATP7B encodes a transmembrane copper transport ATPase, and mutations in ATP7B cause Wilson’s disease by impairing copper homeostasis, leading to copper overload in the liver, brain, and other organs.^441,442^ Iron and copper are essential nutrients involved in fundamental biological processes that play crucial roles in health and diseases.^443–445^ Copper may positively affect iron transport, and systemic copper deficiency impedes iron transport and accumulation in tissues, ultimately leading to iron deficiency.^446^ Conversely, iron may antagonize copper metabolism, and high doses of iron supplementation can lead to copper depletion.^447^ The liver of patients with Wilson disease contains excess copper and a large amount of iron. Iron is also found in the brain, providing conditions conductive to ferroptosis in Wilson disease.^448–450^ The disturbance of copper metabolism in Wilson’s disease is accompanied by an imbalance of iron homeostasis, and anti-copper treatment improves but does not normalize iron metabolism.^451^

Curcumin, the active ingredient in turmeric, has the potential to inhibit ferroptosis. It improves copper metabolism in Wilson’s disease mice and has a protective effect on Wilson’s disease-related liver injury.^452^ In vitro experiments showed that curcumin had a protective effect on cell damage caused by excessive copper. Curcumin increases the expression of Nrf2, HO-1, and GPX4 in hepatocytes, inhibits the decline of mitochondrial membrane potential in hepatocytes, and all these changes could be reversed by ferroptosis inducer erastin, indicating that curcumin protects against Wilson’s disease by inhibiting ferroptosis.

Ferulic acid is a traditional Chinese medicine monomer effective in the clinical treatment of cognitive disorders-related diseases.^453,454^ Ferulic acid can improve the survival rate of copper-overloaded cells, significantly increase the expressions of SIRT1, Nrf2, SLC7A11, and GPX4, and downregulate the levels of MDA, 4-HNE and ROS.^455^ In addition, ferulic acid improves cognition dysfunction in copper-loaded Wilson’s disease rats, suggesting that it reduces hippocampal neuron damage by activating SIRT1-mediated ferroptosis, providing a valuable drug candidate for the clinical treatment of cognitive impairment caused by Wilson’s disease.^455^

#### Ferroptosis in Duchenne muscular dystrophy

Duchenne muscular dystrophy (DMD) is an X-linked recessive disorder caused by mutations in dystrophin protein encoded by the DMD gene.^456^ Muscle cells lacking dystrophin are more sensitive to injury, resulting in abnormal ROS production, which is correlated with the severity of DMD and is a common therapeutic target for muscular dystrophy. The accumulation of ROS leading to lipid peroxidation is considered the principal mechanism underlying sarcolysis in muscular dystrophy.^457^ Iron, an important modulator of oxidative stress, also contributes to the dystrophic pathology.^457^ The production of iron-dependent hydroxyl radicals has been associated with muscle necrosis in a mouse model of DMD, and iron deprivation can decrease muscle necrosis and has potential therapeutic benefits.^458^ Interestingly, iron levels are significantly increased in the gastrocnemius and tibialis anterior muscles in dystrophin-utrophin knockout mice, and treating these mice with the iron chelator DFO reduces both superoxide levels and the dystrophic pathology. Additionally, although dietary iron overload did not increase the dystrophic pathology, total muscle iron content and ferritin expression were increased.^457^ No studies to date have reported that ferroptosis is involved in the pathological progression of DMD. However, the above-mentioned results suggest that altered iron metabolism is closely related to DMD and may serve as a viable new target for clinical treatment. Further studies are needed to determine the roles of altered iron homeostasis and ferroptosis in DMD.

### Ferroptosis in cardiovascular diseases

**Fig. 4:**
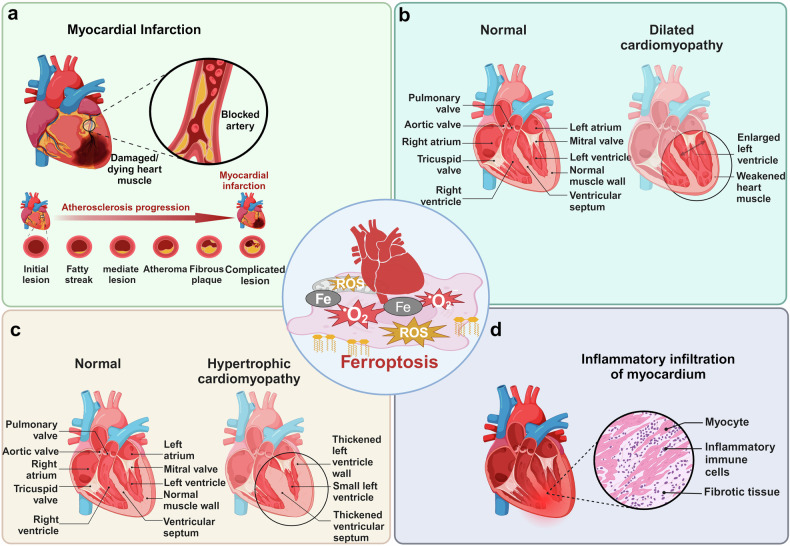
Ferroptosis in cardiovascular diseases. In the circulatory system, iron overload, lipid peroxidation and oxidative stress associated with ferroptosis can gradually induce coronary atherosclerotic heart disease and myocardial infarction (**a**), dilated cardiomyopathy (**b**), hypertrophic cardiomyopathy (**c**), and inflammatory infiltration of myocardium (**d**). This occurs through the damage of vascular endothelial cells and myocardial cells, leading to inflammatory responses. This figure was created with BioRender (https://biorender.com/)

Iron, as an important trace element in the body, can influence the incidence of cardiovascular events when deficient or overloaded.^459–461^ Clinical studies show that 47% of elderly heart failure patients have reduced transferrin saturation, and low transferrin saturation is independently linked to a higher risk of all-cause death.^462^ Increasing evidence suggests that the development of many forms of cardiovascular diseases, such as heart failure, myocardial infarction, and cardiomyopathy, is driven by ferroptosis (Fig. 4). Summarizing the role of ferroptosis in cardiovascular diseases could pave the way for innovative clinical treatments targeting ferroptosis.^260,463^

#### Ferroptosis in heart failure

Heart failure ranks among the most prevalent chronic conditions affecting the elderly population, characterized by cardiac hypertrophy and cardiac fibrosis.^464^ Systemic and pulmonary hypertension are the main clinical causes of heart enlargement, cardiac fibrosis, and eventually heart failure. Because cardiomyocytes are terminal differentiated cells, their loss in heart failure is irreversible, therefore, preventing cardiomyocyte hypertrophy and death is of great physiological significance for maintaining heart function and delaying heart failure. Clinical studies indicate that cardiomyocytes are particularly vulnerable to free iron overload, and excessive heme iron intake has been linked to an elevated risk of cardiovascular diseases.^465^ Both iron overload or deficiency can precipitate heart failure by disrupting iron homeostasis within cardiomyocytes.^466^ GO and KEGG analyses based on the GSE180065 and GSE36074 datasets showed that ferroptosis-related pathways are enriched in heart failure. Heart failure with preserved ejection fraction (HFpEF) mice exhibit significant abnormal activation of ferroptosis, and inhibition of ferroptosis could improve the HFpEF phenotype.^467,468^

The release of free iron from ferritin storage occurs via NCOA4 mediated ferritinophagy. Specifically, knocking out NCOA4 in cardiomyocytes alleviates heart failure caused by transection aortic contraction (TAC) by inhibiting ferritinophagy, free iron overload, and increased lipid peroxidation.^469^ The mitochondrial protein sideroflexin 1 (SFXN1) is responsible for importing iron from the cytoplasm to the mitochondria, and SFXN1 deficiency leads to mitochondrial respiratory chain damage, affecting the biogenesis and assembly of complex III.^470^ In animal models of Apelin-13-caused cardiomyocyte hypertrophy and heart failure, NCOA4-activated ferritinophagy leads to the upregulation of SFXN1 expression, causing mitochondrial iron overload and mitochondrial ROS overproduction, increased lipid peroxidation, and ultimately, ferroptosis.^471^

Knocking down FTH in cardiomyocytes prevents free iron from being stored in ferritin, increases unstable iron pools, promotes intracellular ROS production, activates oxidative stress, and causes ferroptosis in cardiomyocytes, leading to age-related heart damage.^472^ A high-iron diet significantly reduces SLC7A11 expression and GSH levels, and exacerbating cardiac injury and myocardial hypertrophy in cardiomyocyte-specific FTH knockout mice.^67^ Additionally, the Toll-like receptor 4 (TLR4)/NADPH oxidase 4 (NOX4) pathway plays a role in heart failure associated with ferroptosis. In mice with myocardial hypertrophy induced by angiotensin II (ANGII), the expression of SLC7A11 decreased, and the inhibitory effect of SLC7A11 increased the contents of PTGS2, MDA and ROS, which aggravated cardiomyocyte hypertrophy.^473^ Mixed lineage kinase 3, a member of the MAP3K family, regulates the JNK/p53 pathway to mediate SLC7A11 expression and oxidative stress, causing myocardial fibrosis in the late stage of chronic heart failure in the TAC mouse model.^474^ Inhibition of TLR4/NOX4 significantly improves left ventricular remodeling by inhibiting ferritinophagy, confirming the critical role of ferritin-mediated ferroptosis in the development of cardiac hypertrophy and heart failure.^475^ Puerarin alleviates heart failure by inhibiting ferroptosis through the endogenous TLR4/NOX4 pathway.^476^ The sodium-glucose cotransporter 2 inhibitor canagliflozin may also exert its cardiovascular benefits in part by reducing ferroptosis and improving the HFpEF phenotype.^477,478^

Myocardial mitochondria are central to myocardial energy metabolism. Mitochondrial dysfunction leads to the decrease of NAD^+^ level, resulting in lysosomal acidification and impaired autophagy, and induces ferroptosis, involving the accumulation of iron in lysosomes and lipid peroxidation.^479^ Nicotinamide mononucleotide increases NAD^+^ levels, improves lysosomes damaged by mitochondrial dysfunction and autophagy, thereby inhibiting ferroptosis and reducing heart failure.^479^

Oxidative stress and ROS signaling associated with iron overload may mediate important factors in cardiac hypertrophy and heart failure.^480^ However, the precise mechanisms through which these molecules and pathways contribute to ferroptosis, as well as the ways in which ferroptosis exacerbates hypertrophic growth of cardiomyocytes, remain poorly understood and warrant further investigation.

The accumulation of toxic lipid peroxides in myocardium also affects cardiac function. Through lipidomics and RNA sequencing, Bi et al. observed a marked increase in ACSL4 expression in a heart failure model induced by TAC in mice, and ACSL4 overexpression in cardiomyocytes exacerbates the cardiac dysfunction triggered by pressure overload through ferroptosis.^481^ Notably, ACSL4-driven ferroptosis has been found to activate the pyroptosis pathway, leading to elevated levels of IL-1β.^481^ This suggests that ACSL4 initiates a cascade that links ferroptosis to pyroptosis, thereby promoting cardiac hypertrophy and responding to the hemodynamic stress induced by aortic constriction. These findings elucidate the role of the ACSL4–ferroptosis–pyroptosis axis in pressure-overload-induced heart failure and offer potential therapeutic targets for its prevention.

#### Ferroptosis in doxorubicin induced cardiomyopathy

Doxorubicin (DOX) is extensively utilized in chemotherapy; however, its application is significantly constrained by life-threatening cardiotoxic effects and the potential for developing cardiomyopathy.^482^ DOX can directly bind Topoisomerase II beta in cardiomyocytes, causing DNA double-strand breaks.^483^ DOX-induced cardiomyopathy results in myocardial autophagy dysfunction, accumulation of iron in both cell and mitochondria, and elevated levels of ROS, 4-HNE, and MDA.^484^ Patients suffering from DOX-induced cardiomyopathy exhibit significantly higher cardiac mitochondrial iron levels compared to those with normal cardiac function or other types of cardiomyopathies.^485^ DOX can chelate Fe^3+^ to form a complex with Fe^2+^, which triggers excessive lipid peroxidation within mitochondria, resulting in mitochondria-dependent ferroptosis. In cultured cardiomyocytes, DOX concentrates in mitochondria, increasing mitochondrial iron deposition and ROS levels, and dexrazoxane has demonstrated efficacy in reversing DOX-induced cardiomyopathy by lowering mitochondrial iron levels.^485^ Additionally, overexpressing ABCB8, a mitochondrial protein that promotes iron export, reduces both mitochondrial ROS and iron, improving DOX-induced cardiomyopathy.^485^ These findings indicate that mitochondria iron overload, combined with ROS formation and subsequent ferroptosis, intensifies the cardiotoxic effects of DOX-induced cardiomyopathy and may be an important mechanism. Recent research has shown that DOX-induced ferroptosis in cardiomyocytes is reliant on GPX4, specifically GPX4 in mitochondria. The mitochondrial DOX-Fe^2+^ complex induces triggers lipid peroxidation, resulting in mitochondria-dependent ferroptosis and decreased cardiac function in mice, which is reflected in significantly reduced left ventricular ejection fraction and myocardial fibrosis.^486^ Overexpression of GPX4 in cardiomyocytes or targeting the mitochondrial DOX-Fe^2+^ complex prevents DOX-induced ferroptosis and improves cardiomyocyte injury.^486^

Nrf2 can activate the transcription of a series of antioxidant genes and block ferroptosis, playing a protective role in DOX-induced cardiomyopathy by regulating oxidative stress and autophagy.^487–489^ However, these findings appear to contradict Fang’s results.^490^ Fang found DOX increased Nrf2 nuclear accumulation, promoted the expression of HO-1, and caused heme degradation, and led to the rapid and systematic accumulation of free non-heme iron. Knocking out Nrf2 or using HO-1 antagonist reversed DOX-induced heart injury and death in mice, indicating that free iron released by heme degradation is a prerequisite for inducing heart injury.^490^ Ferroptosis inhibitors, such as Fer-1 or dexrazoxane, significantly alleviate myocardial damage caused by DOX and reduce the mortality in mice by inhibiting ferroptosis.^490^ Excessive inhibition of Nrf2 may not improve DOX-induced cardiomyopathy due to insufficient promotion of antioxidant gene expression, while its overactivation increases the breakdown of heme and ferrous iron levels in the HO-1-catalyzed manner, promoting ferroptosis. Therefore, both excessive activation and inhibition of Nrf2 may be detrimental to DOX-induced cardiomyopathy.

Together, these finding indicate that the toxic effects of DOX on the heart are strongly associated with iron overload and ferroptosis in mitochondria, indicating that targeting ferroptosis could serve as a promising therapeutic strategy for protecting the hearts of cancer patients from DOX-induced cardiomyopathy.

#### Ferroptosis in metabolic cardiomyopathy

Lipotoxic damage from myocardial triglycerides accumulation and insulin resistance due to hyperglycemia are considered as metabolic cardiomyopathy, and are the primary causes of heart failure in obese patients. A high-sucrose, high-fat diet causes harmful oxidative modification in metabolically active proteins, resulting in maladaptive tissue remodeling and potentially exacerbating diastolic left ventricular dysfunction.^491^ Inhibiting ferroptosis has a protective effect on obese and diabetic cardiomyopathy.^492^

Epidemiological studies have found that human GPX4 gene variants are associated with obesity and cardiovascular diseases. PA induces ferroptosis in H9c2 cardiomyoblasts in a time- and dose-dependent manner. Mechanistically, PA reduces the expression of GPX4 and heat shock factor 1 and regulates the transcription of genes related to iron metabolism (such as FTH, TFR1, and SLC40A1), causing iron homeostasis imbalance, while GPX4 overexpression reverses PA-induced ferroptosis in cardiomyocytes.^493^ Lipid peroxides and their reactive aldehyde derivatives are the key factors in cardiometabolic disorders in obesity, with GPX4 playing a crucial role. Oral carnosine therapy significantly increases GPX4 expression in the hearts of mice on a high-sucrose, high-fat diet and decreases levels of protein carbonyl and iron to improve myocardial fibrosis.^494^ Compared with peanut oil and lard oil, functional blended oil (BO), which contains a high concentration of oleic acid and a low concentration of α-linolenic acid, significantly increases high-density lipoprotein cholesterol levels, lowers serum triglyceride and LDL cholesterol levels in mice, and reduces the expression of genes associated with lipid anabolism and inflammation.^495^ In addition, BO decreases ROS and MDA content and the atherosclerosis index in tissues, while increasing antioxidant enzyme activity, including SOD, GPX and catalase,^495^ suggesting that mixed oils with a low *n* − 6/*n* − 3 PUFA ratio could be effective in preventing and managing cardiovascular diseases.

The incidence and mortality rates of cardiovascular diseases are significantly higher in individuals with diabetes compared to non-diabetic individuals.^496^ Diabetic patients tend to have worse outcomes after acute myocardial infarction, and hyperglycemia increases the heart’s susceptibility to I/R injury, which in turn induces multiple forms of programmed cell death, including ferroptosis.^497,498^ In a streptozotocin-induced mouse model of type 1 diabetes, hyperglycemia-induced ER stress and ferroptosis are key factors contributing to myocardial I/R damage.^248^ Cardiac retinol metabolism disorders in patients and mice with type 2 diabetes are marked by excessive retinol and a deficiency in all-trans retinoic acid. The decreased expression of retinol dehydrogenase 10 has been proved to be the primary cause of these metabolic disturbances, leading to diabetic cardiomyopathy induced by lipid toxicity and ferroptosis.^499^

GPX4 is significantly decreased in the hearts of patients with diabetes and hyperglycemia, indicating that GPX4 may play an antioxidant role in diabetic cardiomyopathy.^500^ Overexpression of GPX4 in mitochondria protects streptozotocin-induced heart damage.^501^ Activation of Nrf2 prevents ferroptosis by upregulating ferritin, SLC7A11 and GPX4.^502^ In mice with type 2 diabetes, sulforaphane inhibits ferroptosis by inducing Nrf2-mediated metallothionein expression, thereby preventing diabetes-related cardiac inflammation, oxidative damage, and hypertrophy.^503^ Curcumin^504^ and dexmedetomidine^505^ also improve diabetic cardiomyopathy by activating the Nrf2 pathway to inhibit ferroptosis.

In a diabetic cardiomyopathy model, ferroptosis interacts with inflammation. Ubiquitin-specific protease 24 (USP24) promotes ferroptosis by activating NF-κB pathway, upregulating ACSL4 levels, and reducing SLC7A11 and FTH1 levels, thereby decreasing the antioxidant capacity of cardiomyocytes.^506^ Astragaloside IV has antioxidant, anti-inflammatory, and other pharmacological effects. Astragaloside IV ameliorates myocardial injury in diabetic rats by suppressing CD36-mediated ferroptosis and reducing lipid deposition.^507^ Irisin is a myokine secreted by skeletal muscle. Streptozotocin induces lower expression levels of irisin in serum and heart of mice with type 1 diabetes. Intraperitoneal injection of irisin improves the increase of MDA and the decrease of GSH, SLC7A11 and GPX4 in myocardium, thereby inhibiting ferroptosis, improving cardiac function impairment in diabetic cardiomyopathy mice, and protecting cardiomyocytes from high glucose-induced damage.^508^ Nicorandil, an ATP-sensitive K^+^ channel opener, alleviates cardiac microvascular injury by improving microvascular perfusion and structural integrity.^509^ The mechanism may involve nicorandil promoting Pink1/Parkin-dependent mitochondrial autophagy, inhibiting mitochondrial translocation of ACSL4 and inhibiting ferroptosis.^509^ The combination of suberosin and thiazolidinedione reduces serum iron concentration, decreases MDA levels, and downregulates the expression of ACSL4, LPCAT3 and LOX in the heart tissue of diabetic rats. The combination improves GPX4 activity by activating the AKT/PI3K/GSK3β pathway, effectively ameliorating heart injury.^510^

Targeting ferroptosis in cardiomyocytes presents a promising therapeutic pathway for preventing and/or treating diabetic cardiomyopathy. Although various candidate interventions and antioxidants have demonstrated protective effects against ROS production and lipid peroxidation in diabetic hearts, further clinical trials are necessary to establish the definitive connection between ferroptosis and diabetic cardiomyopathy.

#### Ferroptosis in heart transplantation

For patients suffering from severe coronary artery disease and advanced congestive heart failure, heart transplantation stands as the most effective treatment option.^511^ However, I/R injury caused by the restoration of coronary blood flow after transplantation can lead to inflammation, transplant dysfunction, and even death of the patient.

To maintain the function of the donor heart during transplantation, it is usually necessary to store it at a low temperature. A recent study found that cold heart preservation causes damage in elderly donor hearts. Results of RNA-Seq showed that the expression of cold-inducible RNA-binding protein (Cirbp) decreased in the elderly donor heart due to low temperature, leading to severe ferroptosis in recipient hearts after transplantation.^512^ Overexpression of Cirbp in the elderly donor heart and supplementation of cardiac arrest fluid with Cirbp agonists reversed the effects of hypothermia.^512^

In a myocardial I/R injury model induced by coronary artery ligation, inhibiting ferroptosis led to a reduction in infarct size and enhancement of left ventricular systolic function. Mechanistically, the inflammatory response after heart transplantation is mediated by TLR4/TRIF signaling pathway, which facilitates neutrophil migration to coronary endothelial cells, resulting in ferroptosis in the transplant endothelial cells.^513^ Fer-1 reduces the levels of hydroperoxy-arachidonoyl-phosphatidylethanolamine, inhibits neutrophil adhesion to coronary endothelial cells, reduces ferroptosis in cardiomyocytes, and hinders neutrophil recruitment post-heart transplantation.^513^ Therefore, inhibiting ferroptosis before heart transplantation may reduce the inflammatory response in heart injury and enhance the prognosis of heart transplant recipients.

#### Ferroptosis in aortic dissection

Vascular disease is a complex subtype of cardiovascular disease, attributed to multiple factors such as genetic variation, environmental influences, and lifestyle habits. Aortic dissection, also known as an aortic dissection aneurysm, is a serious cardiovascular emergency. Aortic dissection occurs when there is a break in the lining of the artery wall, allowing blood to enter and form a hematoma, further stripping the intima and media of the aorta.^514,515^ Aortic dissection progresses rapidly and has high early mortality. The loss of smooth muscle cells is the main pathological feature of aortic dissection. In patients with Stanford type A aortic dissection, iron levels and the lipid peroxidation product 4-HNE were increased in the aorta, while key ferroptosis regulatory proteins, including SLC7A11, GPX4, and FSP1 were downregulated. The expression of RNA m6A methyltransferase-like 3 (METTL3) was significantly upregulated, with METTL3 protein levels in the aorta showing a negative correlation with the levels of FSP1 and SLC7A11.^516,517^ Knockdown of METTL3 promotes the expression of FSP1 and SLC7A11, whereas METTL3 overexpression aggravates cystine deprivation- and erastin-induced ferroptosis in human aortic smooth muscle cells (HASMCs), suggesting that METTL3 may promote ferroptosis in HASMCs primarily by inhibiting FSP1 and SLC7A11 expression.^516^ The histone methyltransferase inhibitor BRD4770 also inhibits cystine deprivation- or ferroptosis inducer-induced smooth muscle cell ferroptosis, diminishing morbidity and mortality from aortic dilation.^517,518^

lncRNA NORAD is downregulated in the aorta of patients with aortic dissection, and overexpression of NORAD promotes the growth of vascular smooth muscle cells (VSMCs) and inhibits ferroptosis induced by AngII.^519^ The mechanism may be related to the interaction between NORAD and HUR, which promotes the stability of GPX4 mRNA and increase GPX4 levels. Additionally, METTL3 promoted the m6A methylation of NORAD and participated in the regulation of ferroptosis in VSMCs.^519^

It is worth noting that the above studies are only the beginning of the research on the relationship between ferroptosis and aortic dissection. Many key questions remain unsolved, such as the role of inflammation in the development of aortic dissection, how ferroptosis and inflammation work synergistically to promote aortic dissection, and the ‘cause or effect’ relationship between ferroptosis and aortic dissection. Therefore, it is urgent to study the molecular mechanisms of ferroptosis in the progression of aortic dissection and to explore treatment strategies for delaying its progression.

#### Ferroptosis in atherosclerosis

Atherosclerosis is a long-term inflammatory condition marked by the development of lipid-rich plaques within large and medium-sized arteries, leading to diminished blood flow to tissues.^520^ As a class of protective immune cells, macrophages have diverse functions in atherosclerosis. The number and phenotype of macrophages in atherosclerotic plaques are intimately linked to the disease’s onset and progression. In the early stages of atherosclerosis, macrophages bind to lipopolysaccharides to promote cholesterol accumulation, lipid accumulation in plaques, and foam cell formation, which is a hallmark of atherosclerotic lesions.^521–523^ In addition, macrophages exacerbate the inflammatory response by promoting the secretion of ROS, cytokines, and chemokines.^524^ In the advanced stages of atherosclerotic disease, macrophages destroy the collagen of the fibrous cap and induce smooth muscle cell death by secreting multiple types of inflammatory factors and matrix metalloproteinases (MMPs), thus exacerbating plaque instability.^525,526^

Moderate amounts of iron can influence the differentiation and function of macrophages, contributing to the production of ROS to eliminate foreign microorganisms, with important physiological implications for macrophages.^527^ However, excess iron in macrophages can have adverse effects, such as exacerbating the process of atherosclerosis. For example, iron overload promotes the inflammatory response in macrophages by increasing the ability of 5-LOX to bind to the nuclear membrane. High iron content in macrophages affects antioxidant capacity and cytokine release. Macrophages with higher iron content also secrete more MMPs, leading to the rupture of atherosclerotic plaques.^528^ Excess irons can promote the progression of atherosclerosis by affecting macrophages, and a balanced intracellular iron environment is crucial in alleviating atherosclerosis.

Malhotra et al. demonstrated that hepcidin slowed the progression of atherosclerosis by reducing iron levels in macrophages.^529^ The expression levels of hephaestin and ceruloplasmin (CP) in atherosclerotic plaques were significantly lower than those in normal tissues. Consequently, intracellular Fe^2+^ could not be oxidized to Fe^3+^ and excreted from macrophages via FPN, which may be the potential mechanism of iron retention in plaques.^530^ Heat shock protein 27 reduces iron absorption by downregulating TFR1, thus preventing ferroptosis and ameliorating coronary artery disease.^531^ Fer-1 inhibits iron accumulation, lipid peroxidation, thereby alleviating atherosclerotic damage in Apolipoprotein E^-/-^ (ApoE^-/-^) mice fed a high-fat diet.^532^ Iron chelating agents and dietary iron restriction stabilize atherosclerotic plaques and prevent endothelial damage.^532^

ROS and oxidative stress significantly promote the progression of atherosclerotic plaques.^533^ Clinical studies have shown that GPX4 expression in human coronary artery specimens is inversely related to the severity of atherosclerosis.^534^ Downregulation of GPX4 may result in the accumulation of lipid peroxides and the induction of ferroptosis, thereby promoting atherosclerosis.^535^ p53 can accelerate ferroptosis and the development of atherosclerosis by binding to SLC7A11, preventing the synthesis of GSH, and increasing the hydrolysis of GSH and the production of ROS by activating glutaminase 2.^66^ Nrf2 regulates the expression of ATP-binding cassette transporter B6, inhibits ferroptosis, and slows the development of atherosclerosis.^536^ Prenyldiphosphate synthase subunit 2 (PDSS2) may inhibit the formation of atherosclerotic plaque by activating Nrf2 and reducing ferroptosis in human coronary artery endothelial cells.^537^

Lipid peroxidation associated with ferroptosis accelerates the development of atherosclerosis. C1q/TNF-related protein 5 upregulates 12/15-LOX through STAT6 signaling to accelerate the development of atherosclerosis, while inhibition of 12/15-LOX reduces LDL oxidation and improves atherosclerosis.^538^ In addition to LOX, PTGS2 and ACSL4 also play important roles in phospholipid metabolism. The expression of PTGS2 and ACSL4 in human coronary artery specimens is positively correlated with the severity of atherosclerosis.^534^

Elevated hematocrit levels are linked to increased cardiovascular risk, including atherosclerosis. JAK2V617F (Jak2VF) mutations increase the risk of cardiovascular disease. Mice that overexpress Jak2VF in their erythroid lineage (VFEpoR mice) exhibit heightened necrosis in atherosclerotic plaque and erythrocytic ferroptosis, and VFEpoR increases erythrocyte lipid hydroperoxide and decreases antioxidant defense ability.^539^ Low dose of erythropoietin can selectively induce erythrocytosis, accompanied by significant ferroptosis and lipid peroxidation, which further aggravate atherosclerosis. The ferroptosis inhibitor liproxstatin-1 has been shown to reverse iron accumulation, endothelial damage, and increased atherosclerosis in Jak2VF chimeric mice and VFEpoR mice,^539^ indicating a potential therapeutic target for mitigating erythrocytic ferroptosis-mediated cardiovascular risk.

Atherosclerosis is the basis of many cardiovascular diseases. The pathogenesis of atherosclerosis is complex, involving vascular endothelial cells, blood cells and macrophages. The current study of ferroptosis in atherosclerosis has received considerable attention, but it is still at a very early stage, with current research focusing mainly on macrophages. Clarifying the specific role of ferroptosis in the onset of atherosclerosis, as well as its role in other organ lesions, still requires attention and participation of more researchers.

### Ferroptosis in central nervous system diseases

**Fig. 5:**
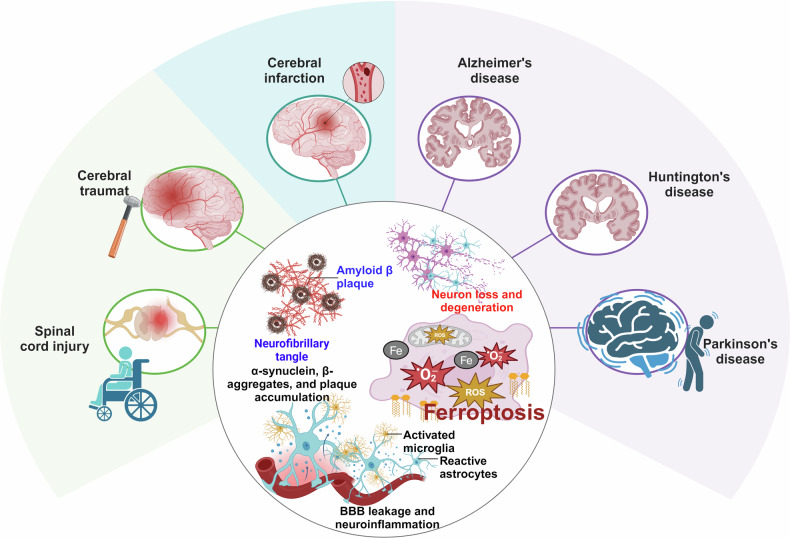
Ferroptosis in neurodegenerative diseases. In the nervous system, ferroptosis primarily induces and promotes neuronal damage and axonal degeneration. It also facilitates the aggregation of amyloid-beta (Aβ) in the brain and contributes to the formation of neurofibrillary tangles. Additionally, ferroptosis activates glial cells, triggering an inflammatory response. These complex pathological changes in the brain interact with each other, leading to neurodegenerative diseases such as Alzheimer’s disease (AD), Parkinson’s disease (PD), and Huntington’s disease (HD), as well as ischemia-reperfusion injury and inflammatory responses. This figure was created with BioRender (https://biorender.com/)

The absorption of iron in the central nervous system, particularly in the brain, is precisely regulated.^540^ Iron homeostasis in the central nervous system is influenced not only by systemic iron metabolism, but also by function of the blood-brain barrier (BBB).^541^ The BBB controls the entry of circulating substances and cells into the brain, maintaining the stability of normal brain function and serving as the primary barrier for iron entry. Under physiological conditions, iron content in the central nervous system is dynamically balanced among uptake, storage, and output to maintain normal neuronal metabolism and function. Neurological diseases can disrupt iron absorption and transport, leading to excessive iron accumulation, increased oxidative stress, and resulting in cellular ferroptosis. Growing research indicates that ferroptosis is a significant contributor to neurodegenerative diseases. As a result, pharmacological approaches to inhibit ferroptosis are being recommended as potential treatments for these diseases (Fig. 5).

#### Ferroptosis in Alzheimer’s disease

AD is the most common clinical senile degenerative cognitive disorder.^542^ Although tau protein aggregation and beta-amyloid protein (Aβ) accumulation play central roles in the pathophysiological hypothesis of AD, the potential role of metal overload, subsequent oxidative stress, and tissue damage cannot be ignored.^543–545^ Increased levels of iron in the brain are an important feature of AD patients, and have been reported since as early as the 1950s. These elevated iron levels are related in the progression of AD and cognitive decline.^546^ Evidence from a large autopsy cohort suggests that iron levels in the brain are associated with the probability of neurodegeneration in AD.^547^ A comprehensive and multidisciplinary analysis of post-mortem brain tissue in AD patients, including protein immunoblotting, elemental analysis, and metabolomics, showed that iron imbalance and elevated ferritin levels were associated with lipid peroxidation in AD patients.^548^ Studies have found that the disturbance of iron homeostasis is related to the pathogenesis of AD, and excess iron will aggravate oxidative damage and cognitive deficits.^549–553^ Single-cell RNA sequencing revealed a significant reduction in astrocytes in AD mice, with ferroptosis activated in astrocytes, and FTH1 in astrocytes was identified as a hub gene associated with ferroptosis.^554^ ApoE activates the PI3K/AKT pathway and then inhibits autophagy degradation of ferritin, thereby avoiding iron-dependent lipid peroxidation. ApoE abundance in the cerebrospinal fluid was reduced in patients with AD, which was associated with cognitive decline.^555^ Allelic variations of the APOE gene may be the greatest genetic risk for AD.

Aβ aggregation leads to significant changes in the transcriptome, proteome, and metabolome of neurons. Results of gene set enrichment analysis suggested that ferroptosis may be an important pathway of Aβ-induced cytotoxicity. Aβ significantly decreases GSH levels in MC65 nerve cells, increases nonenzymatic and enzymatic oxidation of arachidonic acid (COX, 5-LOX, 12-LOX, and 15-LOX), and increases mitochondrial ROS levels and total intracellular lipid peroxides.^556^ The results of animal experiments show that the expression of GPX4 and SLC7A11 in entorhinal cortex is decreased by injection of Aβ, and the level of TFR is increased, which could be inhibited by the application of Fer-1.^557^

GPX4 is recognized as the principal regulator of ferroptosis since it can convert PLOOHs in the membrane into nontoxic alcohols.^558^ Mice with GPX4 conditional knockout in forebrain neurons (GPX4BIKO mice) exhibited notable impairments in spatial learning and memory.^559^ Further analysis revealed that cognitively impaired GPX4BIKO mice experienced neurodegeneration and increased lipid peroxidation in the hippocampus.^559^ Administering small molecule ferroptosis inhibitors improves neurodegeneration in GPX4BIKO mice, indicating ferroptosis may play a crucial role in the pathology of AD.^559^ Presenilin 1 and 2 (PS1 and PS2) control GPX4 expression by regulating intracellular selenium supply through the Notch/LRP8 pathway. Thus, presenilin deficiency makes multiple cell types significantly sensitive to ferroptosis. Presenilin mutations disrupt selenium uptake and thus inhibit GPX4 expression, increasing the sensitivity of multiple cell types to ferroptosis.^560^ Presenilin mutations in patients with AD may contribute to neurodegeneration by downregulating GPX4 and promoting ferroptosis. NOX4 is the primary producer of ROS in cells. In astrocytes within the cerebral cortex of AD patients and APP/PS1 double-transgenic AD mice, NOX4 levels of were found to be significantly increased, as were levels of MDA and 4-HNE. Overexpression of NOX4 inhibits mitochondrial respiration by reducing mitochondrial protein complexes, promoting mitochondrial ROS production, and thus inducing ferroptosis in astrocytes.^561^ This suggests that NOX4 promotes ferroptosis through oxidation stress and may represent a critical molecular mechanism underlying astrocyte injury in AD.

Nrf2 is responsible for regulating the transcription of many key components within the ferroptosis pathway,^562^ and targeting Nrf2 has emerged as a novel strategy for preventing and treating AD.^563^ The expression of Nrf2 was found to be reduced in the frontal cortex of AD patients and AD model mice, while the expression of NOX4 was upregulated.^564^ HO-1 and GPX4 were downregulated due to Nrf2 defects in astrocytes, leading to increased mitochondrial fragmentation, DNA oxidation, and lipid peroxidation in mouse astrocytes,^564^ suggesting that Nrf2 deficiency promotes ferroptosis in astrocytes in AD mice.

The progression of AD is not limited to the ferroptosis of neurons and astrocytes. Pericytes are an important part of the neurovascular unit and BBB. In APP/PS1 mice, BBB permeability and pericyte number are reduced, and β-amyloid induces mitochondrial autophagy-dependent ferroptosis via the CD36/PINK/PARKIN pathway, resulting in blood-brain barrier damage and contributing to AD.^565^ The disturbance of iron homeostasis in the brain and ferroptosis of neurons can exacerbate neuroinflammation, causing abnormal microglial activation. These microglia release various pro-inflammatory factors, which further disrupt iron balance, creating a detrimental feedback loop.^566^ This is also an important mechanism of AD, leading to neurological dysfunction and disease progression.

Based on the important role of ferroptosis in AD pathogenesis, inhibiting ferroptosis has become a significant strategy to reduce neuronal damage in AD in preclinical studies.^567,568^ For example, tannic acid could bind to the GPX4 activation site, enhancing its activity and cellular level, which in turn regulated amyloid and tau protein deposition by reducing oxidative stress and inhibiting ferroptosis,^569^ providing a promising therapeutic avenue for combating the interaction between ferroptosis and AD. Paeoniflorin could reduce neuronal ferroptosis and improve the cognitive ability of AD mice by reducing the levels of iron, ROS and MDA in brain tissue and increasing the expression of SOD.^570^ Eriodictyol markedly improves cognitive deficits in APP/PS1 mice and inhibits tau phosphorylation and Aβ aggregation in the brain of APP/PS1 mice, which may be related to the activation of the Nrf2/HO-1 signaling pathway to inhibit ferroptosis.^571^ Forsythoside A inhibits ferroptosis through Nrf2/GPX4 axis activation, thereby alleviating Alzheimer’s-like pathology.^572^ Hydroxylated chalcones can inhibit Aβ aggregation and Aβ-induced decrease in GPX4 and increases in lipid peroxidation and may be potential candidates for the treatment of AD.^573^ Curculigoside significantly improves cognitive impairment in scopolamine- and okadaic acid-induced AD model mice. The mechanism may be related to curculigoside significantly reducing AD-promoting factors (e.g., Aβ1-42, p-tau) and increasing ferroptosis protective factors (e.g., GPX4, SLC7A11, GSH) in the hippocampus and cortex of AD mice,^574^ suggesting that the natural compound curculigoside can be used as a promising therapeutic agent to improve AD by inhibiting ferroptosis.

Salidroside exerts neuroprotective properties by inhibiting Aβ-induced ferroptosis in AD mouse neurons, and its mechanism is related to Nrf2 activation and the promotion of downstream NQO1, HO-1, and GPX4 protein expression.^575^ In SAMP8 mice, salidroside upregulates SLC7A11 and GPX4 protein expression through activating Nrf2/GPX4 axis, reduces iron deposition and ACSL4 protein expression, thereby inhibiting ferroptosis and Aβ accumulation, and alleviating cognitive impairment.^576^ Tetrahydroxy stilbene glycoside (TSG) enhances the activation of Keap1/Nrf2/ARE and GSH/GPX4 signaling pathways, reduces ferroptosis by regulating the levels of ferroptosis-related proteins and enzymes, and thus inhibits the production and deposition of Aβ in the brain of APP/PS1 mice,^577^ suggesting that TSG may be a promising therapeutic agent for AD by targeting ferroptosis. Spermidine can reduce the expression of TFR1 and ALOX15, and upregulate Nrf2 and SLC7A11 expression. In combination with ciprofloxacin, it can enhance the antioxidant effect of ciprofloxacin and reduce its toxic effect, which may provide potential benefits against AD by regulating ferroptosis.^578^ A polycatechol composed of dopamine and L-Dopa can isolate the unstable iron pool, scavenge free radicals, protect mitochondria, and prevent ferroptosis, while inhibiting Aβ and tau protein aggregation to save neuronal cell death. Interestingly, polycatechol promotes liquid-liquid phase separation of tau and regulates its intermolecular interactions to inhibit the formation of toxic tau aggregates, providing a conceptually innovative approach for treating AD.^579^

In addition, metabolic disorders of neuronal Ca^2+^ can drive ferroptosis through interactions with iron and crosstalk between the ER and mitochondria, and maintaining Ca^2+^ homeostasis to inhibit ferroptosis may be an innovative target for the treatment of AD.^580^ Alpha-lipoic acid exerts its neuroprotective effects by inhibiting calpain1 activity of and significantly reducing Ca^2+^ levels in mouse brain tissue. It also blocks tau-induced iron overload and lipid peroxidation, thereby inhibiting tau hyperphosphorylation and ferroptosis of neurons in AD mice through multiple pathways.^581^ Tenuifolin prevents AD-like phenotypes by maintaining the stability of the calpain system, inhibiting oxidative stress and ferroptosis.^582^

In short, the treatment of AD is still an important clinical problem, targeting the key pathway of ferroptosis for treating AD may be a breakthrough.

#### Ferroptosis in Parkinson’s disease

PD is a progressive neurodegenerative disease marked by motor deficits such as myotonia and resting tremor.^583^ In addition to the traditionally recognized loss of dopaminergic neurons in the substantia nigra and intracellular alpha-synuclein (α-syn) deposition, the brains of PD patients exhibit iron deposition, heightened oxidative stress, and lipid peroxidation damage. These major characteristics are strongly aligned with the features of ferroptosis.^584,585^ Clinical studies have demonstrated a correlation between iron levels in the substantia nigra and PD symptoms, including movement disorders.^586^ Many PD patients have dysregulated iron regulatory protein expression.^587^ For example, TF and TFR2 expression are upregulated in dopaminergic neurons in PD brains, and downregulated TFR expression has also been linked to the prevention of dopaminergic neuron degeneration.^588,589^

α-syn is functionally associated with iron, calcium, and lipid metabolism, and its aggregation has been found to induce ferroptosis by interacting with cellular membrane and accelerating lipid peroxidation. Excess α-syn oligomers bind to nerve membranes, resulting in changes in membrane conductance and abnormal influx of calcium. Reduced intracellular glutathione levels or increased iron concentrations further exacerbate these pathologic phenotypes.^590^ Furthermore, α-syn oligomers induce lipid peroxidation, while inhibition of lipid peroxidation and reduction of iron-dependent free radical accumulation can reduce the neuronal toxicity induced by α-syn oligomers, suggesting the role of ferroptosis in PD.^591,592^ A recent study has shown that melatonin receptor 1 (MT1) is involved in α-synuclein induced ferroptosis. α-syn aggregation leads to increased iron deposition and ferroptosis in PD mice, while MT1 knockout reduces the resistance of neurons to ferroptosis by inhibiting the SIRT1/Nrf2/HO-1/GPX4 pathway and inhibiting the expression of ferritin FTH1, resulting in more ferrous ion release, which further leads to more DA neuron loss and severe dyskinesia.^593^ Bioinformatic analysis of the GSE49036 dataset suggested that the NEAT1-PIK3CA/ATM ceRNA network may be a specific biomarker of α-syn driven ferroptosis.^594^ Transferrin, high mobility group protein 1 (HMGB1), and CP are ferroptosis-related factors associated with PD in the substantia nigra.^584^ These molecules may serve as predictive clinical outcome markers and therapeutic targets for PD patients.

Another characteristic of PD is the loss of dopaminergic neurons in the substantia nigra and the subsequent decrease in dopamine levels in the substantia nigra striatum. Dopamine is also a potent suppressor of ferroptosis, and thus, its reduced levels in PD may increase cellular susceptibility to ferroptosis. Dopamine could target GPX4 to enhance its stability and inhibit iron-mediated ROS production.^595^ These results further confirm the relationship between ferroptosis and PD, providing new perspectives and potential pharmacological targets for PD.

With the increase of α-syn expression and the loss of dopaminergic neurons, cholesterol content and triacylglycerol hydrolysis in the brains of PD mice significantly increase. Iron overload induces imbalanced activity of lipolytic enzymes and acylase in mice, resulting in lipid disorder and increased lipid peroxidation.^596^ Fat mass and obesity (FTO) protein and fat mass are closely related to obesity and lipid metabolism. FTO level is upregulated in PD model, while FTO silencing alleviates cell ferroptosis.^597^ In addition, bioinformatics analysis shows that Nrf2 is the target of FTO, and FTO impairs Nrf2 mRNA stability through an m6A dependent pathway, revealing the potential function of FTO in PD neurological diseases.^597^

RNA sequencing analysis showed that FTH1 was abnormally expressed in the PD rat model.^598^ FTH1 knockdown in neurons significantly inhibits cell viability and causes mitochondrial dysfunction, while overexpression of FTH1 impairs ferritinophagy, downregulates the expression of LC3 and NCOA4, and finally inhibits cell ferroptosis.^598^ Nedd4 family interacting protein 1 (Ndfip1), an adaptor protein for the Nedd4 E3 ubiquitin ligase, shows reduced expression in the substantia nigra of PD mouse models.^599^ Ndfip1 overexpression may restore mitochondrial dysfunction in PD mice by regulating voltage-dependent anion-selective channels.^599^ Additionally, Ndfip1 prevents increased expression of ACSL4, significantly improves motor dysfunction, and antagonizes the loss of dopaminergic neurons by inhibiting ferroptosis,^599^ indicating that Ndfip1 may be a promising target for iron-related diseases, including PD.

Consumption of GSH can promote ferroptosis by decreasing SLC7A11 and GPX4 expression in dopaminergic neurons of PD patients.^558^ For example, hypermethylation of cg06690548 on chromosome 4 in PD patients is associated with downregulation of SLC7A11, which may be a possible biological target for PD.^600^

In addition, ACSL4 and ALOX15/15B are critical for iron and PUFA homeostasis-induced ferroptosis in dopaminergic neurons.^601^ ACSL4 expression is increased in dopaminergic neurons of the substantia nigra in both PD patients and animal models. Silencing ACSL4 in the substantia nigra can prevent dopaminergic neuron death and motor defects in PD mice, and inhibition of ACSL4 activity with triacsin C can improve the PD phenotype.^602^

Epigallocatechin-3-gallate (EGCG), the primary polyphenol in green tea, inhibits iron inflow by inhibiting Malvolio expression, and promotes the upregulation of ferritin, which alleviates the increase of free iron levels in the brain of PD Drosophila.^603^ Meanwhile, EGCG downregulates dual oxidase and NOX expression while increasing SOD and GPX4 activities to reduce lipid peroxidation, suggesting that EGCG plays a neuroprotective role mainly by restoring iron homeostasis and redox balance in PD Drosophila.^603^ Salidroside can improve motor function, decrease α-syn expression, and increase tyrosine hydroxylase expression in PD mice, primarily through activating the Nrf2/GPX4 pathway to curb ferroptosis.^604^ Granulathiazole A increases the expression of FSP1, GPX4, SLC7A11, while lowering ACSL4 levels by activating the Nrf2/HO-1 pathway, improving the accumulation of α-synuclein.^605^ Buddlejasaponin IVb (BJP-IVb) is the main active compound in genus *Clinopodium*. BJP-IVb prevents PD by inhibiting iron regulatory protein (IRP2) from iron overload-mediated ferroptosis in dopaminergic neurons, as demonstrated by diminished lipid peroxidation and reduced iron content.^606^ By activating autophagy and inhibiting ferroptosis, rapamycin reduces the loss of dopamine neurons in the substantia nigra and improves behavioral symptoms in PD model mice, suggesting that the mutual regulation of ferroptosis and autophagy may provide therapeutic targets for drug therapy of PD.^607^

Clausenamide, an alkaloid isolated from *Clausena lansium*, interacts directly with Ser663 of ALOX5, blocking the nuclear translocation of ALOX5, alleviating lipid peroxide accumulation and ferroptosis in dopaminergic neurons, significantly improving behavioral deficits in PD mouse models, and providing an attractive strategy for PD treatment.^608^ Morroniside activates the Nrf2/ARE pathway, upregulates GPX4, SLC7A11, FTH1, and FPN expression, increases GSH content, and decreases MDA and iron levels. More importantly, morroniside repairs mitochondrial damage, restores mitochondrial respiratory chain, inhibits ROS production, and protects dopaminergic neurons from ferroptosis in PD.^609^ Na^+^/K^+^-ATPase (NKA) is a key regulator of ferroptosis and mitochondrial autophagy. In the PD model, DR-AB, an antibody that targets the DR region of the NKA-α subunit, disrupts the cytoplasmic interaction between NKA-α1 and Parkin, promotes Parkin translocation to mitochondria, and enhances mitochondrial autophagy.^610^ In addition, DR-AB enhances the formation of the cell membrane NKA-α1 /SLC7A11 complex and inhibits SLC7A11-dependent ferroptosis.^610^

At present, the pathogenesis and treatment of ferroptosis are hot topics in the field of PD research, indicating that the targeted treatment of ferroptosis is a potential method to prevent and treat PD.^611^ Clinical research on the treatment of PD by targeting ferroptosis is still in its preliminary stages, and many clinical trials are necessary to validate the efficacy of targeted ferroptosis therapy.

#### Ferroptosis in Huntington’s disease

HD is an autosomal dominant, progressive neurodegenerative disease characterized by involuntary movements, behavioral difficulties, and progressive dementia.^612^ However, there are currently no effective interventions for HD patients.^613^ The molecular pathogenesis of HD is complex, mainly due to mutations in the Huntington protein. Several features of ferroptosis, such as iron accumulation,^614,615^ glutathione depletion,^616^ and lipid peroxidation,^617,618^ are also found in HD patients and animal models, suggesting that ferroptosis may be involved in the regulation of HD pathogenesis.^619,620^

The first direct study of trace metal changes in the brains of post-mortem HD patients by Dexter et al. found a 56% increase in iron in the caudate nucleus and a 44% increase in the putamen compared to the control group.^621^ Immunohistochemical analysis of postmortem HD brain sections by Simons et al. found that iron metabolism in the brain was disturbed in HD patients and cellular ferritin in the striatum was increased in early HD patients.^622^ Clinical imaging showed increased iron signaling in the basal ganglia and decreased iron in the white matter and cortex in HD patients.^623^ Synchrotron X-ray fluorescence analysis showed that iron accumulated in the perinuclear cytoplasm of the striatal neurons of HD rats in the form of discrete puncta.^624^ The expression of iron response proteins IRP1, IRP2 and TFR was decreased, and the expression of FPN was increased, and the iron-chelating agent DFO could improve the motor phenotype and cognitive function of HD mice.^624^ In addition, the ferroptosis inhibitor Fer-1 significantly inhibits oxidative lipid damage and ferroptosis in neurons of HD cell models.^625^ RNAi-mediated screening revealed that ALOX5 is the primary factor required for ACSL4-independent ferroptosis induced by mutant huntingtin protein HTTQ94.^626^ The absence of ALOX5 expression eliminates HTTQ94-induced ferroptosis under oxidative stress conditions, suggesting that ALOX5 is essential for ferroptosis mediated by mutant huntingtin and could be a promising new target for HD treatment.^626^

However, research on the relationship between HD and ferroptosis is still limited. Additional in vivo and in vitro studies are necessary to verify the involvement of ferroptosis in HD-specific pathologic mechanisms and development.

#### Ferroptosis in major depressive disorder

Major depressive disorder (MDD), a psychiatric disorder marked by a persistent low mood and loss of interest, has become a leading cause of mental and physical disability worldwide and is widely recognized as one of the most urgent mental health issues.^627^ Traditionally, MDD was attributed to neurotransmitter alterations or brain-derived neurotrophic factor disorders; however, recent research has revealed a significant connection between oxidative stress, ferroptosis in the brain, and depression.^628^

Epidemiological and animal studies have shown that the imbalance of metal ion homeostasis can cause disturbance in emotional regulation.^629,630^ Recent findings have identified ferroptosis in the hippocampus of mice subjected to chronic mild and unpredictable stress (CUMS)-induced major depression, and suggest that the occurrence of depression may be related to ferroptosis-associated pathways.^631,632^ Cao et al. conducted mass spectrometry analysis of hippocampal protein expression in CUMS-depressed mice, and the results showed that ferroptosis was significantly activated in the enriched pathways.^631^ Abnormal lipid metabolism is more common in patients with depression. Excessive lipid accumulation in depressed patients can increase ROS levels and decrease antioxidant capacity. Clinical research has demonstrated that elevated lipid peroxidation is associated with a higher risk of treatment-resistant depression, while antidepressants like fluoxetine and citalopram have been found to reduce MDA levels in MDD patients.^633^ Chronic social defeat stress-induced depressed mice showed iron accumulation and iron homeostasis imbalance in the brain, accompanied by downregulation of GSH levels and GPX4 expression, which may be a potential mechanism of lipid peroxidation and ferroptosis in depressed mice.^634^ Fer-1 could alleviate depression-like behaviors in mice subjected to CUMS and promote neuronal growth. The results of small RNA sequencing showed that CUMS caused a disturbance in the expression profile of tRNA-derived small RNA (tsRNA), a novel class of non-coding small RNAs, in mouse hippocampus. Fer-1 alleviates abnormal expressions of tsRNA, with tsRNA-3029b being an effective target, and inhibiting tsRNA-3029b can inhibit ferroptosis and promote neuronal regeneration.^635^ The depressed mice induced by intermittent alcohol consumption showed significant iron deposition, increased TFRC expression, and decreased GPX4 expression in the hippocampus and prefrontal cortex.^636^ In addition, Fer-1 significantly inhibits alcohol-induced neuronal ferroptosis, suggesting that alcohol exposure-induced depression and anxiety-like behavior may be caused by neuronal ferroptosis.^636^ Protein arginine methyltransferase 2 (PRMT2) is involved in the regulation of β-catenin through arginine methylation, which promotes proteasomal degradation of β-catenin, leading to transcriptional inhibition of GPX4. Thus, the upregulation of PRMT2 activates ferroptosis via the β-catenin/GPX4 axis and participates in lipopolysaccharide-induced depressive behavior.^637^

Recently, Chinese herbal formulas or several natural products have been shown to improve depressive behavior by inhibiting ferroptosis. For instance, Chinese formula Xiaoyaosan alleviates depression-like behaviors by modulating PEBP1-GPX4-mediated ferroptosis in the hippocampus of CUMS mice, implying that ferroptosis could become a novel target for development and research of antidepressant drugs.^632^ Di-Huang-Yin-Zi effectively decreases ROS and MDA levels in prefrontal cortex, increases the expression of ferroptosis-related markers GPX4 and SLC7A11 and, and decreases iron deposition, thereby improving depressive symptoms and synaptic ultrastructure of cortical regions in post-stroke depressed (PSD) rats, suggesting that Di-Huang-Yin-Zi can reduce the symptoms and enhance function in PSD rats by inhibiting ferroptosis through SLC7A11/GPX4 pathway.^638^ Saikosaponin B2 alleviates ER stress by regulating intracellular Ca^2+^ levels, reducing lipid peroxidation and intracellular Fe^2+^ content, and inhibiting ferroptosis in a GPX4-dependent manner, thereby improving CUMS-induced depressive behavior.^639^ Edaravone is a free radical scavenger with brain protective effect. Edaravone inhibits ferroptosis through SIRT1/Nrf2/HO-1/GPX4 signaling pathway, thus playing an anti-depressant and anti-anxiety role.^634^ Chronic constrictive injury (CCI) combined with CUMS can simulate clinical pain and depression comorbidities in the clinic. In the CCI plus CUMS group, iron concentration in the spinal cord tissue of mice was increased, and mitochondrial damage was also observed.^640^ Gallic acid treatment can alleviate depression-like behaviors and pain, and the mechanism may be related to inhibiting ferroptosis by regulating the P2X purinoceptor 7 (P2X7)-ROS signaling pathway in spinal microglia.^640^ Eicosapentaenoic acid and docosahexaenoic acid can also effectively relieve pentylenetetrazol-induced seizures and depression by reducing iron levels in the brain, promoting M2 polarization of microglia, and activating Nrf2.^641^ The mechanism of acupuncture in the treatment of depression has also attracted much attention. Studies have found that acupuncture can effectively reverse the CUMS-induced decline in Nrf2, HO-1, and GPX4 levels in the hippocampus of rats, reduce the activation of astrocytes and microglia, and alleviate depression-like behavior induced by CUMS.^642^ Ketamine may also have a rapid antidepressant effect by increasing FTH1 and GPX4 levels, and reducing TFR1 levels, which in turn inhibits ferroptosis.^643^

Signaling pathways influenced by ferroptosis are implicated in numerous pathological conditions, and their role in depression is likely intricate and multifaceted. Although some studies have shown that inhibiting ferroptosis has an anti-depressive function, the specific mechanisms by which ferroptosis is regulated in MDD remain to be fully elucidated.

#### Ferroptosis in neuropathic pain

Neuropathic pain (NeP) is a prevalent and progressive neurodegenerative disease, usually characterized by spontaneous or induced pain and pain sensitization.^644^ Beyond the well-known mechanisms of central sensitization and peripheral nerve injury, NeP is also characterized by oxidative stress, excessive iron accumulation, and lipid peroxidation damage.^645^ Transcriptome sequencing of neuropathic pain mice showed that TRIM28 is a crucial regulator of ferroptosis, and TRIM28 regulates iron homeostasis and ferroptosis in spinal microglia by downregulating GSK3β expression and regulating autophagy.^646^ Spermidine/spermine N1-acetyltransferase 1 (Sat1) and ALOX15 were highly expressed in the spinal cords of mice with neuropathic pain induced by CCI.^647^ Iron accumulation, dysregulation of ACSL4 and GPX4 expression, and increased lipid peroxidation were observed in the spinal cord of NeP mice induced by CCI. Transmission electron microscopy shows that the mitochondria of dorsal horn neurons of the spinal cord exhibit characteristic changes of ferroptosis, such as mitochondrial shrinkage and increased mitochondrial inner membrane density.^648^ In addition, treatment with liproxstatin-1 mitigates hypersensitivity reactions, reduces iron levels, reduces spinal cord lipid peroxidation, and prevents CCI-induced mitochondrial morphological changes,^648^ suggesting that ferroptosis may be a potential therapeutic target for neuropathic pain. In the rat model of neuropathic pain induced by CCI, Fer-1 upregulates GPX4 expression, downregulates ACSL4 expression, and decreases the content of iron in the spinal cord, while erastin has the opposite effect.^645^ The levels of ROS and MDA in spinal cord of mice suffering from bone cancer pain increase, while those of SOD decrease, accompanied by mitochondrial ultrastructural changes.^649^ Fer-1 attenuates ferroptosis-associated iron accumulation and lipid peroxidation, and reduces pain in mice with bone cancer,^649^ suggesting that ferroptosis is a potential therapeutic target common to bone cancer pain and other types of pain. Electroacupuncture can inhibit ferroptosis and treat neuropathic pain by regulating the SAT1/ALOX15 pathway, providing a new therapeutic target for neuropathic pain.^647^ Gallic acid inhibits ferroptosis in spinal microglia by regulating the P2X7-ROS signaling pathway, alleviates pain in rats, and reverses the CCI-induced increase in iron concentration and mitochondrial damage.^640^ NOX4-induced oxidative stress promote ferroptosis and neuropathic pain. The expression of NOX4, ACSL4 and levels of ROS and iron are increased in the spinal cord of rats subjected to spared nerve injury, while the expression of GPX4 is decreased.^650^ Methyl ferulic acid inhibits the expression of NOX4 protein and alleviates neuropathic pain in mice by regulating the expression of ACSL4 and GPX4, as well as ROS and iron content, suggesting that inhibiting NOX4-induced ferroptosis can reduce neuropathic pain.^650^

#### Ferroptosis in traumatic brain injury

Traumatic brain injury (TBI) is a significant global cause of disability and mortality,^651^ with complex underlying mechanisms such as excitatory neurotoxicity, apoptosis, oxidative stress, inflammatory response, and intracellular calcium overload.^652,653^ Although the primary TBI caused directly by external forces is irreversible, the secondary TBI can be alleviated. Therefore, reducing neuronal death is crucial for the treatment of secondary TBI. Recent research indicates that altered iron metabolism is a key factor in secondary damage after TBI. Due to iron’s hydrophilic properties, iron needs to traverse the BBB to enter the brain parenchyma from the circulating blood, and BBB disruption and intracranial hemorrhage in TBI patients lead to iron deposition in the brain, disrupting iron metabolism.^654,655^ Perls staining and measurements of non-hemoglobin iron revealed iron overload in the ipsilateral cortex of TBI mice and brain microvascular endothelial cells (BMECs).^656^ Xie et al. found that accumulation of iron and ROS, dysfunction of iron metabolism, decreased GPX4 activity, and mitochondrial atrophy after TBI are all typical characteristics of ferroptosis.^657^ Importantly, intraventricular injection of Fer-1 significantly decreases iron deposition and neuronal degeneration, improving motor and cognitive function.^657^ Rui et al. observed temporal changes in ferroptosis-related molecular expression and iron accumulation in the ipsilateral cortex following TBI.^658^ Melatonin, the primary hormone of the pineal gland, has numerous beneficial effects on TBI. FTH neuron-specific knockout (FTH-KO) mice are more susceptible to ferroptosis after TBI, and the absence of FTH offsets the protective effect of melatonin on ferroptosis induced by TBI,^659^ suggesting that melatonin plays a protective role at least in part by inhibiting FTH-mediated ferroptosis in neurons after TBI. Subsequent studies found that melatonin inhibited inflammation and ferroptosis mainly by activating MT2 and IL-33 pathways, and FTH-KO significantly exacerbated the inflammatory response after TBI, and eliminated the anti-inflammatory effects of melatonin.^659^

The level of GPX4 in the brain gradually decreases after TBI, and overexpression of GPX4 can partially reverse TBI-induced hippocampal ferroptosis and synaptic damage, effectively alleviating neuronal dysfunction after cerebral hemorrhage, and improving TBI-induced cognitive deficits.^660^ On the contrary, inhibition of GPX4 with specific pharmacological inhibitors or GPX4 knockdown can aggravate secondary brain injury after cerebral hemorrhage.^661–663^ Selenium (Se) promotes GPX4 expression and protects neurons by activating the transcription factors SP1 and TFAP2c, suggesting that pharmacological supplementation of selenium can effectively inhibit GPX4-dependent ferroptosis.^664^ Annexin A5 (A5) has anticoagulant and anti-inflammatory biological activities. By regulating the Nrf2/HO-1 pathway, A5 improves the activity of SOD and GPX and the level of glutathione, reduces the levels of iron deposition, 4-HNE and MDA, thereby improving the ferroptosis of neurons induced by TBI.^665^ In addition to GPX4, FSP1 plays an important role in inhibiting ferroptosis. Inhibition of the FSP1 pathway can weaken the protective effect of edaravone on TBI, suggesting that edaravone inhibits ferroptosis induced by injury in early TBI by activating FSP1 pathway.^666^

Ultra-high performance liquid chromatography-mass spectrometry analysis showed that moderately controlled cortical impact induces significant expression of PUFA in mouse serum, leading to lipid peroxidation and ferroptosis.^667^ Controlled cortical impact results in increased expression of ACSL4 and ALOX15, as well as ipsilateral cortical GSH consumption, suggesting that the ferroptosis pathway may be a valuable therapeutic target after TBI.^668^

Iron deposition, GSH depletion, and lipid peroxidation in ferroptosis are intricately linked to neurodegeneration and nerve function impairment after TBI. Targeting ferroptosis may offer valuable insights for developing of TBI therapies. Based on iron overload observed in the brain tissue of TBI patients, removal of excess iron could be an effective strategy to improve their prognosis.^669^ Clinical trials have demonstrated that DFO can facilitate the absorption of edema and hematoma after TBI, reduce neuronal degeneration and phospholipid injury.^670,671^ In a TBI mouse model, DFO has been shown to mitigate acute brain edema and long-term neurotoxicity induced by iron deposition, improve grip strength and prognosis in mice.^672,673^ DFO treatment significantly reduces iron accumulation in brain tissue and BMECs, increases brain capillary density, and improves cerebral blood flow and behavioral performance.^656^ This suggests a potential new therapeutic approach for preventing cerebral vascular dysfunction after brain injury. Ferristatin II, an inhibitor of iron uptake, also provides neuroprotective effect against TBI by suppressing ferroptosis. N, N′-bis (2-hydroxybenzyl) ethylenediamine-N, N′-diacetic acid (HBED) binds to Fe^2+^, converting them to Fe^3+^ and reducing brain damage caused by Fe^2+^.^674^ In a TBI mouse model, HBED treatment can reduce microglia proliferation and aquaporin 4 expression, thereby alleviating secondary injury in the cortical region after TBI and improving motor function in mice.^675^

Targeting Nrf2 has also become a research hotspot in TBI therapy due to its capacity to reduce oxidative stress and inhibit ferroptosis. The protein levels of FSP1, FTH, and FTL, which are crucial for redox balance and iron metabolism, are also significantly regulated by Nrf2 after TBI.^676^ Netrin-1 is a secreted laminin closely related to nerve regeneration.^677^ Netrin-1 upregulates GPX4 and prevents ferroptosis post-TBI through the UNC5B/Nrf2 signaling pathway.^677^ Ketamine also exhibits neuroprotective effect in alleviating oxidative stress after TBI through the activation of Nrf2.^678^ Curcumin, a natural phenolic compound, alleviates neuronal apoptosis and cortical damage in mice after TBI; however, its neuroprotective benefits are diminished in Nrf2 knockout mice.^679^ Tert-butylhydroquinone (TBHQ), as an Nrf2 activator, has been shown to activate Nrf2-related pathways in a TBI mouse model by combining vanillin acetate and TBHQ, reducing oxidative stress and protecting gray matter in the mouse brain.^680,681^ The small molecule natural compound hinokitiol alleviates TBI brain tissue injury and significantly improves nerve function by activating the Nrf2/Keap1/HO-1 pathway, and neuronal loss and iron deposition were improved after hinokitiol intervention.^682^

In addition, electroacupuncture promotes the synthesis and metabolism of GSH in the injured area of the cerebral cortex by activating the Xc^−^/GSH/GPX4 axis, thereby inhibiting nerve ferroptosis in rats with TBI.^683^ Intermittent fasting (IF) has been reported to reduce lipid peroxidation and mitochondrial dysfunction, and one month of IF increased the expression of protective GPX4, partially eliminated the ferroptosis of cortical neurons induced by TBI, and helped to alleviate cognitive impairment.^684^ Mesenchymal stromal cell therapy alleviates persistent cognitive deficits caused by repetitive mild TBI by inhibiting ferroptosis, indicating it may be an effective TBI therapy strategy targeting ferroptosis.^685^

However, despite the potential of iron chelating agents, Nrf2 activators and dietary interventions to improve TBI, most of the existing research has been performed on animal models. Therefore, there is a pressing need for more comprehensive experimental research and follow-up clinical trials to establish robust evidence for their clinical application.

#### Ferroptosis in spinal cord injury

Spinal cord injury (SCI) is characterized by high mortality and disability rate, which causes a heavy economic burden to patients and society.^686^ Current understanding of the molecular mechanisms of acute traumatic SCI is limited, and there are no effective treatments. The acute stage of traumatic SCI includes immediate bleeding, accumulation of ROS, and lipid peroxidation,^687^ suggesting that the ferroptosis pathway may play an important role in the secondary injury of SCI. Mitochondrial atrophy was observed under transmission electron microscopy 15 minutes after SCI, which is a typical morphological feature of ferroptosis, becoming more obvious one day later.^688^ The levels of ACSL4 and MDA increased one day after SCI, while GSH levels decreased.^689^ With the consumption of GPX4, SLC7A11 and GSH, a significant increase in total iron and lipid peroxidation could be detected within 2 weeks.^690^ Bioinformatics analysis and qPCR results of rat spinal cord tissue showed that mRNA levels of PTGS2, JUN, RELA, ATF3, TLR4, HMOX1, and STAT3 were upregulated, while mRNA levels of MAPK9, MAPK1, and VEGFA were downregulated.^689^ Clinical analyses of differences in the expression of ferroptosis-related genes in blood samples from SCI patients and healthy controls confirmed the upregulation of TLR4, STAT3, and HO-1, consistent with the bioinformatics results.^691^ These candidate genes and pathways may become therapeutic targets for SCI.

Iron overload in the spinal cord and subsequent neuronal ferroptosis are key factors leading to axonal disruption and failure of neuronal regeneration. Regulating cellular iron homeostasis after SCI by chelating excess iron ions and modulating iron transport pathways can promote the differentiation of neural stem/progenitor cells into neurons and stimulate the regenerative potential of newborn neurons, accompanied by improvements in axonal reinnervation and myelin regeneration.^692^ HO-1 is a regulator of iron and ROS homeostasis. The expression of HO-1 in spinal cord tissue increases rapidly, aggravating ferroptosis after SCI. Fibroblast growth factor 21 (FGF21) inhibits ferroptosis by downregulating HO-1. In addition, FGF21 treatment significantly reduces ACSL4 and iron deposition, increases GPX4 expression, and reduces ferroptosis in nerve cells by activating the FGFR1/β-Klotho pathway.^693^ These findings suggest that FGF21 may be a new therapeutic target for SCI neurorehabilitation, and activation of FGF21 may provide a potential treatment for SCI. However, the role of HO-1 in SCI is still controversial. USP7 has been reported to affect the stability of HO-1 by regulating the ubiquitination of HO-1. In SCI rat models, USP7 expression is downregulated and HO-1 expression is upregulated.^694^ Overexpression of USP7 increases the level of HO-1, inhibits ferroptosis, alleviates spinal cord injury, and finally promotes the recovery of motor function in SCI rats.^694^ The seemingly contradictory role of HO-1 in spinal cord tissue after SCI injury may be related to the time of injury, and HO-1 may play different roles in the acute and chronic stages of injury. Rats with chronic compression SCI showed the most severe behavioral and electrophysiological dysfunction at 4 weeks after compression, with partial recovery at 8 weeks. The ferroptosis pathway is enriched at both 4 and 8 weeks after chronic compression SCI, and MDA content peaked at 4 weeks after chronic compression, decreased at 8 weeks, and was negatively correlated with behavioral scores.^695^ On the contrary, GPX4 expression in neurons was downregulated at 4 weeks after spinal cord compression and upregulated at 8 weeks,^695^ suggesting that it may promote functional recovery after chronic compression SCI. These results suggest that ferroptosis-related proteins may play different roles in different periods after SCI injury.

Oligodendrocytes in the white matter of the spinal cord are rich in unsaturated fatty acids and are susceptible to damage caused by ferroptosis. After SCI, the expression levels of DMT1 and TFR in the white matter of the spinal cord are significantly increased, resulting in iron deposition.^696^ Hepcidin treatment decreased the expression levels of DMT1 and TFR to reduce the accumulation of iron, thus promoting the survival of oligodendrocytes, reducing spinal cord atrophy, and promoting functional recovery.^696^ Fer-1 can also reduce the accumulation of iron and ROS, downregulate the ferroptosis-related genes PTGS2 and their products, thereby inhibiting the ferroptosis of oligodendrocytes, and finally alleviating the white matter injury after SCI in rats and promoting functional recovery.^688^

GPX4 is the central regulator of ferroptosis, and in the spinal cord, GPX4 is mainly expressed in neurons and oligodendrocytes. Neuronal GPX4 is downregulated after SCI, and ferroptosis inhibitors SRS 16-86 and DFO both prevent the reduction of GPX4 and improve the survival rate of neurons in the injured spinal cord to repair SCI-associated motor dysfunction.^690,697^ GPX4 is localized in the nucleus of oligodendrocytes, and liproxstatin-1 can restore the expression of GSH and GPX4 and inhibit mitochondrial lipid peroxidation, thus reducing the ferroptosis of oligodendrocytes, which is also a way to improve SCI.^698^ Selenium is closely related to GPX4 and protects GPX4 from irreversible inactivation.^699^ Sodium selenite significantly reduces iron concentrations and levels of MDA and 4-HNE, and increases GPX4 expression, promoting the survival of neurons and oligodendrocytes and the recovery of motor function in rats with SCI.^700^

Microglia have been shown to be key players in the immune inflammatory response after SCI. Genes related to ferroptosis were found to be differentially expressed in microglia after SCI, with the most significant changes in Stmn1 and Fgfbr1.^701^ These genes can regulate the production of cytokines and participate in the inflammatory response after SCI. Maintaining the integrity of the blood-spinal barrier is essential for the recovery of spinal cord. SCI induces ferroptosis of vascular endothelial cells and destroys the integrity of the blood-spinal cord barrier. Liproxstatin-1 maintains the integrity of the blood-spinal barrier by upregulating the expression of tight junction proteins and reduces ferroptosis in endothelial cells by upregulating GPX4 and downregulating ACSL4 and ALOX5.^702^ These results suggest that liproxstatin-1 improves recovery from SCI by inhibiting endothelial cell ferroptosis and maintaining the integrity of the blood-spinal barrier.

In addition to neuron ferroptosis at the lesion site, SCI causes motor cortex atrophy and functional changes early in the disease, and motor neuron death is thought to be the cause of primary motor cortex atrophy after SCI. In SCI patients and rats, iron deposits in the motor cortex are significantly increased, triggering the accumulation of ROS, and eventually leading to ferroptosis in motor neurons.^703^ Additionally, motor cortical microglia are activated after SCI, inducing iron overload in motor neurons.^703^ Ferroptosis inhibitors, including iron chelating agents and ROS inhibitors, reduce motor neuron death caused by iron overload and promote motor function recovery.^703^ These findings may lead to new treatment strategies for SCI.

Based on the role of ferroptosis in SCI, natural compounds and related molecules targeting ferroptosis show great potential in preclinical treatment. Celastrol, a widely used antioxidant drug, can reduce ROS accumulation by upregulating the Nrf2/GPX4 axis, thus significantly inhibiting ferroptosis of neurons and oligodendrocytes, thereby promoting the recovery of spinal cord tissue and motor function in rats with SCI.^704^ Another antioxidant, resveratrol, also inhibits iron accumulation and lipid peroxide production by activating the Nrf2/GPX4 signaling pathway, thereby inhibiting neuronal ferroptosis and promoting the recovery of motor function in mice.^705^ Albiflorin not only inhibits neuronal ferroptosis by reducing the levels of lipid peroxide and iron and regulating ferroptosis-related proteins, but also inhibits microglia activation and reduces the production of pro-inflammatory cytokines, thereby promoting motor function recovery after SCI in rats.^706^ Metformin therapy activates the Nrf2 signaling pathway and improves SCI-induced motor dysfunction by inhibiting ferroptosis and the inflammatory response.^707^ HO-1 is also involved in the beneficial effects of metformin on ferroptosis of neurons after SCI.^708^ Proanthocyanidin treatment significantly decreases the levels of iron, ACSL4, and ALOX15 in spinal cord tissue of SCI mice, while increasing GSH, GPX4, Nrf2, and HO-1, improving motor function of SCI mice.^709^ These indicate that proanthocyanidins promote functional recovery of SCI by inhibiting ferroptosis. Zinc can activate the Nrf2/HO-1 pathway, increase the contents of GPX4, SOD and GSH, reduce the levels of lipid peroxides, MDA and ROS, and effectively reverse the behavioral and structural changes after SCI.^710^ Erythropoietin inhibits ferroptosis and improves nerve function after SCI by increasing xCT and GPX4 expression. The small molecule drug CA-074-methyl ester reduces lipid peroxidation and mitochondrial dysfunction in macrophages by inhibiting cathepsin B, thereby alleviating ferroptosis and inducing polarization of M2 macrophages, promoting the recovery of nerve function in mice after SCI.^711^

### Ferroptosis in ischemia-reperfusion injury

#### Ferroptosis in cerebral ischemia/reperfusion injury

Ischemic stroke, a brain disorder caused by an inadequate blood supply, accounts for about 87% of all stroke events.^712,713^ Ischemic stroke patients experience a decline in their ability to perform daily activities and overall cognitive function, with a 157% increase in dementia.^714,715^ Therefore, ischemic stroke has become a serious medical and social challenge. When an ischemic stroke occurs, the lack of blood and glucose leads to insufficient energy supply, causing the death of neurons in the infarcted brain area. More importantly, the reperfusion or restoration of blood flow after ischemia can also induce the increase of ROS and lipid peroxidation, leading to tissue neuronal damage and worsening of the inflammatory response.^716,717^ Interestingly, MRI confirmed higher iron levels in stroke patients, and iron levels in the brain were associated with the severity of cerebral infarction, while iron-dependent lipid peroxidation, or ferroptosis, occurred in neurons in the infarct area during ischemic stroke.^718^ A prospective study evaluating the relationship between plasma ferroptosis biomarkers and prognosis during the hyperacute phase of endovascular thrombectomy (EVT) in patients with acute ischemic stroke found that stroke patients had higher levels of 4-HNE before and after EVT and lower levels of soluble transferrin receptor (sTFR) 24 h after EVT compared with controls.^719^ The stroke scale at admission is proportional to the 4-HNE level and inversely proportional to the sTFR level, suggesting that ferroptosis is associated with stroke severity and prognosis in acute ischemic stroke patients receiving EVT.

Under normal physiological conditions, the BBB separates the central nervous system from the peripheral vascular system, thereby maintaining stable iron levels within the brain. During cerebral ischemia, the integrity of the BBB is impaired and free iron from circulation enters the brain.^720^ Cerebral ischemia also increases levels of circulating iron-carrying transferrin in ischemic brain tissue, enhancing ROS induced neuronal death.^721^ A comprehensive nutrition study found that iron intake increased ischemic stroke mortality in Japanese men,^722^ and higher serum ferritin levels were linked to a higher risk of ischemic stroke in type 2 diabetes patients and postmenopausal women,^723,724^ suggesting that peripheral iron accumulation is a risk factor for ischemic stroke. The higher incidence and poorer prognosis of ischemic stroke in older adults may also be associated with iron deposits in the brain.^725^ A randomized clinical trial has found that intravenous administration of the iron-chelating agent DFO reduces systemic iron levels and may have long-term efficacy in patients with ischemic stroke.^726^ Consistent with clinical observations, unilateral, transient middle cerebral artery occlusion/reperfusion (MCAO/R) caused a significant increase in free iron levels, or ferritin levels, in the brains of mice. 24 h after MCAO/R, iron supplementation directly increased the volume of cerebral infarction in rats,^727^ and iron deposits were detected in microglia at 3 weeks after MCAO/R and in the parenchyma at 7 weeks after MCAO/R.^728^ Conditional knockout of FPN1 in mouse endothelial cells (ECs) reduces the level of iron in the brain, oxidative stress, and ferroptosis after stroke, and finally reduces the volume of cerebral infarction and the nerve function injury in the acute stage of ischemic stroke.^729^ The expression of E3 ubiquitin ligase neural precursor cell expressed developmentally downregulated 4-like (NEDD4L) was downregulated in MCAO/R models, and overexpressed m6A RNA methyltransferase METTL3 enhanced NEDD4L expression by methylating and stabilizing NEDD4L mRNA. Then NEDD4L ubiquitinated and degraded TFR1, thereby reducing oxidative damage and ferroptosis and protecting the brain from ischemic damage.^730^ FtMt protein expression is significantly upregulated in MCAO/R mice, and the absence of FtMt promotes free iron deposition and lipid peroxidation, which aggravates ferroptosis and brain injury caused by MCAO/R.^428^ I/R-induced neuronal injury results in increased expression of NCOA4 in nerve cells. Excessive degradation of ferritin is induced by autophagy, resulting in increased free iron levels in neurons. The loss of NCOA4 significantly abolishes ferritinophagy caused by I/R injury, thus inhibiting ferroptosis.^731^ Pharmacological inhibition of USP14 effectively reduces the level of NCOA4 to protect neurons from ferritin-mediated ferroptosis.^731^ These findings provide new and effective targets for the treatment of cerebral I/R injury.

Due to lower levels of endogenous antioxidants, the brain is also more susceptible to oxidative stress.^732^ Reduced antioxidant capacity is another important feature of ferroptosis. Significantly reduced GPX4 levels in MCAO/R mouse or rat brains and in oxygen-glucose deprivation/reperfusion (OGD/R)-treated hippocampal neurons have the potential to exacerbate lipid peroxidation and ferroptosis.^733,734^ Synaptosome-associated protein (Snap25) is a key molecule that regulates vesicle transport, neurotransmitter release, and neuronal plasticity.^735^ Overexpression of Snap25 can significantly inhibit ferroptosis and reduce acute ischemic stroke injury and OGD/R injury by upregulating GPX4 levels, while Snap25 silencing has the opposite effect.^736^ As a cofactor of GPX4, GSH increases irritability in the serum of patients with acute ischemic stroke within one hour of the onset of ischemia.^737^ The level of GSH in MCAO/R mice decreases with the increase of lipid peroxidation.^738^ Abnormal xCT function causes GSH depletion and sufficiently triggers ferroptosis.^739^ In the MCAO/R model of rats, the expression of SLC7A11 is downregulated, accompanied by inhibition of GSH and GPX4, suggesting that the deficiency of xCT in cerebral I/R injury may be the main cause of lipid peroxidation and ferroptosis.^740,741^ Glutamate transporter modulator ceftriaxone or N-acetylcysteine stabilize the levels of xCT and reduce infarct volume and neurological deficits in rats within 24 h after stroke.^740,741^ It is worth noting that SLC7A11/xCT-mediated cystine uptake is a double-edged sword in cellular oxidative regulation.^742^ In ischemic stroke, sudden hypoxia of neurons triggers a large release of glutamate, which induces hypoxia depolarization and leads to rapid cell death. Electrophysiology results from hippocampal sections showed that xCT-deficient mice have a prolonged latency of hypoxia depolarization after complete hypoxia compared with wild-type mice.^743^ Therefore, the role of xCT in cerebral I/R injury remains controversial. Nrf2 is an important transcription factor involved in iron metabolism and oxidative stress in the brain.^744^ Nrf2 can increase GSH synthesis and GPX4 levels by controlling enzymes associated with GSH synthesis.^745^ Nrf2 activated by tertiary butylhydroquinone increases cortical GSH levels and reduces infarct volume in mice and rats within 24 h after MCAO/R,^746^ and miR-27a aggravates ferroptosis in early ischemic stroke in rats by targeting Nrf2.^747^ Fer-1 analog Srs11-92 (AA9) ameliorates oxidative stress and ferroptosis through Nrf2 signaling, and improves cerebral infarction size, neuronal injury, and neural function deficit in MCAO/R model mice.^748^ Melatonin could improve learning and memory abilities in rats with hypoxic-ischemic brain injury by regulating the AKT/Nrf2/GPX4 signaling pathway.^749^ These findings collectively indicate that the Nrf2/GSH/GPX4 axis could be a promising therapeutic target for cerebral I/R injury.

The brain, compared with other organs, is particularly prone to lipid peroxidation due to the abundance of unsaturated fats and redox-active transition metals.^732,750^ The concentration of 12/15-ALOX in the brain increases significantly after 90 minutes of MCAO/R, and 12/15-LOX knockout mice exhibit reduced cerebral edema and infarct volume compared to wild-type mice following MCAO/R.^751,752^ Furthermore, several inhibitors of 12/15-ALOX, such as brozopine and LOXBlock-1, have been demonstrated to decrease infarct volume and salvage nerve damage in mice.^753,754^ The expression of spermidine/spermidine N1-acetyltransferase 1 (SSAT1) is upregulated in the cortical penumbra of transient MCAO/R mice, and SSAT1 knockdown reduces cortical iron content, ROS production and 4-HNE levels, and alleviates I/R-induced cerebral infarction and nerve injury.^755^ Mechanistically, SSAT1 overexpression increases ALOX15 expression and decreases the expression levels of GPX4 and SLC7A11 in primary cortical neurons, suggesting that SSAT1/ALOX15 axis activation may aggravate brain I/R injury by triggering ferroptosis in neurons.

ACSL4 is considered to be an important inducer of ferroptosis during cerebral I/R injury. In the early stages of ischemic stroke, ACSL4 expression is suppressed, which is induced by HIF-1α.^756^ Overexpression of ACSL4 exacerbates cerebral I/R injury in rodents, while genetic or pharmacological reduction of ACSL4 effectively prevents cerebral I/R injury.^756,757^ The combination of circular RNA Carm1 and miR-3098-3p regulates the expression of ACSL4 and has a protective effect on acute cerebral infarction injury.^758^ cPLA2α is highly expressed in ischemic stroke patients and is positively correlated with injury degree and infarct size. In cerebral I/R injury, increases in thrombin in neurons activate cPLA2α and promote the release of arachidonic acid, which is esterified by ACSL4 and utilized as a ferroptotic fuel.^759^ The E3 ligase RING finger protein 146 (RNF146) is responsible for ubiquitination and degradation of ACSL4. Overexpression of RNF146 inhibits OGD/R-induced increases in MDA and Fe^2+^, as well as the expression of ferroptosis related genes. ATF3 can activate transcription and expression of RNF146, thereby inhibiting OGD/R-induced neuronal ferroptosis.^760^ These results together suggest that ACSL4 and LOX-catalyzed lipid peroxidation is involved in cerebral I/R injury, and inhibition of ACSL4 and LOX activity may be therapeutic targets for cerebral I/R injury. Thrombolysis remains a major strategy for the treatment of ischemic stroke. With a deeper understanding of ferroptosis during the occurrence and progression of cerebral I/R injury, new therapies to treat ischemic nerve damage by interfering with the molecular mechanism of ferroptosis are expected to be realized to some extent.

Many small molecules have been found to effectively improve prognosis and cerebral infarction by inhibiting iron accumulation and lipid peroxidation. For instance, baicalein inhibits ferroptosis by regulating the GPX4/ACSL4/ACSL3 axis and ameliorates cerebral I/R injury.^761^ The overexpression of CYP1B1 increases the ubiquitination and degradation of ACSL4, and melatonin could inhibit ferroptosis by regulating the ACSL4/CYP1B1 pathway, significantly reducing the cerebral ischemic area and neuron loss in MCAO mice.^762^ Ecdysterone, one of the main active ingredients of *Achyranthes bidentata Blume*, inhibits ferroptosis via ACSL4, and improves oxidative damage in oxygen-glucose deprivation/reperfusion (OGD/R)-treated PC12 cells and in MCAO rats.^763^ Cottonseed oil significantly improves MCAO/R induced neurological dysfunction in male rats by reducing infarct size and neuron damage and maintaining BBB integrity. The mechanism may be related to the decreased inflow of iron, TF and TFR1, the upregulation of GPX4, xCT, and FTH1, and the downregulation of ACSL4, MDA and LPO levels.^764^ Astragaloside alleviates neuronal ferroptosis in ischemic stroke by modulating m6A levels of ACSL4 and fat mass.^765^ In addition, caffeic acid,^766^ β-caryophyllene,^767^ 15, 16-dihydrotanshinone,^768^ icariside II,^769^ rehmannioside A,^770^ kaempferol,^771^ rhein,^772^ loureirin C,^773^ quercetin,^774^ and kellerin^775^ suppress ferroptosis induced by cerebral I/R injury by activating the Nrf2 signaling pathway, which further suggests that activating the Nrf2 signaling pathway may be a potential treatment for relieving cerebral I/R injury.

In addition to small molecule antioxidants, traditional Chinese medicinal formulations have been widely used in the treatment of cerebral I/R. For example, Angong Niuhuang Wan inhibits the accumulation of ROS and Fe^2+^ and improves mitochondrial dysfunction and BBB structural integrity by activating the PPARγ/AKT/GPX4 pathway, thereby enhancing neurological function in rats with ischemic stroke and reducing cerebral infarction volume.^776^ Danhong injection can significantly reduce the cerebral infarction size and related injury in pMCAO (permanent MCAO) rats, and improve the activity of OGD-damaged neurons.^777^ The mechanism may be related to the activation of transcription factor SATB1, upregulation of the SLC7A11/HO-1 signaling pathway, and improvement of neuronal ferroptosis.^777^ Danlou tablet significantly inhibits ferroptosis by reducing oxidative stress and COX2 levels and increasing SLC7A11 and GPX4 levels, thereby attenuating BBB injury and ischemic stroke injury.^778^ Salvia miltiorrhiza significantly reduces the levels of 4-HNE and MDA in the brain penumbra of tMCAO mice and alleviates the cerebral infarction and neurological dysfunction caused by tMCAO (transient MCAO) by inhibiting ferroptosis.^759^ Z-Guggulsterone (Z-GS) and 11-keto-β-boswellic acid (KBA) are the main active substances of Chinese herbs *Myrrh* and *Frankincense*. Single cell transcriptome results showed that 14 cell types were identified in ischemic penumbra, of which astrocytes and microglia accounted for the largest proportion. KBA and Z-GS synergistically regulate the inflammatory response of microglia and ferroptosis of astrocytes by regulating SLC1A2 and TIMP1.^779^ Other Chinese medicine strategies such as Naotaifang^734^ and electroacupuncture^780^ could improve the neural behavior of animals by regulating iron metabolism and increasing antioxidant capacity to protect neurons from ferroptosis induced by cerebral I/R injury.

Remote ischemic postconditioning (RIPostC) can protect many organs from ischemia. In a rat model of MCAO, RIPostC reverses GPX4 reduction and ACSL4 overexpression by increasing ketone body production, thereby alleviating nerve damage.^781^ In addition, both RIPostC and ketone bodies reduce total iron and ferrous ion content by inhibiting FPN.^781^ Bioinformatics analysis showed that CHAC1 is a key gene in the process of ferroptosis in patients with ischemic stroke. Exosomes from adipose-derived mesenchymal stem cells (ADSC-Exos) can effectively improve the neurobehavioral function of mice after I/R. Mechanically, ADSC-Exos are effectively delivered to the brain through the intranasal administration and is enriched with miR-760-3p to downregulate the expression of CHAC1 and inhibit ferroptosis.^782^ Exosomes secreted by human umbilical cord mesenchymal stem cells (HUC-MSCs) are considered to be an effective treatment for ischemic stroke. HUC-MSCs-derived exosomes enhance the viability of hypoxia/reperfusion (H/R)-exposed cells and inhibit ferroptosis.^783^ Mechanically, HUC-MSCs-derived exosomes inhibit ferroptosis by delivering circBBS2, which sponges miR-494 and enhances SLC7A11 levels, thereby inhibiting ferroptosis and alleviating ischemic stroke.^783^ Stem-cell derived exosome therapy targeting ferroptosis is proved to be a novel strategy against cerebral I/R injury. Exercise interventions have been shown to help restore physical function after stroke and improve prognosis.^784,785^ Pre-stroke exercise intervention alleviates stroke-induced ferroptosis by reducing the production of LPO, upregulating GPX4 and SLC7A11, and downregulating ACSL4, thereby reducing the size of cerebral infarction and improving the neurological function of ischemic stroke rats. High-throughput sequencing and dual-luciferase reporter gene analysis show that exercise induces an increase of skeletal muscle exosomes and exosomal miR-484 could enter the brain through blood circulation to inhibit the expression of ACSL4 and thus inhibit ferroptosis of nerve cells.^786^ Exercise intervention before stroke that increases skeletal muscle-derived exosomes is also an effective treatment strategy for cerebral I/R injury.

#### Ferroptosis in spinal cord ischemia-reperfusion injury

Spinal ischemia-reperfusion injury (SCIRI) is a common complication after thoracic and abdominal aortic surgery, and can also be caused by spinal trauma and spinal degeneration, leading to severe sensory and motor dysfunction.^787^ Ferroptosis has been shown to be associated with the pathological mechanism of SCIRI, and Fer-1 inhibits ferroptosis of spinal neurons in rats through the ERK1/2/SP1/GPX4 signaling pathway, improving nerve function after SCIRI.^788^ Ubiquitin-specific protease 11 (USP11) is significantly upregulated in the spinal cord of mice with I/R injury and neuronal cells after hypoxia-reoxygenation. USP11 knockdown significantly reduces ferroptosis of neuronal cells and promotes motor function recovery in mice after SCIRI.^789^ Conversely, overexpression of USP11 results in heightened ferroptosis in neurons and impaired functional recovery following SCIRI.^789^ USP11 facilitates autophagy activation by stabilizing beclin-1, which may be the potential mechanism by which USP11 enhances ferroptosis.^789^

Tyrosine kinase Eph receptor A4 (EphA4) is notably expressed in the nervous system.^790^ After SCIRI, the permeability of the blood-spinal barrier increases, the expression of EphA4 in spinal dorsal horn neurons increases, and the number of mitochondria showing ferroptosis characteristics increases significantly. Inhibition of EphA4 expression reduces the binding of Beclin1 and p-ERK1/2, significantly reduces the formation of Beclin1-xCT complex, and decreases the expression of c-Myc, TFR1 and p-ERK1/2, thereby largely preventing the ferroptosis of spinal dorsal horn neurons induced by SCIRI.^791^ These results suggest that EphA4 is involved in the regulation of ferroptosis in dorsal horn spinal motor neurons in SCIRI by promoting the formation of Beclin1-xCT complex and activating the ERK1/2/c-Myc/TFR1 pathway. Synovial protein 1 (SYVN1) serves as a promising prognostic marker for neurodegenerative diseases. Overexpression of SYVN1 inhibits ferroptosis in SCIRI rats and OGD/R-treated primary spinal neurons. Mechanistically, SYVN1 binds to HMGB1, promoting its ubiquitination and degradation and alleviating SCIRI in rats by downregulating HMGB1 and activating the Nrf2/HO-1 pathway.^792^ Despite these findings, the study of ferroptosis in SCIRI is still in its early stages, and many questions remain to be explored.

#### Ferroptosis in myocardial ischemia-reperfusion injury

Myocardial I/R injury after acute myocardial infarction and heart transplantation can lead to serious complications and an increased risk of death. Pathological changes caused by myocardial ischemia, such as intracellular acidification, glycolysis, and increased ROS production, can promote the lipid peroxidation process.^492^ Myocardiac I/R induces an increase in non-heme iron and increases transcription of FTL and FTH, indicating iron overload in ischemic myocardium.^490^ The ability of iron chelators or ferroptosis inhibitors to reverse cardiac damage during both acute and chronic I/R injury highlights ferroptosis as a promising new treatment strategy for myocardial I/R injury.^793,794^

Increased intracellular iron levels are a key factor promoting lipid peroxidation and ferroptosis in cardiomyocytes. circRNA sequencing reveals that circular RNA FEACR has a potential regulatory role in cardiomyocyte ferroptosis. FEACR directly binds to nicotinamide phosphoribosyl transferase (NAMPT) to enhance its stability and promotes the expression of NAMPT-dependent SIRT1.^795^ FEACR overexpression inhibits I/R-induced ferroptosis and myocardial infarction through SIRT1-forkhead box O1 (FOXO1)-FTH1 pathway, improving cardiac function.^795^ Therefore, FEACR and its downstream factors may be new targets for reducing ferroptosis in ischemic heart disease. Myocardial DNA (cytosine-5)-methyltransferase 1 (DNMT-1) and NCOA4 expression are increased in myocardial I/R injury rats, accompanied by elevated levels of ferroptosis.^796^ The DNMT-1 inhibitor 5-Aza-2′-deoxycytidine improves myocardial damage by reducing NCOA4-mediated ferritinophagy and reducing ferroptosis during I/R injury.^796^

Proteomic analysis based on tandem mass tag showed that GSH metabolic pathway was downregulated during myocardial ischemia, especially GPX4 in the early and middle stages of myocardial infarction. Using siRNA or chemical inhibitors to inhibit GPX4 leads to the accumulation of lipid peroxidation, causing ferroptosis in H9c2 cardiomyocytes.^797^ Even neonatal rat ventricular muscle cells with low sensitivity to GPX4 inhibition underwent ferroptosis in the presence of cysteine deprivation, suggesting that inhibition of GPX4 promotes ferroptosis of cardiomyocytes under metabolic stress, such as cysteine deprivation.^797^ Histochrome is a water-soluble form of echinochrome with strong antioxidant and iron-chelating effects.^798,799^ Intravenous injection of histochrome significantly reduces cardiac fibrosis, increases capillary density, and significantly improves cardiac function in I/R rats by upregulating the expression of Nrf2 and its downstream genes, including GPX4, and reducing intracellular and mitochondrial ROS levels.^800^

Clinical studies have shown that, compared with healthy volunteers, lysine-specific methyltransferase 2B (KMT2B), riboflavin kinase (RFK) and NOX2 are significantly upregulated in peripheral blood of patients with acute myocardial infarctionup.^801^ KMT2B promotes RFK transcription by upregulating H3 methylation levels, thus activating the TNF-α/NOX2 pathway, promoting ferroptosis and aggravating myocardial infarction size.^801^ In mice with I/R injury, methylmalonic acid promotes the expression of NOX2/4, increases ROS production in cardiomyocytes, aggravates myocardial oxidative stress and ferroptosis, and expands myocardial infarction size and cardiac dysfunction.^802^ Notably, GSH is released from the ischemic area into the extracellular space after I/R, accompanied by a decrease in intracellular GSH concentration. Many endogenous phospholipids increase significantly in the ischemic area, indicating the occurrence of ferroptosis. Pharmacological or gene inhibition of glutathione transporters, including multidrug resistant protein 1, can block cell GSH release, reduce intracellular ROS levels and production of oxidized phosphatidylcholine, thereby inhibiting cell ferroptosis.^803^

Ischemia triggers specific redox reaction of PUFA-PLs in ischemic myocardial cells, leading to strong oxidative damage in reperfusion stage. Oxidized PUFAs enriched phosphatidylethanolamines are proven to be key lipid species in I/R injury.^804^ ALOX15 specificity increases in the left ventricular injury area. Multi-omics results show that ALOX15 is the main mediator of phospholipid peroxidation, acting as an “ignition point” during the ischemia stage, igniting phospholipid oxidation and promoting cell ferroptosis.^804^

Lipid peroxide 4-HNE, which accumulates during myocardial I/R injury, promotes ubiquitination of GPX4 and induces cardiac ferroptosis by targeting the binding site of GPX4 and ovarian tumor deubiquitinase 5, while activation of aldehyde dehydrogenase 2 to degrade 4-HNE reduces myocardial ferroptosis.^805^ The transcription factor yes-associated protein (YAP) promotes the transcription of NEDD4L, leading to the ubiquitination and degradation of ACSL4, and reducing cardiac ferroptosis and myocardial infarction size in I/R mice.^806^

The current clinical treatment of myocardial I/R injury is limited, but antioxidants and active substances from Chinese medicine have shown great potential.^807^ Several animal experiments have demonstrated that antioxidants and active substances from Chinese medicine reduce myocardial I/R Injury and ferroptosis in ischemic cardiomyopathy animals, and protect myocardial function. Salidroside, a natural phenylpropanoid glycoside isolated from *Rhodiola rosea*, has been shown to enhance mitochondrial complex I activity by upregulating and phosphorylating AMPKα2, increasing cardiomyocyte tolerance to I/R injury and reducing ferroptosis.^808^ Consequently, salidroside may be a potential phytochemical candidate for treating myocardial I/R injury. In a mouse model with I/R injury, the combination of cyclosporine A (CsA) and DFX synergistically inhibits I/R-induced iron overload, lipid peroxidation, and cardiomyocyte ferroptosis, reducing infarct size and improving poor cardiac remodeling after I/R injury.^809^ CsA@ApoFn, which encapsulates CsA with apoferritin (ApoFn), enters cardiomyocytes through TFR1-mediated endocytosis.^810^ CsA@ApoFn reduces the content of unstable iron pool and lipid peroxide by increasing the expression of GPX4 protein, thereby inhibiting ferroptosis in ischemic cardiomyocytes,^810^ providing a promising strategy for the treatment of myocardial I/R injury. Fucoxanthin, a natural antioxidant carotenoid, regulates the expression of FTH, TFR1, and GPX4 by activating the Nrf2 signaling pathway, reducing I/R-induced cell ferroptosis and improving myocardial I/R-induced myofibrillar loss.^811^ In addition, other antioxidant, or herbal active substances, including resveratrol, gossypol acetic acid, galangin, kinsenoside, hydroxysafflor yellow A, baicalein, and luteolin, also improve myocardial I/ R injury by inhibiting ferroptosis.^812–817^ HUC-MSCs-derived exosomes directly downregulate the expression of DMT1 through enriched miR-23a-3p, thereby reducing Fe^2+^ and MDA levels in cardiomyocytes, inhibiting ferroptosis, and alleviating myocardial injury.^818^

#### Ferroptosis in renal ischemia-reperfusion injury

The kidney is extremely sensitive to I/R injury, and the mismatch between oxygen supply and oxygen demand leads to decreased oxidative metabolism, resulting in progressive damage of renal tubular epithelial cells. Renal I/R injury is widespread in clinical practice, commonly observed in conditions such as shock, trauma, renal transplantation, urology, and cardiovascular surgery, and can lead to a rapid decline in kidney function and increase patient mortality. The short- and long-term outlook for the patient depends on the reversibility of the injury and the recovery.^819^ Cell death induced by iron-dependent lipid peroxidation, known as ferroptosis, has significant deleterious effects in renal I/R injury models.^820,821^

N-acetyltransferase 10 (NAT10) is a newly identified RNA modifying enzyme that leads to the synthesis of N4-acetylcytidine (ac4C).^822,823^ Both ac4C RNA modification and NAT10 levels are increased in the kidney of the I/R injury group compared with the sham surgery group.^824^ Specifically knockout of NAT10 or inhibiting NAT10 activity in the kidney significantly inhibits ac4C RNA modification and reduces renal I/R injury.^824^ Mechanistically, NAT10 promotes the ac4C RNA modification of NCOA4 mRNA, which increases its stability and ferritinophagy, promoting the ferroptosis of renal tubular epithelial cells induced by I/R injury.^824^ The expression level of the stimulator of interferon genes (STING) in renal tubules increase after I/R treatment.^825^ STING mediates the initiation of ferritinophagy through its interaction with NCOA4, contributing to ferroptosis during ischemia, while STING knockout significantly alleviates I/R-induced lipid peroxidation, tissue damage, and renal dysfunction.^825^

Pannexin 1 (PANX1) is a protein involved in ATP release. Compared with wild-type mice, MDA levels and tubular cell mortality in renal tissue are decreased in Panx1 knockout mice that received renal I/R injury.^826^ Downregulation of Panx1 significantly reduces ferroptosis and iron accumulation in renal cells induced by erastin, through inducing HO-1 expression and the mitogen-activated protein kinase (MAPK)/ERK pathway.^826^ Analysis based on the GSE148420 dataset showed that HO-1 is a key biomarker and modulator of ferroptosis in renal I/R injury.^826^ Repressor element 1-silencing transcription factor (REST) is the main regulator of gene inhibition under hypoxia, and its expression level is positively correlated with the degree of kidney injury. REST directly binds to the promoter region of glutamate-cysteine ligase (GCLM), inhibits the synthesis of GSH through transcriptional inhibition of GCLM expression, and induces ferroptosis.^827^ This suggests that REST is involved in the transition from acute kidney injury to chronic kidney disease. Using spatial transcriptomics, GPX4 has been identified to be located at the interface between the inner cortex and the outer medulla of the kidney, which is the main site of cell ferroptosis after I/R injury.^828^ The GPX4-binding protein OTU deubiquitinase 5 (OTUD5) reduces the sensitivity of cells to ferroptosis by stabilizing GPX4. During I/R, mTOR1-mediated autophagy caused OTUD5 degradation and subsequent GPX4 decay, aggravating tubular cell ferroptosis and aggravating acute renal injury.^828^ OTUD5 overexpression alleviates ferroptosis and promote renal function recovery after I/R injury. MicroRNAs play an important role in I/R-induced acute kidney injury. The expressions of miR-378a-3p and miR-182-5p are upregulated in ferroptosis cells induced by renal I/R injury, and are negatively correlated with the expressions of GPX4 and SLC7A11. It was further found that miR-378a-3p and miR-182-5p negatively regulate the expression of SLC7A11 and GPX4 by directly binding to the 3’ untranslated region (3’UTR) of SLC7A11 and GPX4 mRNA, respectively.^829^

Bioinformatics analysis of the GEO database indicates that nuclear receptor subfamily 4 group A member 1 (NR4A1) may be a key molecule in inducing ferroptosis in renal tubular epithelial cells during renal I/R injury. NR4A1 inhibits the ubiquitination degradation of P53 by regulating the downstream target gene MDM2, thereby affecting the oxidative respiration process of mitochondria, producing oxidized lipids, and inducing ferroptosis in cells.^830^ The expression of ferroptosis-related genes is abnormal in renal tubular epithelial cells (TECs) after I/R injury, with the expression of ACSL4 is upregulated and correlating with renal function.^831^ XJB-5-131 is a mitochondria-targeting nitrogen oxide with dual antioxidant properties, containing mitochondria-targeting semi-gramicidin S and the free radical scavenger TEMPO. XJB-5-131 has a high affinity for TECs and reduces I/R-induced renal injury in mice by downregulating the expression of ACSL4 to inhibit ferroptosis.^832^ miR-20a-5p is significantly upregulated in kidney transplantation patients and mice with acute kidney injury. miR-20a-5p mimics reduce renal I/R injury and ischemic postrenal fibrosis, while miR-20a-5p inhibitors have the opposite effect. Importantly, miR-20a-5p inhibits ACSL4-dependent ferroptosis by targeting the 3’UTR of ACSL4 mRNA as a negative regulator of ACSL4.^833^ HMGB1 is a highly conserved nuclear protein. HMGB1 translocation from nucleus to the cytoplasm of renal tubular cells induces ferroptosis by binding ACSL4 after renal I/R injury, and inhibition the nuclear cytoplasmic translocation of HMGB1 could inhibit ferroptosis and renal I/R injury.^834^

Carnosine is a dipeptide composed of L-histidine and β-alanine that inhibits ferroptosis and reduces kidney damage. In hypoxia/reoxygenation (H/R)-induced human renal tubular epithelial cells, carnosine reduces iron accumulation and lipid peroxidation and inhibits ferroptosis. Results of cellular thermal shift assay and molecular docking indicate that GPX4 is a potential direct target of carnosine, and carnosine shows promise as a potential inhibitor of ferroptosis for the treatment of renal I/R injury and other conditions associated with ferroptosis.^835^ Mitoglitazone has a strong affinity for the mitochondrial outer membrane protein mitoNEET. By inhibiting lipid ROS generation and the hyperpolarization of mitochondrial membrane potential, mitoglitazone could restore mitochondrial DNA copy number, the generation of ATP, and mitochondrial morphology in kidney tissue, inhibiting mitochondrial dysfunction and ferroptosis induced by I/R. In addition, mitoglitazone significantly alleviates renal I/R injury in mice by upregulating GPX4 and reducing iron-related lipid peroxidation.^836^ Quercetin blocks the typical morphological changes of ferroptosis by decreasing MDA and lipid ROS levels and increasing GSH levels, thereby improving I/R-induced acute kidney injury. The mechanism may be related to inhibiting the activation of transcription factor 3 (ATF3) and increasing the levels of SLC7A11 and GPX4.^837^ Cyanidin-3-glucoside (C3G) is a flavonoid that has anti-inflammatory and antioxidant effects on I/R damage. In renal I/R injury, C3G increases GPX4 expression and GSH level, reverses excessive intracellular free iron accumulation, decreases lipid ROS, ACSL4, 4-HNE, and MDA levels, and significantly inhibits the ferroptosis of renal tubular cells.^838^ In addition, paeoniflorin, isoliquiritigenin, and curcumin have also been reported to alleviate I/R-induced acute kidney injury by inhibiting ferroptosis in renal tubule cells.^839–841^

There is growing evidence that stem cell-derived exosomes, which carry partial stem cell biomolecules, are a promising treatment for kidney disease.^842,843^ Human urine-derived stem cell-derived exosomes (USC-Exos) ameliorate renal I/R injury and ferroptosis. lncRNA TUG1 in USC-Exos regulates the stability of ACSL4 mRNA by interacting with the RNA-binding protein SRSF1, serving as a promising therapeutic method for renal I/R injury.^844^

#### Ferroptosis in intestinal ischemia/reperfusion injury

Intestinal ischemia/reperfusion (I/R) injury occurs in many clinical conditions, such as acute mesenteric ischemia, small intestine torsion, and trauma, and is a life-threatening vascular emergency.^845^ Transcriptomic analyses of patients with intestinal I/R injury and mice showed that ferroptosis-related genes such as IL-6, CXCL2, HMOX1, GDF15, HSPA5, and TNFAIP3, may be hub genes in intestinal I/R injury.^846^ Ferroptosis is present in the intestine during intestinal ischemia, along with increased expression of ACSL4 and decreased levels of FTH1 and GPX4, and inhibition of ACSL4 has protective effects on ferroptosis. The transcription factor special protein 1 (Sp1) enhances the transcription of ACSL4 by binding to its promoter region, playing a role in intestinal I/R injury.^847^ In mice with intestinal I/R injury, there is a significant upregulation of ACSL4, 15-LOX and MDA, which are key markers in intestinal epithelial cells. Additionally, there is an increase in NCOA4 and autophagy-related proteins such as Beclin-1 and LC3, along with elevated levels of Fe^2+^.^848^ These suggest that NCOA4 may contribute to intestinal I/R injury in mice by inducing ferroptosis through mediating ferritinophagy.

Intestinal I/R causes disruptions in intestinal flora and significant alterations in metabolites. The level of metabolite capsiate (CAT) in the gut microbiota has been found to have a negative correlation with intestinal I/R damage.^849^ CAT improves intestinal I/R injury by activating the transient receptor potential cationic channel subfamily V member 1 (TRPV1), enhancing GPX4 expression, and inhibiting ferroptosis.^849^ In animal models of intestinal I/R, ferroptosis occurs primarily during the reperfusion phase due to the inactivation of the GSH/GPX4 pathway.^848^ Resveratrol may improve I/R injury by activating SIRT3/FOXO3a pathway, which increases the expression of catalase and SOD2 and inhibits the production of ROS, thereby reducing lipid peroxidation and ferroptosis.^850^ The metabolic regulator sestrin 2 alleviates ferroptosis caused by intestinal I/R injury by activating the Keap1/Nrf2 signaling pathway.^851^

Intestinal I/R causes dysfunction of intestinal microcirculation and aggravates intestinal injury. Increased infiltration of local neutrophil extracellular traps (NETs) around intestinal microvasculature, accompanied by increased ferroptosis of endothelial cells, was detected in both intestinal I/R patients and animal models, which may be a major cause of microcirculation dysfunction.^852^ RNA-seq analysis showed significant enrichment of signaling pathways related to mitochondrial autophagy and ferroptosis in human umbilical vein endothelial cells (HUVECs) incubated with NETs. NETs induce Fundc1 phosphorylation of endothelial cells, inhibit mitochondrial autophagy and lead to mitochondrial ROS overproduction and lipid peroxidation, thus inducing endothelial cell ferroptosis and microvascular dysfunction.^852^

Intestinal I/R can also cause acute lung injury, in which ferroptosis plays an important role. Isoiquiritin apioside, an important component of *Glycyrrhizae radix et rhizoma*, could inhibit the upregulation of HIF-1α and HO-1 protein in lung tissue, downregulate the levels of PTGS2 and ACSL4, inhibit the ferroptosis in lung tissue of mice, and prevent intestinal I/ R-induced lung injury.^853^ Activating the Nrf2 pathway to negatively regulates ferroptosis may also be a possible future strategy for treating lung injury caused by intestinal I/R injury.

### Ferroptosis in musculoskeletal diseases

**Fig. 6:**
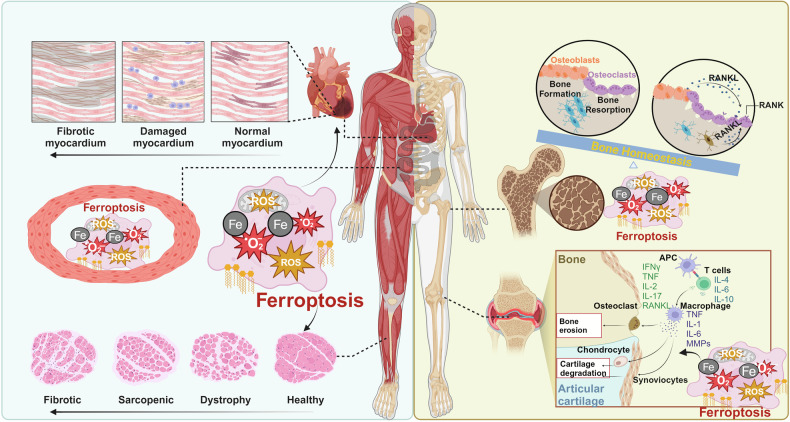
Ferroptosis in musculoskeletal diseases. Ferroptosis in the musculoskeletal system contributes to the occurrence and development of arthritis primarily through lipid peroxidation and oxidative stress, which to damage the joint synovial membrane, matrix, and hyperactivated immune cells. Additionally, ferroptosis disrupts the delicate balance of osteoblasts and osteoclasts in the bone tissue, leading to the development of osteoporosis. Ferroptosis also plays a crucial role in the decline of quality and function in skeletal muscle, cardiac muscle and smooth muscle, there by inducing the onset of sarcopenia. This figure was created with BioRender (https://biorender.com/)

Musculoskeletal diseases, such as osteoarthritis, osteoporosis, sarcopenia, and amyotrophic lateral sclerosis, seriously impair patients’ quality of life and place a substantial strain on global public health resources. Emerging research indicates a strong link between ferroptosis and the occurrence and progression of these musculoskeletal diseases, suggesting that targeting ferroptosis could offer a novel therapeutic approach for these diseases (Fig. 6).

#### Ferroptosis in osteoarthritis

Osteoarthritis (OA) is a debilitating joint disease that has become a significant health issue in the elderly population. OA is characterized by swelling and pain in the affected joints and is often associated with limited mobility and disability.^854^ Changes associated with ferroptosis in the cartilage of the injured articular area in OA patients, including GPX4 downregulation, ACSL4 upregulation, MDA accumulation, and mitochondrial morphological and function changes, are more severe than in the intact articular area and worsen with OA progression.^855^ Analysis of the datasets GSE51588 and GSE55457 revealed that phosphogluconate dehydrogenase (PGD) and LPCAT3 may serve as diagnostic biomarkers for OA and could aid in its diagnosis and treatment.^856^ Multiple studies have demonstrated that ferroptosis occurs in damaged synovial areas in OA patients, who have higher lipid peroxidation levels than healthy individuals.^857,858^ Single-cell RNA sequencing analysis revealed a new subgroup of inflammatory chondrocytes with regulatory potential in the microenvironment of hand osteoarthritis.^859^ The proportion of inflammatory chondrocytes and fibrocartilage chondrocytes increases in osteoarthritic cartilage, with ferroptosis pathways enriched in these two subgroups of osteoarthritis cartilage.^859^ High serum ferritin levels are significantly associated with the prevalence of hand osteoarthritis, suggesting that inflammation and ferroptosis in fibrocartilage chondrocytes may be key pathways of osteoarthritis.^859^

Compared to healthy individuals, the level of iron in synovial fluid of OA patients is significantly higher and positively correlated with the severity of OA. The contents of Fe^2+^, Fe^3+^ and total iron in the cartilage with OA injury are significantly higher than those in the cartilage without OA injury, suggesting iron deposition occurs in the cartilage during OA progression. Serum ferritin level is positively linked to the severity of cartilage injury in knee OA patients, indicating that serum ferritin may actively participate in cartilage injury in knee arthritis.^860^ Nutrition studies have found a U-shaped association between iron intake and OA progression in the knee joint, with appropriate iron intake helping to prevent OA progression, while excess or insufficient iron intake increases the risk of OA progression.^861^ Menopause is a risk factor for OA, and epidemiological studies have found that postmenopausal women have a rapidly increased risk of osteoarthritis compared to premenopausal women of the same age, indicating that estrogen level is a key factor in OA development.^862^ G protein-coupled receptor 30 (GPR30) is an estrogen receptor whose expression in OA cartilage is lower than that in normal tissue. The GPR30 receptor agonist G1 inhibits ferroptosis and significantly improves chondrocyte viability and motor ability in mice through the YAP1/FTH1 pathway, while GPR30 receptor antagonist G15 has the opposite effect, suggesting that hormone-related ferroptosis may emerge as a promising target for alleviating osteoarthritis in postmenopausal women.^863^ NCOA4 mediated ferritinophagy plays a crucial role in regulating intracellular iron levels, and elevated expression of NCOA4 has been observed in the cartilage of OA patients, OA mice, and inflammatory chondrocytes. During the pathogenesis of OA, JUN directly binds to the promoter of NCOA4 and initializes its transcription, increasing ferritinophagy degradation, leading to ferroptosis and extracellular matrix degradation in chondrocytes.^864^

Downregulation of the antioxidant defense system has been noted in OA patients, evidenced by decreased levels of GSH and its oxidized form GSSG in their synovial fluid.^865^ Regulation of the Nrf2 antioxidant system improves IL-1β or FAC-induced ferroptosis in osteoarthritis-like chondrocytes by affecting GPX4 and SLC7A11 expression.^866^ Excitatory amino acid transporter 1(EAAT1) in senescent cartilage membranes can increase intracellular glutamate levels and activate the GSH synthesis system to combat ferroptosis.^867^ HSPA5, member A of the heat shock protein family, directly binds to GPX4 and positively regulates its expression. The RNA-binding protein SND1 binds to the 3’UTR of HSPA5, destabilizing HSPA5 mRNA, thereby inhibiting GPX4 and promoting ferroptosis in osteoarthritis.^868^ P21 significantly affects the recruitment of GPX4 to the linear ubiquitin chain assembly complex and plays an important role in anti-ferroptosis in OA by regulating the stability of GPX4.^869^ FOXO3 attenuates OA progression by promoting the SLC7A11/GPX4 pathway and inhibiting the NF-κB/MAPK pathway, thus inhibiting ferroptosis and extracellular matrix degradation in chondrocytes.^870^

Exposure of chondrocyte to excessive mechanical load leads to increased catabolism. Iron overload induces ferroptosis by activating Piezo1, a pressure-sensitive calcium channel in chondrocytes, leading to subsequent calcium inflow, which could be exacerbated by conditional GPX4 knockout in cartilage. Ferroptosis of chondrocytes caused by high strain mechanical stress can be eliminated by blocking calcium influx in calcium-free medium.^871^

Oxidative stress is characterized by MDA and 4-HNE, and their roles in cartilage degeneration and subchondral bone remodeling during OA development have received extensive attention.^872^ Grigolo et al. found that MDA and 4-HNE levels were increased in the synovial cells of OA patients compared to healthy controls, and 4-HNE levels were also increased in synovial fluid of OA patients.^873^ The expression of ACSL4 and 4-HNE increase in the cartilage of OA patients and OA mice, while metformin increases p-AMPK and p-ACC levels in IL-1β-treated chondrocytes, reverses ACSL4 and 4-HNE positive chondrocytes and OA lesions, and improves the ferroptosis sensitivity of chondrocytes through the AMPK/ACC pathway.^874^ The effect of metformin on OA is also related to improving the microstructure of subchondral osteosclerosis and reducing heterologous angiogenesis.^875^ It is important to note that not all lipids contribute to chondrocyte ferroptosis in OA pathogenesis. Lipoxin A4 is a potent anti-inflammatory lipid mediator that acts as an “inflammatory shutdown signal” to inhibit inflammatory processes. Lipoxin A4 upregulates the expressions of GPX4, lysophosphatidic acid receptor-3, and estrogen receptor beta in FLSs, decreases the expression of MMP13 and MMP3, improves the pain performance of OA rats, and alleviates the synovial and cartilage lesions of OA rats by inhibiting ferroptosis.^876^

The nitrogen oxide XJB-5-131 is an antioxidant that targets mitochondria. XJB-5-131 significantly inhibits the increase of tert-butyl hydroperoxide-induced ferroptosis-driving factors (PTGS2, TFR1, and ATF3) and the accumulation of ferroptosis markers (Fe^2+^, ROS, and lipid peroxide) in chondrocytes. Meanwhile, the expression of ferroptosis inhibitors such as GPX4 and FTH1 is increased, promoting cartilage anabolism, and providing a protective effect on chondrocytes.^877^ Traditional Chinese medicine Jianpi-Tongluo Formula (JTF) effectively reduces joint edema and pain in OA rats, inhibits extracellular matrix degradation, and may work by inhibition of the NCOA4-HMGB1-GSK3β-AQPs axis. JTF may protect cartilage by inhibiting ferroptosis and aquaporin dysregulation driven by NCOA4-HMGB1.^878^ Other active substances in Chinese medicine, such as ruscogenin, curcumin, baicalein, brevilin A, icariin, and cardamonin could also inhibit chondrocyte ferroptosis, reduce cartilage destruction in osteoarthritis.^879–885^

Exosomes from mesenchymal stem cells (MSC-Exos) regulate the inflammatory response and microenvironment, promoting the renewal of injured tissues, including cartilage and subchondral bone. MSC-Exos activate Nrf2/HO-1 expression via the GOT1/CCR2 signaling pathway to prevent ferroptosis, reduce inflammation in OA mice, and improve their performance, providing a potential treatment for OA and other orthopedic diseases.^886^ The intestinal microbiota metabolite capsiate is negatively correlated with cartilage degeneration score in OA mice, and capsiate reduces ferroptosis dependent osteoarthritis both in vivo and in vitro, through activation of SLC2A1 and inhibition of HIF-1α.^887^ D-mannose also protects cartilage by inhibiting HIF-2α and reducing the sensitivity of chondrocytes to ferroptosis.^888^

#### Ferroptosis in osteoporosis

Osteoporosis is an age-related disease with a rising incidence in the elderly population, increasing the risk of fractures. The maintenance of bone tissue integrity and homeostasis necessitates a balance between osteoblasts, which form bone, and osteoclasts, which resorb bone. Excessive iron levels can generate produce ROS via the Fenton reaction, activating various intracellular signaling pathways that enhance bone absorption and inhibit bone formation, thereby contributing to osteoporosis.^61^

Bone remodeling is a continuous and periodic process, with osteoblasts playing a pivotal role in bone regeneration. Bone marrow mesenchymal stem cells (BMSCs) can differentiate into various cell types, including osteoblasts and chondrocytes. Iron overload increases ferritin levels and decreases RUNX2 levels in BMSCs, causing irregular morphological changes, significantly reducing their activity, and inhibiting osteogenic differentiation, while melatonin therapy could reverse this process and promote osteogenic differentiation of BMSCs.^889^ Inhibition of the Wnt signal hinders osteogenic differentiation by affecting the Smad and MAPK signaling pathways. Iron dose-dependently downregulates the expression of proteins in the Wnt pathway and suppresses of Wnt reporter gene TopFlash transcription. Wnt agonists or ferroptosis inhibitors can reverse the typical Wnt signaling inhibition induced by iron overload and restore osteoblast differentiation by reducing ROS and LPO production.^890^ Iron-dependent lipid peroxidation contributes to the development of diabetes-related osteoporosis.^891,892^ Overexpression of FtMt reduces ferroptosis in osteoblasts induced by high glucose, while silence FtMt induces mitochondrial autophagy through the ROS/PINK1/Parkin pathway, thereby inducing ferroptosis in osteoblasts.^893^ This may be one of the important mechanisms of diabetes-induced osteoporosis. Increased advanced glycosylation end products in a high-glucose environment also affects osteoblast differentiation and function.^894^ Acid sphingomyelinase (ASM)-mediated autophagy activation is critical for hyperglycemia -induced GPX4 degradation, and inhibition of ASM improves osteogenic function by reducing hyperglycemia-induced GPX4 degradation and subsequent ferroptosis.^895^

In D-galactose-induced aging mice, the vitamin D receptor activator 1,25-(OH)_2_-D_3_ alleviates D-galactose-induced osteoblast ferroptosis, manifested by improved mitochondrial morphology and decreased lipid peroxidation markers, and the mechanism may be related to the activation of the Nrf2/GPX4 signaling pathway to reduce osteoblast ferroptosis.^896^ Eldecalcitol (ED-71), a novel active vitamin D, also attenuates hyperglycemia-induced HIF1-α overexpression and prevents osteoblast ferroptosis.^897^ Fat-soluble vitamin K2 (VK2) was also found to inhibit high glucose-mediated bone loss and ferroptosis, restore bone mass, and enhance the expression of osteogenic markers in the distal femur.^898^

High doses of dexamethasone induce steroid-induced osteoporosis by downregulating system Xc^−^ and GPX4 and inducing ferroptosis in osteoblasts. Extracellular vesicles from endothelial progenitor cells reverse dexamethasone-induced GSH dyssynthesis and changes in several markers of oxidative damage, preventing glucocorticoid-induced osteoporosis in mice by inhibiting ferroptosis of osteoblasts.^899^ The bone mineral density of the femoral neck in smokers is significantly lower than that in non-smokers.^900^ Clinical investigation confirms that smoking-induced bone homeostasis disruption is an independent risk factor for osteoporosis.^900^ Transcriptional analyses show that the ferroptosis pathway is significantly enriched in rat BMSCs exposed to cigarette smoke extract. Intracellular ROS accumulation increases unstable iron and lipid peroxidation deposition through AMPK/NCOA4-mediated ferritinophagy, ultimately leading to ferroptosis and dysfunction in rat BMSCs. NOX4 was found to be elevated in the bones of osteoporosis patients and mouse models.^901^ The DNA sequence of NOX4 contains iron-responsive element-like (IRE-like) sequences that typically bind to iron-regulatory protein 1 (IRP1). Iron overload induces the separation of IRP1 from IRE-like sequence, activating NOX4 transcription, increasing lipid peroxidation accumulation, and leading to significant dysregulation of mitochondrial morphology and function of osteoblasts.^901^

Osteoclasts are giant, multinucleated cells that arise from the fusion of monocytes/macrophage progenitor cells differentiated from myeloid progenitors in bone marrow. The formation of osteoclasts is regulated by M-CSF and receptor activator of nuclear factor kappa-Β ligand (RANKL). Nrf2 mediates the DNA methylation level of RANKL promoter by regulating the activity of DNA methyltransferase 3a, participating in the regulation of ferroptosis in osteoclasts and bone homeostasis.^902^ Due to the increased demand for iron in osteoclast development, TFR1-mediated iron uptake promotes osteoclast differentiation and bone resorption activity.^903^ Iron-chelating agents inhibit osteoclast bone resorption and prevent bone loss due to ovariectomy.^903^ In a RANKL-induced osteoclast model, the bisphosphonate zoledronic acid promotes ferroptosis by increasing Fe^2+^, ROS, and MDA levels, and decreasing GPX4 and GSH levels.^904^ During the differentiation of osteoclasts induced by RANKL, RANKL stimulates ferritinophagy to regulate intracellular iron homeostasis. However, under hypoxic conditions, HIF-1α inhibits ferritinophagy flux and protects osteoclasts from ferroptosis by inhibiting the formation of autophagosomes.^905^ Inducing osteoclast ferroptosis by targeting HIF-1α and ferritin may be an alternative approach to treating osteoporosis.

Single-cell transcriptome analysis identified transcription factor 3 (ATF3) as a key driver of ferroptosis in osteocytes. Elevated ATF3 expression in aging osteocytes promotes iron uptake by upregulating TFR1 while inhibiting SLC7A11-mediated cystine uptake, leading to iron overload and lipid peroxidation, ultimately resulting in ferroptosis.^906^ Osteocyte ferroptosis in a high-glucose microenvironment has also been shown to be associated with diabetic osteoporosis. RNA sequencing results showed that HO-1 expression is significantly upregulated in siderophile osteocytes. High glucoses levels cause increased ferroptosis in osteocytes, manifested by iron overload, abnormal activation of the ferroptosis pathway, and increased lipid peroxidation products.^907^ Breaking the vicious cycle between lipid peroxidation and HO-1 activation effectively reverses osteocyte ferroptosis of in diabetic osteoporosis and ultimately improves trabecular deterioration.^907^

Ten weeks of treadmill exercise promote the expression and release of irisin, activate the Cav1/AMPKα/Nrf2 pathway, and increase the transcription of HO-1 and FPN, thereby promoting osteoblast proliferation. Myotube-derived exosomes participate in the transport of irisin and enter osteoblasts through vesicle-mediated endocytosis, which may serve as a novel mechanism for exercise to ameliorate osteoporosis.^908^ Natural active substances are widely used in the prevention and treatment of osteoporosis. Mangiferin is a xanthine glycoside isolated from mango. Mangiferin directly binds to Keap1, activates the downstream Nrf2/SLC7A11/GPX4 pathway, inhibits osteoblast ferroptosis, promotes bone formation and improves osteoporosis in ovariectomized mice and iron overload mice.^909^ Poliumoside, extracted from *Callicarpa kwangtungensis Chun*, inhibits bone degradation and ferroptosis induced by a high-glucose and high-fat diet through the Nrf2/GPX4 pathway, increases femoral bone mineral density, and prevents osteoporosis associated with type 2 diabetes.^910^ Natural products such as quercetin, artemisinin, and biochanin A have been shown to play roles in the treatment of osteoporosis by promoting osteoclast ferroptosis and inhibiting osteoblast ferroptosis.^911^

#### Ferroptosis in sarcopenia

The main clinical features of sarcopenia are reduced skeletal muscle mass and decreased muscle function. Primary sarcopenia is mainly age-related, while secondary sarcopenia arises from conditions such as heart failure, kidney failure, malignancy, and chronic obstructive pulmonary disease.^912^ Extensive research indicates that iron accumulation and decreased endogenous antioxidant defenses contribute significantly to the progression of sarcopenia.^913,914^ Non-heme iron content in muscle increases with age, and age-related or disuse-induced muscle atrophy are associated with disrupted iron homeostasis and elevated oxidative stress.^915,916^ Investigations have also demonstrated that muscle atrophy caused by iron overload is connected to the ROS-mediated activation of the ubiquitin-proteasome system.^917^

Ferroptosis of myocytes induced by lipid peroxidation accumulation and iron overload through the p53/SLC7A11 pathway is crucial in sarcopenia’s pathogenesis and can be used as a potential intervention target for intervention.^377^ Iron overload reduces the phosphorylation of FOXO3a and AKT in skeletal muscle, and increases the expression of muscle atrophy-associated E3 ubiquitin ligase muscle ring finger-1 and atrogin-1.^918^ Inhibition of FOXO3a or oxidative stress reverses iron overload-induced muscle atrophy by reactivating the AKT-FOXO3a pathway.^918^

Satellite cells, which are stem cells in skeletal muscle with myogenic differentiation potential, play a vital role in muscle regeneration. Specifically knocking out TFR1 in satellite cells impairs muscle regeneration by activating ferroptosis. In addition, macrophages play an important role in muscle regeneration. Macrophages express CD163, ferritin and HO-1, indicating that they can uptake heme and store iron. Transferrin-mediated iron uptake in regenerated muscle fibers is independent of systemic iron homeostasis, and the iron cycling pathway between macrophages and myoblasts in the injured microenvironment affects the differentiation of myocyte precursors. Macrophage-mediated iron release can promote muscle regeneration, while selective inhibition of macrophage iron output can inhibit muscle regeneration and cause fat accumulation.^919^ The latest bioinformatics analysis identified 11 key genes associated with sarcopenia, including FOXO1, HSPB1, MDH2, CDKN1A, and DLD, which are closely related to ferroptosis in sarcopenia.^920^ In addition, differences in immune cell infiltration were observed between normal and sarcopenia samples. These differences can be used as intervention targets in future studies.

## Potential therapeutic targets and strategies for the treatment of human diseases

Given the important role of ferroptosis in the occurrence and progression of many diseases, targeting ferroptosis may be a promising direction for clinical treatment. Screening promising therapeutic targets related to ferroptosis and potential therapeutic strategies for clinical application has become necessary and urgent. Below, we summarize novel therapeutic targets and strategies with clinical application potential to provide more options for disease treatment (Table 1).

**Table 1:** Recent advances in preclinical research for treating human diseases by targeting ferroptosis

Classification	Items	Diseases or condition	Mechanisms	Ref(s)
Ferroptosis detection	Lipid droplet	Myocardial I/R injury	Lipid droplets are organelles that store neutral lipids in cells, and the balance between degradation and storage of lipid droplets affects the cell’s sensitivity to ferroptosis. TPABTBP is an aggregation-induced emission probe with high lipid droplet specificity and light stability, suitable for imaging dynamic changes of lipid droplets during ferroptosis.	996
	Mitochondrial viscosity	Myocardial I/R injury	Viscosity influences protein-protein interactions in mitochondrial membranes and is associated with various diseases. Mitochondrial viscosity increases during ferroptosis. The fluorescence probe PPAC-C4 is used for ultra-precision quantification of mitochondrial viscosity by attaching mitochondrial-targeting cation fragments to a vibration-based fluorescent scaffold. The probe Mito-3, containing a cationic quinoline unit and a C12 chain, can be used to locate and monitor changes in intracellular mitochondrial viscosity at close range by near infrared fluorescence. The probe CBS, based on the docking of electrostatic force and prop-protein molecules, achieves stable and accurate detection of mitochondrial viscosity.	997–999
	Hydrogen polysulfides (H_2_Sn) and sulfur dioxide (SO_2_)	Myocardial I/R injury	Hydrogen polysulfide (H_2_S_n_) and sulfur dioxide (SO_2_), metabolites of cysteine and glutathione, are closely related to the occurrence of ferroptosis. The nanoprobe UCNP@mSiO2@SP-NP-NAP, which wraps a photoreactive dye (SP-NP-NAP) onto nanoparticles, is activated by visible and near-infrared light to detect H_2_S_n_ and SO_2_ in ferroptotic cells.	1000
	Transferrin receptor1 (TFR1)	Myocardial I/R injury	TFR1 on the surface of cell membranes can bind to transferrin in the blood and promote iron uptake, making TFR1 a promising biomarker for ferroptosis. A multimodal imaging platform based on superparamagnetic cubic iron oxide nanoparticles was used to specifically detect intracellular TFR1 levels using a probe SCIO-ICG-CRT-CPPs NPs. CPPs enable the probe to enter tissues, CRT binds to TFR1 via a non-classical ligand guidance mechanism, ICG has optical imaging properties, and SCIO NPs possess MPI/MRI imaging properties.	1001
Ferroptosis therapeutic targets	Transient receptor potential mucolipin 1 (TRPML1)	Cancer	In AKT-hyperactivated cancer cells, AKT directly phosphorylates the Ser343 site of TRPML1, inhibits the K552 ubiquitination degradation of TRPML1, and promotes ferroptosis, enhancing the sensitivity of radiotherapy and immunotherapy.	922
	Fatty acid desaturases 1 and 2 (FADS1/2)	Triple-negative breast cancer	FADS1/2 controls lipid metabolism and susceptibility to ferroptosis of triple-negative breast cancer cells and may serve as a potential target for cancer therapy.	923
	Migration inhibitory factor (MIF)	Cancer	MIF promotes DNA homologous repair by activating the breast cancer type 1 susceptibility protein, thereby leading to resistance to ferroptosis in cancer cells.	925
	Histone deacetylase 3 (HDAC3)	Liver injury	Inhibition of HDAC3 leads to increased nuclear translocation of YAP through the Hippo pathway and participates in iron overloading induced ferroptosis in liver injury by altering hepcidin levels.	927
	Histone deacetylase 1/2 (HDAC1/2)	Intracerebral hemorrhage	Inhibition of HDAC1/2 reduces nerve ferroptosis by altering microglial heterogeneity, and the mechanism is related to the Nrf2/HO1 pathway.	928
	Transient receptor potential melastatin 2 (TRPM2)	Hepatic I/R injury	TRPM2 induces mitochondrial lipid peroxidation to participate in hepatic I/R injury by increasing the expression of ALOX12.	930
	Mg^2+^/Mn^2+^ dependent 1K (PPM1K)	Cerebral I/R injury	Branched chain amino acids (BCAAs) induce significant changes in genes and proteins associated with ferroptosis in neurons and increase lipid ROS levels. PPM1K inhibits the phosphorylation of the E1α subunit of BCAAs dehydrogenase, promotes the degradation of BCAAs, and thus inhibits cell ferroptosis.	933
	Elabela-apelin receptor axis	Ischemic stroke	After binding to the receptor APJ, elabela activates the Nrf2/ARE antioxidant signaling pathway, downregulates expression of ACSL4 and ALOX15, upregulates of GPX4 and xCT, and reduces ferroptosis.	935
	lncRNA SEMA5A-IT1	Myocardial I/R injury	After uptake by cardiomyocytes, lncRNA SEMA5A-IT1 in serum extracellular vesicles regulates the expression of BCL2 and SLC7A11 through sponge miR-143-3p, thereby inhibiting ferroptosis.	937
	LncRNA WAC-AS1	Renal I/R injury	The transcription factors BACH2 inhibits the transcription of GPX4 and SLC7A11. Once ferroptosis occurs in renal tubular epithelial cells, lncRNA VEC-AS1 in exosomes secreted by injured cells induces ferroptosis in neighboring cells by upregulating the expression of D-fructose-6-phosphate amidotransferase 1 and inhibiting the ubiquitination degradation of BACH2. Ferroptosis can spread widely in the renal tissue microenvironment in a “wave shape”, forming a “ferroptosis wave”.	936
Ferroptosis therapeutic strategies	GalNAc ligand-coupled TMPRSS6-siRNA (SLN124)	β-thalassemia	Hepcidin is negatively regulated by TMPRSS6. Trimeric GalNAc ligand-coupled TMPRSS6-siRNA (SLN124) can achieve effective and targeted delivery of oligonucleotides to the liver, regulate the expression of hepcidin, and effectively reduce liver iron overload.	965
	Triphenylphosphine modified quercetin derived smart nanoparticle (TQCN)	Alzheimer’s disease	TQCN can effectively chelate iron through quercetin mediated spontaneous coordination and self-assemble metal phenol nanocomplexes in situ to reduce iron overload and its induced free radical burst, thereby improving ferroptosis in neurons. In addition, TQCN activates the Nrf2 endogenous defense system.	940
	TPA@Laponite hydrogel	Spinal cord injury	The hydrogel TPA@Laponite has shear-thinning properties and can transition from a gel state to a flowing state when subjected to shearing forces. TPA@Laponite has a strong scavenging effect on ROS, and can inhibit ferroptosis by regulating iron metabolism. Introducing dental pulp stem cells into TPA@Laponite hydrogel can effectively reduce muscle spasm and promote the recovery of spinal cord injury by regulating the ratio of excitatory and inhibitory synapses.	966
	mPEG-b-Lys-BECI-TCO	Spinal cord injury	Mesenchymal stem cells (MSCs) are coupled with fer-1 to form nanoparticles mPEG-b-Lys-BECI-TCO. MSCs-mediated mitochondrial transfer can restore neuronal mitochondrial pools, and MSCs-Fer combined therapy can inhibit ferroptosis and improve the inflammatory microenvironment after spinal cord injury.	952
	Idebenone micelles	Cerebral I/R injury	Idebenone is structurally similar to coenzyme Q10 and has good antioxidant activity. CREKA peptide-modified idebenone cross-linked micelles accumulate in ischemic brain tissue by binding to microthrombus in ipsilateral microvessels. Under ROS stimulation, the diselenide bound in the micelles is converted into hydrophilic selenite, thus achieving the dual role of ROS consumption and idebenone release, thereby preventing the ferroptosis of neurons.	969
	pH/GSH-supported polyamino acid nanogel (NG/EDA)	Cerebral I/R injury	NG/EDA can penetrate the blood-brain barrier and effectively accumulate at the site of cerebral ischemic injury in rats. The acidic and high-glutathione microenvironment triggers NG/EDA, enabling the selective and sustained release of edaravone and inhibiting ferroptosis.	943
	Liproxstatin-1@GluAC4A	Ischemic stroke	The coupling of GluAC4A with liproxstatin-1 (liproxstatin-1@GluAC4A) significantly improves the solubility and release of liproxstatin-1 at hypoxic sites, and significantly reduces ferroptosis induced by rtPA treatment.	971
	M2pep-ADSC-Exos	Ischemic stroke	Stem cell-derived adipocyte exosomes with targeting specificity to M2 microglia are known as M2pep-ADSC-Exos. Fxr2 in M2pep-ADSC-Exos regulates the expression of ATF3 and SLC7A11, effectively reducing the susceptibility of M2 microglia to ferroptosis.	972
	N-Cu5.4O@DFO NPs	Renal I/R injury	Neutrophil cell membrane coated with copper-based nanoparticles (N-Cu5.4O@DFO NPs) is highly biocompatible and stable, capable of removing excess iron, reducing oxidative damage, and inhibiting ferroptosis.	945
	PEG-PDA@rutin NPs (PPR NPs)	Renal I/R injury	Rutin-loaded polydopamine nanoparticles PEG-PDA@rutin NPs (PPR NPs) have ROS-accelerated rutin release and strong ROS clearance ability. PPR NPs can effectively enter cells, reduce Fe^2+^ deposition and lipid peroxidation, repair mitochondrial damage, and inhibit ferroptosis.	949
	PDN@AGL	Intestine I/R injury	Amphiphilic molecules DTPA-N10-10 and mPEG-TK-DA self-assemble with apigenin-7-O-Glucoside (AGL) through hydrophilic interaction to form multi-site ROS clearing nanoparticles called PDN@AGL. PDN@AGL inhibits ferroptosis by reducing ROS levels and lipid peroxidation and regulating the ATF3/SLC7A11 pathway.	954
	Polyphenol-based nanomedics (ES NDs)	Osteoarthritis	Polyphenol-based nanomedicines (ESNDs) are synthesized through Mannich condensation reaction between selenometine and epigallocatechin-3-gallate. These nanomedicines effectively reduce the abnormal accumulation of Fe^2+^, GPX4 inactivation, and lipid peroxidation in chondrocytes, thereby improving the metabolic disorders of chondrocytes caused by ferroptosis.	956
	Cit-AuNRs@Anti-TRPV1	Osteoarthritis	Citrate stabilized gold nanorods coupled with TRPV1 monoclonal antibody Cit-AuNRs@Anti-TRPV1 can be used as a photothermal switch to activate transient receptor potential vanillate-like protein 1 (TRPV1) in chondrocytes under near-infrared irradiation. Intraarticular injection Cit-AuNRs@Anti-TRPV1 upregulates GPX4 by activating TRPV1, thereby inhibiting chondrocyte ferroptosis.	958
	tFNA-cur	Diabetic osteoporosis	Tetrahedral framework nucleic acid (tFNA) encapsulates curcumin to synthesize tFNA-cur, which can transport curcumin to bone marrow, activate the Nrf2/GPX4 pathway, enhance mitochondrial function, and inhibit ferroptosis of bone marrow mesenchymal stem cells.	959

### Potential therapeutic targets for treating of human diseases

Novel targets represent breakthroughs in the discovery of new drugs and treatment strategies, marking the search for disease targets a focus of intense research competition. Currently, 264 ferroptosis-driving targets and 238 ferroptosis-inhibition targets have been identified. NCOA4, which regulates ferritinophagy; NOX4 and Keap1, which regulate the redox state of cells; and ACSL4, ALOX12 and ALOX15, which are involved in lipid oxidation, are all known targets for driving ferroptosis. FTH1, which is involved in iron storage; SLC7A11, which regulates cystine intake; GPX4 and FSP1, which scavenge free radicals; and Nrf2, which promotes the transcription of these molecules, are all known ferroptosis suppressor targets. As research on ferroptosis deepens and expands, more potential therapeutic targets have been discovered that can regulate ferroptosis based on the classical ferroptosis pathway. This points to new directions for drug development and clinical treatment strategies. Although there are currently no corresponding agonists or inhibitors for these potential targets, functional studies of these potential biomolecules using existing techniques at the gene, protein, and cellular levels are crucial for identifying drug candidates and delaying or reversing disease progression.

#### Transient receptor potential mucolipin 1 (TRPML1)

Lysosomal exocytosis is crucial for maintaining lysosomal homeostasis.^921^ Recent studies have found that lysosomal exocytosis reduces lipid peroxidation by decreasing intracellular Fe^2+^, enhances plasma membrane repair and inhibits cell ferroptosis.^922^ Zhang et al. identified TRPML1-mediated lysosomal exocytosis as a potential anti-ferroptosis process.^922^ In AKT-hyperactivated cancer cells, AKT directly phosphorylates the Ser343 site of TRPML1, inhibits the K552 ubiquitination degradation of TRPML1, and promotes the binding of TRPML1 to ARL8B, leading to lysosomal exocytosis.^922^ This process is necessary for the treatment resistance of AKT-hyperactivated cancer cells. Downregulating TRPML1 or blocking the interaction between TRPML1 and ARL8B can inhibit AKT-driven tumorigenesis by promoting ferroptosis, and enhancing the sensitivity of radiotherapy and immunotherapy. These findings suggest that TRPML1 is a potential therapeutic target, and inducing ferroptosis by regulating TRPML1 is important for the treatment of AKT-hyperactivated cancer.^922^

#### Fatty acid desaturases 1 and 2 (FADS1/2)

TNBC is characterized by early recurrence and high metastasis, with limited treatment options. Lipid metabolism is generally dysregulated in TNBC, and fatty acid desaturases 1 and 2 (FADS1/2), which are responsible for PUFA biosynthesis, are highly expressed in TNBC with poor prognosis.^923^ Genetic interference and pharmacological approaches targeting FADS1/2 make TNBC cells resistant to ferroptosis.^923^ However, supplementation with exogenous PUFA to balance the PUFA/MUFA ratio increases the sensitivity of resistant tumors to ferroptosis.^923^ This suggests that FADS1/2 controls lipid metabolism and susceptibility to ferroptosis in TNBC and may serve as a potential target for future therapy.

#### Migration inhibitory factor (MIF)

Macrophage migration inhibitory factor (MIF) is a multipotent pro-inflammatory cytokine found in activated T lymphocytes and macrophages/monocytes, initially reported to be mainly involved in the innate and adaptive immune responses.^924^ MIF promotes cell DNA homologous repair by activating breast cancer type 1 susceptibility protein, thereby leading to resistance to ferroptosis in cancer cells. Inhibiting or knocking down MIF significantly enhances cell sensitivity to ferroptosis by impacting DNA repair mechanisms.^925^ This suggests that targeting MIF can increase the susceptibility of cancer cells to ferroptosis.

#### Histone deacetylase (HDAC)

Histone acetylation is a crucial epigenetic modification that maintains cell homeostasis. Histone deacetylases (HDACs), as key epigenetic regulators, also possess essential functions in regulating iron metabolism and ferroptosis. The HDAC1 inhibitor Entinostat (MS-275) is considered the most potent hepcidin agonist.^926^ Hepatic hepcidin expression is significantly reduced, and iron homeostasis is altered in hepatic *Hdac3*-specific knockout mice (*Hdac3*-LKO mice).^927^ Transcriptional analysis indicates that the Hippo signaling pathway may be downstream of HDAC3, and overexpression of transcriptional regulator YAP or inhibition of the Hippo pathway both significantly reduce hepcidin levels.^927^ Mechanically, inhibition of HDAC3 leads to increased YAP nuclear translocation through the Hippo pathway, altering hepcidin levels and contributing to ferroptosis induced by iron overload in liver injury.^927^ These results suggest that HDAC3 maintains iron homeostasis through the Hippo/YAP pathway and might represent a target to reduce ferroptosis in iron overload-related diseases. After intracerebral hemorrhage, the HDAC1/2 inhibitor Lomidesine (FK228) can alter microglial heterogenization, reduce neuroinflammation, and alleviate neuronal ferroptosis, possibly through regulating the Nrf2/HO-1 signaling pathway.^928^

#### Transient receptor potential melastatin 2 (TRPM2)

Hepatic I/R damage is a major complication of liver resection and transplantation and poses a serious clinical problem. TRPM2 is a non-selective cation channel with Ca^2+^ permeability.^929^ TRPM2 deficiency alleviates I/R-induced liver dysfunction and cell ferroptosis in mice, and ferroptosis induced by TRPM2 is related to the up-regulation of ALOX12 expression caused by mitochondrial Ca^2+^ accumulation.^930^ TRPM2 induces mitochondrial lipid peroxidation in hepatic I/R injury by increasing the expression of ALOX12, and TRPM2 inhibitors or calcium depletion significantly mitigate ferroptosis caused by hepatic IR injury both in vitro and in vivo.^930^ These findings suggest that inhibition of TRPM2 may provide an effective treatment strategy for diseases associated with ferroptosis.

#### Mg^2+^/Mn^2+^ dependent 1K (PPM1K)

Amino acid metabolism disorders are intricately linked to the onset of ferroptosis.^931^ Branched-chain amino acids (BCAAs), including isoleucine, leucine, and valine, all have short aliphatic side chains. High levels of BCAAs or branched alpha-ketoacids reduce the total antioxidant capacity of cells and significantly increase the production of lipid peroxidation products.^932^ After cerebral I/R injury, Mg^2+^/Mn^2+^ dependent 1K (PPM1K) expression in neuronal cells decreases, resulting in increased phosphorylation of BCAAs dehydrogenase E1α subunit (BCKDHA) S293, leading to BCAAs accumulation. BCAAs induce significant changes in genes and proteins associated with ferroptosis in neurons and increase lipid ROS levels, causing ferroptosis in primary cultured cortical neurons and ischemic tissue.^933^ BT2, a highly specific BCKD kinase inhibitor, promotes BCAAs oxidation by inhibiting BCKDHA phosphorylation, thereby reducing neuronal ferroptosis. In addition, BT2 alleviates neurobehavioral disorders in mice after I/R injury, with effects similar to those of Fer-1.^933^ These results suggest that PPM1K mediates the disturbance of BCAA metabolism, regulates cerebral I/R injury by activating neuronal ferroptosis, and may serve as a therapeutic intervention target.

#### Elabela- Apelin receptor axis

Elabela (ELA) is a novel ligand for the endogenous apelin receptor (Aplnr or APJ) that modulates oxidative stress and protects against cardiovascular diseases.^934^ Recent studies have shown increased expression of elabela and APJ in neurons after cerebral I/R injury, with typical morphological and molecular features associated with ferroptosis in neurons, such as mitochondrial shrinkage, iron, and MDA accumulation, upregulation of ACSL4 and ALOX15, and downregulation of GPX4 and xCT. The ELA-32 peptide, a mature form of elabela, alleviates neuronal ferroptosis, reduces cerebral infarction volume, and improves neurobehavioral deficits and cognitive dysfunction.^935^ Mechanistically, elabela interacts to APJ, activating the Nrf2/ARE pathway through guanine nucleotide binding protein alpha 13 subunit (Gα13). The elabela-APJ axis could mitigate neuronal ferroptosis following ischemic stroke, and the ELA-32 peptide may be a potential therapeutic candidate for ischemic stroke and cardiovascular diseases.

#### LncRNA WAC-AS1

Recent studies have found that ferroptosis can spread widely in the renal tissue microenvironment in a “wavy form”, forming a “ferroptosis wave” that results in extensive renal tubule necrosis and aggravates renal I/R injury. After I/R injury, the expression of lncRNA VEC-AS1 is upregulated in small extracellular vesicles secreted by renal TECs (IRI-sEVs).^936^ By inducing the expression of D-fructose-6-phosphate amidotransferase 1 in adjacent renal TECs, the flux of hexosamine biosynthesis pathway increases, and the level of BACH2 o-glycosylation modification rises, inhibiting its ubiquitination degradation.^936^ Furthermore, intranuclear BACH2 inhibits the transcription of GPX4 and SLC7A11 by binding to their promoter regions, thereby inducing ferroptosis and inducing the spread of “ ferroptosis wave” in the renal tissue microenvironment.^936^ GW4869, which inhibits sEVs biogenesis and secretion, or knocking out lncRNA WAC-AS1 in IRI-sEVs, significantly reduces the transmission of the “ferroptosis wave” and protects against renal I/R injury.^936^ Inhibition of sEV-mediated lncRNA WACC-AS1 secretion and targeting the “ferroptosis wave” may be novel strategies for ameliorating I/R injury.

#### lncRNA SEMA5A-IT1

Myocardial cell I/R injury is a common complication during cardiopulmonary bypass (CPB) cardiac surgery. The expression of lncRNA SEMA5A-IT1 in serum small circulating extracellular vesicles of patients after CPB is higher than that before CPB, which is inversely proportional to the level of creatine kinase-MB.^937^ lncRNA SEMA5A-IT1 in circulating small extracellular vesicles is taken up by cardiomyocytes to regulate the expression of Bcl-2 and SLC7A11, inhibiting ferroptosis by sponging miR-143-3p.^937^ SEMA5A-IT1 in peripheral blood circulating small extracellular vesicles is an important regulatory molecule protecting against myocardial I/R injury, providing a target for the prevention and treatment of tissue I/R injury.

### Potential therapeutic strategies for the treatment of human diseases

Small molecule inhibitors or inducers targeting ferroptosis have been widely used in preclinical practice and have demonstrated potential in treating diseases by modulating ferroptosis. Considering that iron metabolism, lipid metabolism, and redox pathways coordinately control ferroptosis, we have summarized bioactive molecules that affect ferroptosis by targeting core molecular of these three pathways.

#### Small molecules targeting ferroptosis by regulating iron metabolism

Iron is essential to many physiological processes; however, the Fenton chain reaction and the rapid increase of free radicals caused by excess iron led to the production of phospholipid hydroperoxide, resulting in ferroptosis. Excess iron has been reported in clinical studies of many diseases and is associated with disease progression and pathological grade. Therefore, regulating iron metabolism is one of the key strategies to target ferroptosis in the treatment of human diseases. For instance, iron chelators such as DFO, DFP, and artesunate significantly improve liver injury by inhibiting ferroptosis.^313–315^ As an iron chelating agent, DpdtbA has good antitumor activity against esophageal and gastric cancer by inhibiting ferritinophagy.^115^ Dihydroartemisinin can significantly inhibit the viability of leukemia cells by accelerating ferritin degradation,^136^ and increase the cytotoxicity of cisplatin to PDAC cells.^187^ Grape seed extract exerts antioxidation against ferroptosis and reduces calcium and iron levels in high fat-fed mice.^282^ FPN is the only known cellular iron exporting protein, and iron absorption and tissue distribution are mainly controlled by the interaction of FPN with hepcidin. Vamifeport (VIT-2763), as a novel oral FPN inhibitor, induces dose-dependent internalization and ubiquitination of FPN in cells, improving iron homeostasis in mouse models of β-thalassemia.^415^ Iron regulatory proteins play an important role in maintaining iron homeostasis in cells and mitochondria. BJP-IVb reduces iron content to prevent PD by inhibiting iron regulatory protein in dopaminergic neurons.^606^ Rapamycin also reduces the loss of dopamine neurons in the substantia nigra by regulating ferritinophagy and ferroptosis.^607^ The iron uptake inhibitor ferristatin II provides neuroprotection by suppressing ferroptosis, and HBED treatment alleviates secondary damage after TBI by binding to Fe^2+^ and converting Fe^2+^ to Fe^3+^.^675^

Although the above small molecule compounds can prevent iron overload-related diseases by regulating iron metabolism, only DFO, DPF, and DFX are currently approved for clinical use. More preclinical studies are needed to optimize iron metabolism regulation protocols in various iron overload conditions and to explore the best treatment strategies to reduce iron levels. These studies on iron chelators or iron absorption inhibitors will help to better understand their therapeutic promise in diseases caused by ferroptosis.

#### Small molecules targeting ferroptosis by regulating the SLC7A11/GPX4 pathway

The SLC7A11/GPX4 axis is vital in preventing cellular ferroptosis. SLC7A11, a key component system Xc^−^, regulates cystine intake and synthesis of GSH. GPX4 is a core inhibitor that directly reduces the formation of lipid hydroperoxides during ferroptosis. By converting lipid peroxides (L-OOH) into non-toxic lipids, GPX4 counteracts oxygen- and iron-dependent lipid peroxidation, with GSH acting as a crucial cofactor. Sulfapyridine increases the sensitivity of leukemia to chemotherapeutic agents by inhibiting SLC7A11 activity and cystine uptake.^215^ Astragalus inhibits ferroptosis in synovial cells and exerts a protective effect through activation of the SLC7A11/GPX4 axis.^364^ Moxibustion also improves synovial tissue by upregulating SLC7A11 and GPX4 expression, thereby reducing ferroptosis.^368^ Saikosaponin B2 improves CUMS-induced depressive behavior in a GPX4-dependent manner.^639^ Quercetin inhibits the activation of ATF3 and increases the expression of SLC7A11 and GPX4.^837^ C3G also increases GPX4 expression and GSH level, significantly inhibiting the ferroptosis of renal tubular cells.^838^

Although these findings have not yet been applied in the clinic, small molecules that regulate the SLC7A11/GPX4 pathway have been proven effective in the treatment of many diseases, at least preclinically. Future research should focus on the development of additional activators of the SLC7A11/GPX4 pathway with pharmaceutical properties and conduct clinical trials where appropriate.

#### Small molecules inhibiting ferroptosis by activating the Nrf2 pathway

Nrf2 is a well-established transcription factor crucial for antioxidant defense. It regulates numerous genes associated with ferroptosis, including those involved in GSH synthesis, NADPH regeneration, and iron metabolism (such as iron storage and export, as well as heme synthesis and degradation). Tagitinin C activates nuclear translocation of Nrf2 and expression of HO-1, increases unstable iron pools and lipid peroxidation, and inhibits the growth of CRC cancer cells.^126^ DMF, dehydroabietic acid, and Ginkgo biloba B can ameliorate alcoholic hepatitis mainly through the activation of the Nrf2 pathway.^328,331,332^ The Chinese medicine monomer ferulic acid improves cognitive dysfunction and reduces hippocampal neuron damage by activating SIRT1/Nrf2-mediated ferroptosis.^455^ Sulforaphane^503^ and dexmedetomidine^505^ both inhibit ferroptosis by activating the Nrf2 pathway to improve diabetic cardiomyopathy. Curcumin increases Nrf2 expression and its downstream targets HO-1 and GPX4 in hepatocytes,^452^ cardiomyocytes,^504^ neurons,^679^ renal tubule cells^865^ and chondrocytes,^882^ protecting against diseases by inhibiting ferroptosis. Eriodictyol significantly improves cognitive deficits by activating the Nrf2/HO-1 pathway to inhibit ferroptosis.^571^ Forsythiin A,^572^ salidroside,^576,604,808^ tetrahydroxy stilbene glycoside,^577^ and spermidine^578^ have similar mechanisms of inhibiting ferroptosis in AD, PD, and myocardial I/R injury. Morroniside protects dopaminergic neurons from ferroptosis in PD by activating the Nrf2 pathway and upregulating expression of GPX4 and SLC7A11.^609^ Edaravone inhibits ferroptosis through the SIRT1/Nrf2 signaling pathway and has therapeutic potential for depression, TBI and stroke.^634,666^ Tert-butylhydroquinonend and hinokitiol have also been shown to have neuroprotective effects by activating Nrf2.^680–682^

#### Small molecules targeting ferroptosis by regulating the ACSL4-LOX axis

Lipid peroxidation is a hallmark of ferroptosis, and lipid-metabolizing enzymes such as ACSL4 and LOX contribute to ferroptosis. Therefore, the ACSL4-LOX axis represents a significant pharmacological target for treating diseases associated with ferroptosis. Rosiglitazone inhibits ferroptosis by downregulating ACSL4 expression in a manner that is independent of PPAR-γ, thereby reducing arsenic-induced ferroptosis and MASH.^327^ Furthermore, liraglutide alleviates MAFLD associated with type 2 diabetes mellitus by activating AMPK/ACC pathway, which enhances lipolysis and inhibits ferroptosis.^334^ Astragaloside IV ameliorates myocardial injury in diabetic rats by inhibiting lipid deposition and reducing ferriotpsis.^507^ Nicorandil could inhibit mitochondrial translocation of ACSL4 and ferroptosis.^509^ AS-252424 is a specific and targeted ACSL4 inhibitor that binds directly to the glutamine 464 of ACSL4 to inhibit its enzymatic activity, thereby inhibiting lipid peroxidation and ferroptosis, effectively alleviating ferroptosis-mediated organ damage in mouse models, including I/R-induced kidney injury and acute liver injury.^938^ Abemaciclib is a potent and selective ACSL4 inhibitor that is effective in improving fatty liver and MASH in multiple mouse models.^939^ The combination of suberosin and thiazolidinedione effectively ameliorates heart injury by downregulating the ACSL4-LOX axis.^510^ Triacsin C can improve PD by inhibiting of ACSL4 activity,^602^ and clausenamide also improves behavioral deficits in PD mouse models by blocking nuclear translocation of ALOX5.^608^ Methyl ferulic acid alleviates neuropathic pain in mice by regulating the expression of ACSL4.^650^ Proanthocyanidin treatment significantly improves spinal cord injury by decreasing the expression of ACSL4 and ALOX15 in spinal cord tissue.^709^ These findings underscore the potential of targeting the ACSL4-LOX axis as a therapeutic strategy for diseases associated with ferroptosis.

#### Nanoparticles therapeutic strategies for the treatment human diseases

The above-mentioned small molecule ferroptosis regulators have demonstrated potential in preclinical practice for treating various diseases. However, the main obstacles to their clinical application are targeting and bioavailability. Modifying the biological, chemical, and physical properties of these small molecules can improve their release rate, solubility, and stability, distribution, and metabolism in vivo. These modifications can also enhance targeting and bioavailability, allowing for precise delivery and release, increased residence time at the disease site, and improved therapeutic effects. Nanoparticles are a common method to improve the bioavailability and retention time of bioactive compounds. Here, we summarize recent progress in improving the efficacy of small molecule compounds targeting ferroptosis by using nanotechnology.

Bioavailability of quercetin is an important issue and a serious obstacle for its use in AD treatment. Liu et al. developed a smart nanoparticle (TQCN) generated from quercetin and modified with triphenylphosphine for treatment of AD targeting ferroptosis.^940^ Utilizing favorable brain targeting and mitochondrial localization properties, TQCN effectively chelates iron through spontaneous coordination mediated by plant polyphenols and self-assemble metal-phenol nanocomplexes in situ to reduce iron overload and associated free radical outburst. Furthermore, TQCN activates the Nrf2 endogenous defense system, restores iron metabolism homeostasis, and reduces cellular lipid peroxidation.^940^ TQCN treatment may improve numerous neurodegenerative diseases related with brain iron accumulation and ameliorate severe cognitive impairment in AD mice due to its multiplex modulation of the pathogenic process that triggers ferroptosis.

Neuroprotective drugs such as edaravone, nerve growth factor, and neurotrophin protect the brain from oxidative stress and ferroptosis.^941^ However, these neuroprotective drugs often fail to achieve the expected therapeutic effect due to poor BBB permeability and short circulatory half-life.^942^ Zhang et al. developed a pH/GSH-supported polyamino acid nanogel (NG/EDA) based on the acidic pathological characteristics of ischemic tissue.^943^ NG/EDA is triggered by the acidic and edaravone-induced high levels of GSH microenvironment, enabling selective and sustained release of edaravone at the site of ischemic injury to enhance its neuroprotective effects.^943^ The results showed that NG/EDA could penetrate the BBB and accumulate efficiently at the site of cerebral ischemic injury in rats with pMCAO.^943^ NG/EDA significantly improves the survival rate of OGD neurons by inhibiting ferroptosis, and significantly decreases the infarct volume and neurobehavioral score of pMCAO mice.^943^ This pH/GSH dual-responsive nanoplatform may provide a unique and promising model for neuroprotection in cerebral I/R injury and other central nervous system disorders. In addition, Zhuge et al. prepared a pH-sensitive nanoplatform (OLCaP NP) using CaCO_3_ co-loaded oleanolic acid and LOX, designed to release drugs effectively in the acidic environments of tumors.^944^ The acidic conditions present at tumor sites stimulates the release of oleanolic acid and LOX, with oleanolic acid upregulating the expression of ACSL4 and collaborating with LOX to promote enzyme-mediated lipid peroxidation.^944^ This pH-responsive drug release mechanism, particularly effective in acidic tumor environments, making it a promising strategy to enhance the specificity and efficacy of cancer therapy.

The production of inflammatory cytokines is crucial in the pathogenesis of I/R injury diseases. Ding et al. reported a type of copper-based, neutrophil membrane-coated nanoparticles (N-Cu5.4O@DFO NPs) with high biocompatibility and stability.^945^ These nanoparticles demonstrate significant antioxidant properties and effectively scavenge iron, thereby mitigating oxidative damage and inflammatory responses ultimately improving the I/R damage.^945^ This development will greatly contribute to the advancement of other nano-antioxidants with multiple antioxidant properties and the treatment of I/R injury-related diseases.

Using antioxidants to eliminate ROS and inhibit ferroptosis presents a promising approach for the prevention and treatment of various diseases. Current research efforts are focused on developing safer and more efficient ROS-scavenging drugs.^946^ Rutin, a flavonoid glycoside derived from locust trees, possesses powerful antioxidant activity and has been extensively used in the treatment of cardiovascular and neurodegenerative diseases.^947,948^ Feng et al. developed rutin-loaded polydopamine nanoparticles (PEG-PDA@rutin NPs) to eliminate ROS and inhibit ferroptosis.^949^ The diameter of PEG-PDA@rutin NPs is about 100 nm, and they exhibit ROS-triggered drug release and excellent ROS clearance ability. PEG-PDA@rutin NPs can effectively enter cells, repair mitochondrial damage, eliminate ROS, and inhibit ferroptosis. In addition, in vivo imaging demonstrated that PEG-PDA@rutin NPs effectively accumulate in the kidneys following I/R injury and ameliorate renal I/R injury.^949^ The excellent ROS elimination ability of PPR NPs gives them potential to inhibit ferroptosis. Another type of polydopamine nanoparticles (PDA NPs) also effectively reduce Fe^2+^ deposition and lipid peroxidation, and alleviate myocardial I/R injury in mice.^950^ These works indicate the therapeutic effects of PDA NPs in combating ferroptosis.

Mitochondrial damage is a key factor in neuronal death, and mitochondrial quality control (MQC) is crucial in neuronal ferroptosis. Single-cell RNA sequencing results show that disturbed MQC aggravates ferroptosis through excessive mitochondrial fission and autophagy. Mitochondrial transfer mediated by mesenchymal stem cells (MSCs) could restore the mitochondrial pool of neurons and inhibit ferroptosis,^951^ providing a promising clinical translational strategy for ferroptosis-related central nervous system diseases based on stem cell-mediated mitochondrial therapy. It is of concern that the efficacy of MSCs is greatly limited by the local inflammatory microenvironment following SCI. Ren et al. constructed a synergistic drug release nanoparticle system combining MSCs and Fer-1 with a ROS-responsive drug nanocore mPEG-b-Lys-BECI-TCO for SCI repair.^952^ This combined treatment strategy could inhibit ferroptosis and inflammation after SCI, promote the recovery of nerve function in rats with SCI, and offer a new strategy for the construction of drug-synergistic cell therapy systems targeting ferroptosis.

Apigenin-7-O-Glucoside (AGL) is a novel flavonoid glycoside with strong antioxidant capacity. AGL can specifically bind to monoamine oxidase b and HO-1, which helps to inhibit Fe^2+^ accumulation and ROS production, thereby maintaining mitochondrial function and preventing ferroptosis.^953^ Nevertheless, AGL’s poor water solubility limits its practical application. To overcome this limitation, Zhao et al. synthesized two amphiphilic molecules mPEG-TK-DA and DTPA-N10-10 with ROS-scavenging functions, and self-assembled AGL through hydrophobic and hydrophilic interactions, forming multi-site ROS-scavenging nanoparticles called PDN@AGL. PDN@AGL inhibits ferroptosis by reducing ROS levels and lipid peroxidation, and the regulation of the ATF3/SLC7A11 pathway is believed to play a crucial role in this process.^954^ The regulation of ATF3/SLC7A11-mediated ferroptosis underpins the potential application of PDN@AGL in treating human diseases. By addressing the solubility issue and enhancing the antioxidant capacity of AGL, PDN@AGL represents a promising therapeutic strategy for diseases characterized by oxidative stress and ferroptosis.

Clinical use of corticosteroids or non-steroidal anti-inflammatory drugs and adjuvant targeted exercise therapy is common in the conservative treatment of OA. However, long-term drug treatment can lead to a series of side effects, limiting their therapeutic effectiveness.^955^ Yu et al. used the Mannich condensation reaction between the antioxidant selenomethionine and EGCG to efficiently synthesize a polyphenol-based nanomedicine (ES NDs) in an aqueous medium for OA treatment. ES NDs effectively reduce the abnormal accumulation of Fe^2+^, GPX4 inactivation, and lipid peroxidation in chondrocytes, thereby improving the metabolic disorders of chondrocytes caused by ferroptosis, and show significant therapeutic effects in alleviating OA.^956^ Intra-articular delivery of ES NDs may be a promising strategy for the treatment of OA and other arthritic diseases.

Previous studies have found that activation of TRPV1 can significantly reduce cartilage degeneration by upregulating GPX4 and eliminating ferroptosis in chondrocytes.^957^ Given the thermal activation properties of TRPV1, citrate-stabilized gold nanorods coupled to a TRPV1 monoclonal antibody formed Cit-AuNRs@Anti-TRPV1, which can be used as a photothermal switch for TRPV1 activation in chondrocytes under near infrared (NIR) irradiation.^958^ Cit-AuNRs@Anti-TRPV1 show flexible photothermal response and good biocompatibility under NIR irradiation. Intra-articular injection of Cit-AuNRs@Anti-TRPV1 alleviates cartilage degradation by activating TRPV1 to inhibit ferroptosis in chondrocytes, reduces pain and improves physical activity in mice with medial meniscus instability-induced OA.^958^ The efficacy of Cit-AuNRs@Anti-TRPV1 in protecting cartilage cells from ferroptosis under NIR irradiation also provides a potential therapeutic strategy for treatment of other types of arthritis.

Diabetic osteoporosis is an important complication among diabetic patients. High glucose levels in diabetic patients affect the osteogenic differentiation of BMSCs, leading to osteoporosis. Li et al used tetrahedral framework nucleic acid (tFNA) to synthesize tFNA-Cur, a nanoparticle that can deliver the natural compound curcumin to the bone marrow, thereby enhancing the bioavailability and stability of curcumin.^959^ tFNA-Cur can enhance mitochondrial function by activating the Nrf2/GPX4 pathway in vitro and in vivo, inhibit ferroptosis of BMSCs, promote osteogenic differentiation of BMSCs under a high glucose microenvironment, and promote bone formation.^959^ tFNA-Cur also has great potential for treating other diseases related to ferroptosis.

#### Other therapeutic strategies for the treatment of human diseases

In addition to nanomedical drug delivery systems, other modification technologies such as micromicellar systems and GalNAc modification are also used to improve the bioavailability of small molecule candidates targeting ferroptosis.

Hepcidin is negatively regulated by TMPRSS6, which inhibits hepcidin expression by suppressing Hjv activity and the bone morphogenetic protein-signaling pathway.^960,961^ Inhibition of TMPRSS6 expression may therefore be a promising therapeutic strategy for ameliorating anemia and iron overload in β-thalassemia. In Hbb^th3/+^ mice, deletion of the TMPRSS6 gene improves ineffective erythropoiesis and anemia.^962^ Downregulating TMPRSS6 using small interfering RNA (siRNA) induces hepcidin expression, reducing tissue and serum iron levels in Hbb^th3/+^ mice.^963^ The main challenge of oligonucleotide therapy is to achieve effective and targeted delivery to tissues and cells. N-acetylgalactosamine (GalNAc) ligand is a specific liver-targeting fragment.^964^ Vadolas et al. developed a novel siRNA called SLN124, consisting of a trimeric GalNAc ligand coupled to TMPRSS6-siRNA.^965^ SLN124 normalizes hepcidin expression and reduces anemia in Hbb^th3/+^mice.^965^ Combined with DFP, SLN124 is more effective in reducing liver iron overload than treatment alone, representing a promising pharmacological therapy for the treatment of ferroptosis-related diseases.

Due to the fragile and deformable nature of the spinal cord, hydrogels that are too hard cannot be used to treat SCI. Ying et al. developed shear-thinning hydrogels called TPA@Laponite. Shear-thinning hydrogels can transition from a gel state to a flow state when subjected to shear forces,^966^ making them ideal for soft tissue treatments such as SCI due to their deformability.^967^ In addition, TPA@Laponite effectively scavenges ROS, inhibits ferroptosis by regulating iron metabolism and enhancing vascular function, and promotes the recovery of SCI. Since neurons cannot be regenerate after SCI, exogenous neurons need to be replenished. Introducing of dental pulp stem cells into TPA@Laponite effectively reduces muscle spasm and promotes the recovery of SCI by regulating the ratio of excitatory and inhibitory synapses.^966^ This type of shear-thinning, ROS-scavenging hydrogels also has significant potential for treating other tissue trauma.

The change of brain microenvironment caused by acute cerebral I/R is the main obstacle to nerve function recovery, and a significant reason of stroke recurrence after thrombolytic therapy.^968^ Jiang et al. developed targeted crosslinked micelles loaded with idebenone to reduce infarct size by curbing neuronal ferroptosis and glial overactivation.^969^ CREKA is an oligopeptide with specific biomimetic properties. In a rat model, CREKA peptide-modified micelles preferentially accumulate in the ischemic brain tissue by binding to microthrombi within the ipsilateral microvessels. Under ROS stimulation, the diselenide bond in the micelle transforms into hydrophilic selenite acid, achieving the dual effect of responsive drug release and ROS consumption. This prevents neuronal ferroptosis induced by oxidative stress, alleviates glia overactivation, inhibits the secretion of pro-inflammatory factors, and regulates the inflammatory microenvironment.^969^ This multifunctional therapeutic micelle demonstrates a proof-of-concept for treating central nervous system diseases by inhibiting glia overactivation and neuronal ferroptosis, thereby reshaping the pathological microenvironment.

The primary treatment for ischemic stroke involves thrombolytic therapy with recombinant tissue plasminogen activator (rtPA). However, rtPA therapy can dramatically enhance the risk of hemorrhagic conversion and BBB permeability.^970^ Geng designed a macrocyclic vector using glucose-modified azocalix[4]arene (GluAC4A) to transport liproxstatin-1 to the ischemic site.^971^ With the help of glucose transporter 1 on the surface of the BBB, glucose-modified GluAC4A is transported into the brain, achieving targeted drug delivery across the BBB.^971^ GluAC4A binds closely to liproxstatin-1, significantly improving its solubility, and liproxstatin-1 is released due to the presence of azo groups and GluAC4A’s reactivity to hypoxia at the site of ischemia.^971^ Lip@GluAC4A greatly decreases ferroptosis, enhances BBB permeability, and alleviates neurological dysfunction caused by rtPA therapy, all of which have important implications for the treatment and prognosis of patients with ischemic stroke.

During the acute phase of ischemic stroke, anti-inflammatory M2 microglia show a brief increase followed by a rapid decline. Fxr2 in adipocytes derived from stem cell-derived exosomes (ADSC-Exos) regulate the expression of ATF3/SLC7A11, effectively reducing the susceptibility of M2 microglia to ferroptosis. This mechanism helps inhibit inflammatory microenvironment in ischemic sites, and promotes the recovery of neural function after brain I/R injury. Based on this, Wang et al. developed more effective exosomes targeting M2 microglia called M2pep-ADSC-Exos. M2pep-ADSC-Exos specifically target M2 microglia, substantially reducing their susceptibility to ferroptosis and enhancing neurological function in ischemic stroke mice.^972^ M2pep-ADSC-Exos represent a novel therapeutic strategy for treating neurological disorders linked to ferroptosis.

These chemical or physical modifications of small molecule ferroptosis modulators improve their bioavailability and targeting, and significantly increase their clinical application potential. While these modified drug candidates have only been shown to be effective for certain diseases, they also have the potential for treating other diseases associated with ferroptosis.

## Clinical therapy of targeting ferroptosis in human diseases

Numerous preclinical studies have demonstrated that iron acts as a potent pro-oxidant, contributing to the development and progression of tumors, central nervous system diseases, metabolic diseases, autoimmune diseases, and cardiovascular diseases through the production of highly reactive and cytotoxic free radicals. Clinically, serum iron overload (indicated by high ferritin levels and high transferrin saturation) is associated with poor functional outcomes in patients. Ferritin is a primary form of iron storage in the human body, capable of binding and storing iron to maintain the iron supply and the relative stability of hemoglobin. The levels of serum ferritin can reflect the body’s iron reserves and are currently the most sensitive indicator for detecting iron deficiency or iron overload. It is noteworthy that serum ferritin is an acute-phase protein, and its level may be influenced by factors such as infection, inflammation, and tumors. Currently, the threshold of serum ferritin concentration under various physiological or pathological conditions is still not clearly defined. Future clinical trials are still needed to determine the appropriate serum ferritin concentration thresholds. In clinical treatment, the drugs currently undergoing clinical trials are mainly iron chelating agents and antioxidants, detailed in Table 2.

**Table 2:** Clinic trials targeting ferroptosis in human diseases

Human diseases or safety evaluation	Conditions	Interventions	NCT Number
Safety evaluation	Healthy Volunteers	BPM31510 (a lipid-drug conjugate nanodispersion)	NCT03002935, NCT02486055
		Coenzyme Q10	NCT03429231, NCT05680857
		Deferiprone	NCT02465489, NCT02442310, NCT02189941, NCT01989455
		Deferasirox	NCT00419172, NCT00427505
		N-Acetylcysteine	NCT00552786, NCT01271088, NCT02723669, NCT02206178, NCT00434005
Cancers	Breast Cancer	Coenzyme Q10	NCT00976131, NCT00096356
	Hepatocellular Carcinoma	Coenzyme Q10	NCT01964001
	Melanoma	N-acetylcysteine	NCT01612221
	Myeloid Leukemia	Deferasirox	NCT01273766
	Pancreatic Cancer	BPM31510	NCT02650804
Metabolic disorders	Acute Liver Failure	N-Acetylcysteine	NCT00004467, NCT00248625, NCT03679442, NCT02182167, NCT03759158, NCT01394497
	End Stage Liver Failure	N-Acetylcysteine	NCT00736541
	Acute Lung Injury	N-Acetylcysteine	NCT00655928
	Acute Renal Failure	N-Acetylcysteine	NCT01612013, NCT01907061, NCT00353340, NCT01467466, NCT00188630, NCT00187330, NCT01394419, NCT00736866, NCT02761577, NCT00356954, NCT00211653, NCT00122018
	Chronic Kidney Disease	N-Acetylcysteine	NCT00498342, NCT00506506, NCT05264584, NCT01232257, NCT00572663, NCT04916080, NCT03636932
		Coenzyme Q10	NCT03579693
	End-Stage Renal Disease	N-Acetylcysteine	NCT00440869, NCT00188630, NCT00187330
	Contrast-induced Nephropathy	N-Acetylcysteine	NCT01160627, NCT00497328, NCT00830193, NCT00492518, NCT00237614
		Deferiprone	NCT01146925
	Kidney Transplantation	N-Acetylcysteine	NCT00851708
	Alcoholic Hepatitis	N-Acetylcysteine	NCT03707951, NCT03216954, NCT03220776, NCT05840640, NCT00863785, NCT00962442, NCT00568087, NCT01214083
	Diabetes	Coenzyme Q10	NCT03111433, NCT02062034, NCT00703482
		N-Acetylcysteine	NCT00493727, NCT00463671, NCT00915200, NCT00556465, NCT01265563, NCT01082445, NCT00337038, NCT01386645, NCT00188773, NCT02206152, NCT00609102, NCT01394510
	Hypercholesterolemia	Coenzyme Q10	NCT06391606
	Obesity	N-Acetylcysteine	NCT01550432, NCT02117700
	Chronic Obstructive Pulmonary Disease	N-Acetylcysteine	NCT02818270, NCT01136239, NCT00969904, NCT03388853
Genetic disorders	Thalassemia	Amlodipine	NCT02065492
		Deferoxamine	NCT00000588, NCT00000595, NCT00000623, NCT00061750, NCT00733811, NCT00105495, NCT01369719
		Deferiprone	NCT03591575, NCT00733811
		Deferasirox	NCT00879242, NCT00171301, NCT00447694, NCT00061763, NCT01610297, NCT00105495, NCT01905774, NCT00901199, NCT00235391, NCT00560820, NCT03637556
		N-acetylcysteine	NCT04260516
	Non-transfusion Dependent Thalassemia	Deferasirox	NCT01709838
	Hemosiderosis	Deferasirox	NCT00303329, NCT00631163, NCT02125877, NCT00673608, NCT01394029, NCT00845871
		Deferiprone	NCT00350662, NCT00349453
		Desferrioxamine	NCT00350662, NCT00349453
		N-acetylcysteine	NCT02481609
	Transfusional Iron Overload	Deferiprone	NCT00529152, NCT03802916
		Deferasirox	NCT01376622, NCT00654589, NCT01335035, NCT01874405, NCT03372083, NCT01838291, NCT01044186, NCT00379483, NCT00171210, NCT00600938, NCT00390858, NCT02435212, NCT00749515, NCT00171821
	Sickle Cell Anemia	Deferoxamine	NCT00067080
		Deferasirox	NCT05392101, NCT00110617, NCT01090323
		Deferiprone	NCT01835496
		N-acetylcysteine	NCT01849016, NCT01800526
	Cystic Fibrosis	N-acetylcysteine	NCT00809094
	Duchenne Muscular Dystrophy	Coenzyme Q10	NCT01126697, NCT00033189
		Idebenone	NCT00758225, NCT00654784, NCT01027884
	Freidreich's Ataxia	Deferiprone	NCT00530127, NCT00897221
		EPI-743 (Vatiquinone, α-Tocotrienol quinone)	NCT01728064
		Idebenone	NCT00993967, NCT00078481, NCT00015808, NCT00905268, NCT00697073, NCT01303406
	Leber's Hereditary Optic Neuropathy	Idebenone	NCT00747487, NCT02771379, NCT02774005
	Myelodysplastic Syndrome	Deferiprone	NCT02477631
		Deferasirox	NCT00110266, NCT01250951, NCT00940602, NCT00564941, NCT00469560, NCT00117507, NCT00481143
Cardiovascular diseases	Cardiac Arrest	Coenzyme Q10	NCT01319110
	Cardiac Iron Overload	Deferasirox	NCT01254227
		Deferoxamine	NCT01254227
	Diabetic Cardiomyopathy	Coenzyme Q10	NCT02255682, NCT02115581
	Hypertrophic Cardiomyopathy	N-acetylcysteine	NCT01537926
	Myocardial Infarction	N-Acetylcysteine	NCT01218178, NCT01501110
	Atherosclerosis	N-acetylcysteine	NCT02422927
		Coenzyme Q10	NCT00908297
	Coronary Artery Disease	Coenzyme Q10	NCT01424761, NCT01163500, NCT00860847
		N-acetylcysteine	NCT01021163
Central nervous system diseases	Parkinson’s Disease	Coenzyme Q10	NCT00076492, NCT01892176, NCT03061513, NCT00004731, NCT00180037
		Deferiprone	NCT02655315, NCT00943748, NCT02728843, NCT01539837 NCT02880033
		EPI-743	NCT01923584
		N-acetylcysteine	NCT01470027, NCT02212678, NCT02445651, NCT01427517
	Huntington's Disease	Coenzyme Q10	NCT00980694, NCT00920699
	Autism Spectrum Disorder	N-acetylcysteine	NCT03008889, NCT00889538, NCT00676195, NCT00627705, NCT00453180
	Depression	N-acetylcysteine	NCT02269540
	Bipolar Depression	Coenzyme Q10	NCT00720369, NCT01390389
		N-acetylcysteine	NCT02294591, NCT02357290, NCT01797575, NCT05340504, NCT03730064
	Cannabis Dependence	N-acetylcysteine	NCT01005810, NCT00542750, NCT01675661, NCT03055377
	Cocaine Abuse	N-acetylcysteine	NCT02141620, NCT00218491, NCT02994875, NCT02124941, NCT00136825, NCT03556371
	Methamphetamine Abuse	N-acetylcysteine	NCT01063205, NCT00332605, NCT04405193
	Nicotine Dependence	N-acetylcysteine	NCT00751257, NCT02723162, NCT02737358,
	Obsessive-compulsive Disorder	N-acetylcysteine	NCT01172275, NCT01555970
	Neuropathic Pain	N-acetylcysteine	NCT01840345, NCT03354572
		Coenzyme Q10	NCT00997269
	Traumatic Brain Injury	N-acetylcysteine	NCT00724594, NCT01322009, NCT04291066, NCT01515839, NCT02791945
	Cognitive Dysfunction	N-acetylcysteine	NCT00611897
	Schizophrenia	N-acetylcysteine	NCT01506765, NCT01232790, NCT01885338, NCT02505477, NCT01339858, NCT03510741, NCT01354132
	Dry Eye Syndrome	Coenzyme Q10	NCT03074344
		N-acetylcysteine	NCT01747616, NCT01753752, NCT01278784, NCT01015209
Ischemia-reperfusion injury	Hepatectomy Reperfusion Injury	Deferasirox	NCT00432627
		N-acetylcysteine	NCT01223326, NCT00564642
	Hypoxic-ischemic Encephalopathy	N-acetylcysteine	NCT04643821
Musculoskeletal diseases	Amyotrophic Lateral Sclerosis	Deferiprone	NCT02164253
		Coenzyme Q10	NCT00243932
	Skeletal Muscle Damage	N-acetylcysteine	NCT02930031, NCT04523675, NCT01778309

### Advances in clinical treatment of cancer

The physical state of cancer patients undergoing treatment significantly impacts their quality of life, emotional health, and treatment tolerance. CoQ10 has been used as a dietary supplement for health maintenance for decades, with its benefits extensively evaluated for cardiovascular and neurodegenerative diseases to improve oxidative stress in patients. A single-blind, randomized, controlled trial (NCT01964001) in patients with primary HCC found that CoQ10 supplementation for 12 weeks post-surgery significantly improved antioxidant capacity and reduced oxidative stress and inflammation levels.^973^ Currently, clinical research on tumor patients is very limited and mainly focuses on postoperative rehabilitation.

### Advances in clinical treatment of metabolic disorders

N-acetylcysteine (NAC) is a thiol-containing antioxidant and a precursor of GSH, exerting an indirect antioxidant effect by inducing GSH synthesis. It is also a commonly used ferroptosis inhibitor. Currently, NAC is involved in numerous clinical trials for patients with metabolic disorders. For example, intravenous NAC improves transplant-free survival (NCT00004467) and graft survival (NCT01394497) in patients with early acute liver failure.^974,975^ However, other studies have shown that NAC does not reduce the risk of contrast-induced acute kidney injury or improve clinical symptoms in patients with diabetes (NCT00736866).^976^

Empagliflozin, a sodium-glucose cotransporter 2 inhibitor, is a novel hypoglycemic agent that increases urinary glucose and sodium excretion. Excitingly, clinical trials have proven that empagliflozin significantly reduces the relative risk of hospitalization for heart failure and cardiovascular death in T2DM patients with cardiovascular disease.^977^ These beneficial effects could not be entirely attributed to glucose-lowering or natriuretic action, and are also related to maintaining the intracellular iron balance.

### Advances in clinical treatment of genetic disorders

Individuals with sickle cell disease (SCD), thalassemia, or other anemias require long-term blood transfusions, often leading to iron overload that necessitates iron chelating therapy. Iron chelating agent DFP is commonly used in the treatment of thalassemia syndrome patients, effectively reducing serum ferritin, and increasing transferrin saturation, and is well-tolerated.^978^ An open-label randomized noninferiority study (NCT02041299) on SCD patients showed that DFP and DFO were equally effective in reducing liver iron concentrations, with acceptable efficacy and safety, consistent with results seen in transfusion-dependent thalassemia patients.^979^ Deferoxamine mesylate (DFO) has long been used clinically to treat iron overload caused by thalassemia.^980,981^ A Phase 3, multicenter, randomized trial (EudraCT 2012-000353-31, NCT01825512) compared the efficacy of DFX and DFP in pediatric patients with transfusion-dependent haemoglobinopathies and found that DFP was more effective and safer in controlling iron overload than DFX over a 12-month treatment period.^982^

Iron chelation therapy may help improve cardiac function in patients with transfusion-dependent myelodysplastic syndrome (MDS) due to cardiac iron overload from repeated blood transfusion. A prospective, placebo-controlled, randomized study (NCT00940602) found that DFX significantly reduced the combined risk of hospitalization for worsening heart function or congestive heart failure in MDS patients, but had no significant effect on left ventricular ejection fraction or pulmonary artery pressure.^983^ DFO and DFP have also been reported to have a similar effect.^311,981,984^ Combining the calcium channel blocker amlodipine with iron chelation therapy may enhance the efficacy of iron chelation, and provide a novel strategy for preventing and treating cardiac iron overload in patients with thalassemia.^985^

Limiting iron availability by inhibiting FPN activity is a novel approach to treat β-thalassemia. Richard et al. evaluated the pharmacodynamics, pharmacokinetics, safety, and tolerability of the single and multiple up-dose oral FPN inhibitor VIT-2763 in healthy volunteers and found that VIT-2763 has a safety profile similar to placebo and is well tolerated, with no serious adverse events and discontinuations due to adverse events. Both single administration of VIT-2763 (more than 60 mg) and multiple administrations reduce serum ferritin and transferrin saturation.^986^ These results support clinical studies of VIT-2763 in patients with thalassemia and suggest that VIT-2763 could be used clinically as a potential inhibitor of ferroptosis for treating ferroptosis-related diseases.

### Advances in clinical treatment of cardiovascular diseases

DFX monotherapy has been shown to be effective in reducing myocardial and hepatic iron concentrations. Combined treatment with DFX-DFO rapidly reduces myocardial and hepatic iron concentrations and significantly improves myocardial function and ejection fraction in patients with severe transfusional myocardial siderosis (NCT01254227).^311^ Antioxidant NAC therapy prevents oxidative balance disturbance in patients with acute myocardial infarction (NCT01501110),^987^ and affects myocardial hypertrophy or fibrosis (NCT01537926).^988^ CoQ10 supplementation (300 mg/day) significantly increases antioxidant enzyme activity in patients with coronary artery disease and reduces side effects of statins (NCT01424761).^989^

### Advances in clinical treatment of central nervous system diseases

Iron chelation therapy is also a neuroprotective strategy. A multicenter, randomized, double-blind, placebo-controlled Phase 2 clinical trial (EudraCT 2007-0006731-31, NCT00777140) evaluated the safety, tolerability, and potential efficacy of DFO in patients with acute ischemic stroke in the middle cerebral artery region. The results showed that DFO is safe and well tolerated in stroke patients, with no difference in adverse effects compared to placebo. DFO (40–60 mg/kg/day) significantly reduces serum transferrin saturation in stroke patients and has a positive effect on patients with moderate to severe ischemic stroke. At day 90, 50–58% of patients in the DFO group have favorable outcomes, much higher than those in the placebo group (31%), suggesting that iron chelating therapy reduces systemic iron overload and may provide long-term benefits for patients with acute stroke.^726^ Another clinical study (NCT02175225) has confirmed that DFO also positively affects the recovery of intracerebral hemorrhage patients.^990–992^ A randomized, blind, placebo-controlled crossover study (NCT013655104) has found that DFO increases plasma erythropoietin and VEGF concentrations, resulting in significant time- and age-dependent improvements in cerebrovascular reactivity in the elderly.^993^ DFO reduces nigrostriatal iron in patients with PD, and 36 weeks of DFO treatment significantly reduces PD scores compared to placebo in early-stage patients with PD who have never been treated with levodopa (NCT02655315).^994^ Iron chelating therapy may be a promising neuroprotective strategy for elderly patients.

Despite the increasing number of research articles and reviews elaborating on the mechanisms of ferroptosis, the development of ferroptosis inducers and inhibitors has been slow, with few drugs reaching clinical trials and practice. Omaveloxolone has recently been approved by the US FDA for the treatment of Friedreich’s Ataxia,^995^ although its relationship with ferroptosis has not yet been reported in the literature. Its action as an Nrf2 agonist suggests it might treat diseases by inhibiting ferroptosis. These encouraging developments give us great confidence in clinical treatments targeting ferroptosis.

## Limitationa and perspectives

Targeting ferroptosis appears to have a favorable impact on the clinical treatment of multiple human diseases. However, several issues must be addressed before clinical applications can proceed. First, current research on ferroptosis has only touched on the phenomenon, and the detailed roles of ferroptosis in the onset and development of human diseases have not been deeply studied, posing a challenge for precision medicine. Second, targeting ferroptosis seems to be a double-edged sword. Previous studies have focused more on disease-related tissues and cells, and the toxicity of ferroptosis inducers or inhibitors in other organs remains largely unknown. The interaction between ferroptosis and other modes of cell death adds complexity and uncertainty of the research. Third, although several characteristics and serum biomarkers such as serum transferrin saturation and ferritin have been suggested, accurately measuring ferroptosis, especially in vivo, remains a significant challenge. Finally, most studies are preclinical, based on cell and animal models, and lack valid clinical evidence. The clinical application of iron chelating agents is mostly in patients with thalassemia or hemochromatosis. Clinical trials for other diseases are ongoing but have not yet obtained definitive results. Therefore, more clinical trials are needed to verify the effectiveness of ferroptosis-based treatments for human diseases.

## Conclusions

Unstable iron deposits within cells lead to an unbalanced redox state, causing a type of cell death known as ferroptosis which differs from other types of death. Furthermore, ferroptosis interacts with other forms of cell death, including apoptosis, autophagy, and pyroptosis, with growing evidence of crosstalk between these processes. These interactions make the role of ferroptosis in human diseases more complicated and variable. Fortunately, we are in an exciting era of rapid scientific and technological discovery, and rapid advances in ferroptosis research have made it possible to overcome the above challenges and successfully translate personalized ferroptosis treatments into clinical practice. Research into the pathways and interactive networks associated with ferroptosis in various pathological as well as physiological circumstances will greatly advance our comprehension of ferroptosis and how it might be beneficial in in treating human diseases.

## References

[1] Stockwell, B. R. Ferroptosis turns 10: emerging mechanisms, physiological functions, and therapeutic applications. *Cell* **185**, 2401–2421 (2022). 35803244 10.1016/j.cell.2022.06.003 PMC9273022

[2] Dixon, S. J. & Olzmann, J. A. The cell biology of ferroptosis. *Nat. Rev. Mol. Cell Biol.* **25**, 424–442 (2024). 38366038 10.1038/s41580-024-00703-5 PMC12187608

[3] Jiang, X., Stockwell, B. R. & Conrad, M. Ferroptosis: mechanisms, biology and role in disease. *Nat. Rev. Mol. Cell Biol.* **22**, 266–282 (2021). 33495651 10.1038/s41580-020-00324-8 PMC8142022

[4] Li, J. et al. Ferroptosis: past, present and future. *Cell Death Dis.* **11**, 88 (2020). 32015325 10.1038/s41419-020-2298-2 PMC6997353

[5] Berndt, C. et al. Ferroptosis in health and disease. *Redox Biol.* **75**, 103211 (2024). 38908072 10.1016/j.redox.2024.103211 PMC11253697

[6] Zeng, F. et al. Ferroptosis detection: from approaches to applications. *Angew. Chem. Int. Ed. Engl.* **62**, e202300379 (2023). 36828775 10.1002/anie.202300379

[7] Pan, J. et al. The imbalance of p53-Park7 signaling axis induces iron homeostasis dysfunction in doxorubicin-challenged cardiomyocytes. *Adv. Sci.* **10**, e2206007 (2023). 10.1002/advs.202206007 PMC10214246 36967569

[8] Liang, D., Minikes, A. M. & Jiang, X. Ferroptosis at the intersection of lipid metabolism and cellular signaling. *Mol. Cell* **82**, 2215–2227 (2022). 35390277 10.1016/j.molcel.2022.03.022 PMC9233073

[9] Tang, D., Chen, X., Kang, R. & Kroemer, G. Ferroptosis: molecular mechanisms and health implications. *Cell Res.* **31**, 107–125 (2021). 33268902 10.1038/s41422-020-00441-1 PMC8026611

[10] Li, W. et al. FSP1: a key regulator of ferroptosis. *Trends Mol. Med.* **29**, 753–764 (2023). 37357101 10.1016/j.molmed.2023.05.013

[11] Dixon, S. J. et al. Ferroptosis: an iron-dependent form of nonapoptotic cell death. *Cell* **149**, 1060–1072 (2012). 22632970 10.1016/j.cell.2012.03.042 PMC3367386

[12] Peng, F. et al. Regulated cell death (RCD) in cancer: key pathways and targeted therapies. *Signal Transduct. Target Ther.* **7**, 286 (2022). 35963853 10.1038/s41392-022-01110-y PMC9376115

[13] Liao, M. et al. Targeting regulated cell death (RCD) with small-molecule compounds in triple-negative breast cancer: a revisited perspective from molecular mechanisms to targeted therapies. *J. Hematol. Oncol.* **15**, 44 (2022). 35414025 10.1186/s13045-022-01260-0 PMC9006445

[14] Qin, R. et al. Naturally derived indole alkaloids targeting regulated cell death (RCD) for cancer therapy: from molecular mechanisms to potential therapeutic targets. *J. Hematol. Oncol.* **15**, 133 (2022). 36104717 10.1186/s13045-022-01350-z PMC9471064

[15] Sun, S. et al. Targeting ferroptosis opens new avenues for the development of novel therapeutics. *Signal Transduct. Target Ther.* **8**, 372 (2023). 37735472 10.1038/s41392-023-01606-1 PMC10514338

[16] Aisen, P., Enns, C. & Wessling-Resnick, M. Chemistry and biology of eukaryotic iron metabolism. *Int. J. Biochem. Cell Biol.* **33**, 940–959 (2001). 11470229 10.1016/s1357-2725(01)00063-2

[17] Zhang, D. D. Ironing out the details of ferroptosis. *Nat. Cell Biol.* **26**, 386–1393 (2024). 10.1038/s41556-024-01361-7 38429476

[18] Hentze, M. W., Muckenthaler, M. U., Galy, B. & Camaschella, C. Two to tango: regulation of Mammalian iron metabolism. *Cell* **142**, 24–38 (2010). 20603012 10.1016/j.cell.2010.06.028

[19] Hentze, M. W., Muckenthaler, M. U. & Andrews, N. C. Balancing acts: molecular control of mammalian iron metabolism. *Cell* **117**, 285–297 (2004). 15109490 10.1016/s0092-8674(04)00343-5

[20] Yu, Y. et al. Hepatic transferrin plays a role in systemic iron homeostasis and liver ferroptosis. *Blood* **136**, 726–739 (2020). 32374849 10.1182/blood.2019002907 PMC7414596

[21] Dutt, S., Hamza, I. & Bartnikas, T. B. Molecular mechanisms of iron and heme metabolism. *Annu Rev. Nutr.* **42**, 311–335 (2022). 35508203 10.1146/annurev-nutr-062320-112625 PMC9398995

[22] Park, C. H., Valore, E. V., Waring, A. J. & Ganz, T. Hepcidin, a urinary antimicrobial peptide synthesized in the liver. *J. Biol. Chem.* **276**, 7806–7810 (2001). 11113131 10.1074/jbc.M008922200

[23] Gao, J. et al. Interaction of the hereditary hemochromatosis protein HFE with transferrin receptor 2 is required for transferrin-induced hepcidin expression. *Cell Metab.* **9**, 217–227 (2009). 19254567 10.1016/j.cmet.2009.01.010 PMC2673483

[24] Babitt, J. L. et al. Bone morphogenetic protein signaling by hemojuvelin regulates hepcidin expression. *Nat. Genet.* **38**, 531–539 (2006). 16604073 10.1038/ng1777

[25] Arezes, J. et al. Erythroferrone inhibits the induction of hepcidin by BMP6. *Blood* **132**, 1473–1477 (2018). 30097509 10.1182/blood-2018-06-857995 PMC6238155

[26] Nemeth, E. et al. IL-6 mediates hypoferremia of inflammation by inducing the synthesis of the iron regulatory hormone hepcidin. *J. Clin. Investig.* **113**, 1271–1276 (2004). 15124018 10.1172/JCI20945 PMC398432

[27] Roemhild, K. et al. Iron metabolism: pathophysiology and pharmacology. *Trends Pharm. Sci.* **42**, 640–656 (2021). 34090703 10.1016/j.tips.2021.05.001 PMC7611894

[28] Mastrogiannaki, M. et al. Hepatic hypoxia-inducible factor-2 down-regulates hepcidin expression in mice through an erythropoietin-mediated increase in erythropoiesis. *Haematologica* **97**, 827–834 (2012). 22207682 10.3324/haematol.2011.056119 PMC3366646

[29] Beavers, C. J. et al. Iron deficiency in heart failure: a scientific statement from the heart failure society of America. *J. Card. Fail.* **29**, 1059–1077 (2023). 37137386 10.1016/j.cardfail.2023.03.025

[30] Pasricha, S. R., Tye-Din, J., Muckenthaler, M. U. & Swinkels, D. W. Iron deficiency. *Lancet* **397**, 233–248 (2021). 33285139 10.1016/S0140-6736(20)32594-0

[31] Roy, R., Kuck, M., Radziwolek, L. & Kerling, A. Iron deficiency in adolescent and young adult German athletes-a retrospective study. *Nutrients* **14**, 4511 (2022). 10.3390/nu14214511 PMC9657900 36364775

[32] Chambers, I. G., Willoughby, M. M., Hamza, I. & Reddi, A. R. One ring to bring them all and in the darkness bind them: The trafficking of heme without deliverers. *Biochim. Biophys. Acta Mol. Cell Res.* **1868**, 118881 (2021). 33022276 10.1016/j.bbamcr.2020.118881 PMC7756907

[33] Muckenthaler, M. U., Rivella, S., Hentze, M. W. & Galy, B. A red carpet for iron metabolism. *Cell* **168**, 344–361 (2017). 28129536 10.1016/j.cell.2016.12.034 PMC5706455

[34] Cappellini, M. D. et al. Iron deficiency across chronic inflammatory conditions: international expert opinion on definition, diagnosis, and management. *Am. J. Hematol.* **92**, 1068–1078 (2017). 28612425 10.1002/ajh.24820 PMC5599965

[35] Yang, J., Li, Q., Feng, Y. & Zeng, Y. Iron deficiency and iron deficiency anemia: potential risk factors in bone loss. *Int. J. Mol. Sci.* **24**, 6891 (2023). 10.3390/ijms24086891 PMC10138976 37108056

[36] Dziegala, M. et al. Iron deficiency as energetic insult to skeletal muscle in chronic diseases. *J. Cachexia Sarcopenia Muscle* **9**, 802–815 (2018). 30178922 10.1002/jcsm.12314 PMC6204587

[37] Vinke, J. S. J. et al. Iron deficiency is related to lower muscle mass in community-dwelling individuals and impairs myoblast proliferation. *J. Cachexia Sarcopenia Muscle* **14**, 1865–1879 (2023). 37386912 10.1002/jcsm.13277 PMC10401536

[38] Xu, W. et al. Lethal cardiomyopathy in mice lacking transferrin receptor in the heart. *Cell Rep.* **13**, 533–545 (2015). 26456827 10.1016/j.celrep.2015.09.023 PMC4618069

[39] Fleming, R. E. & Ponka, P. Iron overload in human disease. *N. Engl. J. Med.* **366**, 348–359 (2012). 22276824 10.1056/NEJMra1004967

[40] Gattermann, N. et al. The evaluation of iron deficiency and iron overload. *Dtsch. Arztebl. Int.* **118**, 847–856 (2021). 34755596 10.3238/arztebl.m2021.0290 PMC8941656

[41] Corradini, E., Buzzetti, E. & Pietrangelo, A. Genetic iron overload disorders. *Mol. Asp. Med.* **75**, 100896 (2020). 10.1016/j.mam.2020.100896 32912773

[42] Piperno, A. Classification and diagnosis of iron overload. *Haematologica* **83**, 447–455 (1998). 9658731

[43] Reeder, S. B. et al. Quantification of liver iron overload with MRI: review and guidelines from the ESGAR and SAR. *Radiology* **307**, e221856 (2023). 36809220 10.1148/radiol.221856 PMC10068892

[44] Niederau, C. et al. Survival and causes of death in cirrhotic and in noncirrhotic patients with primary hemochromatosis. *N. Engl. J. Med.* **313**, 1256–1262 (1985). 4058506 10.1056/NEJM198511143132004

[45] Handa, P. et al. Iron alters macrophage polarization status and leads to steatohepatitis and fibrogenesis. *J. Leukoc. Biol.* **105**, 1015–1026 (2019). 30835899 10.1002/JLB.3A0318-108R

[46] Sumneang, N. et al. The effects of iron overload on mitochondrial function, mitochondrial dynamics, and ferroptosis in cardiomyocytes. *Arch. Biochem. Biophys.* **680**, 108241 (2020). 31891670 10.1016/j.abb.2019.108241

[47] Vinchi, F. et al. Atherosclerosis is aggravated by iron overload and ameliorated by dietary and pharmacological iron restriction. *Eur. Heart J.* **41**, 2681–2695 (2020). 30903157 10.1093/eurheartj/ehz112

[48] Cornelissen, A. et al. New insights into the role of iron in inflammation and atherosclerosis. *EBioMedicine* **47**, 598–606 (2019). 31416722 10.1016/j.ebiom.2019.08.014 PMC6796517

[49] Ward, R. J. & Crichton, R. R. Ironing out the brain. *Met. Ions Life Sci.* **19** (2019). 10.1515/9783110527872-010 30855105

[50] Qian, Z. M. & Ke, Y. Hepcidin and its therapeutic potential in neurodegenerative disorders. *Med. Res. Rev.* **40**, 633–653 (2020). 31471929 10.1002/med.21631

[51] Garton, T., Keep, R. F., Hua, Y. & Xi, G. Brain iron overload following intracranial haemorrhage. *Stroke Vasc. Neurol.* **1**, 172–184 (2016). 28959481 10.1136/svn-2016-000042 PMC5435218

[52] Ganz, T. & Nemeth, E. Iron homeostasis in host defence and inflammation. *Nat. Rev. Immunol.* **15**, 500–510 (2015). 26160612 10.1038/nri3863 PMC4801113

[53] Stefanova, D. et al. Endogenous hepcidin and its agonist mediate resistance to selected infections by clearing non-transferrin-bound iron. *Blood* **130**, 245–257 (2017). 28465342 10.1182/blood-2017-03-772715 PMC5520472

[54] Cavezzi, A., Troiani, E. & Corrao, S. COVID-19: hemoglobin, iron, and hypoxia beyond inflammation. a narrative review. *Clin. Pr.* **10**, 1271 (2020). 10.4081/cp.2020.1271 PMC7267810 32509258

[55] Torti, S. V. et al. Iron and Cancer. *Annu Rev. Nutr.* **38**, 97–125 (2018). 30130469 10.1146/annurev-nutr-082117-051732 PMC8118195

[56] Simcox, J. A. & McClain, D. A. Iron and diabetes risk. *Cell Metab.* **17**, 329–341 (2013). 23473030 10.1016/j.cmet.2013.02.007 PMC3648340

[57] Harrison, A. V., Lorenzo, F. R. & McClain, D. A. Iron and the pathophysiology of diabetes. *Annu. Rev. Physiol.* **85**, 339–362 (2023). 36137277 10.1146/annurev-physiol-022522-102832 PMC10161568

[58] Alves, F. M. et al. Age-related changes in skeletal muscle iron homeostasis. *J. Gerontol. A Biol. Sci. Med. Sci.* **78**, 16–24 (2023). 35869751 10.1093/gerona/glac139

[59] Tsay, J. et al. Bone loss caused by iron overload in a murine model: importance of oxidative stress. *Blood* **116**, 2582–2589 (2010). 20554970 10.1182/blood-2009-12-260083 PMC2953890

[60] Zhang, H. et al. The influence of iron on bone metabolism disorders. *Osteoporos. Int.* **35**, 243–253 (2024). 37857915 10.1007/s00198-023-06937-x

[61] Ru, Q. et al. Fighting age-related orthopedic diseases: focusing on ferroptosis. *Bone Res* **11**, 12 (2023). 36854703 10.1038/s41413-023-00247-y PMC9975200

[62] Zhang, Z. et al. Discovery of benzylisothioureas as potent divalent metal transporter 1 (DMT1) inhibitors. *Bioorg. Med Chem. Lett.* **22**, 5108–5113 (2012). 22749870 10.1016/j.bmcl.2012.05.129

[63] Wetli, H. A., Buckett, P. D. & Wessling-Resnick, M. Small-molecule screening identifies the selanazal drug ebselen as a potent inhibitor of DMT1-mediated iron uptake. *Chem. Biol.* **13**, 965–972 (2006). 16984886 10.1016/j.chembiol.2006.08.005 PMC2542486

[64] Altamura, S. et al. SLN124, a GalNAc-siRNA conjugate targeting TMPRSS6, efficiently prevents iron overload in hereditary haemochromatosis type 1. *Hemasphere* **3**, e301 (2019). 31976476 10.1097/HS9.0000000000000301 PMC6924545

[65] Arezes, J. et al. Antibodies against the erythroferrone N-terminal domain prevent hepcidin suppression and ameliorate murine thalassemia. *Blood* **135**, 547–557 (2020). 31899794 10.1182/blood.2019003140 PMC7046598

[66] Gao, M. et al. Glutaminolysis and transferrin regulate ferroptosis. *Mol. Cell* **59**, 298–308 (2015). 26166707 10.1016/j.molcel.2015.06.011 PMC4506736

[67] Fang, X. et al. Loss of cardiac ferritin H facilitates cardiomyopathy via Slc7a11-mediated ferroptosis. *Circ. Res* **127**, 486–501 (2020). 32349646 10.1161/CIRCRESAHA.120.316509

[68] Geng, N. et al. Knockdown of ferroportin accelerates erastin-induced ferroptosis in neuroblastoma cells. *Eur. Rev. Med Pharm. Sci.* **22**, 3826–3836 (2018). 10.26355/eurrev_201806_15267 29949159

[69] Katsarou, A. & Pantopoulos, K. Hepcidin therapeutics. *Pharmaceuticals* **11**, 127 (2018). 10.3390/ph11040127 PMC6316648 30469435

[70] Le, Y., Zhang, Z., Wang, C. & Lu, D. Ferroptotic cell death: new regulatory mechanisms for metabolic diseases. *Endocr. Metab. Immune Disord. Drug Targets* **21**, 785–800 (2021). 32735532 10.2174/1871530320666200731175328

[71] Minotti, G. & Aust, S. D. The role of iron in oxygen radical mediated lipid peroxidation. *Chem. Biol. Interact.* **71**, 1–19 (1989). 2550151 10.1016/0009-2797(89)90087-2

[72] Doll, S. et al. ACSL4 dictates ferroptosis sensitivity by shaping cellular lipid composition. *Nat. Chem. Biol.* **13**, 91–98 (2017). 27842070 10.1038/nchembio.2239 PMC5610546

[73] Magtanong, L. et al. Exogenous monounsaturated fatty acids promote a ferroptosis-resistant cell state. *Cell Chem. Biol.* **26**, 420-432 (2019). 10.1016/j.chembiol.2018.11.016 PMC6430697 30686757

[74] Cui, J. et al. LPCAT3 is transcriptionally regulated by YAP/ZEB/EP300 and collaborates with ACSL4 and YAP to determine ferroptosis sensitivity. *Antioxid. Redox Signal.* **39**, 491–511 (2023). 37166352 10.1089/ars.2023.0237

[75] Chu, B. et al. ALOX12 is required for p53-mediated tumour suppression through a distinct ferroptosis pathway. *Nat. Cell Biol.* **21**, 579–591 (2019). 30962574 10.1038/s41556-019-0305-6 PMC6624840

[76] Forcina, G. C. & Dixon, S. J. GPX4 at the crossroads of lipid homeostasis and ferroptosis. *Proteomics* **19**, e1800311 (2019). 30888116 10.1002/pmic.201800311

[77] Forman, H. J., Zhang, H. & Rinna, A. Glutathione: overview of its protective roles, measurement, and biosynthesis. *Mol. Asp. Med.* **30**, 1–12 (2009). 10.1016/j.mam.2008.08.006 PMC2696075 18796312

[78] Yang, W. S. et al. Regulation of ferroptotic cancer cell death by GPX4. *Cell* **156**, 317–331 (2014). 24439385 10.1016/j.cell.2013.12.010 PMC4076414

[79] Chen, Z., Putt, D. A. & Lash, L. H. Enrichment and functional reconstitution of glutathione transport activity from rabbit kidney mitochondria: further evidence for the role of the dicarboxylate and 2-oxoglutarate carriers in mitochondrial glutathione transport. *Arch. Biochem. Biophys.* **373**, 193–202 (2000). 10620338 10.1006/abbi.1999.1527

[80] Seiler, A. et al. Glutathione peroxidase 4 senses and translates oxidative stress into 12/15-lipoxygenase dependent- and AIF-mediated cell death. *Cell Metab.* **8**, 237–248 (2008). 18762024 10.1016/j.cmet.2008.07.005

[81] Doll, S. et al. FSP1 is a glutathione-independent ferroptosis suppressor. *Nature* **575**, 693–698 (2019). 31634899 10.1038/s41586-019-1707-0

[82] Bersuker, K. et al. The CoQ oxidoreductase FSP1 acts parallel to GPX4 to inhibit ferroptosis. *Nature* **575**, 688–692 (2019). 31634900 10.1038/s41586-019-1705-2 PMC6883167

[83] Liang, D. et al. Ferroptosis surveillance independent of GPX4 and differentially regulated by sex hormones. *Cell* **186**, 2748–2764 e22 (2023). 37267948 10.1016/j.cell.2023.05.003 PMC10330611

[84] Cronin, S. J. F. et al. The metabolite BH4 controls T cell proliferation in autoimmunity and cancer. *Nature* **563**, 564–568 (2018). 30405245 10.1038/s41586-018-0701-2 PMC6438708

[85] Sun, X. et al. Activation of the p62-Keap1-NRF2 pathway protects against ferroptosis in hepatocellular carcinoma cells. *Hepatology* **63**, 173–184 (2016). 26403645 10.1002/hep.28251 PMC4688087

[86] Liu, Y. et al. The diversified role of mitochondria in ferroptosis in cancer. *Cell Death Dis.* **14**, 519 (2023). 37580393 10.1038/s41419-023-06045-y PMC10425449

[87] Quinlan, C. L. et al. Mitochondrial complex II can generate reactive oxygen species at high rates in both the forward and reverse reactions. *J. Biol. Chem.* **287**, 27255–27264 (2012). 22689576 10.1074/jbc.M112.374629 PMC3411067

[88] Zou, Y. et al. A GPX4-dependent cancer cell state underlies the clear-cell morphology and confers sensitivity to ferroptosis. *Nat. Commun.* **10**, 1617 (2019). 30962421 10.1038/s41467-019-09277-9 PMC6453886

[89] Schnurr, K., Borchert, A. & Kuhn, H. Inverse regulation of lipid-peroxidizing and hydroperoxyl lipid-reducing enzymes by interleukins 4 and 13. *FASEB J.* **13**, 143–154 (1999). 9872939 10.1096/fasebj.13.1.143

[90] Lee, J., You, J. H., Kim, M. S. & Roh, J. L. Epigenetic reprogramming of epithelial-mesenchymal transition promotes ferroptosis of head and neck cancer. *Redox Biol.* **37**, 101697 (2020). 32896720 10.1016/j.redox.2020.101697 PMC7484553

[91] Pastushenko, I. et al. Identification of the tumour transition states occurring during EMT. *Nature* **556**, 463–468 (2018). 29670281 10.1038/s41586-018-0040-3

[92] Na, T. Y., Schecterson, L., Mendonsa, A. M. & Gumbiner, B. M. The functional activity of E-cadherin controls tumor cell metastasis at multiple steps. *Proc. Natl. Acad. Sci. USA* **117**, 5931–5937 (2020). 32127478 10.1073/pnas.1918167117 PMC7084067

[93] Lu, H. et al. Reflux conditions induce E-cadherin cleavage and EMT via APE1 redox function in oesophageal adenocarcinoma. *Gut* **73**, 47–62 (2023). 37734913 10.1136/gutjnl-2023-329455 PMC10872865

[94] Wenz, C. et al. Cell-cell contacts protect against t-BuOOH-induced cellular damage and ferroptosis in vitro. *Arch. Toxicol.* **93**, 1265–1279 (2019). 30798349 10.1007/s00204-019-02413-w

[95] Ren, Y. et al. Ferroptosis and EMT: key targets for combating cancer progression and therapy resistance. *Cell Mol. Life Sci.* **80**, 263 (2023). 37598126 10.1007/s00018-023-04907-4 PMC10439860

[96] Wang, M. et al. Gambogenic acid induces ferroptosis in melanoma cells undergoing epithelial-to-mesenchymal transition. *Toxicol. Appl Pharm.* **401**, 115110 (2020). 10.1016/j.taap.2020.115110 32533954

[97] Sun, L. et al. Lipid peroxidation, GSH depletion, and SLC7A11 inhibition are common causes of EMT and ferroptosis in A549 cells, but different in specific mechanisms. *DNA Cell Biol.* **40**, 172–183 (2021). 33351681 10.1089/dna.2020.5730

[98] Cen, J. et al. Hsa_circ_0057105 modulates a balance of epithelial-mesenchymal transition and ferroptosis vulnerability in renal cell carcinoma. *Clin. Transl. Med.* **13**, e1339 (2023). 37496319 10.1002/ctm2.1339 PMC10372385

[99] Takaoka, Y. et al. Mitochondrial pyruvate carrier 1 expression controls cancer epithelial-mesenchymal transition and radioresistance. *Cancer Sci.* **110**, 1331–1339 (2019). 30801869 10.1111/cas.13980 PMC6447954

[100] Cui, J. et al. A novel KDM5A/MPC-1 signaling pathway promotes pancreatic cancer progression via redirecting mitochondrial pyruvate metabolism. *Oncogene* **39**, 1140–1151 (2020). 31641207 10.1038/s41388-019-1051-8

[101] You, J. H., Lee, J. & Roh, J. L. Mitochondrial pyruvate carrier 1 regulates ferroptosis in drug-tolerant persister head and neck cancer cells via epithelial-mesenchymal transition. *Cancer Lett.* **507**, 40–54 (2021). 33741422 10.1016/j.canlet.2021.03.013

[102] De Craene, B. & Berx, G. Regulatory networks defining EMT during cancer initiation and progression. *Nat. Rev. Cancer* **13**, 97–110 (2013). 23344542 10.1038/nrc3447

[103] Wang, Y. et al. Histone demethylase KDM3B protects against ferroptosis by upregulating SLC7A11. *FEBS Open Bio.* **10**, 637–643 (2020). 32107878 10.1002/2211-5463.12823 PMC7137800

[104] Salvatori, I., Valle, C., Ferri, A. & Carri, M. T. SIRT3 and mitochondrial metabolism in neurodegenerative diseases. *Neurochem. Int.* **109**, 184–192 (2017). 28449871 10.1016/j.neuint.2017.04.012

[105] Liu, L. et al. SIRT3 inhibits gallbladder cancer by induction of AKT-dependent ferroptosis and blockade of epithelial-mesenchymal transition. *Cancer Lett.* **510**, 93–104 (2021). 33872694 10.1016/j.canlet.2021.04.007

[106] Zhang, H., Zhou, L., Davies, K. J. A. & Forman, H. J. Silencing Bach1 alters aging-related changes in the expression of Nrf2-regulated genes in primary human bronchial epithelial cells. *Arch. Biochem. Biophys.* **672**, 108074 (2019). 31422075 10.1016/j.abb.2019.108074

[107] Nishizawa, H. et al. Ferroptosis is controlled by the coordinated transcriptional regulation of glutathione and labile iron metabolism by the transcription factor BACH1. *J. Biol. Chem.* **295**, 69–82 (2020). 31740582 10.1074/jbc.RA119.009548 PMC6952604

[108] Sato, M. et al. BACH1 promotes pancreatic cancer metastasis by repressing epithelial genes and enhancing epithelial-mesenchymal transition. *Cancer Res.* **80**, 1279–1292 (2020). 31919242 10.1158/0008-5472.CAN-18-4099

[109] Nishizawa, H., Yamanaka, M. & Igarashi, K. Ferroptosis: regulation by competition between NRF2 and BACH1 and propagation of the death signal. *FEBS J.* **290**, 1688–1704 (2023). 35107212 10.1111/febs.16382

[110] Chen, P. et al. Combinative treatment of beta-elemene and cetuximab is sensitive to KRAS mutant colorectal cancer cells by inducing ferroptosis and inhibiting epithelial-mesenchymal transformation. *Theranostics* **10**, 5107–5119 (2020). 32308771 10.7150/thno.44705 PMC7163451

[111] Sung, H. et al. Global cancer statistics 2020: GLOBOCAN estimates of incidence and mortality worldwide for 36 cancers in 185 countries. *CA Cancer J. Clin.* **71**, 209–249 (2021). 33538338 10.3322/caac.21660

[112] Siegel, R. L., Miller, K. D., Wagle, N. S. & Jemal, A. Cancer statistics, 2023. *CA Cancer J. Clin.* **73**, 17–48 (2023). 36633525 10.3322/caac.21763

[113] Schmitt, A. M. & Chang, H. Y. Long noncoding RNAs in cancer pathways. *Cancer Cell* **29**, 452–463 (2016). 27070700 10.1016/j.ccell.2016.03.010 PMC4831138

[114] Xi, S. et al. Downregulation of N6-methyladenosine-modified LINC00641 promotes EMT, but provides a ferroptotic vulnerability in lung cancer. *Cell Death Dis.* **14**, 359 (2023). 37311754 10.1038/s41419-023-05880-3 PMC10264399

[115] Guan, D. et al. The DpdtbA induced EMT inhibition in gastric cancer cell lines was through ferritinophagy-mediated activation of p53 and PHD2/hif-1alpha pathway. *J. Inorg. Biochem.* **218**, 111413 (2021). 33713969 10.1016/j.jinorgbio.2021.111413

[116] Wang, S. et al. Upregulation of ARNTL2 is associated with poor survival and immune infiltration in clear cell renal cell carcinoma. *Cancer Cell Int.* **21**, 341 (2021). 34217271 10.1186/s12935-021-02046-z PMC8255002

[117] Wang, X. et al. ARNTL2 is a prognostic biomarker and correlates with immune cell infiltration in triple-negative breast cancer. *Pharmacogenomics Pers. Med.* **14**, 1425–1440 (2021). 10.2147/PGPM.S331431 PMC8591114 34785930

[118] Zhang, H. et al. ARNTL2 is an indicator of poor prognosis, promotes epithelial-to-mesenchymal transition and inhibits ferroptosis in lung adenocarcinoma. *Transl. Oncol.* **26**, 101562 (2022). 36228410 10.1016/j.tranon.2022.101562 PMC9563212

[119] Kuroki, L. & Guntupalli, S. R. Treatment of epithelial ovarian cancer. *BMJ* **371**, m3773 (2020). 33168565 10.1136/bmj.m3773

[120] Liu, Y. et al. Agrimonolide inhibits cancer progression and induces ferroptosis and apoptosis by targeting SCD1 in ovarian cancer cells. *Phytomedicine* **101**, 154102 (2022). 35526323 10.1016/j.phymed.2022.154102

[121] Wang, H. et al. Crystal structure of human stearoyl-coenzyme A desaturase in complex with substrate. *Nat. Struct. Mol. Biol.* **22**, 581–585 (2015). 26098317 10.1038/nsmb.3049

[122] Bohnsack, M. T. & Sloan, K. E. The mitochondrial epitranscriptome: the roles of RNA modifications in mitochondrial translation and human disease. *Cell Mol. Life Sci.* **75**, 241–260 (2018). 28752201 10.1007/s00018-017-2598-6 PMC5756263

[123] Delaunay, S. & Frye, M. RNA modifications regulating cell fate in cancer. *Nat. Cell Biol.* **21**, 552–559 (2019). 31048770 10.1038/s41556-019-0319-0

[124] Shi, Z. et al. Mettl17, a regulator of mitochondrial ribosomal RNA modifications, is required for the translation of mitochondrial coding genes. *FASEB J.* **33**, 13040–13050 (2019). 31487196 10.1096/fj.201901331R

[125] Li, H. et al. METTL17 coordinates ferroptosis and tumorigenesis by regulating mitochondrial translation in colorectal cancer. *Redox Biol.* **71**, 103087 (2024). 38377789 10.1016/j.redox.2024.103087 PMC10884776

[126] Wei, R. et al. Tagitinin C induces ferroptosis through PERK-Nrf2-HO-1 signaling pathway in colorectal cancer cells. *Int J. Biol. Sci.* **17**, 2703–2717 (2021). 34345202 10.7150/ijbs.59404 PMC8326123

[127] Siegel, R. L., Miller, K. D., Fuchs, H. E. & Jemal, A. Cancer statistics, 2022. *CA Cancer J. Clin.* **72**, 7–33 (2022). 35020204 10.3322/caac.21708

[128] Makker, V. et al. Endometrial cancer. *Nat. Rev. Dis. Prim.* **7**, 88 (2021). 34887451 10.1038/s41572-021-00324-8 PMC9421940

[129] Miao, X. & Zhang, N. Role of RBM3 in the regulation of cell proliferation in hepatocellular carcinoma. *Exp. Mol. Pathol.* **117**, 104546 (2020). 32976820 10.1016/j.yexmp.2020.104546

[130] Melling, N. et al. Prevalence and clinical significance of RBM3 immunostaining in non-small cell lung cancers. *J. Cancer Res. Clin. Oncol.* **145**, 873–879 (2019). 30758670 10.1007/s00432-019-02850-1 PMC11810404

[131] Wang, Z. et al. Sodium butyrate induces ferroptosis in endometrial cancer cells via the RBM3/SLC7A11 axis. *Apoptosis* **28**, 1168–1183 (2023). 37170022 10.1007/s10495-023-01850-4

[132] Smyth, E. C. et al. Gastric cancer. *Lancet* **396**, 635–648 (2020). 32861308 10.1016/S0140-6736(20)31288-5

[133] Saha, G., Roy, S., Basu, M. & Ghosh, M. K. USP7 - a crucial regulator of cancer hallmarks. *Biochim. Biophys. Acta Rev. Cancer* **1878**, 188903 (2023). 37127084 10.1016/j.bbcan.2023.188903

[134] Guan, X. et al. Blocking Ubiquitin-Specific Protease 7 Induces Ferroptosis in Gastric Cancer via Targeting Stearoyl-CoA desaturase. *Adv. Sci.* **11**, e2307899 (2024). 10.1002/advs.202307899 PMC11095140 38460164

[135] Whiteley, A. E., Price, T. T., Cantelli, G. & Sipkins, D. A. Leukaemia: a model metastatic disease. *Nat. Rev. Cancer* **21**, 461–475 (2021). 33953370 10.1038/s41568-021-00355-z PMC8722462

[136] Du, J. et al. DHA inhibits proliferation and induces ferroptosis of leukemia cells through autophagy dependent degradation of ferritin. *Free Radic. Biol. Med.* **131**, 356–369 (2019). 30557609 10.1016/j.freeradbiomed.2018.12.011

[137] Jiang, J. et al. Let‑7d inhibits colorectal cancer cell proliferation through the CST1/p65 pathway. *Int J. Oncol.* **53**, 781–790 (2018). 29845224 10.3892/ijo.2018.4419

[138] Li, D. et al. CST1 inhibits ferroptosis and promotes gastric cancer metastasis by regulating GPX4 protein stability via OTUB1. *Oncogene* **42**, 83–98 (2023). 36369321 10.1038/s41388-022-02537-x PMC9816059

[139] Gu, J. et al. Astragalus mongholicus Bunge-Curcuma aromatica Salisb. suppresses growth and metastasis of colorectal cancer cells by inhibiting M2 macrophage polarization via a Sp1/ZFAS1/miR-153-3p/CCR5 regulatory axis. *Cell Biol. Toxicol.* **38**, 679–697 (2022). 35072892 10.1007/s10565-021-09679-w

[140] Yi, C. et al. Ferroptosis-dependent breast cancer cell-derived exosomes inhibit migration and invasion of breast cancer cells by suppressing M2 macrophage polarization. *PeerJ* **11**, e15060 (2023). 36949762 10.7717/peerj.15060 PMC10026718

[141] Chen, S., Zhou, L. & Wang, Y. ALKBH5-mediated m(6)A demethylation of lncRNA PVT1 plays an oncogenic role in osteosarcoma. *Cancer Cell Int.* **20**, 34 (2020). 32021563 10.1186/s12935-020-1105-6 PMC6993345

[142] Li, Q. et al. WTAP facilitates progression of endometrial cancer via CAV-1/NF-kappaB axis. *Cell Biol. Int.* **45**, 1269–1277 (2021). 33559954 10.1002/cbin.11570

[143] Wang, C. Q. et al. Upregulated WTAP expression appears to both promote breast cancer growth and inhibit lymph node metastasis. *Sci. Rep.* **12**, 1023 (2022). 35046505 10.1038/s41598-022-05035-y PMC8770795

[144] Liu, J. et al. NUPR1 is a critical repressor of ferroptosis. *Nat. Commun.* **12**, 647 (2021). 33510144 10.1038/s41467-021-20904-2 PMC7843652

[145] Tan, M. et al. WTAP mediates NUPR1 regulation of LCN2 through m(6)A modification to influence ferroptosis, thereby promoting breast cancer proliferation, migration and invasion. *Biochem. Genet.* **62**, 876–891 (2024). 37477758 10.1007/s10528-023-10423-8

[146] Faisham, W. I. et al. Prognostic factors and survival rate of osteosarcoma: a single-institution study. *Asia Pac. J. Clin. Oncol.* **13**, e104–e110 (2017). 25870979 10.1111/ajco.12346

[147] Jiang, M. et al. Exosome-mediated miR-144-3p promotes ferroptosis to inhibit osteosarcoma proliferation, migration, and invasion through regulating ZEB1. *Mol. Cancer* **22**, 113 (2023). 37461104 10.1186/s12943-023-01804-z PMC10351131

[148] Wang, J. et al. Eight proteins play critical roles in RCC with bone metastasis via mitochondrial dysfunction. *Clin. Exp. Metastasis* **32**, 605–622 (2015). 26115722 10.1007/s10585-015-9731-4 PMC4503866

[149] Rane, M. J., Zhao, Y. & Cai, L. Krupsilonppel-like factors (KLFs) in renal physiology and disease. *EBio Med.* **40**, 743–750 (2019). 10.1016/j.ebiom.2019.01.021 PMC6414320 30662001

[150] Lu, Y. et al. KLF2 inhibits cancer cell migration and invasion by regulating ferroptosis through GPX4 in clear cell renal cell carcinoma. *Cancer Lett.* **522**, 1–13 (2021). 34520818 10.1016/j.canlet.2021.09.014

[151] Siegel, R. L., Miller, K. D., Fuchs, H. E. & Jemal, A. Cancer statistics, 2021. *CA Cancer J. Clin.* **71**, 7–33 (2021). 33433946 10.3322/caac.21654

[152] Chang, J. W. et al. An integrative model for alternative polyadenylation, IntMAP, delineates mTOR-modulated endoplasmic reticulum stress response. *Nucleic Acids Res* **46**, 5996–6008 (2018). 29733382 10.1093/nar/gky340 PMC6158760

[153] Zhang, X. et al. CEBPG suppresses ferroptosis through transcriptional control of SLC7A11 in ovarian cancer. *J. Transl. Med* **21**, 334 (2023). 37210575 10.1186/s12967-023-04136-0 PMC10199564

[154] Modest, D. P. et al. Outcome according to KRAS-, NRAS- and BRAF-mutation as well as KRAS mutation variants: pooled analysis of five randomized trials in metastatic colorectal cancer by the AIO colorectal cancer study group. *Ann. Oncol.* **27**, 1746–1753 (2016). 27358379 10.1093/annonc/mdw261 PMC4999563

[155] Miao, Q. et al. Erianin inhibits the growth and metastasis through autophagy-dependent ferroptosis in KRAS(G13D) colorectal cancer. *Free Radic. Biol. Med.* **204**, 301–312 (2023). 37217090 10.1016/j.freeradbiomed.2023.05.008

[156] Arden, K. C., Viars, C. S., Fu, K. & Rozen, R. Localization of short/branched chain acyl-CoA dehydrogenase (ACADSB) to human chromosome 10. *Genomics* **25**, 743–745 (1995). 7759115 10.1016/0888-7543(95)80023-f

[157] Lu, D. et al. ACADSB regulates ferroptosis and affects the migration, invasion, and proliferation of colorectal cancer cells. *Cell Biol. Int.* **44**, 2334–2343 (2020). 32776663 10.1002/cbin.11443

[158] Groot, V. P. et al. Patterns, Timing, and predictors of recurrence following pancreatectomy for pancreatic ductal adenocarcinoma. *Ann. Surg.* **267**, 936–945 (2018). 28338509 10.1097/SLA.0000000000002234

[159] Sancho, P., Barneda, D. & Heeschen, C. Hallmarks of cancer stem cell metabolism. *Br. J. Cancer* **114**, 1305–1312 (2016). 27219018 10.1038/bjc.2016.152 PMC4984474

[160] Baughman, J. M. et al. Integrative genomics identifies MCU as an essential component of the mitochondrial calcium uniporter. *Nature* **476**, 341–345 (2011). 21685886 10.1038/nature10234 PMC3486726

[161] De Stefani, D., Rizzuto, R. & Pozzan, T. Enjoy the trip: calcium in mitochondria back and forth. *Annu. Rev. Biochem.* **85**, 161–192 (2016). 27145841 10.1146/annurev-biochem-060614-034216

[162] Pan, X. et al. The physiological role of mitochondrial calcium revealed by mice lacking the mitochondrial calcium uniporter. *Nat. Cell Biol.* **15**, 1464–1472 (2013). 24212091 10.1038/ncb2868 PMC3852190

[163] Wang, X. et al. Mitochondrial calcium uniporter drives metastasis and confers a targetable cystine dependency in pancreatic cancer. *Cancer Res.* **82**, 2254–2268 (2022). 35413105 10.1158/0008-5472.CAN-21-3230 PMC9203979

[164] Borhani, S., Borhani, R. & Kajdacsy-Balla, A. Artificial intelligence: a promising frontier in bladder cancer diagnosis and outcome prediction. *Crit. Rev. Oncol. Hematol.* **171**, 103601 (2022). 35065220 10.1016/j.critrevonc.2022.103601

[165] Patel, V. G., Oh, W. K. & Galsky, M. D. Treatment of muscle-invasive and advanced bladder cancer in 2020. *CA Cancer J. Clin.* **70**, 404–423 (2020). 32767764 10.3322/caac.21631

[166] Lager, T. W. et al. Cell surface GRP78 and Dermcidin cooperate to regulate breast cancer cell migration through Wnt signaling. *Oncogene* **40**, 4050–4059 (2021). 33981001 10.1038/s41388-021-01821-6 PMC8197743

[167] Samanta, S. et al. The hydroxyquinoline analogue YUM70 inhibits GRP78 to induce er stress-mediated apoptosis in pancreatic cancer. *Cancer Res.* **81**, 1883–1895 (2021). 33531374 10.1158/0008-5472.CAN-20-1540 PMC8137563

[168] Kim, S. Y. et al. HSPA5 negatively regulates lysosomal activity through ubiquitination of MUL1 in head and neck cancer. *Autophagy* **14**, 385–403 (2018). 29260979 10.1080/15548627.2017.1414126 PMC5915028

[169] Wang, Q. et al. HSPA5 promotes the proliferation, metastasis and regulates ferroptosis of bladder cancer. *Int. J. Mol. Sci.* **24**, 5144 (2023). 10.3390/ijms24065144 PMC10048805 36982218

[170] Ketteler, J. & Klein, D. Caveolin-1, cancer and therapy resistance. *Int. J. Cancer* **143**, 2092–2104 (2018). 29524224 10.1002/ijc.31369

[171] Lu, T. et al. Caveolin-1 promotes cancer progression via inhibiting ferroptosis in head and neck squamous cell carcinoma. *J. Oral. Pathol. Med.* **51**, 52–62 (2022). 34874578 10.1111/jop.13267 PMC9300096

[172] Florek, M. et al. Prominin-2 is a cholesterol-binding protein associated with apical and basolateral plasmalemmal protrusions in polarized epithelial cells and released into urine. *Cell Tissue Res.* **328**, 31–47 (2007). 17109118 10.1007/s00441-006-0324-z

[173] Dowland, S. N. et al. Prominin-2 prevents the formation of caveolae in normal and ovarian hyperstimulated pregnancy. *Reprod. Sci.* **25**, 1231–1242 (2018). 29113580 10.1177/1933719117737842

[174] Singh, R. D. et al. Prominin-2 expression increases protrusions, decreases caveolae and inhibits Cdc42 dependent fluid phase endocytosis. *Biochem. Biophys. Res. Commun.* **434**, 466–472 (2013). 23583380 10.1016/j.bbrc.2013.03.097 PMC3659420

[175] Paris, J. et al. PROM2 overexpression induces metastatic potential through epithelial-to-mesenchymal transition and ferroptosis resistance in human cancers. *Clin. Transl. Med.* **14**, e1632 (2024). 38515278 10.1002/ctm2.1632 PMC10958126

[176] Zhang, C. et al. Ferroptosis in cancer therapy: a novel approach to reversing drug resistance. *Mol. Cancer* **21**, 47 (2022). 35151318 10.1186/s12943-022-01530-y PMC8840702

[177] Chen, T. C. et al. AR ubiquitination induced by the curcumin analog suppresses growth of temozolomide-resistant glioblastoma through disrupting GPX4-Mediated redox homeostasis. *Redox Biol.* **30**, 101413 (2020). 31896509 10.1016/j.redox.2019.101413 PMC6940696

[178] de Souza, I. et al. High levels of NRF2 sensitize temozolomide-resistant glioblastoma cells to ferroptosis via ABCC1/MRP1 upregulation. *Cell Death Dis.* **13**, 591 (2022). 35803910 10.1038/s41419-022-05044-9 PMC9270336

[179] Lou, J. S. et al. Ginkgetin derived from Ginkgo biloba leaves enhances the therapeutic effect of cisplatin via ferroptosis-mediated disruption of the Nrf2/HO-1 axis in EGFR wild-type non-small-cell lung cancer. *Phytomedicine* **80**, 153370 (2021). 33113504 10.1016/j.phymed.2020.153370

[180] Liang, Z. et al. Cisplatin synergizes with PRLX93936 to induce ferroptosis in non-small cell lung cancer cells. *Biochem. Biophys. Res. Commun.* **569**, 79–85 (2021). 34237431 10.1016/j.bbrc.2021.06.088

[181] Cui, Z., Li, D., Zhao, J. & Chen, K. Falnidamol and cisplatin combinational treatment inhibits non-small cell lung cancer (NSCLC) by targeting DUSP26-mediated signal pathways. *Free Radic. Biol. Med.* **183**, 106–124 (2022). 35278641 10.1016/j.freeradbiomed.2022.03.003

[182] Gentile, D. et al. Surgical treatment of hepatocholangiocarcinoma: a systematic review. *Liver Cancer* **9**, 15–27 (2020). 32071906 10.1159/000503719 PMC7024854

[183] Gigante, E. et al. Systemic treatments with tyrosine kinase inhibitor and platinum-based chemotherapy in patients with unresectable or metastatic hepatocholangiocarcinoma. *Liver Cancer* **11**, 460–473 (2022). 36158591 10.1159/000525488 PMC9485952

[184] Shang, Y. et al. Pharmaceutical immunoglobulin G impairs anti-carcinoma activity of oxaliplatin in colon cancer cells. *Br. J. Cancer* **124**, 1411–1420 (2021). 33558709 10.1038/s41416-021-01272-6 PMC8039037

[185] Lin, Z. et al. m(6)A-mediated lnc-OXAR promotes oxaliplatin resistance by enhancing Ku70 stability in non-alcoholic steatohepatitis-related hepatocellular carcinoma. *J. Exp. Clin. Cancer Res.* **43**, 206 (2024). 39054531 10.1186/s13046-024-03134-4 PMC11271202

[186] Tang, J. et al. ATR-dependent ubiquitin-specific protease 20 phosphorylation confers oxaliplatin and ferroptosis resistance. *MedComm* **4**, e463 (2023). 38124786 10.1002/mco2.463 PMC10732327

[187] Du, J. et al. DHA exhibits synergistic therapeutic efficacy with cisplatin to induce ferroptosis in pancreatic ductal adenocarcinoma via modulation of iron metabolism. *Cell Death Dis.* **12**, 705 (2021). 34262021 10.1038/s41419-021-03996-y PMC8280115

[188] Roh, J. L. et al. Induction of ferroptotic cell death for overcoming cisplatin resistance of head and neck cancer. *Cancer Lett.* **381**, 96–103 (2016). 27477897 10.1016/j.canlet.2016.07.035

[189] Chaudhary, N. et al. Lipocalin 2 expression promotes tumor progression and therapy resistance by inhibiting ferroptosis in colorectal cancer. *Int. J. Cancer* **149**, 1495–1511 (2021). 34146401 10.1002/ijc.33711

[190] Zeng, K. et al. Inhibition of CDK1 overcomes oxaliplatin resistance by regulating ACSL4-mediated ferroptosis in colorectal cancer. *Adv. Sci.* **10**, e2301088 (2023). 10.1002/advs.202301088 PMC10477855 37428466

[191] Turcu, A. L. et al. DMT1 inhibitors kill cancer stem cells by blocking lysosomal iron translocation. *Chemistry* **26**, 7369–7373 (2020). 32083771 10.1002/chem.202000159

[192] Ouyang, S. et al. Inhibition of STAT3-ferroptosis negative regulatory axis suppresses tumor growth and alleviates chemoresistance in gastric cancer. *Redox Biol.* **52**, 102317 (2022). 35483272 10.1016/j.redox.2022.102317 PMC9108091

[193] Fu, D., Wang, C., Yu, L. & Yu, R. Induction of ferroptosis by ATF3 elevation alleviates cisplatin resistance in gastric cancer by restraining Nrf2/Keap1/xCT signaling. *Cell Mol. Biol. Lett.* **26**, 26 (2021). 34098867 10.1186/s11658-021-00271-y PMC8186082

[194] Ajani, J. A. et al. A phase III trial comparing oral S-1/cisplatin and intravenous 5-fluorouracil/cisplatin in patients with untreated diffuse gastric cancer. *Ann. Oncol.* **28**, 2142–2148 (2017). 28911091 10.1093/annonc/mdx275

[195] Kang, Y. K. et al. S-1 plus leucovorin and oxaliplatin versus S-1 plus cisplatin as first-line therapy in patients with advanced gastric cancer (SOLAR): a randomised, open-label, phase 3 trial. *Lancet Oncol.* **21**, 1045–1056 (2020). 32682457 10.1016/S1470-2045(20)30315-6

[196] Zhan, T., Rindtorff, N. & Boutros, M. Wnt signaling in cancer. *Oncogene* **36**, 1461–1473 (2017). 27617575 10.1038/onc.2016.304 PMC5357762

[197] Wang, Y. et al. Wnt/beta-catenin signaling confers ferroptosis resistance by targeting GPX4 in gastric cancer. *Cell Death Differ.* **29**, 2190–2202 (2022). 35534546 10.1038/s41418-022-01008-w PMC9613693

[198] Ren, J. et al. Carcinoma-associated fibroblasts promote the stemness and chemoresistance of colorectal cancer by transferring exosomal lncRNA H19. *Theranostics* **8**, 3932–3948 (2018). 30083271 10.7150/thno.25541 PMC6071523

[199] Zhou, L., Li, J., Tang, Y. & Yang, M. Exosomal LncRNA LINC00659 transferred from cancer-associated fibroblasts promotes colorectal cancer cell progression via miR-342-3p/ANXA2 axis. *J. Transl. Med.* **19**, 8 (2021). 33407563 10.1186/s12967-020-02648-7 PMC7789760

[200] Qu, X. et al. Loss of cancer-associated fibroblast-derived exosomal DACT3-AS1 promotes malignant transformation and ferroptosis-mediated oxaliplatin resistance in gastric cancer. *Drug Resist. Updat.* **68**, 100936 (2023). 36764075 10.1016/j.drup.2023.100936

[201] Zhang, H. et al. CAF secreted miR-522 suppresses ferroptosis and promotes acquired chemo-resistance in gastric cancer. *Mol. Cancer* **19**, 43 (2020). 32106859 10.1186/s12943-020-01168-8 PMC7045485

[202] Qi, R. et al. Cancer-associated fibroblasts suppress ferroptosis and induce gemcitabine resistance in pancreatic cancer cells by secreting exosome-derived ACSL4-targeting miRNAs. *Drug Resist. Updat.* **68**, 100960 (2023). 37003125 10.1016/j.drup.2023.100960

[203] Zhang, Q. et al. Metabolic reprogramming of ovarian cancer involves ACSL1-mediated metastasis stimulation through upregulated protein myristoylation. *Oncogene* **40**, 97–111 (2021). 33082557 10.1038/s41388-020-01516-4

[204] Tan, Y. et al. Metabolic reprogramming from glycolysis to fatty acid uptake and beta-oxidation in platinum-resistant cancer cells. *Nat. Commun.* **13**, 4554 (2022). 35931676 10.1038/s41467-022-32101-w PMC9356138

[205] Zhang, Q. et al. ACSL1-induced ferroptosis and platinum resistance in ovarian cancer by increasing FSP1 N-myristylation and stability. *Cell Death Discov.* **9**, 83 (2023). 36882396 10.1038/s41420-023-01385-2 PMC9992462

[206] Guo, C. et al. Pharmacological properties and derivatives of shikonin-a review in recent years. *Pharm. Res.* **149**, 104463 (2019). 10.1016/j.phrs.2019.104463 31553936

[207] Ni, M. et al. Shikonin and cisplatin synergistically overcome cisplatin resistance of ovarian cancer by inducing ferroptosis via upregulation of HMOX1 to promote Fe(2+) accumulation. *Phytomedicine* **112**, 154701 (2023). 36773431 10.1016/j.phymed.2023.154701

[208] Wang, Y. et al. Frizzled-7 identifies platinum-tolerant ovarian cancer cells susceptible to ferroptosis. *Cancer Res.* **81**, 384–399 (2021). 33172933 10.1158/0008-5472.CAN-20-1488 PMC7855035

[209] Khan, S. A., Tavolari, S. & Brandi, G. Cholangiocarcinoma: epidemiology and risk factors. *Liver Int.* **39**, 19–31 (2019). 30851228 10.1111/liv.14095

[210] Duan, S. et al. Loss of FBXO31-mediated degradation of DUSP6 dysregulates ERK and PI3K-AKT signaling and promotes prostate tumorigenesis. *Cell Rep.* **37**, 109870 (2021). 34686346 10.1016/j.celrep.2021.109870 PMC8577224

[211] Zou, S. et al. FBXO31 suppresses gastric cancer EMT by targeting Snail1 for proteasomal degradation. *Mol. Cancer Res.* **16**, 286–295 (2018). 29117943 10.1158/1541-7786.MCR-17-0432

[212] Zhu, Z. et al. FBXO31 sensitizes cancer stem cells-like cells to cisplatin by promoting ferroptosis and facilitating proteasomal degradation of GPX4 in cholangiocarcinoma. *Liver Int.* **42**, 2871–2888 (2022). 36269678 10.1111/liv.15462

[213] Wu, C. C. et al. Tumor sidedness and efficacy of first-line therapy in patients with RAS/BRAF wild-type metastatic colorectal cancer: a network meta-analysis. *Crit. Rev. Oncol. Hematol.* **145**, 102823 (2020). 31783291 10.1016/j.critrevonc.2019.102823

[214] Ye, Z. et al. Abrogation of ARF6 promotes RSL3-induced ferroptosis and mitigates gemcitabine resistance in pancreatic cancer cells. *Am. J. Cancer Res.* **10**, 1182–1193 (2020). 32368394 PMC7191101

[215] Pardieu, B. et al. Cystine uptake inhibition potentiates front-line therapies in acute myeloid leukemia. *Leukemia* **36**, 1585–1595 (2022). 35474100 10.1038/s41375-022-01573-6 PMC12860451

[216] Han, L., Li, L. & Wu, G. Induction of ferroptosis by carnosic acid-mediated inactivation of Nrf2/HO-1 potentiates cisplatin responsiveness in OSCC cells. *Mol. Cell Probes* **64**, 101821 (2022). 35490795 10.1016/j.mcp.2022.101821

[217] Zheng, J. & Conrad, M. The metabolic underpinnings of ferroptosis. *Cell Metab.* **32**, 920–937 (2020). 33217331 10.1016/j.cmet.2020.10.011

[218] Wang, H., Liu, C., Zhao, Y. & Gao, G. Mitochondria regulation in ferroptosis. *Eur. J. Cell Biol.* **99**, 151058 (2020). 31810634 10.1016/j.ejcb.2019.151058

[219] Sedlackova, L. & Korolchuk, V. I. Mitochondrial quality control as a key determinant of cell survival. *Biochim. Biophys. Acta Mol. Cell Res.* **1866**, 575–587 (2019). 30594496 10.1016/j.bbamcr.2018.12.012

[220] Hao, S. et al. Metabolic networks in ferroptosis. *Oncol. Lett.* **15**, 5405–5411 (2018). 29556292 10.3892/ol.2018.8066 PMC5844144

[221] Salaye, L. et al. A low iron diet protects from steatohepatitis in a mouse model. *Nutrients* **11**, 2172 (2019). 10.3390/nu11092172 PMC6769937 31510077

[222] Backe, M. B. et al. Iron regulation of pancreatic beta-cell functions and oxidative stress. *Annu. Rev. Nutr.* **36**, 241–273 (2016). 27146016 10.1146/annurev-nutr-071715-050939

[223] Rehman, K. & Akash, M. S. H. Mechanism of generation of oxidative stress and pathophysiology of type 2 diabetes mellitus: how are they interlinked? *J. Cell Biochem.* **118**, 3577–3585 (2017). 28460155 10.1002/jcb.26097

[224] Krummel, B. et al. The central role of glutathione peroxidase 4 in the regulation of ferroptosis and its implications for pro-inflammatory cytokine-mediated beta-cell death. *Biochim. Biophys. Acta Mol. Basis Dis.* **1867**, 166114 (2021). 33662571 10.1016/j.bbadis.2021.166114

[225] Sampaio, A. F. et al. Iron toxicity mediated by oxidative stress enhances tissue damage in an animal model of diabetes. *Biometals* **27**, 349–361 (2014). 24549594 10.1007/s10534-014-9717-8

[226] Thompson, P. J. et al. Targeted elimination of senescent beta cells prevents type 1 diabetes. *Cell Metab.* **29**, 1045–1060 (2019). 30799288 10.1016/j.cmet.2019.01.021

[227] Fleming, M. D. et al. Nramp2 is mutated in the anemic Belgrade (b) rat: evidence of a role for Nramp2 in endosomal iron transport. *Proc. Natl. Acad. Sci. USA* **95**, 1148–1153 (1998). 9448300 10.1073/pnas.95.3.1148 PMC18702

[228] Hansen, J. B. et al. Divalent metal transporter 1 regulates iron-mediated ROS and pancreatic beta cell fate in response to cytokines. *Cell Metab.* **16**, 449–461 (2012). 23000401 10.1016/j.cmet.2012.09.001

[229] Cooksey, R. C. et al. Oxidative stress, beta-cell apoptosis, and decreased insulin secretory capacity in mouse models of hemochromatosis. *Endocrinology* **145**, 5305–5312 (2004). 15308612 10.1210/en.2004-0392

[230] Lee, Y. S. & Olefsky, J. Chronic tissue inflammation and metabolic disease. *Genes Dev.* **35**, 307–328 (2021). 33649162 10.1101/gad.346312.120 PMC7919414

[231] Zhang, Y. et al. Positional cloning of the mouse obese gene and its human homologue. *Nature* **372**, 425–432 (1994). 7984236 10.1038/372425a0

[232] Hu, E., Liang, P. & Spiegelman, B. M. AdipoQ is a novel adipose-specific gene dysregulated in obesity. *J. Biol. Chem.* **271**, 10697–10703 (1996). 8631877 10.1074/jbc.271.18.10697

[233] Gabrielsen, J. S. et al. Adipocyte iron regulates adiponectin and insulin sensitivity. *J. Clin. Investig.* **122**, 3529–3540 (2012). 22996660 10.1172/JCI44421 PMC3461897

[234] Gao, Y. et al. Adipocyte iron regulates leptin and food intake. *J. Clin. Investig.* **125**, 3681–3691 (2015). 26301810 10.1172/JCI81860 PMC4588289

[235] Kusminski, C. M. et al. A novel model of diabetic complications: adipocyte mitochondrial dysfunction triggers massive beta-cell hyperplasia. *Diabetes* **69**, 313–330 (2020). 31882562 10.2337/db19-0327 PMC7034182

[236] Altamura, S. et al. Iron aggravates hepatic insulin resistance in the absence of inflammation in a novel db/db mouse model with iron overload. *Mol. Metab.* **51**, 101235 (2021). 33872860 10.1016/j.molmet.2021.101235 PMC8131719

[237] Simcox, J. A. et al. Dietary iron controls circadian hepatic glucose metabolism through heme synthesis. *Diabetes* **64**, 1108–1119 (2015). 25315005 10.2337/db14-0646 PMC4375081

[238] Li, W. et al. A meta-analysis of cohort studies including dose-response relationship between shift work and the risk of diabetes mellitus. *Eur. J. Epidemiol.* **34**, 1013–1024 (2019). 31512118 10.1007/s10654-019-00561-y

[239] Pain, D. & Dancis, A. Roles of Fe-S proteins: from cofactor synthesis to iron homeostasis to protein synthesis. *Curr. Opin. Genet. Dev.* **38**, 45–51 (2016). 27061491 10.1016/j.gde.2016.03.006 PMC5055408

[240] Mena, N. P. et al. Mitochondrial iron homeostasis and its dysfunctions in neurodegenerative disorders. *Mitochondrion* **21**, 92–105 (2015). 25667951 10.1016/j.mito.2015.02.001

[241] Wei, S. et al. Arsenic induces pancreatic dysfunction and ferroptosis via mitochondrial ROS-autophagy-lysosomal pathway. *J. Hazard. Mater.* **384**, 121390 (2020). 31735470 10.1016/j.jhazmat.2019.121390

[242] Hesselink, M. K., Schrauwen-Hinderling, V. & Schrauwen, P. Skeletal muscle mitochondria as a target to prevent or treat type 2 diabetes mellitus. *Nat. Rev. Endocrinol.* **12**, 633–645 (2016). 27448057 10.1038/nrendo.2016.104

[243] Stugiewicz, M. et al. The influence of iron deficiency on the functioning of skeletal muscles: experimental evidence and clinical implications. *Eur. J. Heart Fail.* **18**, 762–773 (2016). 26800032 10.1002/ejhf.467

[244] Lascar, N. et al. Type 2 diabetes in adolescents and young adults. *Lancet Diabetes Endocrinol.* **6**, 69–80 (2018). 28847479 10.1016/S2213-8587(17)30186-9

[245] Luo, E. F. et al. Role of ferroptosis in the process of diabetes-induced endothelial dysfunction. *World J. Diabetes* **12**, 124–137 (2021). 33594332 10.4239/wjd.v12.i2.124 PMC7839168

[246] Hao, L. et al. SLC40A1 mediates ferroptosis and cognitive dysfunction in type 1 diabetes. *Neuroscience* **463**, 216–226 (2021). 33727075 10.1016/j.neuroscience.2021.03.009

[247] Ajoolabady, A. et al. Ferritinophagy and ferroptosis in the management of metabolic diseases. *Trends Endocrinol. Metab.* **32**, 444–462 (2021). 34006412 10.1016/j.tem.2021.04.010

[248] Li, W. et al. Ferroptosis is involved in diabetes myocardial ischemia/reperfusion injury through endoplasmic reticulum stress. *DNA Cell Biol.* **39**, 210–225 (2020). 31809190 10.1089/dna.2019.5097

[249] Liu, P. et al. Ferroptosis: mechanisms and role in diabetes mellitus and its complications. *Ageing Res. Rev.* **94**, 102201 (2024). 38242213 10.1016/j.arr.2024.102201

[250] Fernandez-Real, J. M. et al. Blood letting in high-ferritin type 2 diabetes: effects on insulin sensitivity and beta-cell function. *Diabetes* **51**, 1000–1004 (2002). 11916918 10.2337/diabetes.51.4.1000

[251] Yan, H. F., Liu, Z. Y., Guan, Z. A. & Guo, C. Deferoxamine ameliorates adipocyte dysfunction by modulating iron metabolism in ob/ob mice. *Endocr. Connect.* **7**, 604–616 (2018). 29678877 10.1530/EC-18-0054 PMC5911700

[252] Dongiovanni, P. et al. Iron depletion by deferoxamine up-regulates glucose uptake and insulin signaling in hepatoma cells and in rat liver. *Am. J. Pathol.* **172**, 738–747 (2008). 18245813 10.2353/ajpath.2008.070097 PMC2258266

[253] Cooksey, R. C. et al. Dietary iron restriction or iron chelation protects from diabetes and loss of beta-cell function in the obese (ob/ob lep-/-) mouse. *Am. J. Physiol. Endocrinol. Metab.* **298**, E1236–E1243 (2010). 20354157 10.1152/ajpendo.00022.2010 PMC2886527

[254] Abdul, Y. et al. Deferoxamine treatment prevents post-stroke vasoregression and neurovascular unit remodeling leading to improved functional outcomes in type 2 male diabetic rats: role of endothelial ferroptosis. *Transl. Stroke Res.* **12**, 615–630 (2021). 32875455 10.1007/s12975-020-00844-7 PMC7917163

[255] Zhou, Y. The protective effects of cryptochlorogenic acid on beta-cells function in diabetes in vivo and vitro via inhibition of ferroptosis. *Diabetes Metab. Syndr. Obes.* **13**, 1921–1931 (2020). 32606852 10.2147/DMSO.S249382 PMC7294720

[256] Palacka, P. et al. Complementary therapy in diabetic patients with chronic complications: a pilot study. *Bratisl. Lek. Listy* **111**, 205–211 (2010). 20586147

[257] Sloan, G., Selvarajah, D. & Tesfaye, S. Pathogenesis, diagnosis and clinical management of diabetic sensorimotor peripheral neuropathy. *Nat. Rev. Endocrinol.* **17**, 400–420 (2021). 34050323 10.1038/s41574-021-00496-z

[258] Yarahmadi, A. et al. The effect of platelet-rich plasma-fibrin glue dressing in combination with oral vitamin E and C for treatment of non-healing diabetic foot ulcers: a randomized, double-blind, parallel-group, clinical trial. *Expert Opin. Biol. Ther.* **21**, 687–696 (2021). 33646060 10.1080/14712598.2021.1897100

[259] Liu, C., Wang, W. & Gu, J. Targeting ferroptosis: new perspectives of Chinese herbal medicine in the treatment of diabetes and its complications. *Heliyon* **9**, e22250 (2023). 38076182 10.1016/j.heliyon.2023.e22250 PMC10709212

[260] Fang, X., Ardehali, H., Min, J. & Wang, F. The molecular and metabolic landscape of iron and ferroptosis in cardiovascular disease. *Nat. Rev. Cardiol.* **20**, 7–23 (2023). 35788564 10.1038/s41569-022-00735-4 PMC9252571

[261] Miao, R. et al. Iron metabolism and ferroptosis in type 2 diabetes mellitus and complications: mechanisms and therapeutic opportunities. *Cell Death Dis.* **14**, 186 (2023). 36882414 10.1038/s41419-023-05708-0 PMC9992652

[262] Sha, W. et al. Mechanism of ferroptosis and its role in type 2 diabetes mellitus. *J. Diabetes Res* **2021**, 9999612 (2021). 34258295 10.1155/2021/9999612 PMC8257355

[263] Butler, L. M. et al. Lipids and cancer: emerging roles in pathogenesis, diagnosis and therapeutic intervention. *Adv. Drug Deliv. Rev.* **159**, 245–293 (2020). 32711004 10.1016/j.addr.2020.07.013 PMC7736102

[264] Hoy, A. J., Nagarajan, S. R. & Butler, L. M. Tumour fatty acid metabolism in the context of therapy resistance and obesity. *Nat. Rev. Cancer* **21**, 753–766 (2021). 34417571 10.1038/s41568-021-00388-4

[265] Pope, L. E. & Dixon, S. J. Regulation of ferroptosis by lipid metabolism. *Trends Cell Biol.* **33**, 1077–1087 (2023). 37407304 10.1016/j.tcb.2023.05.003 PMC10733748

[266] Kim, J. et al. Iron loading impairs lipoprotein lipase activity and promotes hypertriglyceridemia. *FASEB J.* **27**, 1657–1663 (2013). 23241313 10.1096/fj.12-224386 PMC3606537

[267] Kim, S. H. et al. High consumption of iron exacerbates hyperlipidemia, atherosclerosis, and female sterility in zebrafish via acceleration of glycation and degradation of serum lipoproteins. *Nutrients* **9**, 960 (2017). 10.3390/nu9070690 PMC5537805 28671593

[268] Mateo-Gallego, R. et al. Serum ferritin is a major determinant of lipid phenotype in familial combined hyperlipidemia and familial hypertriglyceridemia. *Metabolism* **59**, 154–158 (2010). 19913843 10.1016/j.metabol.2009.06.021

[269] Zhou, B., Ren, H., Zhou, X. & Yuan, G. Associations of iron status with apolipoproteins and lipid ratios: a cross-sectional study from the China Health and Nutrition Survey. *Lipids Health Dis.* **19**, 140 (2020). 32546165 10.1186/s12944-020-01312-9 PMC7298938

[270] Li, G. et al. Relationship between serum ferritin level and dyslipidemia in US adults based on data from the national health and nutrition examination surveys 2017 to 2020. *Nutrients* **15** (2023). 10.3390/nu15081878 PMC10143246 37111096

[271] Varela, R. et al. Hyperglycemia and hyperlipidemia can induce morphophysiological changes in rat cardiac cell line. *Biochem. Biophys. Rep.* **26**, 100983 (2021). 33912691 10.1016/j.bbrep.2021.100983 PMC8063753

[272] Wu, X. et al. DiDang decoction improves mitochondrial function and lipid metabolism via the HIF-1 signaling pathway to treat atherosclerosis and hyperlipidemia. *J. Ethnopharmacol.* **308**, 116289 (2023). 36822344 10.1016/j.jep.2023.116289

[273] Fan, Y. et al. Primordial drivers of diabetes heart disease: comprehensive insights into insulin resistance. *Diabetes Metab. J.* **48**, 19–36 (2024). 38173376 10.4093/dmj.2023.0110 PMC10850268

[274] Ma, W. Q., Sun, X. J., Zhu, Y. & Liu, N. F. Metformin attenuates hyperlipidaemia-associated vascular calcification through anti-ferroptotic effects. *Free Radic. Biol. Med.* **165**, 229–242 (2021). 33513420 10.1016/j.freeradbiomed.2021.01.033

[275] Chen, Z., Sun, X., Li, X. & Liu, N. Oleoylethanolamide alleviates hyperlipidaemia-mediated vascular calcification via attenuating mitochondrial DNA stress triggered autophagy-dependent ferroptosis by activating PPARalpha. *Biochem. Pharm.* **208**, 115379 (2023). 36525991 10.1016/j.bcp.2022.115379

[276] Yang, Z. et al. Antiferroptosis therapy alleviated the development of atherosclerosis. *MedComm* **5**, e520 (2024). 38576455 10.1002/mco2.520 PMC10993356

[277] Zhang, J. et al. Echinatin maintains glutathione homeostasis in vascular smooth muscle cells to protect against matrix remodeling and arterial stiffening. *Matrix Biol.* **119**, 1–18 (2023). 36958467 10.1016/j.matbio.2023.03.007

[278] Yang, L. et al. Scavenger receptor class B Type I deficiency induces iron overload and ferroptosis in renal tubular epithelial cells via hypoxia-inducible factor-1alpha/transferrin receptor 1 signaling pathway. *Antioxid. Redox Signal.* **41**, 56-73 (2024). 10.1089/ars.2023.0380 38062756

[279] Zhang, M. et al. Effect of tetramethylpyrazine and hyperlipidemia on hepcidin homeostasis in mice. *Int. J. Mol. Med.* **43**, 501–506 (2019). 30387806 10.3892/ijmm.2018.3968

[280] Xiang, X. et al. Liproxstatin-1 attenuates acute hypertriglyceridemic pancreatitis through inhibiting ferroptosis in rats. *Sci. Rep.* **14**, 9548 (2024). 38664508 10.1038/s41598-024-60159-7 PMC11045844

[281] Yang, M. et al. Puerarin ameliorates metabolic dysfunction-associated fatty liver disease by inhibiting ferroptosis and inflammation. *Lipids Health Dis.* **22**, 202 (2023). 38001459 10.1186/s12944-023-01969-y PMC10668385

[282] El Ayed, M. et al. Protective effects of grape seed and skin extract against high-fat-diet-induced lipotoxicity in rat lung. *Lipids Health Dis.* **16**, 174 (2017). 28903761 10.1186/s12944-017-0561-z PMC5598067

[283] He, L. P., Zhou, Z. X. & Li, C. P. Narrative review of ferroptosis in obesity. *J. Cell Mol. Med.* **27**, 920–926 (2023). 36871273 10.1111/jcmm.17701 PMC10064023

[284] Gonzalez-Dominguez, A. et al. Iron metabolism in obesity and metabolic syndrome. *Int. J. Mol. Sci.* **21**, 5529 (2020). 10.3390/ijms21155529 PMC7432525 32752277

[285] Lecube, A. et al. Iron deficiency in obese postmenopausal women. *Obesity* **14**, 1724–1730 (2006). 17062801 10.1038/oby.2006.198

[286] Yanoff, L. B. et al. Inflammation and iron deficiency in the hypoferremia of obesity. *Int. J. Obes.* **31**, 1412–1419 (2007). 10.1038/sj.ijo.0803625 PMC2266872 17438557

[287] Qiu, F. et al. The role of iron metabolism in chronic diseases related to obesity. *Mol. Med.* **28**, 130 (2022). 36335331 10.1186/s10020-022-00558-6 PMC9636637

[288] Moore Heslin, A. et al. Risk of iron overload in obesity and implications in metabolic health. *Nutrients* **13**,1539 (2021). 10.3390/nu13051539 PMC8147503 34063273

[289] Gotardo, E. M. et al. Mice that are fed a high-fat diet display increased hepcidin expression in adipose tissue. *J. Nutr. Sci. Vitaminol.* **59**, 454–461 (2013). 24418880 10.3177/jnsv.59.454

[290] Bekri, S. et al. Increased adipose tissue expression of hepcidin in severe obesity is independent from diabetes and NASH. *Gastroenterology* **131**, 788–796 (2006). 16952548 10.1053/j.gastro.2006.07.007

[291] Li, W. et al. Identifying ferroptosis-related genes associated with weight loss outcomes and regulation of adipocyte microenvironment. *Mol. Nutr. Food Res.* **67**, e2300168 (2023). 37599272 10.1002/mnfr.202300168

[292] Zhang, Y. et al. High-altitude hypoxia exposure induces iron overload and ferroptosis in adipose tissue. *Antioxidants* **11**, 2367 (2022). 10.3390/antiox11122367 PMC9774922 36552575

[293] Lu, J. et al. Skeletal muscle cystathionine gamma-lyase deficiency promotes obesity and insulin resistance and results in hyperglycemia and skeletal muscle injury upon HFD in mice. *Redox Rep.* **29**, 2347139 (2024). 38718286 10.1080/13510002.2024.2347139 PMC11734987

[294] Zhou, J. et al. The transcriptome reveals the molecular regulatory network of primordial follicle depletion in obese mice. *Fertil. Steril.* **120**, 899–910 (2023). 37247688 10.1016/j.fertnstert.2023.05.165

[295] Schwarzler, J. et al. Adipocyte GPX4 protects against inflammation, hepatic insulin resistance and metabolic dysregulation. *Int. J. Obes.* **46**, 951–959 (2022). 10.1038/s41366-022-01064-9 35031697

[296] Zhang, X., Bao, J., Zhang, Y. & Wang, X. Alpha-linolenic acid ameliorates cognitive impairment and liver damage caused by obesity. *Diabetes Metab. Syndr. Obes.* **17**, 981–995 (2024). 38435630 10.2147/DMSO.S434671 PMC10909331

[297] Zhong, X. et al. Dynamic transcriptome analysis of the muscles in high-fat diet-induced obese zebrafish (Danio rerio) under 5-HT treatment. *Gene* **819**, 146265 (2022). 35121026 10.1016/j.gene.2022.146265

[298] Wan, Y. et al. Nuciferine, an active ingredient derived from lotus leaf, lights up the way for the potential treatment of obesity and obesity-related diseases. *Pharm. Res.* **175**, 106002 (2022). 10.1016/j.phrs.2021.106002 34826599

[299] Kordi, N. et al. Ferroptosis and aerobic training in ageing. *Clin. Hemorheol. Microcirc.* **87**, 347–366 (2024). 38306027 10.3233/CH-232076

[300] Yang, Y. et al. Electroacupuncture reduces inflammatory bowel disease in obese mice by activating the nrf2/ho-1 signaling pathways and repairing the intestinal barrier. *Diabetes Metab. Syndr. Obes.* **17**, 435–452 (2024). 38299195 10.2147/DMSO.S449112 PMC10829509

[301] Krishan, S. Correlation between non-alcoholic fatty liver disease (NAFLD) and dyslipidemia in type 2 diabetes. *Diabetes Metab. Syndr.* **10**, S77–S81 (2016). 26810159 10.1016/j.dsx.2016.01.034

[302] Wang, S. et al. An overview of ferroptosis in non-alcoholic fatty liver disease. *Biomed. Pharmacother.* **153**, 113374 (2022). 35834990 10.1016/j.biopha.2022.113374

[303] Wu, S. et al. Macrophage extracellular traps aggravate iron overload-related liver ischaemia/reperfusion injury. *Br. J. Pharm.* **178**, 3783–3796 (2021). 10.1111/bph.15518 33959955

[304] Dai, X., Zhang, R. & Wang, B. Contribution of classification based on ferroptosis-related genes to the heterogeneity of MAFLD. *BMC Gastroenterol.* **22**, 55 (2022). 35144542 10.1186/s12876-022-02137-9 PMC8830092

[305] Shen, J. et al. Essentiality of SLC7A11-mediated nonessential amino acids in MASLD. *Sci. Bull.*10.1016/j.scib.2024.09.019 (2024). 10.1016/j.scib.2024.09.019 39366830

[306] Loguercio, C. et al. Non-alcoholic fatty liver disease in an area of southern Italy: main clinical, histological, and pathophysiological aspects. *J. Hepatol.* **35**, 568–574 (2001). 11690701 10.1016/s0168-8278(01)00192-1

[307] Jia, M. et al. Ferroptosis as a new therapeutic opportunity for nonviral liver disease. *Eur. J. Pharm.* **908**, 174319 (2021). 10.1016/j.ejphar.2021.174319 34252441

[308] Sun, J. et al. Fatty acid binding protein 5 suppression attenuates obesity-induced hepatocellular carcinoma by promoting ferroptosis and intratumoral immune rewiring. *Nat. Metab.* **6**, 741–763 (2024). 38664583 10.1038/s42255-024-01019-6 PMC12355809

[309] Fu, J. T. et al. Targeting EFHD2 inhibits interferon-gamma signaling and ameliorates non-alcoholic steatohepatitis. *J. Hepatol.* **81***,* 389-403 (2024). 10.1016/j.jhep.2024.04.009 38670321

[310] Franchini, M. et al. Safety and efficacy of subcutaneous bolus injection of deferoxamine in adult patients with iron overload. *Blood* **95**, 2776–2779 (2000). 10779420

[311] Aydinok, Y. et al. Effects of deferasirox-deferoxamine on myocardial and liver iron in patients with severe transfusional iron overload. *Blood* **125**, 3868–3877 (2015). 25934475 10.1182/blood-2014-07-586677 PMC4490296

[312] Olivieri, N. F. et al. Long-term safety and effectiveness of iron-chelation therapy with deferiprone for thalassemia major. *N. Engl. J. Med.* **339**, 417–423 (1998). 9700174 10.1056/NEJM199808133390701

[313] Kong, Z., Liu, R. & Cheng, Y. Artesunate alleviates liver fibrosis by regulating ferroptosis signaling pathway. *Biomed. Pharmacother.* **109**, 2043–2053 (2019). 30551460 10.1016/j.biopha.2018.11.030

[314] Nobuta, H. et al. Oligodendrocyte death in pelizaeus-merzbacher disease is rescued by iron chelation. *Cell Stem Cell* **25**, 531–541.e6 (2019). 31585094 10.1016/j.stem.2019.09.003 PMC8282124

[315] Tsurusaki, S. et al. Hepatic ferroptosis plays an important role as the trigger for initiating inflammation in nonalcoholic steatohepatitis. *Cell Death Dis.* **10**, 449 (2019). 31209199 10.1038/s41419-019-1678-y PMC6579767

[316] Tao, L. et al. Integrative clinical and preclinical studies identify FerroTerminator1 as a potent therapeutic drug for MASH. *Cell Metabolism.*10.1016/j.cmet.2024.07.013 (2024). 10.1016/j.cmet.2024.07.013 39142286

[317] Li, X. et al. Targeting ferroptosis alleviates methionine-choline deficient (MCD)-diet induced NASH by suppressing liver lipotoxicity. *Liver Int* **40**, 1378–1394 (2020). 32145145 10.1111/liv.14428

[318] Qi, J. et al. Ferroptosis affects the progression of nonalcoholic steatohepatitis via the modulation of lipid peroxidation-mediated cell death in mice. *Am. J. Pathol.* **190**, 68–81 (2020). 31610178 10.1016/j.ajpath.2019.09.011

[319] Anthonymuthu, T. S. et al. Resolving the paradox of ferroptotic cell death: ferrostatin-1 binds to 15LOX/PEBP1 complex, suppresses generation of peroxidized ETE-PE, and protects against ferroptosis. *Redox Biol.* **38**, 101744 (2021). 33126055 10.1016/j.redox.2020.101744 PMC7596334

[320] Zhu, Z. et al. Thymosin beta 4 alleviates non-alcoholic fatty liver by inhibiting ferroptosis via up-regulation of GPX4. *Eur. J. Pharm.* **908**, 174351 (2021). 10.1016/j.ejphar.2021.174351 34280397

[321] Tong, J. et al. Targeting a novel inducible GPX4 alternative isoform to alleviate ferroptosis and treat metabolic-associated fatty liver disease. *Acta Pharm. Sin. B* **12**, 3650–3666 (2022). 36176906 10.1016/j.apsb.2022.02.003 PMC9513461

[322] Carlson, B. A. et al. Glutathione peroxidase 4 and vitamin E cooperatively prevent hepatocellular degeneration. *Redox Biol.* **9**, 22–31 (2016). 27262435 10.1016/j.redox.2016.05.003 PMC4900515

[323] Lavine, J. E. et al. Effect of vitamin E or metformin for treatment of nonalcoholic fatty liver disease in children and adolescents: the TONIC randomized controlled trial. *JAMA* **305**, 1659–1668 (2011). 21521847 10.1001/jama.2011.520 PMC3110082

[324] Sanyal, A. J. et al. Pioglitazone, vitamin E, or placebo for nonalcoholic steatohepatitis. *N. Engl. J. Med.* **362**, 1675–1685 (2010). 20427778 10.1056/NEJMoa0907929 PMC2928471

[325] Saryusz-Wolska, M. et al. Rosiglitazone treatment in nondiabetic subjects with nonalcoholic fatty liver disease. *Pol. Arch. Med. Wewn.* **121**, 61–66 (2011). 21430606

[326] Wang, C. H., Leung, C. H., Liu, S. C. & Chung, C. H. Safety and effectiveness of rosiglitazone in type 2 diabetes patients with nonalcoholic Fatty liver disease. *J. Formos. Med. Assoc.* **105**, 743–752 (2006). 16959622 10.1016/S0929-6646(09)60202-3 PMC7134933

[327] Wei, S. et al. Ferroptosis mediated by the interaction between Mfn2 and IREalpha promotes arsenic-induced nonalcoholic steatohepatitis. *Environ. Res.* **188**, 109824 (2020). 32593899 10.1016/j.envres.2020.109824

[328] Zhang, Y. et al. Computational repositioning of dimethyl fumarate for treating alcoholic liver disease. *Cell Death Dis.* **11**, 641 (2020). 32811823 10.1038/s41419-020-02890-3 PMC7434920

[329] Kim, J. et al. The natural phytochemical dehydroabietic acid is an anti-aging reagent that mediates the direct activation of SIRT1. *Mol. Cell Endocrinol.* **412**, 216–225 (2015). 25976661 10.1016/j.mce.2015.05.006

[330] Kim, E. et al. Dehydroabietic acid suppresses inflammatory response via suppression of Src-, Syk-, and TAK1-mediated pathways. *Int. J. Mol. Sci.* **20**, 1593 (2019). 10.3390/ijms20071593 PMC6480320 30934981

[331] Gao, G. et al. Dehydroabietic acid improves nonalcoholic fatty liver disease through activating the Keap1/Nrf2-ARE signaling pathway to reduce ferroptosis. *J. Nat. Med.* **75**, 540–552 (2021). 33590347 10.1007/s11418-021-01491-4

[332] Yang, Y. et al. Study on the attenuated effect of Ginkgolide B on ferroptosis in high fat diet induced nonalcoholic fatty liver disease. *Toxicology* **445**, 152599 (2020). 32976958 10.1016/j.tox.2020.152599

[333] Guan, Q. et al. Melatonin ameliorates hepatic ferroptosis in NAFLD by inhibiting ER stress via the MT2/cAMP/PKA/IRE1 signaling pathway. *Int J. Biol. Sci.* **19**, 3937–3950 (2023). 37564204 10.7150/ijbs.85883 PMC10411470

[334] Guo, T. et al. Liraglutide attenuates type 2 diabetes mellitus-associated non-alcoholic fatty liver disease by activating AMPK/ACC signaling and inhibiting ferroptosis. *Mol. Med.* **29**, 132 (2023). 37770820 10.1186/s10020-023-00721-7 PMC10540362

[335] Choi, J., Choi, H. & Chung, J. Icariin supplementation suppresses the markers of ferroptosis and attenuates the progression of nonalcoholic steatohepatitis in mice fed a methionine choline-deficient diet. *Int. J. Mol. Sci.* **24**, 12510 (2023). 10.3390/ijms241512510 PMC10419585 37569885

[336] Liu, H. et al. Zeaxanthin prevents ferroptosis by promoting mitochondrial function and inhibiting the p53 pathway in free fatty acid-induced HepG2 cells. *Biochim. Biophys. Acta Mol. Cell Biol. Lipids* **1868**, 159287 (2023). 36690321 10.1016/j.bbalip.2023.159287

[337] Liu, B. et al. Enoyl coenzyme A hydratase 1 alleviates nonalcoholic steatohepatitis in mice by suppressing hepatic ferroptosis. *Am. J. Physiol. Endocrinol. Metab.* **320**, E925–E937 (2021). 33813878 10.1152/ajpendo.00614.2020

[338] Wu, A. et al. Fibroblast growth factor 21 attenuates iron overload-induced liver injury and fibrosis by inhibiting ferroptosis. *Redox Biol.* **46**, 102131 (2021). 34530349 10.1016/j.redox.2021.102131 PMC8445902

[339] Gravallese, E. M. et al. What is rheumatoid arthritis? *N. Engl. J. Med.* **390**, e32 (2024). 38598569 10.1056/NEJMp2310178

[340] Brown, P., Pratt, A. G. & Hyrich, K. L. Therapeutic advances in rheumatoid arthritis. *BMJ* **384**, e070856 (2024). 38233032 10.1136/bmj-2022-070856

[341] Xie, Z. et al. ROS-dependent lipid peroxidation and reliant antioxidant ferroptosis-suppressor-protein 1 in rheumatoid arthritis: a covert clue for potential therapy. *Inflammation* **44**, 35–47 (2021). 32920707 10.1007/s10753-020-01338-2

[342] Pelissier, A. et al. Gene network analyses identify co-regulated transcription factors and BACH1 as a key driver in rheumatoid arthritis fibroblast-like synoviocytes. *bioRxiv* (2024). 10.26508/lsa.202402808 PMC11519322 39467637

[343] Walker, E. M. Jr. & Walker, S. M. Effects of iron overload on the immune system. *Ann. Clin. Lab Sci.* **30**, 354–365 (2000). 11045759

[344] Baker, J. F. & Ghio, A. J. Iron homoeostasis in rheumatic disease. *Rheumatology* **48**, 1339–1344 (2009). 19628641 10.1093/rheumatology/kep221

[345] Pantopoulos, K., Porwal, S. K., Tartakoff, A. & Devireddy, L. Mechanisms of mammalian iron homeostasis. *Biochemistry* **51**, 5705–5724 (2012). 22703180 10.1021/bi300752r PMC3572738

[346] Yazar, M., Sarban, S., Kocyigit, A. & Isikan, U. E. Synovial fluid and plasma selenium, copper, zinc, and iron concentrations in patients with rheumatoid arthritis and osteoarthritis. *Biol. Trace Elem. Res.* **106**, 123–132 (2005). 16116244 10.1385/BTER:106:2:123

[347] Ahmadzadeh, N., Shingu, M. & Nobunaga, M. Iron-binding proteins and free iron in synovial fluids of rheumatoid arthritis patients. *Clin. Rheumatol.* **8**, 345–351 (1989). 2805610 10.1007/BF02030347

[348] Dabbagh, A. J., Blake, D. R. & Morris, C. J. Effect of iron complexes on adjuvant arthritis in rats. *Ann. Rheum. Dis.* **51**, 516–521 (1992). 1586252 10.1136/ard.51.4.516 PMC1004704

[349] Danks, L. et al. RANKL expressed on synovial fibroblasts is primarily responsible for bone erosions during joint inflammation. *Ann. Rheum. Dis.* **75**, 1187–1195 (2016). 26025971 10.1136/annrheumdis-2014-207137

[350] Hassan, S. Z. et al. Oxidative stress in systemic lupus erythematosus and rheumatoid arthritis patients: relationship to disease manifestations and activity. *Int. J. Rheum. Dis.* **14**, 325–331 (2011). 22004228 10.1111/j.1756-185X.2011.01630.x

[351] Chadha, S. et al. Role of Nrf2 in rheumatoid arthritis. *Curr. Res. Transl. Med.* **68**, 171–181 (2020). 32620467 10.1016/j.retram.2020.05.002

[352] Zhang, Y. et al. Nrf2-Keap1 pathway-mediated effects of resveratrol on oxidative stress and apoptosis in hydrogen peroxide-treated rheumatoid arthritis fibroblast-like synoviocytes. *Ann. N. Y Acad. Sci.* **1457**, 166–178 (2019). 31475364 10.1111/nyas.14196

[353] Drijvers, J. M. et al. Pharmacologic screening identifies metabolic vulnerabilities of CD8(+) T cells. *Cancer Immunol. Res.* **9**, 184–199 (2021). 33277233 10.1158/2326-6066.CIR-20-0384 PMC7864883

[354] Tsaltskan, V. & Firestein, G. S. Targeting fibroblast-like synoviocytes in rheumatoid arthritis. *Curr. Opin. Pharm.* **67**, 102304 (2022). 10.1016/j.coph.2022.102304 PMC9942784 36228471

[355] Liu, Y. et al. Heterogeneous ferroptosis susceptibility of macrophages caused by focal iron overload exacerbates rheumatoid arthritis. *Redox Biol.* **69**, 103008 (2024). 38142586 10.1016/j.redox.2023.103008 PMC10788633

[356] Ba, T. et al. L-citrulline supplementation restrains ferritinophagy-mediated ferroptosis to alleviate iron overload-induced thymus oxidative damage and immune dysfunction. *Nutrients* **14**, 4549 (2022). 10.3390/nu14214549 PMC9655478 36364817

[357] Ling, H. et al. Glycine increased ferroptosis via SAM-mediated GPX4 promoter methylation in rheumatoid arthritis. *Rheumatology* **61**, 4521–4534 (2022). 35136972 10.1093/rheumatology/keac069

[358] Taghadosi, M. et al. The p53 status in rheumatoid arthritis with focus on fibroblast-like synoviocytes. *Immunol. Res.* **69**, 225–238 (2021). 33983569 10.1007/s12026-021-09202-7

[359] Su, Y. et al. Ferroptosis, a novel pharmacological mechanism of anti-cancer drugs. *Cancer Lett.* **483**, 127–136 (2020). 32067993 10.1016/j.canlet.2020.02.015

[360] Zhou, X., Mi, J. & Liu, Z. Causal association of diet-derived circulating antioxidants with the risk of rheumatoid arthritis: a Mendelian randomization study. *Semin. Arthritis Rheum.* **56**, 152079 (2022). 35932494 10.1016/j.semarthrit.2022.152079

[361] Jhun, J. et al. Liposome/gold hybrid nanoparticle encoded with CoQ10 (LGNP-CoQ10) suppressed rheumatoid arthritis via STAT3/Th17 targeting. *PLoS One* **15**, e0241080 (2020). 33156836 10.1371/journal.pone.0241080 PMC7647073

[362] Ravalli, S., Szychlinska, M. A., Leonardi, R. M. & Musumeci, G. Recently highlighted nutraceuticals for preventive management of osteoarthritis. *World J. Orthop.* **9**, 255–261 (2018). 30479972 10.5312/wjo.v9.i11.255 PMC6242728

[363] Jean-Gilles, D. et al. Inhibitory effects of polyphenol punicalagin on type-II collagen degradation in vitro and inflammation in vivo. *Chem. Biol. Interact.* **205**, 90–99 (2013). 23830812 10.1016/j.cbi.2013.06.018

[364] Luo, H. & Zhang, R. Icariin enhances cell survival in lipopolysaccharide-induced synoviocytes by suppressing ferroptosis via the Xc-/GPX4 axis. *Exp. Ther. Med.* **21**, 72 (2021). 33365072 10.3892/etm.2020.9504 PMC7716635

[365] Zhou, S. et al. Emodin alleviates joint inflammation and bone erosion in rats with collagen-induced arthritis by inhibiting ferroptosis and degrading matrix metalloproteinases. *Nan Fang Yi Ke Da Xue Xue Bao* **43**, 1776–1781 (2023). 37933654 10.12122/j.issn.1673-4254.2023.10.16 PMC10630201

[366] Zhou, M. J. et al. Total triterpenes of Euphorbium alleviates rheumatoid arthritis via Nrf2/HO-1/GPX4 pathway. *Zhongguo Zhong Yao Za Zhi* **48**, 4834–4842 (2023). 37802825 10.19540/j.cnki.cjcmm.20230601.704

[367] Demirtzoglou, G. et al. Haloperidol’s cytogenetic effect on T lymphocytes of systemic lupus erythematosus and rheumatoid arthritis patients: an in vitro study. *Cureus* **15**, e42283 (2023). 37609095 10.7759/cureus.42283 PMC10440589

[368] Peng, C. Y. et al. Effects of moxibustion on p53, SLC7A11, and GPX4 expression in synovial tissues of rats with adjuvant arthritis]. *Zhen Ci Yan Jiu* **47**, 21–26 (2022). 35128866 10.13702/j.1000-0607.20210837

[369] Garcia-Romo, G. S. et al. Netting neutrophils are major inducers of type I IFN production in pediatric systemic lupus erythematosus. *Sci. Transl. Med.* **3**, 73ra20 (2011). 21389264 10.1126/scitranslmed.3001201 PMC3143837

[370] Li, P. et al. Glutathione peroxidase 4-regulated neutrophil ferroptosis induces systemic autoimmunity. *Nat. Immunol.* **22**, 1107–1117 (2021). 34385713 10.1038/s41590-021-00993-3 PMC8609402

[371] Ohl, K., Rauen, T. & Tenbrock, K. Dysregulated neutrophilic cell death in SLE: a spotlight on ferroptosis. *Signal Transduct. Target Ther.* **6**, 392 (2021). 34764247 10.1038/s41392-021-00804-z PMC8586233

[372] Yang, B. et al. Ferroptosis inhibitor regulates the disease progression of systematic lupus erythematosus mice model through Th1/Th2 ratio. *Curr. Mol. Med.* **23**, 799–807 (2023). 35619279 10.2174/1566524022666220525144630

[373] Tao, K. et al. Ferroptosis in peripheral blood mononuclear cells of systemic lupus erythematosus. *Clin. Exp. Rheumatol.* **42**, 651–657 (2024). 38294021 10.55563/clinexprheumatol/kylvva

[374] Chang, Y. et al. Erucic acid improves the progress of pregnancy complicated with systemic lupus erythematosus by inhibiting the effector function of CD8(+) T cells. *MedComm (2020)* **4**, e382 (2023). 37771913 10.1002/mco2.382 PMC10522964

[375] Payet, C. A. et al. Myasthenia gravis: an acquired interferonopathy? *Cells* **11**, 1218 (2022). 10.3390/cells11071218 PMC8997999 35406782

[376] Huang, J., Yan, Z., Song, Y. & Chen, T. Nanodrug delivery systems for myasthenia gravis: advances and perspectives. *Pharmaceutics* **16**, 651 (2024). 10.3390/pharmaceutics16050651 PMC11125447 38794313

[377] Huang, Y. et al. Ferroptosis in a sarcopenia model of senescence accelerated mouse prone 8 (SAMP8). *Int. J. Biol. Sci.* **17**, 151–162 (2021). 33390840 10.7150/ijbs.53126 PMC7757032

[378] Li, K. et al. Iron metabolism in non-anemic myasthenia gravis patients: a cohort study. *J. Neuroimmunol.* **375**, 578015 (2023). 36682196 10.1016/j.jneuroim.2023.578015

[379] Huang, P. The relationship between serum iron levels and AChR-Ab and IL-6 in patients with myasthenia gravis. *Eur. Rev. Med. Pharm. Sci.* **27**, 98–102 (2023). 10.26355/eurrev_202301_30857 36647855

[380] Sung, H. K., Murugathasan, M., Abdul-Sater, A. A. & Sweeney, G. Autophagy deficiency exacerbates iron overload induced reactive oxygen species production and apoptotic cell death in skeletal muscle cells. *Cell Death Dis.* **14**, 252 (2023). 37029101 10.1038/s41419-022-05484-3 PMC10081999

[381] Ding, H. et al. Transferrin receptor 1 ablation in satellite cells impedes skeletal muscle regeneration through activation of ferroptosis. *J. Cachexia Sarcopenia Muscle* **12**, 746–768 (2021). 33955709 10.1002/jcsm.12700 PMC8200440

[382] Krishnan, K., Trobe, J. D. & Adams, P. T. Myasthenia gravis following iron chelation therapy with intravenous desferrioxamine. *Eur. J. Haematol.* **55**, 138–139 (1995). 7628591 10.1111/j.1600-0609.1995.tb01826.x

[383] Duan, G. et al. Mitochondrial iron metabolism: the crucial actors in diseases. *Molecules* **28**, 29 (2022). 10.3390/molecules28010029 PMC9822237 36615225

[384] Rouault, T. A. Mitochondrial iron overload: causes and consequences. *Curr. Opin. Genet Dev.* **38**, 31–37 (2016). 27026139 10.1016/j.gde.2016.02.004 PMC5035716

[385] Bellanti, F., Lo Buglio, A. & Vendemiale, G. Muscle delivery of mitochondria-targeted drugs for the treatment of sarcopenia: rationale and perspectives. *Pharmaceutics* **14**, 2588 (2022). 10.3390/pharmaceutics14122588 PMC9782427 36559079

[386] Liu, Y. et al. Potential mechanisms of uremic muscle wasting and the protective role of the mitochondria-targeted antioxidant Mito-TEMPO. *Int. Urol. Nephrol.* **52**, 1551–1561 (2020). 32488756 10.1007/s11255-020-02508-9

[387] van de Weijer, T. et al. Evidence for a direct effect of the NAD+ precursor acipimox on muscle mitochondrial function in humans. *Diabetes* **64**, 1193–1201 (2015). 25352640 10.2337/db14-0667 PMC4375076

[388] Zhou, Y. et al. Extracellular vesicles encapsulated with caspase-1 inhibitor ameliorate experimental autoimmune myasthenia gravis through targeting macrophages. *J. Control Release* **364**, 458–472 (2023). 37935259 10.1016/j.jconrel.2023.11.006

[389] Raimondo, T. M. & Mooney, D. J. Anti-inflammatory nanoparticles significantly improve muscle function in a murine model of advanced muscular dystrophy. *Sci. Adv.* **7**, eabh3693 (2021). 10.1126/sciadv.abh3693 PMC8221619 34162554

[390] Turjeman, K. et al. Liposomal steroid nano-drug is superior to steroids as-is in mdx mouse model of Duchenne muscular dystrophy. *Nanomedicine* **16**, 34–44 (2019). 30529791 10.1016/j.nano.2018.11.012

[391] Raimondo, T. M. & Mooney, D. J. Functional muscle recovery with nanoparticle-directed M2 macrophage polarization in mice. *Proc. Natl. Acad. Sci. USA* **115**, 10648–10653 (2018). 30275293 10.1073/pnas.1806908115 PMC6196479

[392] Adams, P. C., Jeffrey, G. & Ryan, J. Haemochromatosis. *Lancet* **401**, 1811–1821 (2023). 37121243 10.1016/S0140-6736(23)00287-8

[393] Girelli, D. et al. Hemochromatosis classification: update and recommendations by the BIOIRON Society. *Blood* **139**, 3018–3029 (2022). 34601591 10.1182/blood.2021011338 PMC11022970

[394] Brissot, P. et al. Haemochromatosis. *Nat. Rev. Dis. Prim.* **4**, 18016 (2018). 29620054 10.1038/nrdp.2018.16 PMC7775623

[395] Huang, F. W. et al. A mouse model of juvenile hemochromatosis. *J. Clin. Investig.* **115**, 2187–2191 (2005). 16075059 10.1172/JCI25049 PMC1180543

[396] Wang, H. et al. Characterization of ferroptosis in murine models of hemochromatosis. *Hepatology* **66**, 449–465 (2017). 28195347 10.1002/hep.29117 PMC5573904

[397] Brissot, P., Bardou-Jacquet, E., Jouanolle, A. M. & Loreal, O. Iron disorders of genetic origin: a changing world. *Trends Mol. Med.* **17**, 707–713 (2011). 21862411 10.1016/j.molmed.2011.07.004

[398] Musallam, K. M., Cappellini, M. D., Wood, J. C. & Taher, A. T. Iron overload in non-transfusion-dependent thalassemia: a clinical perspective. *Blood Rev.* **26**, S16–S19 (2012). 22631036 10.1016/S0268-960X(12)70006-1

[399] Ramos, E. et al. Minihepcidins prevent iron overload in a hepcidin-deficient mouse model of severe hemochromatosis. *Blood* **120**, 3829–3836 (2012). 22990014 10.1182/blood-2012-07-440743 PMC3488893

[400] Preza, G. C. et al. Minihepcidins are rationally designed small peptides that mimic hepcidin activity in mice and may be useful for the treatment of iron overload. *J. Clin. Investig.* **121**, 4880–4888 (2011). 22045566 10.1172/JCI57693 PMC3225996

[401] Park, T. J. et al. Cloning and characterization of TMPRSS6, a novel type 2 transmembrane serine protease. *Mol. Cells* **19**, 223–227 (2005). 15879706

[402] Taher, A. T., Weatherall, D. J. & Cappellini, M. D. Thalassaemia. *Lancet* **391**, 155–167 (2018). 28774421 10.1016/S0140-6736(17)31822-6

[403] Merkeley, H. & Bolster, L. Thalassemia. *CMAJ* **192**, E1210 (2020). 33051316 10.1503/cmaj.191613 PMC7588257

[404] Hokland, P. et al. Thalassaemia-A global view. *Br. J. Haematol.* **201**, 199–214 (2023). 36799486 10.1111/bjh.18671

[405] Motta, I., Bou-Fakhredin, R., Taher, A. T. & Cappellini, M. D. Beta thalassemia: new therapeutic options beyond transfusion and iron chelation. *Drugs* **80**, 1053–1063 (2020). 32557398 10.1007/s40265-020-01341-9 PMC7299245

[406] Saliba, A. N., Musallam, K. M. & Taher, A. T. How I treat non-transfusion-dependent beta-thalassemia. *Blood* **142**, 949–960 (2023). 37478396 10.1182/blood.2023020683 PMC10644094

[407] Taher, A. T. & Saliba, A. N. Iron overload in thalassemia: different organs at different rates. *Hematol. Am. Soc. Hematol. Educ. Program* **2017**, 265–271 (2017). 10.1182/asheducation-2017.1.265 PMC6142532 29222265

[408] Moukhadder, H. M., Halawi, R., Cappellini, M. D. & Taher, A. T. Hepatocellular carcinoma as an emerging morbidity in the thalassemia syndromes: a comprehensive review. *Cancer* **123**, 751–758 (2017). 27911488 10.1002/cncr.30462

[409] Kurtoglu, A. U., Kurtoglu, E. & Temizkan, A. K. Effect of iron overload on endocrinopathies in patients with beta-thalassaemia major and intermedia. *Endokrynol. Pol.* **63**, 260–263 (2012). 22933160

[410] Quinn, C. T. et al. Renal dysfunction in patients with thalassaemia. *Br. J. Haematol.* **153**, 111–117 (2011). 21332704 10.1111/j.1365-2141.2010.08477.x PMC4250090

[411] Somparn, N. et al. Cellular adaptation mediated through Nrf2-induced glutamate cysteine ligase up-regulation against oxidative stress caused by iron overload in beta-thalassemia/HbE patients. *Free Radic. Res.* **53**, 791–799 (2019). 31198069 10.1080/10715762.2019.1632444

[412] Gardenghi, S. et al. Hepcidin as a therapeutic tool to limit iron overload and improve anemia in beta-thalassemic mice. *J. Clin. Investig.* **120**, 4466–4477 (2010). 21099112 10.1172/JCI41717 PMC2993583

[413] Casu, C. et al. Minihepcidins improve ineffective erythropoiesis and splenomegaly in a new mouse model of adult beta-thalassemia major. *Haematologica* **105**, 1835–1844 (2020). 31582543 10.3324/haematol.2018.212589 PMC7327634

[414] Casu, C. et al. Minihepcidin peptides as disease modifiers in mice affected by beta-thalassemia and polycythemia vera. *Blood* **128**, 265–276 (2016). 27154187 10.1182/blood-2015-10-676742 PMC4946204

[415] Manolova, V. et al. Oral ferroportin inhibitor ameliorates ineffective erythropoiesis in a model of beta-thalassemia. *J. Clin. Investig.* **130**, 491–506 (2019). 31638596 10.1172/JCI129382 PMC6934209

[416] Delatycki, M. B. & Bidichandani, S. I. Friedreich ataxia- pathogenesis and implications for therapies. *Neurobiol. Dis.* **132**, 104606 (2019). 31494282 10.1016/j.nbd.2019.104606

[417] Cook, A. & Giunti, P. Friedreich’s ataxia: clinical features, pathogenesis and management. *Br. Med. Bull.* **124**, 19–30 (2017). 29053830 10.1093/bmb/ldx034 PMC5862303

[418] La Rosa, P. et al. Ferroptosis in friedreich’s ataxia: a metal-induced neurodegenerative disease. *Biomolecules* **10**, 1551 (2020). 10.3390/biom10111551 PMC7696618 33202971

[419] Lees, J. G. et al. Cellular pathophysiology of Friedreich’s ataxia cardiomyopathy. *Int. J. Cardiol.* **346**, 71–78 (2022). 34798207 10.1016/j.ijcard.2021.11.033

[420] Koeppen, A. H. et al. The pathogenesis of cardiomyopathy in Friedreich ataxia. *PLoS One* **10**, e0116396 (2015). 25738292 10.1371/journal.pone.0116396 PMC4349588

[421] Abeti, R. et al. Mitochondrial energy imbalance and lipid peroxidation cause cell death in Friedreich’s ataxia. *Cell Death Dis.* **7**, e2237 (2016). 27228352 10.1038/cddis.2016.111 PMC4917650

[422] Abeti, R. et al. Targeting lipid peroxidation and mitochondrial imbalance in Friedreich’s ataxia. *Pharm. Res.* **99**, 344–350 (2015). 10.1016/j.phrs.2015.05.015 26141703

[423] Lupoli, F. et al. The role of oxidative stress in Friedreich’s ataxia. *FEBS Lett.* **592**, 718–727 (2018). 29197070 10.1002/1873-3468.12928 PMC5887922

[424] Turchi, R., Faraonio, R., Lettieri-Barbato, D. & Aquilano, K. An overview of the ferroptosis hallmarks in Friedreich’s ataxia. *Biomolecules* **10**, 1489(2020). 10.3390/biom10111489 PMC7693407 33126466

[425] Wilson, R. B. et al. Increased serum transferrin receptor concentrations in Friedreich ataxia. *Ann. Neurol.* **47**, 659–661 (2000). 10805340

[426] Scarano, V. et al. Serum transferrin receptor levels in Friedreich’s and other degenerative ataxias. *Neurology* **57**, 159–160 (2001). 11445653 10.1212/wnl.57.1.159

[427] Huang, M. L. et al. Elucidation of the mechanism of mitochondrial iron loading in Friedreich’s ataxia by analysis of a mouse mutant. *Proc. Natl. Acad. Sci. USA* **106**, 16381–16386 (2009). 19805308 10.1073/pnas.0906784106 PMC2752539

[428] Wang, P. et al. Mitochondrial ferritin attenuates cerebral ischaemia/reperfusion injury by inhibiting ferroptosis. *Cell Death Dis.* **12**, 447 (2021). 33953171 10.1038/s41419-021-03725-5 PMC8099895

[429] Campanella, A. et al. The expression of human mitochondrial ferritin rescues respiratory function in frataxin-deficient yeast. *Hum. Mol. Genet.* **13**, 2279–2288 (2004). 15282205 10.1093/hmg/ddh232

[430] Desmyter, L. et al. Expression of the human ferritin light chain in a frataxin mutant yeast affects ageing and cell death. *Exp. Gerontol.* **39**, 707–715 (2004). 15130665 10.1016/j.exger.2004.01.008

[431] Tozzi, G. et al. Antioxidant enzymes in blood of patients with Friedreich’s ataxia. *Arch. Dis. Child* **86**, 376–379 (2002). 11970939 10.1136/adc.86.5.376 PMC1751091

[432] Cotticelli, M. G. et al. Ferroptosis as a novel therapeutic target for Friedreich’s ataxia. *J. Pharm. Exp. Ther.* **369**, 47–54 (2019). 10.1124/jpet.118.252759 30635474

[433] Auchere, F. et al. Glutathione-dependent redox status of frataxin-deficient cells in a yeast model of Friedreich’s ataxia. *Hum. Mol. Genet.* **17**, 2790–2802 (2008). 18562474 10.1093/hmg/ddn178

[434] Turchi, R. et al. Frataxin deficiency induces lipid accumulation and affects thermogenesis in brown adipose tissue. *Cell Death Dis.* **11**, 51 (2020). 31974344 10.1038/s41419-020-2253-2 PMC6978516

[435] Jauslin, M. L., Wirth, T., Meier, T. & Schoumacher, F. A cellular model for Friedreich ataxia reveals small-molecule glutathione peroxidase mimetics as novel treatment strategy. *Hum. Mol. Genet.* **11**, 3055–3063 (2002). 12417527 10.1093/hmg/11.24.3055

[436] La Rosa, P. et al. The Nrf2 induction prevents ferroptosis in Friedreich’s ataxia. *Redox Biol.* **38**, 101791 (2021). 33197769 10.1016/j.redox.2020.101791 PMC7677700

[437] Coppola, G. et al. Functional genomic analysis of frataxin deficiency reveals tissue-specific alterations and identifies the PPARgamma pathway as a therapeutic target in Friedreich’s ataxia. *Hum. Mol. Genet.* **18**, 2452–2461 (2009). 19376812 10.1093/hmg/ddp183 PMC2694693

[438] Puccio, H. et al. Mouse models for Friedreich ataxia exhibit cardiomyopathy, sensory nerve defect and Fe-S enzyme deficiency followed by intramitochondrial iron deposits. *Nat. Genet.* **27**, 181–186 (2001). 11175786 10.1038/84818

[439] Bradley, J. L. et al. Role of oxidative damage in Friedreich’s ataxia. *Neurochem. Res.* **29**, 561–567 (2004). 15038603 10.1023/b:nere.0000014826.00881.c3

[440] Czlonkowska, A. et al. Wilson disease. *Nat. Rev. Dis. Prim.* **4**, 21 (2018). 30190489 10.1038/s41572-018-0018-3 PMC6416051

[441] Chen, J. et al. The molecular mechanisms of copper metabolism and its roles in human diseases. *Pflug. Arch.* **472**, 1415–1429 (2020). 10.1007/s00424-020-02412-2 32506322

[442] Bandmann, O., Weiss, K. H. & Kaler, S. G. Wilson’s disease and other neurological copper disorders. *Lancet Neurol.* **14**, 103–113 (2015). 25496901 10.1016/S1474-4422(14)70190-5 PMC4336199

[443] Li, Y. et al. Iron and copper: critical executioners of ferroptosis, cuproptosis and other forms of cell death. *Cell Commun. Signal.* **21**, 327 (2023). 37974196 10.1186/s12964-023-01267-1 PMC10652626

[444] Tsang, T., Davis, C. I. & Brady, D. C. Copper biology. *Curr. Biol.* **31**, R421–R427 (2021). 33974864 10.1016/j.cub.2021.03.054

[445] Xue, Q. et al. Copper metabolism in cell death and autophagy. *Autophagy* **19**, 2175–2195 (2023). 37055935 10.1080/15548627.2023.2200554 PMC10351475

[446] Arredondo, M. & Nunez, M. T. Iron and copper metabolism. *Mol. Asp. Med.* **26**, 313–327 (2005). 10.1016/j.mam.2005.07.010 16112186

[447] Doguer, C., Ha, J. H. & Collins, J. F. Intersection of iron and copper metabolism in the mammalian intestine and liver. *Compr. Physiol.* **8**, 1433–1461 (2018). 30215866 10.1002/cphy.c170045 PMC6460475

[448] Pak, K. et al. Wilson’s disease and iron overload: pathophysiology and therapeutic implications. *Clin. Liver Dis.* **17**, 61–66 (2021). 10.1002/cld.986 PMC7916432 33680437

[449] Weiskirchen, S., Kim, P. & Weiskirchen, R. Determination of copper poisoning in Wilson’s disease using laser ablation inductively coupled plasma mass spectrometry. *Ann. Transl. Med.* **7**, S72 (2019). 31179309 10.21037/atm.2018.10.67 PMC6531650

[450] Teschke, R. & Eickhoff, A. Wilson disease: copper-mediated cuproptosis, iron-related ferroptosis, and clinical highlights, with comprehensive and critical analysis update. *Int. J. Mol. Sci.* **25**, 4753 (2024). 10.3390/ijms25094753 PMC11084815 38731973

[451] Gromadzka, G., Wierzbicka, D., Litwin, T. & Przybylkowski, A. Iron metabolism is disturbed and anti-copper treatment improves but does not normalize iron metabolism in Wilson’s disease. *Biometals* **34**, 407–414 (2021). 33555495 10.1007/s10534-021-00289-x PMC7940312

[452] Sun, X. et al. Protective effect of curcumin on hepatolenticular degeneration through copper excretion and inhibition of ferroptosis. *Phytomedicine* **113**, 154539 (2023). 36898256 10.1016/j.phymed.2022.154539

[453] Ghobadi, M. et al. Ferulic acid ameliorates cell injuries, cognitive and motor impairments in cuprizone-induced demyelination model of multiple sclerosis. *Cell J.* **24**, 681–688 (2022). 36377218 10.22074/cellj.2022.8261 PMC9663966

[454] Wang, H. et al. Ferulic acid attenuates diabetes-induced cognitive impairment in rats via regulation of PTP1B and insulin signaling pathway. *Physiol. Behav.* **182**, 93–100 (2017). 28988132 10.1016/j.physbeh.2017.10.001

[455] Wang, X. et al. Ferulic acid activates SIRT1-mediated ferroptosis signaling pathway to improve cognition dysfunction in Wilson’s disease. *Neuropsychiatr. Dis. Treat.* **19**, 2681–2696 (2023). 38077239 10.2147/NDT.S443278 PMC10710261

[456] Duan, D. et al. Duchenne muscular dystrophy. *Nat. Rev. Dis. Prim.* **7**, 13 (2021). 33602943 10.1038/s41572-021-00248-3 PMC10557455

[457] Alves, F. M. et al. Iron overload and impaired iron handling contribute to the dystrophic pathology in models of Duchenne muscular dystrophy. *J. Cachexia Sarcopenia Muscle* **13**, 1541–1553 (2022). 35249268 10.1002/jcsm.12950 PMC9178167

[458] Bornman, L., Rossouw, H., Gericke, G. S. & Polla, B. S. Effects of iron deprivation on the pathology and stress protein expression in murine X-linked muscular dystrophy. *Biochem. Pharm.* **56**, 751–757 (1998). 9751080 10.1016/s0006-2952(98)00055-0

[459] van der Wal, H. H. et al. Iron deficiency in worsening heart failure is associated with reduced estimated protein intake, fluid retention, inflammation, and antiplatelet use. *Eur. Heart J.* **40**, 3616–3625 (2019). 31556953 10.1093/eurheartj/ehz680 PMC6868426

[460] van der Meer, P., van der Wal, H. H. & Melenovsky, V. Mitochondrial function, skeletal muscle metabolism, and iron deficiency in heart failure. *Circulation* **139**, 2399–2402 (2019). 31107619 10.1161/CIRCULATIONAHA.119.040134

[461] Wu, X., Li, Y., Zhang, S. & Zhou, X. Ferroptosis as a novel therapeutic target for cardiovascular disease. *Theranostics* **11**, 3052–3059 (2021). 33537073 10.7150/thno.54113 PMC7847684

[462] Ambrosy, A. P. et al. A reduced transferrin saturation is independently associated with excess morbidity and mortality in older adults with heart failure and incident anemia. *Int. J. Cardiol.* **309**, 95–99 (2020). 32201101 10.1016/j.ijcard.2020.03.020 PMC13282765

[463] Guo, Y. et al. Ferroptosis in cardiovascular diseases: current status, challenges, and future perspectives. *Biomolecules* **12**, 390 (2022). 10.3390/biom12030390 PMC8945958 35327582

[464] Baman, J. R. & Ahmad, F. S. Heart Failure. *JAMA* **324**, 1015 (2020). 32749448 10.1001/jama.2020.13310

[465] Fang, X. et al. Dietary intake of heme iron and risk of cardiovascular disease: a dose-response meta-analysis of prospective cohort studies. *Nutr. Metab. Cardiovasc. Dis.* **25**, 24–35 (2015). 25439662 10.1016/j.numecd.2014.09.002

[466] Zhang, H., Zhabyeyev, P., Wang, S. & Oudit, G. Y. Role of iron metabolism in heart failure: from iron deficiency to iron overload. *Biochim. Biophys. Acta Mol. Basis Dis.* **1865**, 1925–1937 (2019). 31109456 10.1016/j.bbadis.2018.08.030

[467] Xiong, Y. et al. Inhibition of ferroptosis reverses heart failure with preserved ejection fraction in mice. *J. Transl. Med.* **22**, 199 (2024). 38402404 10.1186/s12967-023-04734-y PMC10894491

[468] Gu, J. J. et al. Identification of ferroptosis-related genes in heart failure induced by transverse aortic constriction. *J. Inflamm. Res.* **16**, 4899–4912 (2023). 37927963 10.2147/JIR.S433387 PMC10625389

[469] Ito, J. et al. Iron derived from autophagy-mediated ferritin degradation induces cardiomyocyte death and heart failure in mice. *Elife* **10**, e62174 (2021). 10.7554/eLife.62174 PMC7853718 33526170

[470] Acoba, M. G. et al. The mitochondrial carrier SFXN1 is critical for complex III integrity and cellular metabolism. *Cell Rep.* **34**, 108869 (2021). 33730581 10.1016/j.celrep.2021.108869 PMC8048093

[471] Tang, M. et al. Ferritinophagy activation and sideroflexin1-dependent mitochondria iron overload is involved in apelin-13-induced cardiomyocytes hypertrophy. *Free Radic. Biol. Med.* **134**, 445–457 (2019). 30731113 10.1016/j.freeradbiomed.2019.01.052

[472] Omiya, S. et al. Downregulation of ferritin heavy chain increases labile iron pool, oxidative stress and cell death in cardiomyocytes. *J. Mol. Cell Cardiol.* **46**, 59–66 (2009). 18992754 10.1016/j.yjmcc.2008.09.714

[473] Zhang, X. et al. SLC7A11/xCT prevents cardiac hypertrophy by inhibiting ferroptosis. *Cardiovasc. Drugs Ther.* **36**, 437–447 (2022). 34259984 10.1007/s10557-021-07220-z

[474] Wang, J. et al. Pyroptosis and ferroptosis induced by mixed lineage kinase 3 (MLK3) signaling in cardiomyocytes are essential for myocardial fibrosis in response to pressure overload. *Cell Death Dis.* **11**, 574 (2020). 32710001 10.1038/s41419-020-02777-3 PMC7382480

[475] Chen, X., Xu, S., Zhao, C. & Liu, B. Role of TLR4/NADPH oxidase 4 pathway in promoting cell death through autophagy and ferroptosis during heart failure. *Biochem. Biophys. Res. Commun.* **516**, 37–43 (2019). 31196626 10.1016/j.bbrc.2019.06.015

[476] Liu, B. et al. Puerarin protects against heart failure induced by pressure overload through mitigation of ferroptosis. *Biochem. Biophys. Res. Commun.* **497**, 233–240 (2018). 29427658 10.1016/j.bbrc.2018.02.061

[477] Ma, S. et al. Canagliflozin mitigates ferroptosis and ameliorates heart failure in rats with preserved ejection fraction. *Naunyn Schmiedebergs Arch. Pharm.* **395**, 945–962 (2022). 10.1007/s00210-022-02243-1 PMC9276585 35476142

[478] Packer, M. Potential interactions when prescribing SGLT2 inhibitors and intravenous iron in combination in heart failure. *JACC Heart Fail.* **11**, 106–114 (2023). 36396554 10.1016/j.jchf.2022.10.004

[479] Yagi, M. et al. Improving lysosomal ferroptosis with NMN administration protects against heart failure. *Life Sci. Alliance* **6**, e202302116 (2023). 10.26508/lsa.202302116 PMC10551641 37793777

[480] Yang, X. et al. Ferroptosis in heart failure. *J. Mol. Cell Cardiol.* **173**, 141–153 (2022). 36273661 10.1016/j.yjmcc.2022.10.004 PMC11225968

[481] Bi et al. Characterization of ferroptosis-triggered pyroptotic signaling in heart failure. *Signal Transduct Target Ther*. 10.1038/s41392-024-01962-6 (2024). 10.1038/s41392-024-01962-6 PMC11427671 39327446

[482] Li, D. et al. Role of acetylation in doxorubicin-induced cardiotoxicity. *Redox Biol.* **46**, 102089 (2021). 34364220 10.1016/j.redox.2021.102089 PMC8350499

[483] Lyu, Y. L. et al. Topoisomerase IIbeta mediated DNA double-strand breaks: implications in doxorubicin cardiotoxicity and prevention by dexrazoxane. *Cancer Res.* **67**, 8839–8846 (2007). 17875725 10.1158/0008-5472.CAN-07-1649

[484] Koleini, N. et al. Oxidized phospholipids in Doxorubicin-induced cardiotoxicity. *Chem. Biol. Interact.* **303**, 35–39 (2019). 30707978 10.1016/j.cbi.2019.01.032

[485] Ichikawa, Y. et al. Cardiotoxicity of doxorubicin is mediated through mitochondrial iron accumulation. *J. Clin. Investig.* **124**, 617–630 (2014). 24382354 10.1172/JCI72931 PMC3904631

[486] Tadokoro, T. et al. Mitochondria-dependent ferroptosis plays a pivotal role in doxorubicin cardiotoxicity. *JCI Insight* **5**, e132747 (2020). 10.1172/jci.insight.132747 PMC7253028 32376803

[487] Singh, P. et al. Sulforaphane protects the heart from doxorubicin-induced toxicity. *Free Radic. Biol. Med.* **86**, 90–101 (2015). 26025579 10.1016/j.freeradbiomed.2015.05.028 PMC4554811

[488] Koh, J. S. et al. Protective effect of cilostazol against doxorubicin-induced cardiomyopathy in mice. *Free Radic. Biol. Med.* **89**, 54–61 (2015). 26191652 10.1016/j.freeradbiomed.2015.07.016

[489] Shi, S. et al. Role of oxidative stress and inflammation-related signaling pathways in doxorubicin-induced cardiomyopathy. *Cell Commun. Signal.* **21**, 61 (2023). 36918950 10.1186/s12964-023-01077-5 PMC10012797

[490] Fang, X. et al. Ferroptosis as a target for protection against cardiomyopathy. *Proc. Natl. Acad. Sci. USA* **116**, 2672–2680 (2019). 30692261 10.1073/pnas.1821022116 PMC6377499

[491] Behring, J. B. et al. Does reversible cysteine oxidation link the Western diet to cardiac dysfunction? *FASEB J.* **28**, 1975–1987 (2014). 24469991 10.1096/fj.13-233445 PMC4046179

[492] Li, N. et al. Ferroptosis and its emerging roles in cardiovascular diseases. *Pharm. Res.* **166**, 105466 (2021). 10.1016/j.phrs.2021.105466 33548489

[493] Wang, N. et al. HSF1 functions as a key defender against palmitic acid-induced ferroptosis in cardiomyocytes. *J. Mol. Cell Cardiol.* **150**, 65–76 (2021). 33098823 10.1016/j.yjmcc.2020.10.010

[494] Berdaweel, I. A. et al. Iron scavenging and suppression of collagen cross-linking underlie antifibrotic effects of carnosine in the heart with obesity. *Front. Pharm.* **14**, 1275388 (2023). 10.3389/fphar.2023.1275388 PMC10859874 38348353

[495] Uriho, A. et al. Benefits of blended oil consumption over other sources of lipids on the cardiovascular system in obese rats. *Food Funct.* **10**, 5290–5301 (2019). 31475703 10.1039/c9fo01353a

[496] Ramprasath, T. & Selvam, G. S. Potential impact of genetic variants in Nrf2 regulated antioxidant genes and risk prediction of diabetes and associated cardiac complications. *Curr. Med. Chem.* **20**, 4680–4693 (2013). 23834171 10.2174/09298673113209990154

[497] Lejay, A. et al. Ischemia reperfusion injury, ischemic conditioning and diabetes mellitus. *J. Mol. Cell Cardiol.* **91**, 11–22 (2016). 26718721 10.1016/j.yjmcc.2015.12.020

[498] Dillmann, W. H. Diabetic cardiomyopathy. *Circ. Res.* **124**, 1160–1162 (2019). 30973809 10.1161/CIRCRESAHA.118.314665 PMC6578576

[499] Wu, Y. et al. Retinol dehydrogenase 10 reduction mediated retinol metabolism disorder promotes diabetic cardiomyopathy in male mice. *Nat. Commun.* **14**, 1181 (2023). 36864033 10.1038/s41467-023-36837-x PMC9981688

[500] Katunga, L. A. et al. Obesity in a model of gpx4 haploinsufficiency uncovers a causal role for lipid-derived aldehydes in human metabolic disease and cardiomyopathy. *Mol. Metab.* **4**, 493–506 (2015). 26042203 10.1016/j.molmet.2015.04.001 PMC4443294

[501] Baseler, W. A. et al. Reversal of mitochondrial proteomic loss in Type 1 diabetic heart with overexpression of phospholipid hydroperoxide glutathione peroxidase. *Am. J. Physiol. Regul. Integr. Comp. Physiol.* **304**, R553–R565 (2013). 23408027 10.1152/ajpregu.00249.2012 PMC3627941

[502] Wang, X. et al. Ferroptosis is essential for diabetic cardiomyopathy and is prevented by sulforaphane via AMPK/NRF2 pathways. *Acta Pharm. Sin. B* **12**, 708–722 (2022). 35256941 10.1016/j.apsb.2021.10.005 PMC8897044

[503] Gu, J. et al. Metallothionein is downstream of Nrf2 and partially mediates sulforaphane prevention of diabetic cardiomyopathy. *Diabetes* **66**, 529–542 (2017). 27903744 10.2337/db15-1274 PMC5248986

[504] Wei, Z., Shaohuan, Q., Pinfang, K. & Chao, S. Curcumin attenuates ferroptosis-induced myocardial injury in diabetic cardiomyopathy through the Nrf2 pathway. *Cardiovasc. Ther.* **2022**, 3159717 (2022). 35909950 10.1155/2022/3159717 PMC9307414

[505] Li, F., Hu, Z., Huang, Y. & Zhan, H. Dexmedetomidine ameliorates diabetic cardiomyopathy by inhibiting ferroptosis through the Nrf2/GPX4 pathway. *J. Cardiothorac. Surg.* **18**, 223 (2023). 37430319 10.1186/s13019-023-02300-7 PMC10334540

[506] Wu, S. et al. Upregulation of NF-kappaB by USP24 aggravates ferroptosis in diabetic cardiomyopathy. *Free Radic. Biol. Med.* **210**, 352–366 (2024). 38056575 10.1016/j.freeradbiomed.2023.11.032

[507] Li, X. et al. Astragaloside IV attenuates myocardial dysfunction in diabetic cardiomyopathy rats through downregulation of CD36-mediated ferroptosis. *Phytother. Res.* **37**, 3042–3056 (2023). 36882189 10.1002/ptr.7798

[508] Tang, Y. J. et al. Irisin attenuates type 1 diabetic cardiomyopathy by anti-ferroptosis via SIRT1-mediated deacetylation of p53. *Cardiovasc. Diabetol.* **23**, 116 (2024). 38566123 10.1186/s12933-024-02183-5 PMC10985893

[509] Chen, Z. et al. Nicorandil alleviates cardiac microvascular ferroptosis in diabetic cardiomyopathy: role of the mitochondria-localized AMPK-Parkin-ACSL4 signaling pathway. *Pharm. Res.* **200**, 107057 (2024). 10.1016/j.phrs.2024.107057 38218357

[510] Iqbal, S., Jabeen, F., Kahwa, I. & Omara, T. Suberosin alleviates thiazolidinedione-induced cardiomyopathy in diabetic rats by inhibiting ferroptosis via modulation of ACSL4-LPCAT3 and PI3K-AKT signaling pathways. *Cardiovasc. Toxicol.* **23**, 295–304 (2023). 37676618 10.1007/s12012-023-09804-7

[511] Kilic, A. et al. Outcomes of the first 1300 adult heart transplants in the united states after the allocation policy change. *Circulation* **141**, 1662–1664 (2020). 32421414 10.1161/CIRCULATIONAHA.119.045354

[512] Zhu, Y. et al. Cirbp suppression compromises DHODH-mediated ferroptosis defense and attenuates hypothermic cardioprotection in an aged donor transplantation model. *J. Clin. Investig.* **134**, e175645 (2024). 10.1172/JCI175645 PMC11060748 38690728

[513] Li, W. et al. Ferroptotic cell death and TLR4/Trif signaling initiate neutrophil recruitment after heart transplantation. *J. Clin. Investig.* **129**, 2293–2304 (2019). 30830879 10.1172/JCI126428 PMC6546457

[514] Zhu, Y. et al. Type A aortic dissection-experience over 5 decades: JACC Historical Breakthroughs In Perspective. *J. Am. Coll. Cardiol.* **76**, 1703–1713 (2020). 33004136 10.1016/j.jacc.2020.07.061

[515] Rylski, B., Schilling, O. & Czerny, M. Acute aortic dissection: evidence, uncertainties, and future therapies. *Eur. Heart J.* **44**, 813–821 (2023). 36540036 10.1093/eurheartj/ehac757

[516] Li, N. et al. Targeting ferroptosis as a novel approach to alleviate aortic dissection. *Int J. Biol. Sci.* **18**, 4118–4134 (2022). 35844806 10.7150/ijbs.72528 PMC9274489

[517] Chen, Y. et al. BRD4770 functions as a novel ferroptosis inhibitor to protect against aortic dissection. *Pharm. Res.* **177**, 106122 (2022). 10.1016/j.phrs.2022.106122 35149187

[518] Chen, Y., Yi, X., Wei, X. & Jiang, D. S. Ferroptosis: a novel pathological mechanism of aortic dissection. *Pharm. Res.* **182**, 106351 (2022). 10.1016/j.phrs.2022.106351 35835368

[519] Liao, M. et al. METTL3-mediated m6A modification of NORAD inhibits the ferroptosis of vascular smooth muscle cells to attenuate the aortic dissection progression in an YTHDF2-dependent manner. *Mol. Cell. Biochem.*10.1007/s11010-024-04930-4 (2024). 10.1007/s11010-024-04930-4 38383916

[520] Puylaert, P. et al. Regulated necrosis in atherosclerosis. *Arterioscler. Thromb. Vasc. Biol.* **42**, 1283–1306 (2022). 36134566 10.1161/ATVBAHA.122.318177

[521] Robichaud, S. et al. Identification of novel lipid droplet factors that regulate lipophagy and cholesterol efflux in macrophage foam cells. *Autophagy* **17**, 3671–3689 (2021). 33590792 10.1080/15548627.2021.1886839 PMC8632324

[522] Ouimet, M. & Marcel, Y. L. Regulation of lipid droplet cholesterol efflux from macrophage foam cells. *Arterioscler. Thromb. Vasc. Biol.* **32**, 575–581 (2012). 22207731 10.1161/ATVBAHA.111.240705

[523] Moore, K. J. & Tabas, I. Macrophages in the pathogenesis of atherosclerosis. *Cell* **145**, 341–355 (2011). 21529710 10.1016/j.cell.2011.04.005 PMC3111065

[524] Singh, R. K. et al. TLR4 (Toll-Like Receptor 4)-dependent signaling drives extracellular catabolism of LDL (low-density lipoprotein) aggregates. *Arterioscler. Thromb. Vasc. Biol.* **40**, 86–102 (2020). 31597445 10.1161/ATVBAHA.119.313200 PMC6928397

[525] Yan, J. & Horng, T. Lipid metabolism in regulation of macrophage functions. *Trends Cell Biol.* **30**, 979–989 (2020). 33036870 10.1016/j.tcb.2020.09.006

[526] Remmerie, A. & Scott, C. L. Macrophages and lipid metabolism. *Cell Immunol.* **330**, 27–42 (2018). 29429624 10.1016/j.cellimm.2018.01.020 PMC6108423

[527] Haschka, D., Hoffmann, A. & Weiss, G. Iron in immune cell function and host defense. *Semin. Cell Dev. Biol.* **115**, 27–36 (2021). 33386235 10.1016/j.semcdb.2020.12.005

[528] Cai, J. et al. Iron accumulation in macrophages promotes the formation of foam cells and development of atherosclerosis. *Cell Biosci.* **10**, 137 (2020). 33292517 10.1186/s13578-020-00500-5 PMC7691057

[529] Malhotra, R. et al. Hepcidin deficiency protects against atherosclerosis. *Arterioscler. Thromb. Vasc. Biol.* **39**, 178–187 (2019). 30587002 10.1161/ATVBAHA.118.312215 PMC6344241

[530] Ji, J. et al. Low expression of ferroxidases is implicated in the iron retention in human atherosclerotic plaques. *Biochem. Biophys. Res. Commun.* **464**, 1134–1138 (2015). 26208458 10.1016/j.bbrc.2015.07.091

[531] Chiu, M. H. et al. Coronary artery disease in post-menopausal women: are there appropriate means of assessment? *Clin. Sci.* **132**, 1937–1952 (2018). 10.1042/CS20180067 30185615

[532] Bai, T. et al. Inhibition of ferroptosis alleviates atherosclerosis through attenuating lipid peroxidation and endothelial dysfunction in mouse aortic endothelial cell. *Free Radic. Biol. Med.* **160**, 92–102 (2020). 32768568 10.1016/j.freeradbiomed.2020.07.026

[533] Ito, F., Sono, Y. & Ito, T. Measurement and clinical significance of lipid peroxidation as a biomarker of oxidative stress: oxidative stress in diabetes, atherosclerosis, and chronic inflammation. *Antioxidants* **8**, 72 (2019). 10.3390/antiox8030072 PMC6466575 30934586

[534] Zhou, Y. et al. Verification of ferroptosis and pyroptosis and identification of PTGS2 as the hub gene in human coronary artery atherosclerosis. *Free Radic. Biol. Med.* **171**, 55–68 (2021). 33974977 10.1016/j.freeradbiomed.2021.05.009

[535] Feng, H. & Stockwell, B. R. Unsolved mysteries: how does lipid peroxidation cause ferroptosis? *PLoS Biol.* **16**, e2006203 (2018). 29795546 10.1371/journal.pbio.2006203 PMC5991413

[536] Kaisar, M. A., Sivandzade, F., Bhalerao, A. & Cucullo, L. Conventional and electronic cigarettes dysregulate the expression of iron transporters and detoxifying enzymes at the brain vascular endothelium: in vivo evidence of a gender-specific cellular response to chronic cigarette smoke exposure. *Neurosci. Lett.* **682**, 1–9 (2018). 29879439 10.1016/j.neulet.2018.05.045 PMC6102071

[537] Yang, K., Song, H. & Yin, D. PDSS2 Inhibits The Ferroptosis Of Vascular Endothelial Cells In Atherosclerosis By Activating Nrf2. *J. Cardiovasc. Pharm.* **77**, 767–776 (2021). 10.1097/FJC.0000000000001030 PMC8274586 33929387

[538] Li, C. et al. CTRP5 promotes transcytosis and oxidative modification of low-density lipoprotein and the development of atherosclerosis. *Atherosclerosis* **278**, 197–209 (2018). 30300788 10.1016/j.atherosclerosis.2018.09.037

[539] Liu, W. et al. Erythroid lineage Jak2V617F expression promotes atherosclerosis through erythrophagocytosis and macrophage ferroptosis. *J. Clin. Investig.* **132**, e155724 (2022). 10.1172/JCI155724 PMC9246386 35587375

[540] Long, H., Zhu, W., Wei, L. & Zhao, J. Iron homeostasis imbalance and ferroptosis in brain diseases. *MedComm* **4**, e298 (2023). 37377861 10.1002/mco2.298 PMC10292684

[541] Wei, Z. et al. Broadening horizons: ferroptosis as a new target for traumatic brain injury. *Burns Trauma* **12**, tkad051 (2024). 38250705 10.1093/burnst/tkad051 PMC10799763

[542] Scheltens, P. et al. Alzheimer’s disease. *Lancet* **397**, 1577–1590 (2021). 33667416 10.1016/S0140-6736(20)32205-4 PMC8354300

[543] Derry, P. J. et al. Revisiting the intersection of amyloid, pathologically modified tau and iron in Alzheimer’s disease from a ferroptosis perspective. *Prog. Neurobiol.* **184**, 101716 (2020). 31604111 10.1016/j.pneurobio.2019.101716 PMC7850812

[544] Nikseresht, S., Bush, A. I. & Ayton, S. Treating Alzheimer’s disease by targeting iron. *Br. J. Pharm.* **176**, 3622–3635 (2019). 10.1111/bph.14567 PMC6715619 30632143

[545] Yong, Y. Y. et al. Penthorum chinense Pursh inhibits ferroptosis in cellular and Caenorhabditis elegans models of Alzheimer’s disease. *Phytomedicine* **127**, 155463 (2024). 38452694 10.1016/j.phymed.2024.155463

[546] Masaldan, S., Belaidi, A. A., Ayton, S. & Bush, A. I. Cellular senescence and iron dyshomeostasis in Alzheimer’s disease. *Pharmaceuticals* **12**, 93 (2019). 10.3390/ph12020093 PMC6630536 31248150

[547] Ayton, S. et al. Regional brain iron associated with deterioration in Alzheimer’s disease: a large cohort study and theoretical significance. *Alzheimers Dement.* **17**, 1244–1256 (2021). 33491917 10.1002/alz.12282 PMC9701539

[548] Ashraf, A., Jeandriens, J., Parkes, H. G. & So, P. W. Iron dyshomeostasis, lipid peroxidation and perturbed expression of cystine/glutamate antiporter in Alzheimer’s disease: evidence of ferroptosis. *Redox Biol.* **32**, 101494 (2020). 32199332 10.1016/j.redox.2020.101494 PMC7083890

[549] Jakaria, M., Belaidi, A. A., Bush, A. I. & Ayton, S. Ferroptosis as a mechanism of neurodegeneration in Alzheimer’s disease. *J. Neurochem.* **159**, 804–825 (2021). 34553778 10.1111/jnc.15519

[550] Plascencia-Villa, G. & Perry, G. Preventive and therapeutic strategies in Alzheimer’s disease: focus on oxidative stress, redox metals, and ferroptosis. *Antioxid. Redox Signal.* **34**, 591–610 (2021). 32486897 10.1089/ars.2020.8134 PMC8098758

[551] Feng, L. et al. Ferroptosis mechanism and Alzheimer’s disease. *Neural Regen. Res.* **19**, 1741–1750 (2024). 38103240 10.4103/1673-5374.389362 PMC10960301

[552] Gleason, A. & Bush, A. I. Iron and ferroptosis as therapeutic targets in Alzheimer’s disease. *Neurotherapeutics* **18**, 252–264 (2021). 33111259 10.1007/s13311-020-00954-y PMC8116360

[553] Pal, A. et al. Iron in Alzheimer’s disease: from physiology to disease disabilities. *Biomolecules* **12**, 1248 (2022). 10.3390/biom12091248 PMC9496246 36139084

[554] Dang, Y. et al. FTH1- and SAT1-induced astrocytic ferroptosis is involved in Alzheimer’s disease: evidence from single-cell transcriptomic analysis. *Pharmaceuticals* **15**, 1177 (2022). 10.3390/ph15101177 PMC9610574 36297287

[555] Belaidi, A. A. et al. Apolipoprotein E potently inhibits ferroptosis by blocking ferritinophagy. *Mol. Psychiatry* **29**, 211–220 (2024). 35484240 10.1038/s41380-022-01568-w PMC9757994

[556] Huang, L. et al. Intracellular amyloid toxicity induces oxytosis/ferroptosis regulated cell death. *Cell Death Dis.* **11**, 828 (2020). 33024077 10.1038/s41419-020-03020-9 PMC7538552

[557] Naderi, S. et al. Role of amyloid beta (25-35) neurotoxicity in the ferroptosis and necroptosis as modalities of regulated cell death in Alzheimer’s disease. *Neurotoxicology* **94**, 71–86 (2023). 36347329 10.1016/j.neuro.2022.11.003

[558] Dar, N. J. et al. Oxytosis/ferroptosis in neurodegeneration: the underlying role of master regulator glutathione peroxidase 4 (GPX4). *Mol. Neurobiol.* **61**, 1507–1526 (2024). 37725216 10.1007/s12035-023-03646-8

[559] Hambright, W. S. et al. Ablation of ferroptosis regulator glutathione peroxidase 4 in forebrain neurons promotes cognitive impairment and neurodegeneration. *Redox Biol.* **12**, 8–17 (2017). 28212525 10.1016/j.redox.2017.01.021 PMC5312549

[560] Greenough, M. A. et al. Selective ferroptosis vulnerability due to familial Alzheimer’s disease presenilin mutations. *Cell Death Differ.* **29**, 2123–2136 (2022). 35449212 10.1038/s41418-022-01003-1 PMC9613996

[561] Park, M. W. et al. NOX4 promotes ferroptosis of astrocytes by oxidative stress-induced lipid peroxidation via the impairment of mitochondrial metabolism in Alzheimer’s diseases. *Redox Biol.* **41**, 101947 (2021). 33774476 10.1016/j.redox.2021.101947 PMC8027773

[562] Lane, D. J. R., Alves, F., Ayton, S. J. & Bush, A. I. Striking a NRF2: the rusty and rancid vulnerabilities toward ferroptosis in Alzheimer’s disease. *Antioxid. Redox Signal.* **39**, 141–161 (2023). 37212212 10.1089/ars.2023.0318

[563] Qu, Z. et al. Transcription factor NRF2 as a promising therapeutic target for Alzheimer’s disease. *Free Radic. Biol. Med.* **159**, 87–102 (2020). 32730855 10.1016/j.freeradbiomed.2020.06.028

[564] Tang, Z. et al. NRF2 Deficiency promotes ferroptosis of astrocytes mediated by oxidative stress in Alzheimer’s disease. *Mol. Neurobiol.*10.1007/s12035-024-04023-9 (2024). 10.1007/s12035-024-04023-9 38401046

[565] Li, J. et al. beta-amyloid protein induces mitophagy-dependent ferroptosis through the CD36/PINK/PARKIN pathway leading to blood-brain barrier destruction in Alzheimer’s disease. *Cell Biosci.* **12**, 69 (2022). 35619150 10.1186/s13578-022-00807-5 PMC9134700

[566] Wang, M. et al. Revisiting the intersection of microglial activation and neuroinflammation in Alzheimer’s disease from the perspective of ferroptosis. *Chem. Biol. Interact.* **375**, 110387 (2023). 36758888 10.1016/j.cbi.2023.110387

[567] Wang, Y. et al. Pharmacological Inhibition of Ferroptosis as a Therapeutic Target for Neurodegenerative Diseases and Strokes. *Adv. Sci.* **10**, e2300325 (2023). 10.1002/advs.202300325 PMC10460905 37341302

[568] Li, X., Wang, X., Huang, B. & Huang, R. Sennoside A restrains TRAF6 level to modulate ferroptosis, inflammation and cognitive impairment in aging mice with Alzheimer’s disease. *Int. Immunopharmacol.* **120**, 110290 (2023). 37216800 10.1016/j.intimp.2023.110290

[569] Baruah, P. et al. A natural polyphenol activates and enhances GPX4 to mitigate amyloid-beta induced ferroptosis in Alzheimer’s disease. *Chem. Sci.* **14**, 9427–9438 (2023). 37712018 10.1039/d3sc02350h PMC10498722

[570] Zhai, L. et al. Paeoniflorin suppresses neuronal ferroptosis to improve the cognitive behaviors in Alzheimer’s disease mice. *Phytother. Res.* **37**, 4791–4800 (2023). 37448137 10.1002/ptr.7946

[571] Li, L. et al. Eriodictyol ameliorates cognitive dysfunction in APP/PS1 mice by inhibiting ferroptosis via vitamin D receptor-mediated Nrf2 activation. *Mol. Med.* **28**, 11 (2022). 35093024 10.1186/s10020-022-00442-3 PMC8800262

[572] Wang, C. et al. Forsythoside A mitigates Alzheimer’s-like pathology by inhibiting ferroptosis-mediated neuroinflammation via Nrf2/GPX4 axis activation. *Int. J. Biol. Sci.* **18**, 2075–2090 (2022). 35342364 10.7150/ijbs.69714 PMC8935224

[573] Cong, L. et al. On the role of synthesized hydroxylated chalcones as dual functional amyloid-beta aggregation and ferroptosis inhibitors for potential treatment of Alzheimer’s disease. *Eur. J. Med. Chem.* **166**, 11–21 (2019). 30684867 10.1016/j.ejmech.2019.01.039

[574] Gong, Y. et al. Curculigoside, a traditional Chinese medicine monomer, ameliorates oxidative stress in Alzheimer’s disease mouse model via suppressing ferroptosis. *Phytother. Res.* **38**, 2462–2481 (2024). 38444049 10.1002/ptr.8152

[575] Yang, S. et al. Salidroside attenuates neuronal ferroptosis by activating the Nrf2/HO1 signaling pathway in Abeta(1-42)-induced Alzheimer’s disease mice and glutamate-injured HT22 cells. *Chin. Med.* **17**, 82 (2022). 35787281 10.1186/s13020-022-00634-3 PMC9254541

[576] Yang, S. et al. Salidroside alleviates cognitive impairment by inhibiting ferroptosis via activation of the Nrf2/GPX4 axis in SAMP8 mice. *Phytomedicine* **114**, 154762 (2023). 36965372 10.1016/j.phymed.2023.154762

[577] Gao, Y. et al. Tetrahydroxy stilbene glycoside ameliorates Alzheimer’s disease in APP/PS1 mice via glutathione peroxidase related ferroptosis. *Int. Immunopharmacol.* **99**, 108002 (2021). 34333354 10.1016/j.intimp.2021.108002

[578] Youssef, M. A. M., Mohamed, T. M., Bakry, A. A. & El-Keiy, M. M. Synergistic effect of spermidine and ciprofloxacin against Alzheimer’s disease in male rat via ferroptosis modulation. *Int. J. Biol. Macromol.* **263**, 130387 (2024). 38401586 10.1016/j.ijbiomac.2024.130387

[579] Moorthy, H. et al. Polycatechols inhibit ferroptosis and modulate tau liquid-liquid phase separation to mitigate Alzheimer’s disease. *Mater. Horiz.* **11**, 3082-3089 (2024). 10.1039/d4mh00023d 38647314

[580] Sun, Y. et al. Inhibition of ferroptosis through regulating neuronal calcium homeostasis: an emerging therapeutic target for Alzheimer’s disease. *Ageing Res. Rev.* **87**, 101899 (2023). 36871781 10.1016/j.arr.2023.101899

[581] Zhang, Y. H. et al. alpha-Lipoic acid improves abnormal behavior by mitigation of oxidative stress, inflammation, ferroptosis, and tauopathy in P301S Tau transgenic mice. *Redox Biol.* **14**, 535–548 (2018). 29126071 10.1016/j.redox.2017.11.001 PMC5684493

[582] Li, C. et al. Tenuifolin in the prevention of Alzheimer’s disease-like phenotypes: investigation of the mechanisms from the perspectives of calpain system, ferroptosis, and apoptosis. *Phytother. Res.*10.1002/ptr.7930 (2023). 10.1002/ptr.7930 37364988

[583] Tolosa, E., Garrido, A., Scholz, S. W. & Poewe, W. Challenges in the diagnosis of Parkinson’s disease. *Lancet Neurol.* **20**, 385–397 (2021). 33894193 10.1016/S1474-4422(21)00030-2 PMC8185633

[584] Liu, L., Cui, Y., Chang, Y. Z. & Yu, P. Ferroptosis-related factors in the substantia nigra are associated with Parkinson’s disease. *Sci. Rep.* **13**, 15365 (2023). 37717088 10.1038/s41598-023-42574-4 PMC10505210

[585] Mahoney-Sanchez, L. et al. Ferroptosis and its potential role in the physiopathology of Parkinson’s Disease. *Prog. Neurobiol.* **196**, 101890 (2021). 32726602 10.1016/j.pneurobio.2020.101890

[586] Martin-Bastida, A. et al. Motor associations of iron accumulation in deep grey matter nuclei in Parkinson’s disease: a cross-sectional study of iron-related magnetic resonance imaging susceptibility. *Eur. J. Neurol.* **24**, 357–365 (2017). 27982501 10.1111/ene.13208

[587] Rhodes, S. L. et al. Pooled analysis of iron-related genes in Parkinson’s disease: association with transferrin. *Neurobiol. Dis.* **62**, 172–178 (2014). 24121126 10.1016/j.nbd.2013.09.019 PMC3968945

[588] Mastroberardino, P. G. et al. A novel transferrin/TfR2-mediated mitochondrial iron transport system is disrupted in Parkinson’s disease. *Neurobiol. Dis.* **34**, 417–431 (2009). 19250966 10.1016/j.nbd.2009.02.009 PMC2784936

[589] Milanese, C. et al. Gender biased neuroprotective effect of Transferrin Receptor 2 deletion in multiple models of Parkinson’s disease. *Cell Death Differ.* **28**, 1720–1732 (2021). 33323945 10.1038/s41418-020-00698-4 PMC8166951

[590] Agostini, F., Bubacco, L., Chakrabarti, S. & Bisaglia, M. Alpha-synuclein toxicity in drosophila melanogaster is enhanced by the presence of iron: implications for Parkinson’s Disease. *Antioxidants* **12**, 261 (2023). 10.3390/antiox12020261 PMC9952566 36829820

[591] Deas, E. et al. Alpha-synuclein oligomers interact with metal ions to induce oxidative stress and neuronal death in Parkinson’s Disease. *Antioxid. Redox Signal.* **24**, 376–391 (2016). 26564470 10.1089/ars.2015.6343 PMC4999647

[592] Ludtmann, M. H. R. et al. alpha-synuclein oligomers interact with ATP synthase and open the permeability transition pore in Parkinson’s disease. *Nat. Commun.* **9**, 2293 (2018). 29895861 10.1038/s41467-018-04422-2 PMC5997668

[593] Lv, Q. K. et al. Melatonin MT1 receptors regulate the Sirt1/Nrf2/Ho-1/Gpx4 pathway to prevent alpha-synuclein-induced ferroptosis in Parkinson’s disease. *J. Pineal Res.* **76**, e12948 (2024). 38488331 10.1111/jpi.12948

[594] Wang, M. et al. Identifying the potential genes in alpha synuclein driving ferroptosis of Parkinson’s disease. *Sci. Rep.* **13**, 16893 (2023). 37803093 10.1038/s41598-023-44124-4 PMC10558439

[595] Wang, D. et al. Antiferroptotic activity of non-oxidative dopamine. *Biochem. Biophys. Res. Commun.* **480**, 602–607 (2016). 27793671 10.1016/j.bbrc.2016.10.099

[596] Maniscalchi, A. et al. New insights on neurodegeneration triggered by iron accumulation: intersections with neutral lipid metabolism, ferroptosis, and motor impairment. *Redox Biol.* **71**, 103074 (2024). 38367511 10.1016/j.redox.2024.103074 PMC10879836

[597] Pang, P., Zhang, S., Fan, X. & Zhang, S. Knockdown of fat mass and obesity alleviates the ferroptosis in Parkinson’s disease through m6A-NRF2-dependent manner. *Cell Biol. Int.* **48**, 431–439 (2024). 38180302 10.1002/cbin.12118

[598] Tian, Y. et al. FTH1 inhibits ferroptosis through ferritinophagy in the 6-OHDA model of Parkinson’s disease. *Neurotherapeutics* **17**, 1796–1812 (2020). 32959272 10.1007/s13311-020-00929-z PMC7851296

[599] Fu, X., Qu, L., Xu, H. & Xie, J. Ndfip1 protected dopaminergic neurons via regulating mitochondrial function and ferroptosis in Parkinson’s disease. *Exp. Neurol.* **375**, 114724 (2024). 38365133 10.1016/j.expneurol.2024.114724

[600] Vallerga, C. L. et al. Analysis of DNA methylation associates the cystine-glutamate antiporter SLC7A11 with risk of Parkinson’s disease. *Nat. Commun.* **11**, 1238 (2020). 32144264 10.1038/s41467-020-15065-7 PMC7060318

[601] Bouchaoui, H. et al. ACSL4 and the lipoxygenases 15/15B are pivotal for ferroptosis induced by iron and PUFA dyshomeostasis in dopaminergic neurons. *Free Radic. Biol. Med.* **195**, 145–157 (2023). 36581060 10.1016/j.freeradbiomed.2022.12.086

[602] Tang, F. et al. Inhibition of ACSL4 alleviates parkinsonism phenotypes by reduction of lipid reactive oxygen species. *Neurotherapeutics* **20**, 1154–1166 (2023). 37133631 10.1007/s13311-023-01382-4 PMC10457271

[603] Xia, Y. et al. Inhibition of ferroptosis underlies EGCG mediated protection against Parkinson’s disease in a Drosophila model. *Free Radic. Biol. Med.* **211**, 63–76 (2024). 38092273 10.1016/j.freeradbiomed.2023.12.005

[604] Shen, J. et al. Salidroside Mediated the Nrf2/GPX4 pathway to attenuates ferroptosis in Parkinson’s disease. *Neurochem. Res.* **49**, 1291–1305 (2024). 38424396 10.1007/s11064-024-04116-w PMC10991011

[605] Kong, L. et al. Granulathiazole A protects 6-OHDA-induced Parkinson’s disease from ferroptosis via activating Nrf2/HO-1 pathway. *Bioorg. Chem.* **147**, 107399 (2024). 38678778 10.1016/j.bioorg.2024.107399

[606] Li, Q. M. et al. Buddlejasaponin IVb ameliorates ferroptosis of dopaminergic neuron by suppressing IRP2-mediated iron overload in Parkinson’s disease. *J. Ethnopharmacol.* **319**, 117196 (2024). 37717841 10.1016/j.jep.2023.117196

[607] Liu, T. et al. Rapamycin reverses ferroptosis by increasing autophagy in MPTP/MPP(+)-induced models of Parkinson’s disease. *Neural Regen. Res.* **18**, 2514–2519 (2023). 37282484 10.4103/1673-5374.371381 PMC10360095

[608] Li, K. et al. ALOX5 inhibition protects against dopaminergic neurons undergoing ferroptosis. *Pharm. Res.* **193**, 106779 (2023). 10.1016/j.phrs.2023.106779 37121496

[609] Li, M. et al. Neuroprotective effects of morroniside from Cornus officinalis sieb. Et zucc against Parkinson’s disease via inhibiting oxidative stress and ferroptosis. *BMC Complement. Med. Ther.* **23**, 218 (2023). 37393274 10.1186/s12906-023-03967-0 PMC10314491

[610] Zhang, X. et al. Targeting NKAalpha1 to treat Parkinson’s disease through inhibition of mitophagy-dependent ferroptosis. *Free Radic. Biol. Med.* **218**, 190–204 (2024). 38574977 10.1016/j.freeradbiomed.2024.04.002

[611] Chen, Y. et al. Mapping the research of ferroptosis in Parkinson’s Disease from 2013 to 2023: a scientometric review. *Drug Des. Dev. Ther.* **18**, 1053–1081 (2024). 10.2147/DDDT.S458026 PMC10999190 38585257

[612] Walker, F. O. Huntington’s disease. *Lancet* **369**, 218–228 (2007). 17240289 10.1016/S0140-6736(07)60111-1

[613] Tabrizi, S. J. et al. Potential disease-modifying therapies for Huntington’s disease: lessons learned and future opportunities. *Lancet Neurol.* **21**, 645–658 (2022). 35716694 10.1016/S1474-4422(22)00121-1 PMC7613206

[614] Dominguez, J. F. et al. Iron accumulation in the basal ganglia in Huntington’s disease: cross-sectional data from the IMAGE-HD study. *J. Neurol. Neurosurg. Psychiatry* **87**, 545–549 (2016). 25952334 10.1136/jnnp-2014-310183

[615] Muller, M. & Leavitt, B. R. Iron dysregulation in Huntington’s disease. *J. Neurochem.* **130**, 328–350 (2014). 24717009 10.1111/jnc.12739

[616] Ribeiro, M. et al. Glutathione redox cycle dysregulation in Huntington’s disease knock-in striatal cells. *Free Radic. Biol. Med.* **53**, 1857–1867 (2012). 22982598 10.1016/j.freeradbiomed.2012.09.004

[617] Brocardo, P. S., McGinnis, E., Christie, B. R. & Gil-Mohapel, J. Time-course analysis of protein and lipid oxidation in the brains of Yac128 Huntington’s disease transgenic mice. *Rejuvenation Res.* **19**, 140–148 (2016). 26371883 10.1089/rej.2015.1736

[618] Hatami, A. et al. Deuterium-reinforced linoleic acid lowers lipid peroxidation and mitigates cognitive impairment in the Q140 knock in mouse model of Huntington’s disease. *FEBS J.* **285**, 3002–3012 (2018). 29933522 10.1111/febs.14590

[619] Stockwell, B. R. et al. Ferroptosis: a regulated cell death nexus linking metabolism, redox biology, and disease. *Cell* **171**, 273–285 (2017). 28985560 10.1016/j.cell.2017.09.021 PMC5685180

[620] Mi, Y. et al. The emerging roles of ferroptosis in Huntington’s Disease. *Neuromol. Med.* **21**, 110–119 (2019). 10.1007/s12017-018-8518-6 30600476

[621] Dexter, D. T. et al. Alterations in the levels of iron, ferritin and other trace metals in Parkinson’s disease and other neurodegenerative diseases affecting the basal ganglia. *Brain* **114**, 1953–1975 (1991). 1832073 10.1093/brain/114.4.1953

[622] van den Bogaard, S. J., Dumas, E. M. & Roos, R. A. The role of iron imaging in Huntington’s disease. *Int. Rev. Neurobiol.* **110**, 241–250 (2013). 24209441 10.1016/B978-0-12-410502-7.00011-9

[623] Simmons, D. A. et al. Ferritin accumulation in dystrophic microglia is an early event in the development of Huntington’s disease. *Glia* **55**, 1074–1084 (2007). 17551926 10.1002/glia.20526

[624] Chen, J. et al. Iron accumulates in Huntington’s disease neurons: protection by deferoxamine. *PLoS One* **8**, e77023 (2013). 24146952 10.1371/journal.pone.0077023 PMC3795666

[625] Skouta, R. et al. Ferrostatins inhibit oxidative lipid damage and cell death in diverse disease models. *J. Am. Chem. Soc.* **136**, 4551–4556 (2014). 24592866 10.1021/ja411006a PMC3985476

[626] Song, S. et al. ALOX5-mediated ferroptosis acts as a distinct cell death pathway upon oxidative stress in Huntington’s disease. *Genes Dev.* **37**, 204–217 (2023). 36921996 10.1101/gad.350211.122 PMC10111862

[627] Monroe, S. M. & Harkness, K. L. Major depression and its recurrences: life course matters. *Annu Rev. Clin. Psychol.* **18**, 329–357 (2022). 35216520 10.1146/annurev-clinpsy-072220-021440

[628] Wang, L. et al. Targeting the ferroptosis crosstalk: novel alternative strategies for the treatment of major depressive disorder. *Gen. Psychiatr.* **36**, e101072 (2023). 37901286 10.1136/gpsych-2023-101072 PMC10603325

[629] Kim, J. & Wessling-Resnick, M. Iron and mechanisms of emotional behavior. *J. Nutr. Biochem.* **25**, 1101–1107 (2014). 25154570 10.1016/j.jnutbio.2014.07.003 PMC4253901

[630] Li, Y. et al. Metal ions in cerebrospinal fluid: associations with anxiety, depression, and insomnia among cigarette smokers. *CNS Neurosci. Ther.* **28**, 2141–2147 (2022). 36168907 10.1111/cns.13955 PMC9627395

[631] Cao, H. et al. Hippocampal proteomic analysis reveals activation of necroptosis and ferroptosis in a mouse model of chronic unpredictable mild stress-induced depression. *Behav. Brain Res.* **407**, 113261 (2021). 33775778 10.1016/j.bbr.2021.113261

[632] Jiao, H. et al. Traditional chinese formula xiaoyaosan alleviates depressive-like behavior in CUMS mice by regulating PEBP1-GPX4-mediated ferroptosis in the hippocampus. *Neuropsychiatr. Dis. Treat.* **17**, 1001–1019 (2021). 33854318 10.2147/NDT.S302443 PMC8039849

[633] Sowa-Kucma, M. et al. Lipid peroxidation and immune biomarkers are associated with major depression and its phenotypes, including treatment-resistant depression and melancholia. *Neurotox. Res.* **33**, 448–460 (2018). 29103192 10.1007/s12640-017-9835-5 PMC5766730

[634] Dang, R. et al. Edaravone ameliorates depressive and anxiety-like behaviors via Sirt1/Nrf2/HO-1/Gpx4 pathway. *J. Neuroinflamm.* **19**, 41 (2022). 10.1186/s12974-022-02400-6 PMC8822843 35130906

[635] Li, E. et al. Inhibition of ferroptosis alleviates chronic unpredictable mild stress-induced depression in mice via tsRNA-3029b. *Brain Res. Bull.* **204**, 110773 (2023). 37793597 10.1016/j.brainresbull.2023.110773

[636] Xu, C. et al. Alcohol exposure induces depressive and anxiety-like behaviors via activating ferroptosis in mice. *Int. J. Mol. Sci.* **23**, 13828 (2022). 10.3390/ijms232213828 PMC9698590 36430312

[637] Mao, L. et al. Arginine methylation of beta-catenin induced by PRMT2 aggravates LPS-Induced Cognitive Dysfunction And Depression-like Behaviors By Promoting Ferroptosis. *Mol. Neurobiol.*10.1007/s12035-024-04019-5 (2024). 10.1007/s12035-024-04019-5 38430350

[638] Yang, Z. et al. Di-Huang-Yin-Zi regulates P53/SLC7A11 signaling pathway to improve the mechanism of post-stroke depression. *J. Ethnopharmacol.* **319**, 117226 (2024). 37748635 10.1016/j.jep.2023.117226

[639] Wang, X. et al. Saikosaponin B2 ameliorates depression-induced microglia activation by inhibiting ferroptosis-mediated neuroinflammation and ER stress. *J. Ethnopharmacol.* **316**, 116729 (2023). 37277081 10.1016/j.jep.2023.116729

[640] Yang, R. et al. Gallic acid improves comorbid chronic pain and depression behaviors by inhibiting P2X7 receptor-mediated ferroptosis in the spinal cord of rats. *ACS Chem. Neurosci.* **14**, 667–676 (2023). 36719132 10.1021/acschemneuro.2c00532

[641] Wang, X. et al. DHA and EPA prevent seizure and depression-like behavior by inhibiting ferroptosis and neuroinflammation via different mode-of-actions in a pentylenetetrazole-induced kindling model in mice. *Mol. Nutr. Food Res.* **66**, e2200275 (2022). 36099650 10.1002/mnfr.202200275

[642] Shen, J. et al. Acupuncture alleviates CUMS-induced depression-like behaviors of rats by regulating oxidative stress, neuroinflammation and ferroptosis. *Brain Res.* **1826**, 148715 (2024). 38142722 10.1016/j.brainres.2023.148715

[643] Zhang, M. et al. Ketamine may exert rapid antidepressant effects through modulation of neuroplasticity, autophagy, and ferroptosis in the habenular nucleus. *Neuroscience* **506**, 29–37 (2022). 36280022 10.1016/j.neuroscience.2022.10.015

[644] Finnerup, N. B., Kuner, R. & Jensen, T. S. Neuropathic pain: from mechanisms to treatment. *Physiol. Rev.* **101**, 259–301 (2021). 32584191 10.1152/physrev.00045.2019

[645] Wang, H. et al. Ferroptosis is involved in the development of neuropathic pain and allodynia. *Mol. Cell Biochem.* **476**, 3149–3161 (2021). 33864570 10.1007/s11010-021-04138-w

[646] Tang, J. et al. TRIM28 Fosters microglia ferroptosis via autophagy modulation to enhance neuropathic pain and neuroinflammation. *Mol. Neurobiol.*10.1007/s12035-024-04133-4 (2024). 10.1007/s12035-024-04133-4 38647647

[647] Wan, K. et al. Electroacupuncture alleviates neuropathic pain by suppressing ferroptosis in dorsal root ganglion via SAT1/ALOX15 Signaling. *Mol. Neurobiol.* **60**, 6121–6132 (2023). 37421564 10.1007/s12035-023-03463-z

[648] Guo, Y. et al. Inhibition of ferroptosis-like cell death attenuates neuropathic pain reactions induced by peripheral nerve injury in rats. *Eur. J. Pain.* **25**, 1227–1240 (2021). 33497529 10.1002/ejp.1737

[649] Ding, Z. et al. Inhibition of spinal ferroptosis-like cell death alleviates hyperalgesia and spontaneous pain in a mouse model of bone cancer pain. *Redox Biol.* **62**, 102700 (2023). 37084690 10.1016/j.redox.2023.102700 PMC10141498

[650] Liu, T. et al. Methyl ferulic acid alleviates neuropathic pain by inhibiting Nox4-induced ferroptosis in dorsal root ganglia neurons in rats. *Mol. Neurobiol.* **60**, 3175–3189 (2023). 36813954 10.1007/s12035-023-03270-6

[651] Maas et al. Traumatic brain injury: integrated approaches to improve prevention, clinical care, and research. *Lancet Neurol.* **16**, 987–1048 (2017). 29122524 10.1016/S1474-4422(17)30371-X

[652] Shi, H. et al. Role of Toll-like receptor mediated signaling in traumatic brain injury. *Neuropharmacology* **145**, 259–267 (2019). 30075158 10.1016/j.neuropharm.2018.07.022

[653] Zhao, Z. A. et al. Cellular and molecular mechanisms in vascular repair after traumatic brain injury: a narrative review. *Burns Trauma* **11**, tkad033 (2023). 37675267 10.1093/burnst/tkad033 PMC10478165

[654] Fang, J. et al. Ferroptosis in brain microvascular endothelial cells mediates blood-brain barrier disruption after traumatic brain injury. *Biochem. Biophys. Res. Commun.* **619**, 34–41 (2022). 35728282 10.1016/j.bbrc.2022.06.040

[655] Geng, Z. et al. Ferroptosis and traumatic brain injury. *Brain Res. Bull.* **172**, 212–219 (2021). 33932492 10.1016/j.brainresbull.2021.04.023

[656] Liang, Y. et al. Deferoxamine reduces endothelial ferroptosis and protects cerebrovascular function after experimental traumatic brain injury. *Brain Res. Bull.* **207**, 110878 (2024). 38218407 10.1016/j.brainresbull.2024.110878

[657] Xie, B. S. et al. Inhibition of ferroptosis attenuates tissue damage and improves long-term outcomes after traumatic brain injury in mice. *CNS Neurosci. Ther.* **25**, 465–475 (2019). 30264934 10.1111/cns.13069 PMC6488926

[658] Rui, T. et al. Deletion of ferritin H in neurons counteracts the protective effect of melatonin against traumatic brain injury-induced ferroptosis. *J. Pineal Res* **70**, e12704 (2021). 33206394 10.1111/jpi.12704

[659] Gao, Y. et al. Melatonin ameliorates neurological deficits through MT2/IL-33/ferritin H signaling-mediated inhibition of neuroinflammation and ferroptosis after traumatic brain injury. *Free Radic. Biol. Med.* **199**, 97–112 (2023). 36805045 10.1016/j.freeradbiomed.2023.02.014

[660] Fang, J. et al. Overexpression of GPX4 attenuates cognitive dysfunction through inhibiting hippocampus ferroptosis and neuroinflammation after traumatic brain injury. *Free Radic. Biol. Med.* **204**, 68–81 (2023). 37105419 10.1016/j.freeradbiomed.2023.04.014

[661] Zhang, Z. et al. Glutathione peroxidase 4 participates in secondary brain injury through mediating ferroptosis in a rat model of intracerebral hemorrhage. *Brain Res.* **1701**, 112–125 (2018). 30205109 10.1016/j.brainres.2018.09.012

[662] Zhang, Y. et al. Netrin-1 upregulates GPX4 and prevents ferroptosis after traumatic brain injury via the UNC5B/Nrf2 signaling pathway. *CNS Neurosci. Ther.* **29**, 216–227 (2023). 36468399 10.1111/cns.13997 PMC9804081

[663] Huang, L. et al. Polydatin alleviates traumatic brain injury: role of inhibiting ferroptosis. *Biochem. Biophys. Res. Commun.* **556**, 149–155 (2021). 33839410 10.1016/j.bbrc.2021.03.108

[664] Alim, I. et al. Selenium drives a transcriptional adaptive program to block ferroptosis and treat stroke. *Cell* **177**, 1262–1279 e25 (2019). 31056284 10.1016/j.cell.2019.03.032

[665] Gao, Y. et al. Annexin A5 ameliorates traumatic brain injury-induced neuroinflammation and neuronal ferroptosis by modulating the NF-kB/HMGB1 and Nrf2/HO-1 pathways. *Int. Immunopharmacol.* **114**, 109619 (2023). 36700781 10.1016/j.intimp.2022.109619

[666] Shi, H. et al. Edaravone alleviates traumatic brain injury by inhibition of ferroptosis via FSP1 pathway. *Mol. Neurobiol.*10.1007/s12035-024-04216-2 (2024). 10.1007/s12035-024-04216-2 PMC11584507 38733490

[667] Hogan, S. R. et al. Discovery of lipidome alterations following traumatic brain injury via high-resolution metabolomics. *J. Proteome Res.* **17**, 2131–2143 (2018). 29671324 10.1021/acs.jproteome.8b00068 PMC7341947

[668] Kenny, E. M. et al. Ferroptosis contributes to neuronal death and functional outcome after traumatic brain injury. *Crit. Care Med.* **47**, 410–418 (2019). 30531185 10.1097/CCM.0000000000003555 PMC6449247

[669] Crichton, R. R., Ward, R. J. & Hider, R. C. The efficacy of iron chelators for removing iron from specific brain regions and the pituitary-ironing out the brain. *Pharmaceuticals* **12**, 138 (2019). 10.3390/ph12030138 PMC6789569 31533229

[670] Yu, J. et al. Effects of deferoxamine mesylate on hematoma and perihematoma edema after traumatic intracerebral hemorrhage. *J. Neurotrauma* **34**, 2753–2759 (2017). 28462672 10.1089/neu.2017.5033

[671] Yu, Y. et al. The clinical effect of deferoxamine mesylate on edema after intracerebral hemorrhage. *PLoS One* **10**, e0122371 (2015). 25875777 10.1371/journal.pone.0122371 PMC4395224

[672] Jia, H. et al. Deferoxamine ameliorates neurological dysfunction by inhibiting ferroptosis and neuroinflammation after traumatic brain injury. *Brain Res.* **1812**, 148383 (2023). 37149247 10.1016/j.brainres.2023.148383

[673] Zhang, C. et al. Deferoxamine induces autophagy following traumatic brain injury via TREM2 on microglia. *Mol. Neurobiol.* **61***,* 4649-4662 (2023). 10.1007/s12035-023-03875-x 38110648

[674] Cheng, Y. et al. Ferristatin II, an iron uptake inhibitor, exerts neuroprotection against traumatic brain injury via suppressing ferroptosis. *ACS Chem. Neurosci.* **13**, 664–675 (2022). 35143157 10.1021/acschemneuro.1c00819

[675] Khalaf, S., Ahmad, A. S., Chamara, K. & Dore, S. Unique properties associated with the brain penetrant iron chelator HBED reveal remarkable beneficial effects after brain trauma. *J. Neurotrauma* **36**, 43–53 (2018). 29743006 10.1089/neu.2017.5617 PMC6306957

[676] Cheng, H. et al. Neuroprotection of NRF2 against ferroptosis after traumatic brain injury in mice. *Antioxidants* **12**,731 (2023). 10.3390/antiox12030731 PMC10044792 36978979

[677] Zheng, B. et al. Netrin-1 mediates nerve innervation and angiogenesis leading to discogenic pain. *J. Orthop. Transl.* **39**, 21–33 (2023). 10.1016/j.jot.2022.11.006 PMC9804017 36605621

[678] Liang, J., Wu, S., Xie, W. & He, H. Ketamine ameliorates oxidative stress-induced apoptosis in experimental traumatic brain injury via the Nrf2 pathway. *Drug Des. Devel Ther.* **12**, 845–853 (2018). 29713142 10.2147/DDDT.S160046 PMC5907785

[679] Dong, W. et al. Curcumin plays neuroprotective roles against traumatic brain injury partly via Nrf2 signaling. *Toxicol. Appl. Pharm.* **346**, 28–36 (2018). 10.1016/j.taap.2018.03.020 29571711

[680] Zagorski, J. W. et al. Differential effects of the Nrf2 activators tBHQ and CDDO-Im on the early events of T cell activation. *Biochem. Pharm.* **147**, 67–76 (2018). 29155145 10.1016/j.bcp.2017.11.005 PMC5905342

[681] Bursley, J. K. & Rockwell, C. E. Nrf2-dependent and -independent effects of tBHQ in activated murine B cells. *Food Chem. Toxicol.* **145**, 111595 (2020). 32702509 10.1016/j.fct.2020.111595 PMC7568862

[682] Tang, H. et al. Protective effects of hinokitiol on neuronal ferroptosis by activating the keap1/Nrf2/HO-1 pathway in traumatic brain injury. *J. Neurotrauma* **41**, 734–750 (2024). 37962273 10.1089/neu.2023.0150

[683] Li, N. et al. Electroacupuncture inhibits neural ferroptosis in rat model of traumatic brain injury via activating system Xc-/GSH/GPX4 Axis. *Curr. Neurovasc. Res.* **21**, 86-100 (2024). 10.2174/0115672026297775240405073502 38629369

[684] Yang, Q. et al. Intermittent fasting ameliorates neuronal ferroptosis and cognitive impairment in mice after traumatic brain injury. *Nutrition* **109**, 111992 (2023). 36871445 10.1016/j.nut.2023.111992

[685] Wang, D. et al. Mesenchymal stromal cell treatment attenuates repetitive mild traumatic brain injury-induced persistent cognitive deficits via suppressing ferroptosis. *J. Neuroinflammation* **19**, 185 (2022). 35836233 10.1186/s12974-022-02550-7 PMC9281149

[686] Hu, X. et al. Spinal cord injury: molecular mechanisms and therapeutic interventions. *Signal. Transduct. Target Ther.* **8**, 245 (2023). 37357239 10.1038/s41392-023-01477-6 PMC10291001

[687] Liu, D. et al. ROS-scavenging hydrogels synergize with neural stem cells to enhance spinal cord injury repair via regulating microenvironment and facilitating nerve regeneration. *Adv. Health. Mater.* **12**, e2300123 (2023). 10.1002/adhm.202300123 36989238

[688] Ge, H. et al. Ferrostatin-1 alleviates white matter injury via decreasing ferroptosis following spinal cord injury. *Mol. Neurobiol.* **59**, 161–176 (2022). 34635980 10.1007/s12035-021-02571-y

[689] Li, J. Z. et al. Bioinformatics analysis of ferroptosis in spinal cord injury. *Neural Regen. Res.* **18**, 626–633 (2023). 36018187 10.4103/1673-5374.350209 PMC9727440

[690] Zhang, Y. et al. Ferroptosis inhibitor SRS 16-86 attenuates ferroptosis and promotes functional recovery in contusion spinal cord injury. *Brain Res.* **1706**, 48–57 (2019). 30352209 10.1016/j.brainres.2018.10.023

[691] Qu, D. et al. Identification and validation of ferroptosis-related genes in patients with acute spinal cord injury. *Mol. Neurobiol.* **60**, 5411–5425 (2023). 37316756 10.1007/s12035-023-03423-7

[692] Geng, H. et al. Restoring neuronal iron homeostasis revitalizes neurogenesis after spinal cord injury. *Proc. Natl. Acad. Sci. USA* **120**, e2220300120 (2023). 37948584 10.1073/pnas.2220300120 PMC10655560

[693] Xu, T. et al. FGF21 prevents neuronal cell ferroptosis after spinal cord injury by activating the FGFR1/beta-Klotho pathway. *Brain Res. Bull.* **202**, 110753 (2023). 37660729 10.1016/j.brainresbull.2023.110753

[694] Wang, C. et al. USP7 regulates HMOX-1 via deubiquitination to suppress ferroptosis and ameliorate spinal cord injury in rats. *Neurochem. Int.* **168**, 105554 (2023). 37257587 10.1016/j.neuint.2023.105554

[695] Yu, Z. et al. The ferroptosis activity is associated with neurological recovery following chronic compressive spinal cord injury. *Neural Regen. Res.* **18**, 2482–2488 (2023). 37282480 10.4103/1673-5374.371378 PMC10360078

[696] Shi, J. et al. Amelioration of white matter injury through mitigating ferroptosis following hepcidin treatment after spinal cord injury. *Mol. Neurobiol.* **60**, 3365–3378 (2023). 36853431 10.1007/s12035-023-03287-x

[697] Yao, X. et al. Deferoxamine promotes recovery of traumatic spinal cord injury by inhibiting ferroptosis. *Neural Regen. Res.* **14**, 532–541 (2019). 30539824 10.4103/1673-5374.245480 PMC6334606

[698] Fan, B. Y. et al. Liproxstatin-1 is an effective inhibitor of oligodendrocyte ferroptosis induced by inhibition of glutathione peroxidase 4. *Neural Regen. Res.* **16**, 561–566 (2021). 32985488 10.4103/1673-5374.293157 PMC7996026

[699] Friedmann Angeli, J. P. & Conrad, M. Selenium and GPX4, a vital symbiosis. *Free Radic. Biol. Med.* **127**, 153–159 (2018). 29522794 10.1016/j.freeradbiomed.2018.03.001

[700] Chen, Y. X. et al. Sodium selenite promotes neurological function recovery after spinal cord injury by inhibiting ferroptosis. *Neural Regen. Res* **17**, 2702–2709 (2022). 35662217 10.4103/1673-5374.339491 PMC9165358

[701] Bao, J. & Yang, S. ScRNA analysis and ferroptosis-related ceRNA regulatory network investigation in microglia cells at different time points after spinal cord injury. *J. Orthop. Surg. Res.* **18**, 701 (2023). 37726826 10.1186/s13018-023-04195-5 PMC10507978

[702] Li, W. et al. Ferroptosis inhibition protects vascular endothelial cells and maintains integrity of the blood-spinal cord barrier after spinal cord injury. *Neural Regen. Res.* **18**, 2474–2481 (2023). 37282479 10.4103/1673-5374.371377 PMC10360107

[703] Feng, Z. et al. Iron overload in the motor cortex induces neuronal ferroptosis following spinal cord injury. *Redox Biol.* **43**, 101984 (2021). 33933882 10.1016/j.redox.2021.101984 PMC8105676

[704] Shen, W. et al. Celastrol inhibits oligodendrocyte and neuron ferroptosis to promote spinal cord injury recovery. *Phytomedicine* **128**, 155380 (2024). 38507854 10.1016/j.phymed.2024.155380

[705] Ni, C. et al. Resveratrol inhibits ferroptosis via activating NRF2/GPX4 pathway in mice with spinal cord injury. *Microsc. Res. Tech.* **86**, 1378–1390 (2023). 37129001 10.1002/jemt.24335

[706] Zhang, L. et al. Albiflorin attenuates neuroinflammation and improves functional recovery after spinal cord injury through regulating LSD1-mediated microglial activation and ferroptosis. *Inflammation* **47**,1313-1327 (2024). 10.1007/s10753-024-01978-8 38340239

[707] Wang, Z. et al. Metformin attenuates ferroptosis and promotes functional recovery of spinal cord injury. *World Neurosurg.* **167**, e929–e939 (2022). 36058489 10.1016/j.wneu.2022.08.121

[708] Wang, Z. et al. Metformin alleviates spinal cord injury by inhibiting nerve cell ferroptosis through upregulation of heme oxygenase-1 expression. *Neural Regen. Res.* **19**, 2041–2049 (2024). 38227534 10.4103/1673-5374.390960 PMC11040287

[709] Zhou, H. et al. Proanthocyanidin promotes functional recovery of spinal cord injury via inhibiting ferroptosis. *J. Chem. Neuroanat.* **107**, 101807 (2020). 32474063 10.1016/j.jchemneu.2020.101807

[710] Ge, M. H. et al. Zinc attenuates ferroptosis and promotes functional recovery in contusion spinal cord injury by activating Nrf2/GPX4 defense pathway. *CNS Neurosci. Ther.* **27**, 1023–1040 (2021). 33951302 10.1111/cns.13657 PMC8339532

[711] Xu, J. et al. Identification of cathepsin B as a therapeutic target for ferroptosis of macrophage after spinal cord injury. *Aging Dis.* **15**, 421–443 (2023). 37307830 10.14336/AD.2023.0509 PMC10796092

[712] Virani, S. S. et al. Heart disease and stroke statistics-2021 update: a report from the american heart association. *Circulation* **143**, e254–e743 (2021). 33501848 10.1161/CIR.0000000000000950 PMC13036842

[713] Ajoolabady, A. et al. Targeting autophagy in ischemic stroke: from molecular mechanisms to clinical therapeutics. *Pharm. Ther.* **225**, 107848 (2021). 10.1016/j.pharmthera.2021.107848 PMC8263472 33823204

[714] Walter, K. What is acute ischemic stroke? *JAMA* **327**, 885 (2022). 35230392 10.1001/jama.2022.1420

[715] Koton, S. et al. Association of ischemic stroke incidence, severity, and recurrence with dementia in the atherosclerosis risk in communities cohort study. *JAMA Neurol.* **79**, 271–280 (2022). 35072712 10.1001/jamaneurol.2021.5080 PMC8787684

[716] Eltzschig, H. K. & Eckle, T. Ischemia and reperfusion-from mechanism to translation. *Nat. Med.* **17**, 1391–1401 (2011). 22064429 10.1038/nm.2507 PMC3886192

[717] Ding, S. et al. Delivery-mediated exosomal therapeutics in ischemia-reperfusion injury: advances, mechanisms, and future directions. *Nano Converg.* **11**, 18 (2024). 38689075 10.1186/s40580-024-00423-8 PMC11061094

[718] Cross, P. A., Atlas, S. W. & Grossman, R. I. MR evaluation of brain iron in children with cerebral infarction. *AJNR Am. J. Neuroradiol.* **11**, 341–348 (1990). 2107716 PMC8334700

[719] Yeh, S. J. et al. Association of ferroptosis with severity and outcomes in acute ischemic stroke patients undergoing endovascular thrombectomy: a case-control study. *Mol. Neurobiol.* **60**, 5902–5914 (2023). 37357230 10.1007/s12035-023-03448-y

[720] Millerot, E. et al. Serum ferritin in stroke: a marker of increased body iron stores or stroke severity? *J. Cereb. Blood Flow. Metab.* **25**, 1386–1393 (2005). 15902198 10.1038/sj.jcbfm.9600140

[721] DeGregorio-Rocasolano, N. et al. Iron-loaded transferrin (Tf) is detrimental whereas iron-free Tf confers protection against brain ischemia by modifying blood Tf saturation and subsequent neuronal damage. *Redox Biol.* **15**, 143–158 (2018). 29248829 10.1016/j.redox.2017.11.026 PMC5975212

[722] Zhang, W. et al. Associations of dietary iron intake with mortality from cardiovascular disease: the JACC study. *J. Epidemiol.* **22**, 484–493 (2012). 22986645 10.2188/jea.JE20120006 PMC3798559

[723] Zhang, Y. et al. Serum ferritin is associated with the presence of ischemic stroke among individuals with type 2 diabetes. *Heliyon* **10**, e27898 (2024). 38486737 10.1016/j.heliyon.2024.e27898 PMC10938112

[724] van der, A. D. et al. Serum ferritin is a risk factor for stroke in postmenopausal women. *Stroke* **36**, 1637–1641 (2005). 16002760 10.1161/01.STR.0000173172.82880.72

[725] Valdes Hernandez, M. D. C. et al. Association between striatal brain iron deposition, microbleeds and cognition 1 year after a minor ischaemic stroke. *Int. J. Mol. Sci.* **20**, 1293 (2019). 10.3390/ijms20061293 PMC6470500 30875807

[726] Millan, M. et al. Targeting pro-oxidant iron with deferoxamine as a treatment for ischemic stroke: safety and optimal dose selection in a randomized clinical trial. *Antioxidants* **10**, 1270 (2021). 10.3390/antiox10081270 PMC8389327 34439518

[727] Mehta, S. H. et al. Neuroprotection by tempol in a model of iron-induced oxidative stress in acute ischemic stroke. *Am. J. Physiol. Regul. Integr. Comp. Physiol.* **286**, R283–R288 (2004). 14592931 10.1152/ajpregu.00446.2002

[728] Justicia, C., Ramos-Cabrer, P. & Hoehn, M. MRI detection of secondary damage after stroke: chronic iron accumulation in the thalamus of the rat brain. *Stroke* **39**, 1541–1547 (2008). 18323485 10.1161/STROKEAHA.107.503565

[729] Zheng, H. et al. Cdh5-mediated Fpn1 deletion exerts neuroprotective effects during the acute phase and inhibitory effects during the recovery phase of ischemic stroke. *Cell Death Dis.* **14**, 161 (2023). 36841833 10.1038/s41419-023-05688-1 PMC9968354

[730] Su, W. et al. METTL3 regulates TFRC ubiquitination and ferroptosis through stabilizing NEDD4L mRNA to impact stroke. *Cell Biol. Toxicol.* **40**, 8 (2024). 38302612 10.1007/s10565-024-09844-x PMC10834616

[731] Li, C. et al. Nuclear receptor coactivator 4-mediated ferritinophagy contributes to cerebral ischemia-induced ferroptosis in ischemic stroke. *Pharm. Res.* **174**, 105933 (2021). 10.1016/j.phrs.2021.105933 34634471

[732] Cobley, J. N., Fiorello, M. L. & Bailey, D. M. 13 reasons why the brain is susceptible to oxidative stress. *Redox Biol.* **15**, 490–503 (2018). 29413961 10.1016/j.redox.2018.01.008 PMC5881419

[733] Tuo, Q. Z. et al. Characterization of selenium compounds for anti-ferroptotic activity in neuronal cells and after cerebral ischemia-reperfusion injury. *Neurotherapeutics* **18**, 2682–2691 (2021). 34498224 10.1007/s13311-021-01111-9 PMC8804037

[734] Lan, B. et al. Extract of Naotaifang, a compound Chinese herbal medicine, protects neuron ferroptosis induced by acute cerebral ischemia in rats. *J. Integr. Med.* **18**, 344–350 (2020). 32107172 10.1016/j.joim.2020.01.008

[735] Dingjan, I. et al. Endosomal and phagosomal SNAREs. *Physiol. Rev.* **98**, 1465–1492 (2018). 29790818 10.1152/physrev.00037.2017

[736] Si, W. et al. Snap25 attenuates neuronal injury via reducing ferroptosis in acute ischemic stroke. *Exp. Neurol.* **367**, 114476 (2023). 37393984 10.1016/j.expneurol.2023.114476

[737] Zimmermann, C. et al. Antioxidant status in acute stroke patients and patients at stroke risk. *Eur. Neurol.* **51**, 157–161 (2004). 15073440 10.1159/000077662

[738] Ahmad, S. et al. Sesamin attenuates neurotoxicity in mouse model of ischemic brain stroke. *Neurotoxicology* **45**, 100–110 (2014). 25316624 10.1016/j.neuro.2014.10.002

[739] Doll, S. & Conrad, M. Iron and ferroptosis: a still ill-defined liaison. *IUBMB Life* **69**, 423–434 (2017). 28276141 10.1002/iub.1616

[740] Krzyzanowska, W. et al. Ceftriaxone- and N-acetylcysteine-induced brain tolerance to ischemia: influence on glutamate levels in focal cerebral ischemia. *PLoS One* **12**, e0186243 (2017). 29045497 10.1371/journal.pone.0186243 PMC5646803

[741] Krzyzanowska, W. et al. N-acetylcysteine and ceftriaxone as preconditioning strategies in focal brain ischemia: influence on glutamate transporters expression. *Neurotox. Res.* **29**, 539–550 (2016). 26861954 10.1007/s12640-016-9602-z PMC4820483

[742] Liu, X. et al. NADPH debt drives redox bankruptcy: SLC7A11/xCT-mediated cystine uptake as a double-edged sword in cellular redox regulation. *Genes Dis.* **8**, 731–745 (2021). 34522704 10.1016/j.gendis.2020.11.010 PMC8427322

[743] Heit, B. S. et al. Tonic extracellular glutamate and ischaemia. *glutamate antiporter Syst. x(c.) (-) regulates anoxic depolarization hippocampus. J. Physiol.* **601**, 607–629 (2023). 10.1113/JP283880 PMC10107724 36321247

[744] Wang, L. et al. Nrf2 regulates oxidative stress and its role in cerebral ischemic stroke. *Antioxidants* **11**, 2377 (2022). 10.3390/antiox11122377 PMC9774301 36552584

[745] Dodson, M., Castro-Portuguez, R. & Zhang, D. D. NRF2 plays a critical role in mitigating lipid peroxidation and ferroptosis. *Redox Biol.* **23**, 101107 (2019). 30692038 10.1016/j.redox.2019.101107 PMC6859567

[746] Shih, A. Y., Li, P. & Murphy, T. H. A small-molecule-inducible Nrf2-mediated antioxidant response provides effective prophylaxis against cerebral ischemia in vivo. *J. Neurosci.* **25**, 10321–10335 (2005). 16267240 10.1523/JNEUROSCI.4014-05.2005 PMC6725780

[747] Zhang, J. et al. Micro ribonucleic acid 27a aggravates ferroptosis during early ischemic stroke of rats through nuclear factor erythroid-2-related factor 2. *Neuroscience* **504**, 10–20 (2022). 36180007 10.1016/j.neuroscience.2022.09.014

[748] Chen, Y. et al. Srs11-92, a ferrostatin-1 analog, improves oxidative stress and neuroinflammation via Nrf2 signal following cerebral ischemia/reperfusion injury. *CNS Neurosci. Ther.* **29**, 1667–1677 (2023). 36852441 10.1111/cns.14130 PMC10173707

[749] Gou, Z. et al. Melatonin improves hypoxic-ischemic brain damage through the Akt/Nrf2/Gpx4 signaling pathway. *Brain Res. Bull.* **163**, 40–48 (2020). 32679060 10.1016/j.brainresbull.2020.07.011

[750] Kloska, A., Malinowska, M., Gabig-Ciminska, M. & Jakobkiewicz-Banecka, J. Lipids and lipid mediators associated with the risk and pathology of ischemic stroke. *Int. J. Mol. Sci.* **21**, 3618 (2020). 10.3390/ijms21103618 PMC7279232 32443889

[751] Jin, G. et al. Protecting against cerebrovascular injury: contributions of 12/15-lipoxygenase to edema formation after transient focal ischemia. *Stroke* **39**, 2538–2543 (2008). 18635843 10.1161/STROKEAHA.108.514927 PMC2754072

[752] van Leyen, K. et al. Baicalein and 12/15-lipoxygenase in the ischemic brain. *Stroke* **37**, 3014–3018 (2006). 17053180 10.1161/01.STR.0000249004.25444.a5

[753] Cheng, G. et al. Effects of ML351 and tissue plasminogen activator combination therapy in a rat model of focal embolic stroke. *J. Neurochem.* **157**, 586–598 (2021). 33481248 10.1111/jnc.15308

[754] Karatas, H., Eun Jung, J., Lo, E. H. & van Leyen, K. Inhibiting 12/15-lipoxygenase to treat acute stroke in permanent and tPA induced thrombolysis models. *Brain Res.* **1678**, 123–128 (2018). 29079502 10.1016/j.brainres.2017.10.024 PMC5714685

[755] Zhao, J., Wu, Y., Liang, S. & Piao, X. Activation of SSAT1/ALOX15 axis aggravates cerebral ischemia/reperfusion injury via triggering neuronal ferroptosis. *Neuroscience* **485**, 78–90 (2022). 35090880 10.1016/j.neuroscience.2022.01.017

[756] Cui, Y. et al. ACSL4 exacerbates ischemic stroke by promoting ferroptosis-induced brain injury and neuroinflammation. *Brain Behav. Immun.* **93**, 312–321 (2021). 33444733 10.1016/j.bbi.2021.01.003

[757] Tuo, Q. Z. et al. Thrombin induces ACSL4-dependent ferroptosis during cerebral ischemia/reperfusion. *Signal. Transduct. Target Ther.* **7**, 59 (2022). 35197442 10.1038/s41392-022-00917-z PMC8866433

[758] Mao, R. & Liu, H. Depletion of mmu_circ_0001751 (circular RNA Carm1) protects against acute cerebral infarction injuries by binding with microRNA-3098-3p to regulate acyl-CoA synthetase long-chain family member 4. *Bioengineered* **13**, 4063–4075 (2022). 35114894 10.1080/21655979.2022.2032971 PMC8974190

[759] Ko, G. et al. Salvia miltiorrhiza alleviates memory deficit induced by ischemic brain injury in a transient mcao mouse model by inhibiting ferroptosis. *Antioxidants* **12**, 785 (2023). 10.3390/antiox12040785 PMC10135292 37107160

[760] Jin, Z. L. et al. Ring finger protein 146-mediated long-chain fatty-acid-coenzyme a ligase 4 ubiquitination regulates ferroptosis-induced neuronal damage in ischemic stroke. *Neuroscience* **529**, 148–161 (2023). 37591333 10.1016/j.neuroscience.2023.08.007

[761] Li, M. et al. Baicalein ameliorates cerebral ischemia-reperfusion injury by inhibiting ferroptosis via regulating GPX4/ACSL4/ACSL3 axis. *Chem. Biol. Interact.* **366**, 110137 (2022). 36055377 10.1016/j.cbi.2022.110137

[762] Sun, Y. et al. Melatonin alleviates ischemic stroke by inhibiting ferroptosis through the CYP1B1/ACSL4 pathway. *Environ. Toxicol.* **39**, 2623–2633 (2024). 38205686 10.1002/tox.24136

[763] Sun, J. et al. Ecdysterone improves oxidative damage induced by acute ischemic stroke via inhibiting ferroptosis in neurons through ACSL4. *J. Ethnopharmacol.* **331**, 118204 (2024). 38679397 10.1016/j.jep.2024.118204

[764] Sun, M. et al. Cottonseed oil alleviates ischemic stroke injury by inhibiting ferroptosis. *Brain Behav.* **13**, e3179 (2023). 37480159 10.1002/brb3.3179 PMC10570467

[765] Jin, Z. et al. Astragaloside IV alleviates neuronal ferroptosis in ischemic stroke by regulating fat mass and obesity-associated-N6-methyladenosine-acyl-CoA synthetase long-chain family member 4 axis. *J. Neurochem* **166**, 328–345 (2023). 37300304 10.1111/jnc.15871

[766] Li, X. N. et al. Caffeic acid alleviates cerebral ischemic injury in rats by resisting ferroptosis via Nrf2 signaling pathway. *Acta Pharm. Sin.* **45**, 248–267 (2024). 10.1038/s41401-023-01177-5 PMC10789749 37833536

[767] Hu, Q. et al. beta-Caryophyllene suppresses ferroptosis induced by cerebral ischemia reperfusion via activation of the NRF2/HO-1 signaling pathway in MCAO/R rats. *Phytomedicine* **102**, 154112 (2022). 35550220 10.1016/j.phymed.2022.154112

[768] Wu, C. et al. 15, 16-Dihydrotanshinone I protects against ischemic stroke by inhibiting ferroptosis via the activation of nuclear factor erythroid 2-related factor 2. *Phytomedicine* **114**, 154790 (2023). 37028247 10.1016/j.phymed.2023.154790

[769] Gao, J. et al. Icariside II preconditioning evokes robust neuroprotection against ischaemic stroke, by targeting Nrf2 and the OXPHOS/NF-kappaB/ferroptosis pathway. *Br. J. Pharm.* **180**, 308–329 (2023). 10.1111/bph.15961 36166825

[770] Fu, C. et al. Rehmannioside A improves cognitive impairment and alleviates ferroptosis via activating PI3K/AKT/Nrf2 and SLC7A11/GPX4 signaling pathway after ischemia. *J. Ethnopharmacol.* **289**, 115021 (2022). 35091012 10.1016/j.jep.2022.115021

[771] Yuan, Y. et al. Kaempferol ameliorates oxygen-glucose deprivation/reoxygenation-induced neuronal ferroptosis by activating Nrf2/SLC7A11/GPX4 axis. *Biomolecules* **11**, 3618 (2021). 10.3390/biom11070923 PMC8301948 34206421

[772] Liu, H. et al. Rhein attenuates cerebral ischemia-reperfusion injury via inhibition of ferroptosis through NRF2/SLC7A11/GPX4 pathway. *Exp. Neurol.* **369**, 114541 (2023). 37714424 10.1016/j.expneurol.2023.114541

[773] Liu, Y. et al. Loureirin C inhibits ferroptosis after cerebral ischemia reperfusion through regulation of the Nrf2 pathway in mice. *Phytomedicine* **113**, 154729 (2023). 36878093 10.1016/j.phymed.2023.154729

[774] Peng, C. et al. Quercetin attenuates cerebral ischemic injury by inhibiting ferroptosis via Nrf2/HO-1 signaling pathway. *Eur. J. Pharm.* **963**, 176264 (2024). 10.1016/j.ejphar.2023.176264 38123006

[775] Mi, Y. et al. Kellerin alleviates cerebral ischemic injury by inhibiting ferroptosis via targeting Akt-mediated transcriptional activation of Nrf2. *Phytomedicine* **128**, 155406 (2024). 38520834 10.1016/j.phymed.2024.155406

[776] Bai, X. et al. Angong Niuhuang Wan inhibit ferroptosis on ischemic and hemorrhagic stroke by activating PPARgamma/AKT/GPX4 pathway. *J. Ethnopharmacol.* **321**, 117438 (2024). 37984544 10.1016/j.jep.2023.117438

[777] Zhan, S. et al. SATB1/SLC7A11/HO-1 axis ameliorates ferroptosis in neuron cells after ischemic stroke by danhong injection. *Mol. Neurobiol.* **60**, 413–427 (2023). 36274077 10.1007/s12035-022-03075-z

[778] Liu, C. et al. Danlou tablet attenuates ischemic stroke injury and blood‒brain barrier damage by inhibiting ferroptosis. *J. Ethnopharmacol.* **322**, 117657 (2024). 38145861 10.1016/j.jep.2023.117657

[779] Liu, T. et al. Novel synergistic mechanism of 11-keto-beta-boswellic acid and Z-Guggulsterone on ischemic stroke revealed by single-cell transcriptomics. *Pharm. Res.* **193**, 106803 (2023). 10.1016/j.phrs.2023.106803 37230158

[780] Wang, G. L. et al. Electroacupuncture inhibits ferroptosis induced by cerebral ischemiareperfusion. *Curr. Neurovasc. Res.* **20**, 346–353 (2023). 37357521 10.2174/1567202620666230623153728

[781] Huang, L. Y. et al. Remote ischemic postconditioning-mediated neuroprotection against stroke by promoting ketone body-induced ferroptosis inhibition. *ACS Chem. Neurosci.* **15**, 2223–2232 (2024). 38634698 10.1021/acschemneuro.4c00014

[782] Wang, Y. et al. Anti-CHAC1 exosomes for nose-to-brain delivery of miR-760-3p in cerebral ischemia/reperfusion injury mice inhibiting neuron ferroptosis. *J. Nanobiotechnol.* **21**, 109 (2023). 10.1186/s12951-023-01862-x PMC10041751 36967397

[783] Hong, T. et al. Exosomal circBBS2 inhibits ferroptosis by targeting miR-494 to activate SLC7A11 signaling in ischemic stroke. *FASEB J.* **37**, e23152 (2023). 37603538 10.1096/fj.202300317RRR

[784] Ali, A. et al. Effect of exercise interventions on health-related quality of life after stroke and transient ischemic attack: a systematic review and meta-analysis. *Stroke* **52**, 2445–2455 (2021). 34039033 10.1161/STROKEAHA.120.032979

[785] Abdelmoez, A. M. et al. Comparative profiling of skeletal muscle models reveals heterogeneity of transcriptome and metabolism. *Am. J. Physiol. Cell Physiol.* **318**, C615–C626 (2020). 31825657 10.1152/ajpcell.00540.2019 PMC7099524

[786] Huang, M. et al. Preconditioning exercise inhibits neuron ferroptosis and ameliorates brain ischemia damage by skeletal muscle-derived exosomes via regulating miR-484/ACSL4 axis. *Antioxid. Redox Signal.*10.1089/ars.2023.0492 (2024). 10.1089/ars.2023.0492 38545792

[787] Smith, P. D. et al. The evolution of chemokine release supports a bimodal mechanism of spinal cord ischemia and reperfusion injury. *Circulation* **126**, S110–S117 (2012). 22965970 10.1161/CIRCULATIONAHA.111.080275

[788] Liu, S. et al. Ferrostatin-1 improves neurological impairment induced by ischemia/reperfusion injury in the spinal cord through ERK1/2/SP1/GPX4. *Exp. Neurol.* **373**, 114659 (2024). 38141803 10.1016/j.expneurol.2023.114659

[789] Rong, Y. et al. USP11 regulates autophagy-dependent ferroptosis after spinal cord ischemia-reperfusion injury by deubiquitinating Beclin 1. *Cell Death Differ.* **29**, 1164–1175 (2022). 34839355 10.1038/s41418-021-00907-8 PMC9177661

[790] Kania, A. & Klein, R. Mechanisms of ephrin-Eph signalling in development, physiology and disease. *Nat. Rev. Mol. Cell Biol.* **17**, 240–256 (2016). 26790531 10.1038/nrm.2015.16

[791] Dong, Y. et al. Eph receptor A4 regulates motor neuron ferroptosis in spinal cord ischemia/reperfusion injury in rats. *Neural Regen. Res.* **18**, 2219–2228 (2023). 37056141 10.4103/1673-5374.369118 PMC10328289

[792] Guo, L., Zhang, D., Ren, X. & Liu, D. SYVN1 attenuates ferroptosis and alleviates spinal cord ischemia-reperfusion injury in rats by regulating the HMGB1/NRF2/HO-1 axis. *Int. Immunopharmacol.* **123**, 110802 (2023). 37591122 10.1016/j.intimp.2023.110802

[793] Xiang, Q. et al. Regulated cell death in myocardial ischemia-reperfusion injury. *Trends Endocrinol. Metab.* **35**, 219–234 (2024). 37981501 10.1016/j.tem.2023.10.010

[794] Chang, H. C. et al. Reduction in mitochondrial iron alleviates cardiac damage during injury. *EMBO Mol. Med.* **8**, 247–267 (2016). 26896449 10.15252/emmm.201505748 PMC4772952

[795] Ju, J. et al. Circular RNA FEACR inhibits ferroptosis and alleviates myocardial ischemia/reperfusion injury by interacting with NAMPT. *J. Biomed. Sci.* **30**, 45 (2023). 37370086 10.1186/s12929-023-00927-1 PMC10304620

[796] Li, W. et al. Inhibition of DNMT-1 alleviates ferroptosis through NCOA4 mediated ferritinophagy during diabetes myocardial ischemia/reperfusion injury. *Cell Death Discov.* **7**, 267 (2021). 34588431 10.1038/s41420-021-00656-0 PMC8481302

[797] Park, T. J. et al. Quantitative proteomic analyses reveal that GPX4 downregulation during myocardial infarction contributes to ferroptosis in cardiomyocytes. *Cell Death Dis.* **10**, 835 (2019). 31685805 10.1038/s41419-019-2061-8 PMC6828761

[798] Coates, C. J., McCulloch, C., Betts, J. & Whalley, T. Echinochrome a release by red spherule cells is an iron-withholding strategy of sea urchin innate immunity. *J. Innate Immun.* **10**, 119–130 (2018). 29212075 10.1159/000484722 PMC6757146

[799] Park, J. H. et al. TherApeutic cell protective role of histochrome under oxidative stress in human cardiac progenitor cells. *Mar. Drugs* **17**, 368 (2019). 10.3390/md17060368 PMC6628112 31234277

[800] Hwang, J. W. et al. Histochrome attenuates myocardial ischemia-reperfusion injury by inhibiting ferroptosis-induced cardiomyocyte death. *Antioxidants* **10**, 1624 (2021). 10.3390/antiox10101624 PMC8533175 34679760

[801] Cao, Y. et al. KMT2B-dependent RFK transcription activates the TNF-alpha/NOX2 pathway and enhances ferroptosis caused by myocardial ischemia-reperfusion. *J. Mol. Cell Cardiol.* **173**, 75–91 (2022). 36162497 10.1016/j.yjmcc.2022.09.003

[802] Guo, J. et al. Mitochondria-derived methylmalonic acid aggravates ischemia-reperfusion injury by activating reactive oxygen species-dependent ferroptosis. *Cell Commun. Signal.* **22**, 53 (2024). 38238728 10.1186/s12964-024-01479-z PMC10797736

[803] Ichihara, G. et al. MRP1-dependent extracellular release of glutathione induces cardiomyocyte ferroptosis after ischemia-reperfusion. *Circ. Res.* **133**, 861–876 (2023). 37818671 10.1161/CIRCRESAHA.123.323517

[804] Ma, X. H. et al. ALOX15-launched PUFA-phospholipids peroxidation increases the susceptibility of ferroptosis in ischemia-induced myocardial damage. *Signal. Transduct. Target Ther.* **7**, 288 (2022). 35970840 10.1038/s41392-022-01090-z PMC9378747

[805] Liu, L. et al. Deubiquitinase OTUD5 as a novel protector against 4-HNE-triggered ferroptosis in myocardial ischemia/reperfusion injury. *Adv. Sci.* **10**, e2301852 (2023). 10.1002/advs.202301852 PMC10558642 37552043

[806] Qiu, M., Yan, W. & Liu, M. YAP facilitates NEDD4L-mediated ubiquitination and degradation of ACSL4 to alleviate ferroptosis in myocardial ischemia-reperfusion injury. *Can. J. Cardiol.* **39**, 1712–1727 (2023). 37541340 10.1016/j.cjca.2023.07.030

[807] Dong, L. et al. Research progress of chinese medicine in the treatment of myocardial ischemia-reperfusion injury. *Am. J. Chin. Med.* **51**, 1–17 (2023). 36437553 10.1142/S0192415X23500015

[808] Yang, B. et al. Salidroside pretreatment alleviates ferroptosis induced by myocardial ischemia/reperfusion through mitochondrial superoxide-dependent AMPKalpha2 activation. *Phytomedicine* **128**, 155365 (2024). 38552436 10.1016/j.phymed.2024.155365

[809] Ishimaru, K. et al. Deferasirox targeting ferroptosis synergistically ameliorates myocardial ischemia reperfusion injury in conjunction with cyclosporine A. *J. Am. Heart Assoc.* **13**, e031219 (2024). 38158218 10.1161/JAHA.123.031219 PMC10863836

[810] Qian, W. et al. Cyclosporine A-loaded apoferritin alleviates myocardial ischemia-reperfusion injury by simultaneously blocking ferroptosis and apoptosis of cardiomyocytes. *Acta Biomater.* **160**, 265–280 (2023). 36822483 10.1016/j.actbio.2023.02.025

[811] Yan, J. et al. Fucoxanthin alleviated myocardial ischemia and reperfusion injury through inhibition of ferroptosis via the NRF2 signaling pathway. *Food Funct.* **14**, 10052–10068 (2023). 37861458 10.1039/d3fo02633g

[812] Hu, T. et al. Resveratrol protects cardiomyocytes against ischemia/reperfusion-induced ferroptosis via inhibition of the VDAC1/GPX4 pathway. *Eur. J. Pharm.* **971**, 176524 (2024). 10.1016/j.ejphar.2024.176524 38561102

[813] Yang, T. et al. Galangin attenuates myocardial ischemic reperfusion-induced ferroptosis by targeting Nrf2/Gpx4 signaling pathway. *Drug Des. Dev. Ther.* **17**, 2495–2511 (2023). 10.2147/DDDT.S409232 PMC10460190 37637264

[814] Wang, R. et al. Kinsenoside mitigates myocardial ischemia/reperfusion-induced ferroptosis via activation of the Akt/Nrf2/HO-1 pathway. *Eur. J. Pharm.* **956**, 175985 (2023). 10.1016/j.ejphar.2023.175985 37572943

[815] Ge, C. et al. Hydroxysafflor yellow a alleviates acute myocardial ischemia/reperfusion injury in mice by inhibiting ferroptosis via the activation of the HIF-1alpha/SLC7A11/GPX4 signaling pathway. *Nutrients* **15**, 3411 (2023). 10.3390/nu15153411 PMC10420812 37571350

[816] Wang, I. C. et al. Baicalein and luteolin inhibit ischemia/reperfusion-induced ferroptosis in rat cardiomyocytes. *Int. J. Cardiol.* **375**, 74–86 (2023). 36513286 10.1016/j.ijcard.2022.12.018

[817] Lin, J. H. et al. Gossypol acetic acid attenuates cardiac ischemia/reperfusion injury in rats via an antiferroptotic mechanism. *Biomolecules* **11**, 1667 (2021). 10.3390/biom11111667 PMC8615989 34827665

[818] Song, Y. et al. Human umbilical cord blood-derived MSCs exosome attenuate myocardial injury by inhibiting ferroptosis in acute myocardial infarction mice. *Cell Biol. Toxicol.* **37**, 51–64 (2021). 32535745 10.1007/s10565-020-09530-8

[819] Pefanis, A., Ierino, F. L., Murphy, J. M. & Cowan, P. J. Regulated necrosis in kidney ischemia-reperfusion injury. *Kidney Int*. **96**, 291–301 (2019). 31005270 10.1016/j.kint.2019.02.009

[820] Thapa, K., Singh, T. G. & Kaur, A. Targeting ferroptosis in ischemia/reperfusion renal injury. *Naunyn Schmiedebergs Arch. Pharm.* **395**, 1331–1341 (2022). 10.1007/s00210-022-02277-5 35920897

[821] Tang, Q., Li, J., Wang, Y. & Sun, Q. Identification and verification of hub genes associated with ferroptosis in ischemia and reperfusion injury during renal transplantation. *Int. Immunopharmacol.* **120**, 110393 (2023). 37279643 10.1016/j.intimp.2023.110393

[822] Xie, R. et al. NAT10 drives cisplatin chemoresistance by enhancing ac4C-associated DNA repair in bladder cancer. *Cancer Res.* **83**, 1666–1683 (2023). 36939377 10.1158/0008-5472.CAN-22-2233

[823] Arango, D. et al. Acetylation of cytidine in mRNA promotes translation efficiency. *Cell* **175**, 1872–1886 e24 (2018). 30449621 10.1016/j.cell.2018.10.030 PMC6295233

[824] Shen, J. et al. NAT10 promotes renal ischemia-reperfusion injury via activating NCOA4-mediated ferroptosis. *Heliyon* **10**, e24573 (2024). 38312597 10.1016/j.heliyon.2024.e24573 PMC10835180

[825] Jin, L. et al. STING promotes ferroptosis through NCOA4-dependent ferritinophagy in acute kidney injury. *Free Radic. Biol. Med.* **208**, 348–360 (2023). 37634745 10.1016/j.freeradbiomed.2023.08.025

[826] Su, L. et al. Pannexin 1 mediates ferroptosis that contributes to renal ischemia/reperfusion injury. *J. Biol. Chem.* **294**, 19395–19404 (2019). 31694915 10.1074/jbc.RA119.010949 PMC6916502

[827] Gong, S. et al. REST contributes to AKI-to-CKD transition through inducing ferroptosis in renal tubular epithelial cells. *JCI Insight* **8** (2023). 10.1172/jci.insight.166001 PMC10393228 37288660

[828] Chu, L. K. et al. Autophagy of OTUD5 destabilizes GPX4 to confer ferroptosis-dependent kidney injury. *Nat. Commun.* **14**, 8393 (2023). 38110369 10.1038/s41467-023-44228-5 PMC10728081

[829] Ding, C. et al. miR-182-5p and miR-378a-3p regulate ferroptosis in I/R-induced renal injury. *Cell Death Dis.* **11**, 929 (2020). 33116120 10.1038/s41419-020-03135-z PMC7595188

[830] Lin, G. et al. Molecular mechanism of NR4A1/MDM2/P53 signaling pathway regulation inducing ferroptosis in renal tubular epithelial cells involved in the progression of renal ischemia-reperfusion injury. *Biochim. Biophys. Acta Mol. Basis Dis.* **1870**, 166968 (2024). 38008232 10.1016/j.bbadis.2023.166968

[831] Polyzos, A. A. et al. XJB-5-131-mediated improvement in physiology and behaviour of the R6/2 mouse model of Huntington’s disease is age- and sex- dependent. *PLoS One* **13**, e0194580 (2018). 29630611 10.1371/journal.pone.0194580 PMC5890981

[832] Zhao, Z. et al. XJB-5-131 inhibited ferroptosis in tubular epithelial cells after ischemia-reperfusion injury. *Cell Death Dis.* **11**, 629 (2020). 32796819 10.1038/s41419-020-02871-6 PMC7429848

[833] Shi, L. et al. MiR-20a-5p alleviates kidney ischemia/reperfusion injury by targeting ACSL4-dependent ferroptosis. *Am. J. Transpl.* **23**, 11–25 (2023). 10.1016/j.ajt.2022.09.003 36695612

[834] Zhao, Z. et al. Cytoplasmic HMGB1 induces renal tubular ferroptosis after ischemia/reperfusion. *Int. Immunopharmacol.* **116**, 109757 (2023). 36731154 10.1016/j.intimp.2023.109757

[835] Wang, H. et al. Carnosine attenuates renal ischemia-reperfusion injury by inhibiting GPX4-mediated ferroptosis. *Int. Immunopharmacol.* **124**, 110850 (2023). 37633236 10.1016/j.intimp.2023.110850

[836] Qi, Y. et al. Mitoglitazone ameliorates renal ischemia/reperfusion injury by inhibiting ferroptosis via targeting mitoNEET. *Toxicol. Appl. Pharm.* **465**, 116440 (2023). 10.1016/j.taap.2023.116440 36870574

[837] Wang, Y. et al. Quercetin alleviates acute kidney injury by inhibiting ferroptosis. *J. Adv. Res.* **28**, 231–243 (2021). 33364059 10.1016/j.jare.2020.07.007 PMC7753233

[838] Du, Y. W. et al. Cyanidin-3-glucoside inhibits ferroptosis in renal tubular cells after ischemia/reperfusion injury via the AMPK pathway. *Mol. Med.* **29**, 42 (2023). 37013504 10.1186/s10020-023-00642-5 PMC10069074

[839] Ma, L. et al. Paeoniflorin alleviates ischemia/reperfusion induced acute kidney injury by inhibiting Slc7a11-mediated ferroptosis. *Int. Immunopharmacol.* **116**, 109754 (2023). 36753983 10.1016/j.intimp.2023.109754

[840] Tang, Y. et al. Isoliquiritigenin attenuates septic acute kidney injury by regulating ferritinophagy-mediated ferroptosis. *Ren. Fail* **43**, 1551–1560 (2021). 34791966 10.1080/0886022X.2021.2003208 PMC8604484

[841] Kar, F. et al. LoxBlock-1 or Curcumin attenuates liver, pancreas and cardiac ferroptosis, oxidative stress and injury in Ischemia/reperfusion-damaged rats by facilitating ACSL/GPx4 signaling. *Tissue Cell* **82**, 102114 (2023). 37210761 10.1016/j.tice.2023.102114

[842] Lu, Y., Wang, L., Zhang, M. & Chen, Z. Mesenchymal stem cell-derived small extracellular vesicles: a novel approach for kidney disease treatment. *Int. J. Nanomed.* **17**, 3603–3618 (2022). 10.2147/IJN.S372254 PMC9386173 35990308

[843] Eirin, A. & Lerman, L. O. Mesenchymal stem/stromal cell-derived extracellular vesicles for chronic kidney disease: are we there yet? *Hypertension* **78**, 261–269 (2021). 34176287 10.1161/HYPERTENSIONAHA.121.14596 PMC8266760

[844] Sun, Z., Wu, J., Bi, Q. & Wang, W. Exosomal lncRNA TUG1 derived from human urine-derived stem cells attenuates renal ischemia/reperfusion injury by interacting with SRSF1 to regulate ASCL4-mediated ferroptosis. *Stem Cell Res. Ther.* **13**, 297 (2022). 35841017 10.1186/s13287-022-02986-x PMC9284726

[845] Jia, Y. et al. Metformin protects against intestinal ischemia-reperfusion injury and cell pyroptosis via TXNIP-NLRP3-GSDMD pathway. *Redox Biol.* **32**, 101534 (2020). 32330868 10.1016/j.redox.2020.101534 PMC7178548

[846] Zhu, L. et al. Integrated analysis of ferroptosis and immunity-related genes associated with intestinal ischemia/reperfusion injury. *J. Inflamm. Res.* **15**, 2397–2411 (2022). 35444445 10.2147/JIR.S351990 PMC9015787

[847] Li, Y. et al. Ischemia-induced ACSL4 activation contributes to ferroptosis-mediated tissue injury in intestinal ischemia/reperfusion. *Cell Death Differ.* **26**, 2284–2299 (2019). 30737476 10.1038/s41418-019-0299-4 PMC6889315

[848] Li, K., Wang, A., Diao, Y. & Fan, S. Oxidative medicine and cellular longevity the role and mechanism of NCOA4 in ferroptosis induced by intestinal ischemia reperfusion. *Int. Immunopharmacol.* **133**, 112155 (2024). 38688134 10.1016/j.intimp.2024.112155

[849] Deng, F. et al. The gut microbiota metabolite capsiate promotes Gpx4 expression by activating TRPV1 to inhibit intestinal ischemia reperfusion-induced ferroptosis. *Gut Microbes* **13**, 1–21 (2021). 33779497 10.1080/19490976.2021.1902719 PMC8009132

[850] Wang, X. et al. Resveratrol reduces ROS-induced ferroptosis by activating SIRT3 and compensating the GSH/GPX4 pathway. *Mol. Med.* **29**, 137 (2023). 37858064 10.1186/s10020-023-00730-6 PMC10588250

[851] Zhang, L. L. et al. Sestrin2 reduces ferroptosis via the Keap1/Nrf2 signaling pathway after intestinal ischemia-reperfusion. *Free Radic. Biol. Med.* **214**, 115–128 (2024). 38331008 10.1016/j.freeradbiomed.2024.02.003

[852] Chu, C. et al. Neutrophil extracellular traps drive intestinal microvascular endothelial ferroptosis by impairing Fundc1-dependent mitophagy. *Redox Biol.* **67**, 102906 (2023). 37812880 10.1016/j.redox.2023.102906 PMC10579540

[853] Zhongyin, Z., Wei, W., Juan, X. & Guohua, F. Isoliquiritin apioside relieves intestinal ischemia/reperfusion-induced acute lung injury by blocking Hif-1alpha-mediated ferroptosis. *Int. Immunopharmacol.* **108**, 108852 (2022). 35597117 10.1016/j.intimp.2022.108852

[854] Tong, L. et al. Current understanding of osteoarthritis pathogenesis and relevant new approaches. *Bone Res.* **10**, 60 (2022). 36127328 10.1038/s41413-022-00226-9 PMC9489702

[855] Xu, Y. et al. Characteristics and time points to inhibit ferroptosis in human osteoarthritis. *Sci. Rep.* **13**, 21592 (2023). 38062071 10.1038/s41598-023-49089-y PMC10703773

[856] Wang, L. et al. Ferroptosis-related genes LPCAT3 and PGD are potential diagnostic biomarkers for osteoarthritis. *J. Orthop. Surg. Res.* **18**, 699 (2023). 37723556 10.1186/s13018-023-04128-2 PMC10507893

[857] Han, Z. et al. Ferroptosis: a new target for iron overload-induced hemophilic arthropathy synovitis. *Ann. Hematol.* **102**, 1229–1237 (2023). 36951967 10.1007/s00277-023-05190-w

[858] Zhang, S. et al. The role played by ferroptosis in osteoarthritis: evidence based on iron dyshomeostasis and lipid peroxidation. *Antioxidants* **11**, 1668 (2022). 10.3390/antiox11091668 PMC9495695 36139742

[859] Li, H. et al. Combining single-cell RNA sequencing and population-based studies reveals hand osteoarthritis-associated chondrocyte subpopulations and pathways. *Bone Res.* **11**, 58 (2023). 37914703 10.1038/s41413-023-00292-7 PMC10620170

[860] Nugzar, O. et al. The role of ferritin and adiponectin as predictors of cartilage damage assessed by arthroscopy in patients with symptomatic knee osteoarthritis. *Best. Pr. Res Clin. Rheumatol.* **32**, 662–668 (2018). 10.1016/j.berh.2019.04.004 31203924

[861] Wu, L. et al. Association between iron intake and progression of knee osteoarthritis. *Nutrients* **14**, 1674 (2022). 10.3390/nu14081674 PMC9033045 35458236

[862] Hunter, D. J. & Bierma-Zeinstra, S. Osteoarthritis. *Lancet* **393**, 1745–1759 (2019). 31034380 10.1016/S0140-6736(19)30417-9

[863] Zhao, Z. et al. G protein-coupled receptor 30 activation inhibits ferroptosis and protects chondrocytes against osteoarthritis. *J. Orthop. Transl.* **44**, 125–138 (2024). 10.1016/j.jot.2023.12.003 PMC10839561 38318490

[864] Sun, K. et al. JNK-JUN-NCOA4 axis contributes to chondrocyte ferroptosis and aggravates osteoarthritis via ferritinophagy. *Free Radic. Biol. Med.* **200**, 87–101 (2023). 36907253 10.1016/j.freeradbiomed.2023.03.008

[865] Regan, E. A., Bowler, R. P. & Crapo, J. D. Joint fluid antioxidants are decreased in osteoarthritic joints compared to joints with macroscopically intact cartilage and subacute injury. *Osteoarthr. Cartil.* **16**, 515–521 (2008). 10.1016/j.joca.2007.09.001 18203633

[866] Yao, X. et al. Chondrocyte ferroptosis contribute to the progression of osteoarthritis. *J. Orthop. Transl.* **27**, 33–43 (2021). 10.1016/j.jot.2020.09.006 PMC7750492 33376672

[867] Wen, Z. et al. Selective clearance of senescent chondrocytes in osteoarthritis by targeting excitatory amino acid transporter protein 1 to induce ferroptosis. *Antioxid. Redox Signal.* **39**, 262–277 (2023). 36601724 10.1089/ars.2022.0141

[868] Lv, M. et al. The RNA-binding protein SND1 promotes the degradation of GPX4 by destabilizing the HSPA5 mRNA and suppressing HSPA5 expression, promoting ferroptosis in osteoarthritis chondrocytes. *Inflamm. Res.* **71**, 461–472 (2022). 35320827 10.1007/s00011-022-01547-5

[869] Zheng, Z. et al. P21 resists ferroptosis in osteoarthritic chondrocytes by regulating GPX4 protein stability. *Free Radic. Biol. Med.* **212**, 336–348 (2024). 38176476 10.1016/j.freeradbiomed.2023.12.047

[870] Zhao, C. et al. Forkhead box O3 attenuates osteoarthritis by suppressing ferroptosis through inactivation of NF-kappaB/MAPK signaling. *J. Orthop. Transl.* **39**, 147–162 (2023). 10.1016/j.jot.2023.02.005 PMC10175709 37188001

[871] Wang, S. et al. Mechanical overloading induces GPX4-regulated chondrocyte ferroptosis in osteoarthritis via Piezo1 channel facilitated calcium influx. *J. Adv. Res.* **41**, 63–75 (2022). 36328754 10.1016/j.jare.2022.01.004 PMC9637484

[872] Zhang, X. et al. Lipid peroxidation in osteoarthritis: focusing on 4-hydroxynonenal, malondialdehyde, and ferroptosis. *Cell Death Discov.* **9**, 320 (2023). 37644030 10.1038/s41420-023-01613-9 PMC10465515

[873] Grigolo, B., Roseti, L., Fiorini, M. & Facchini, A. Enhanced lipid peroxidation in synoviocytes from patients with osteoarthritis. *J. Rheumatol.* **30**, 345–347 (2003). 12563693

[874] Zou, Z. et al. Interplay between lipid dysregulation and ferroptosis in chondrocytes and the targeted therapy effect of metformin on osteoarthritis. *J. Adv. Res.* S2090-S1232 (2024).10.1016/j.jare.2024.04.01238621621

[875] Yan, J. et al. Metformin alleviates osteoarthritis in mice by inhibiting chondrocyte ferroptosis and improving subchondral osteosclerosis and angiogenesis. *J. Orthop. Surg. Res.* **17**, 333 (2022). 35765024 10.1186/s13018-022-03225-y PMC9238069

[876] Hu, Z. et al. Lipoxin A(4) ameliorates knee osteoarthritis progression in rats by antagonizing ferroptosis through activation of the ESR2/LPAR3/Nrf2 axis in synovial fibroblast-like synoviocytes. *Redox Biol.* **73**, 103143 (2024). 38754271 10.1016/j.redox.2024.103143 PMC11126537

[877] Sun, W. et al. XJB-5-131 protects chondrocytes from ferroptosis to alleviate osteoarthritis progression via restoring Pebp1 expression. *J. Orthop. Transl.* **44**, 114–124 (2024). 10.1016/j.jot.2023.12.005 PMC10830431 38304614

[878] Liu, Y. et al. Cartilage protective and anti-edema effects of JTF in osteoarthritis via inhibiting NCOA4-HMGB1-driven ferroptosis and aquaporin dysregulation. *Phytomedicine* **129**, 155593 (2024). 38621329 10.1016/j.phymed.2024.155593

[879] Xiao, J. et al. Icariin inhibits chondrocyte ferroptosis and alleviates osteoarthritis by enhancing the SLC7A11/GPX4 signaling. *Int. Immunopharmacol.* **133**, 112010 (2024). 38636375 10.1016/j.intimp.2024.112010

[880] Liu, J. et al. Baicalin inhibits IL-1beta-induced ferroptosis in human osteoarthritis chondrocytes by activating Nrf-2 signaling pathway. *J. Orthop. Surg. Res.* **19**, 23 (2024). 38166985 10.1186/s13018-023-04483-0 PMC10763085

[881] Ruan, Q., Wang, C., Zhang, Y. & Sun, J. Ruscogenin attenuates cartilage destruction in osteoarthritis through suppressing chondrocyte ferroptosis via Nrf2/SLC7A11/GPX4 signaling pathway. *Chem. Biol. Interact.* **388**, 110835 (2024). 38122922 10.1016/j.cbi.2023.110835

[882] Zhou, Y. et al. Curcumin reverses erastin-induced chondrocyte ferroptosis by upregulating Nrf2. *Heliyon* **9**, e20163 (2023). 37771529 10.1016/j.heliyon.2023.e20163 PMC10522940

[883] Ruan, Q., Wang, C., Zhang, Y. & Sun, J. Brevilin A attenuates cartilage destruction in osteoarthritis mouse model by inhibiting inflammation and ferroptosis via SIRT1/Nrf2/GPX4 signaling pathway. *Int. Immunopharmacol.* **124**, 110924 (2023). 37717314 10.1016/j.intimp.2023.110924

[884] Gong, Z. et al. Cardamonin alleviates chondrocytes inflammation and cartilage degradation of osteoarthritis by inhibiting ferroptosis via p53 pathway. *Food Chem. Toxicol.* **174**, 113644 (2023). 36731815 10.1016/j.fct.2023.113644

[885] Wan, Y., Shen, K., Yu, H. & Fan, W. Baicalein limits osteoarthritis development by inhibiting chondrocyte ferroptosis. *Free Radic. Biol. Med.* **196**, 108–120 (2023). 36657732 10.1016/j.freeradbiomed.2023.01.006

[886] Peng, S., Sun, C., Lai, C. & Zhang, L. Exosomes derived from mesenchymal stem cells rescue cartilage injury in osteoarthritis through Ferroptosis by GOT1/CCR2 expression. *Int. Immunopharmacol.* **122**, 110566 (2023). 37418985 10.1016/j.intimp.2023.110566

[887] Guan, Z. et al. The gut microbiota metabolite capsiate regulate SLC2A1 expression by targeting HIF-1alpha to inhibit knee osteoarthritis-induced ferroptosis. *Aging Cell* **22**, e13807 (2023). 36890785 10.1111/acel.13807 PMC10265160

[888] Zhou, X. et al. D-mannose alleviates osteoarthritis progression by inhibiting chondrocyte ferroptosis in a HIF-2alpha-dependent manner. *Cell Prolif.* **54**, e13134 (2021). 34561933 10.1111/cpr.13134 PMC8560605

[889] Yang, F. et al. Melatonin protects bone marrow mesenchymal stem cells against iron overload-induced aberrant differentiation and senescence. *J. Pineal Res.* **63**, e12422 (2017). 10.1111/jpi.12422 28500782

[890] Luo, C. et al. Canonical Wnt signaling works downstream of iron overload to prevent ferroptosis from damaging osteoblast differentiation. *Free Radic. Biol. Med.* **188**, 337–350 (2022). 35752374 10.1016/j.freeradbiomed.2022.06.236

[891] Chen, Y. et al. Type 2 diabetic mellitus related osteoporosis: focusing on ferroptosis. *J. Transl. Med.* **22**, 409 (2024). 38693581 10.1186/s12967-024-05191-x PMC11064363

[892] Zhang, T. et al. The multifaceted regulation of mitophagy by endogenous metabolites. *Autophagy* **18**, 1216–1239 (2022). 34583624 10.1080/15548627.2021.1975914 PMC9225590

[893] Wang, X. et al. Mitochondrial ferritin deficiency promotes osteoblastic ferroptosis via mitophagy in type 2 diabetic osteoporosis. *Biol. Trace Elem. Res.* **200**, 298–307 (2022). 33594527 10.1007/s12011-021-02627-z

[894] Park, S. Y., Choi, K. H., Jun, J. E. & Chung, H. Y. Effects of advanced glycation end products on differentiation and function of osteoblasts and osteoclasts. *J. Korean Med. Sci.* **36**, e239 (2021). 34581519 10.3346/jkms.2021.36.e239 PMC8476938

[895] Du, Y. X., Zhao, Y. T., Sun, Y. X. & Xu, A. H. Acid sphingomyelinase mediates ferroptosis induced by high glucose via autophagic degradation of GPX4 in type 2 diabetic osteoporosis. *Mol. Med.* **29**, 125 (2023). 37710183 10.1186/s10020-023-00724-4 PMC10500928

[896] Xu, P. et al. VDR activation attenuates osteoblastic ferroptosis and senescence by stimulating the Nrf2/GPX4 pathway in age-related osteoporosis. *Free Radic. Biol. Med.* **193**, 720–735 (2022). 36402439 10.1016/j.freeradbiomed.2022.11.013

[897] Wang, M. et al. ED-71 ameliorates bone regeneration in type 2 diabetes by reducing ferroptosis in osteoblasts via the HIF1alpha pathway. *Eur. J. Pharm.* **969**, 176303 (2024). 10.1016/j.ejphar.2023.176303 38211715

[898] Jin, C. et al. A novel anti-osteoporosis mechanism of VK2: interfering with ferroptosis via AMPK/SIRT1 pathway in type 2 diabetic osteoporosis. *J. Agric Food Chem.* **71**, 2745–2761 (2023). 36719855 10.1021/acs.jafc.2c05632

[899] Lu, J. et al. Extracellular vesicles from endothelial progenitor cells prevent steroid-induced osteoporosis by suppressing the ferroptotic pathway in mouse osteoblasts based on bioinformatics evidence. *Sci. Rep.* **9**, 16130 (2019). 31695092 10.1038/s41598-019-52513-x PMC6834614

[900] Jing, Z. et al. Tobacco toxins induce osteoporosis through ferroptosis. *Redox Biol.* **67**, 102922 (2023). 37826866 10.1016/j.redox.2023.102922 PMC10571034

[901] Zhang, H. et al. Osteoporotic bone loss from excess iron accumulation is driven by NOX4-triggered ferroptosis in osteoblasts. *Free Radic. Biol. Med.* **198**, 123–136 (2023). 36738798 10.1016/j.freeradbiomed.2023.01.026

[902] Jiang, Z. et al. Ferroptosis in osteocytes as a target for protection against postmenopausal osteoporosis. *Adv. Sci.* **11**, e2307388 (2024). 10.1002/advs.202307388 PMC10966575 38233202

[903] Ishii, K. A. et al. Coordination of PGC-1beta and iron uptake in mitochondrial biogenesis and osteoclast activation. *Nat. Med.* **15**, 259–266 (2009). 19252502 10.1038/nm.1910

[904] Qu, X., Sun, Z., Wang, Y. & Ong, H. S. Zoledronic acid promotes osteoclasts ferroptosis by inhibiting FBXO9-mediated p53 ubiquitination and degradation. *PeerJ* **9**, e12510 (2021). 35003915 10.7717/peerj.12510 PMC8684721

[905] Ni, S. et al. Hypoxia inhibits RANKL-induced ferritinophagy and protects osteoclasts from ferroptosis. *Free Radic. Biol. Med.* **169**, 271–282 (2021). 33895289 10.1016/j.freeradbiomed.2021.04.027

[906] Yin, Y. et al. Osteocyte ferroptosis induced by ATF3/TFR1 contributes to cortical bone loss during ageing. *Cell Prolif*. 10.1111/cpr.13657 (2024). 10.1111/cpr.13657 PMC11471391 38764128

[907] Yang, Y. et al. Targeting ferroptosis suppresses osteocyte glucolipotoxicity and alleviates diabetic osteoporosis. *Bone Res.* **10**, 26 (2022). 35260560 10.1038/s41413-022-00198-w PMC8904790

[908] Tao, L. et al. Exerkine FNDC5/irisin-enriched exosomes promote proliferation and inhibit ferroptosis of osteoblasts through interaction with Caveolin-1. *Aging Cell* **23**, e14181 (2024). 38689463 10.1111/acel.14181 PMC11320359

[909] Deng, X. et al. Mangiferin attenuates osteoporosis by inhibiting osteoblastic ferroptosis through Keap1/Nrf2/SLC7A11/GPX4 pathway. *Phytomedicine* **124**, 155282 (2024). 38176266 10.1016/j.phymed.2023.155282

[910] Xu, C. Y. et al. Poliumoside protects against type 2 diabetes-related osteoporosis by suppressing ferroptosis via activation of the Nrf2/GPX4 pathway. *Phytomedicine* **125**, 155342 (2024). 38295665 10.1016/j.phymed.2024.155342

[911] Yang, Y. et al. Prevention and treatment of osteoporosis with natural products: regulatory mechanism based on cell ferroptosis. *J. Orthop. Surg. Res.* **18**, 951 (2023). 38082321 10.1186/s13018-023-04448-3 PMC10712195

[912] Bauer, J. et al. Sarcopenia: a time for action. an SCWD position paper. *J. Cachexia Sarcopenia Muscle* **10**, 956–961 (2019). 31523937 10.1002/jcsm.12483 PMC6818450

[913] Scicchitano, B. M., Pelosi, L., Sica, G. & Musaro, A. The physiopathologic role of oxidative stress in skeletal muscle. *Mech. Ageing Dev.* **170**, 37–44 (2018). 28851603 10.1016/j.mad.2017.08.009

[914] Kozakowska, M., Pietraszek-Gremplewicz, K., Jozkowicz, A. & Dulak, J. The role of oxidative stress in skeletal muscle injury and regeneration: focus on antioxidant enzymes. *J. Muscle Res. Cell Motil.* **36**, 377–393 (2015). 26728750 10.1007/s10974-015-9438-9 PMC4762917

[915] DeRuisseau, K. C. et al. Aging-related changes in the iron status of skeletal muscle. *Exp. Gerontol.* **48**, 1294–1302 (2013). 23994517 10.1016/j.exger.2013.08.011 PMC3812819

[916] Hofer, T. et al. Increased iron content and RNA oxidative damage in skeletal muscle with aging and disuse atrophy. *Exp. Gerontol.* **43**, 563–570 (2008). 18395385 10.1016/j.exger.2008.02.007 PMC2601529

[917] Supruniuk, E., Gorski, J. & Chabowski, A. Endogenous and exogenous antioxidants in skeletal muscle fatigue development during exercise. *Antioxidants* **12**, 501 (2023). 10.3390/antiox12020501 PMC9952836 36830059

[918] Ikeda, Y. et al. Iron-induced skeletal muscle atrophy involves an Akt-forkhead box O3-E3 ubiquitin ligase-dependent pathway. *J. Trace Elem. Med. Biol.* **35**, 66–76 (2016). 27049128 10.1016/j.jtemb.2016.01.011

[919] Corna, G. et al. The repair of skeletal muscle requires iron recycling through macrophage ferroportin. *J. Immunol.* **197**, 1914–1925 (2016). 27465531 10.4049/jimmunol.1501417

[920] Chen, Y., Zhang, Y., Zhang, S. & Ren, H. Molecular insights into sarcopenia: ferroptosis-related genes as diagnostic and therapeutic targets. *J. Biomol. Struct. Dyn.*10.1080/07391102.2023.2298390 (2024). 10.1080/07391102.2023.2298390 38229237

[921] Zhong, D. et al. Induction of lysosomal exocytosis and biogenesis via TRPML1 activation for the treatment of uranium-induced nephrotoxicity. *Nat. Commun.* **14**, 3997 (2023). 37414766 10.1038/s41467-023-39716-7 PMC10326073

[922] Zhang, H. L. et al. TRPML1 triggers ferroptosis defense and is a potential therapeutic target in AKT-hyperactivated cancer. *Sci. Transl. Med.* **16**, eadk0330 (2024). 38924427 10.1126/scitranslmed.adk0330

[923] Lorito, N. et al. FADS1/2 control lipid metabolism and ferroptosis susceptibility in triple-negative breast cancer. *EMBO Mol. Med*. **16**,1533-1559 (2024). 10.1038/s44321-024-00090-6 PMC11251055 38926633

[924] Zeng, L. et al. Macrophage migration inhibitor factor (MIF): potential role in cognitive impairment disorders. *Cytokine Growth Factor Rev.* **77**, 67–75 (2024). 38548489 10.1016/j.cytogfr.2024.03.003

[925] Chen, D. et al. Small molecule mif modulation enhances ferroptosis by impairing DNA repair mechanisms. *Adv. Sci.* **11**, 2403963 (2024). 10.1002/advs.202403963 PMC11348242 38924362

[926] Yin, X. et al. HDAC1 governs iron homeostasis independent of histone deacetylation in iron-overload murine models. *Antioxid. Redox Signal.* **28**, 1224–1237 (2018). 29113455 10.1089/ars.2017.7161

[927] Meng, H. et al. Hepatic HDAC3 regulates systemic iron homeostasis and ferroptosis via the hippo signaling pathway. *Research* **6**, 0281 (2023). 38034086 10.34133/research.0281 PMC10687581

[928] Jiang, Z. et al. Attenuation of neuronal ferroptosis in intracerebral hemorrhage by inhibiting HDAC1/2: microglial heterogenization via the Nrf2/HO1 pathway. *CNS Neurosci. Ther.* **30**, e14646 (2024). 38523117 10.1111/cns.14646 PMC10961428

[929] Wang, L. et al. Structures and gating mechanism of human TRPM2. *Science* **362**, eaav4809 (2018). 10.1126/science.aav4809 PMC6459600 30467180

[930] Zhong, C. et al. TRPM2 mediates hepatic ischemia-reperfusion injury via Ca(2+)-induced mitochondrial lipid peroxidation through increasing ALOX12 expression. *Research* **6**, 0159 (2023). 37275121 10.34133/research.0159 PMC10232356

[931] Angeli, J. P. F., Shah, R., Pratt, D. A. & Conrad, M. Ferroptosis inhibition: mechanisms and opportunities. *Trends Pharm. Sci.* **38**, 489–498 (2017). 28363764 10.1016/j.tips.2017.02.005

[932] Funchal, C. et al. Morphological alterations and induction of oxidative stress in glial cells caused by the branched-chain alpha-keto acids accumulating in maple syrup urine disease. *Neurochem. Int.* **49**, 640–650 (2006). 16822590 10.1016/j.neuint.2006.05.007

[933] Li, T. et al. PPM1K mediates metabolic disorder of branched-chain amino acid and regulates cerebral ischemia-reperfusion injury by activating ferroptosis in neurons. *Cell Death Dis.* **14**, 634 (2023). 37752100 10.1038/s41419-023-06135-x PMC10522625

[934] Read, C. et al. International union of basic and clinical pharmacology. CVII. Structure and pharmacology of the apelin receptor with a recommendation that elabela/toddler is a second endogenous peptide ligand. *Pharm. Rev.* **71**, 467–502 (2019). 31492821 10.1124/pr.119.017533 PMC6731456

[935] Xu, P. et al. Elabela-APJ axis attenuates cerebral ischemia/reperfusion injury by inhibiting neuronal ferroptosis. *Free Radic. Biol. Med.* **196**, 171–186 (2023). 36681202 10.1016/j.freeradbiomed.2023.01.008

[936] Li, X. et al. Small extracellular vesicles delivering lncRNA WAC-AS1 aggravate renal allograft ischemia‒reperfusion injury by inducing ferroptosis propagation. *Cell Death Differ.* **30**, 2167–2186 (2023). 37532764 10.1038/s41418-023-01198-x PMC10482833

[937] Wu, T. et al. Circulating small extracellular vesicle-encapsulated SEMA5A-IT1 attenuates myocardial ischemia-reperfusion injury after cardiac surgery with cardiopulmonary bypass. *Cell Mol. Biol. Lett.* **27**, 95 (2022). 36284269 10.1186/s11658-022-00395-9 PMC9594885

[938] Huang, Q. et al. Identification of a targeted ACSL4 inhibitor to treat ferroptosis-related diseases. *Sci. Adv.* **10**, eadk1200 (2024). 38552012 10.1126/sciadv.adk1200 PMC10980261

[939] Duan, J. et al. Therapeutic targeting of hepatic ACSL4 ameliorates NASH in mice. *Hepatology* **75**, 140–153 (2022). 34510514 10.1002/hep.32148 PMC8688219

[940] Liu, Y. et al. In situ self-assembled phytopolyphenol-coordinated intelligent nanotherapeutics for multipronged management of ferroptosis-driven Alzheimer’s disease. *ACS Nano* **18**, 7890–7906 (2024). 38445977 10.1021/acsnano.3c09286

[941] Herpich, F. & Rincon, F. Management of acute ischemic stroke. *Crit. Care Med.* **48**, 1654–1663 (2020). 32947473 10.1097/CCM.0000000000004597 PMC7540624

[942] Jin, Q. et al. Edaravone-encapsulated agonistic micelles rescue ischemic brain tissue by tuning blood-brain barrier permeability. *Theranostics* **7**, 884–898 (2017). 28382161 10.7150/thno.18219 PMC5381251

[943] Zhang, Y. et al. Edaravone-loaded poly(amino acid) nanogel inhibits ferroptosis for neuroprotection in cerebral ischemia injury. *Asian J. Pharm. Sci.* **19**, 100886 (2024). 38590795 10.1016/j.ajps.2024.100886 PMC10999513

[944] Zhuge, X. et al. A multifunctional nanoplatform for chemotherapy and nanocatalytic synergistic cancer therapy achieved by amplified lipid peroxidation. *Acta Biomater.* **184**, 419-430 (2024). 10.1016/j.actbio.2024.06.029 38936754

[945] Ding, C. et al. Neutrophil membrane-inspired nanorobots act as antioxidants ameliorate ischemia reperfusion-induced acute kidney injury. *ACS Appl. Mater. Interfaces* **15**, 40292–40303 (2023). 37603686 10.1021/acsami.3c08573

[946] Niu, B. et al. Application of glutathione depletion in cancer therapy: enhanced ROS-based therapy, ferroptosis, and chemotherapy. *Biomaterials* **277**, 121110 (2021). 34482088 10.1016/j.biomaterials.2021.121110

[947] Muvhulawa, N. et al. Rutin ameliorates inflammation and improves metabolic function: a comprehensive analysis of scientific literature. *Pharm. Res.* **178**, 106163 (2022). 10.1016/j.phrs.2022.106163 35257898

[948] Negahdari, R. et al. Therapeutic benefits of rutin and its nanoformulations. *Phytother. Res.* **35**, 1719–1738 (2021). 33058407 10.1002/ptr.6904

[949] Feng, W. et al. Melanin-like nanoparticles alleviate ischemia-reperfusion injury in the kidney by scavenging reactive oxygen species and inhibiting ferroptosis. *iScience* **27**, 109504 (2024). 38632989 10.1016/j.isci.2024.109504 PMC11022057

[950] Zhang, Y. et al. Targeting ferroptosis by polydopamine nanoparticles protects heart against ischemia/reperfusion injury. *ACS Appl. Mater. Interfaces* **13**, 53671–53682 (2021). 34730938 10.1021/acsami.1c18061

[951] Yao, S. et al. Mesenchymal stem cell attenuates spinal cord injury by inhibiting mitochondrial quality control-associated neuronal ferroptosis. *Redox Biol.* **67**, 102871 (2023). 37699320 10.1016/j.redox.2023.102871 PMC10506061

[952] Hua, R. et al. ROS-responsive nanoparticle delivery of ferroptosis inhibitor prodrug to facilitate mesenchymal stem cell-mediated spinal cord injury repair. *Bioact. Mater.* **38**, 438–454 (2024). 38770428 10.1016/j.bioactmat.2024.05.015 PMC11103787

[953] Feng, Y. D. et al. Old targets, new strategy: apigenin-7-O-beta-d-(-6”-p-coumaroyl)-glucopyranoside prevents endothelial ferroptosis and alleviates intestinal ischemia-reperfusion injury through HO-1 and MAO-B inhibition. *Free Radic. Biol. Med.* **184**, 74–88 (2022). 35398494 10.1016/j.freeradbiomed.2022.03.033

[954] Zhao, X. et al. Apigenin-7-glucoside-loaded nanoparticle alleviates intestinal ischemia-reperfusion by ATF3/SLC7A11-mediated ferroptosis. *J. Control Release* **366**, 182–193 (2024). 38145659 10.1016/j.jconrel.2023.12.038

[955] Katz, J. N., Arant, K. R. & Loeser, R. F. Diagnosis and treatment of hip and knee osteoarthritis: a review. *JAMA* **325**, 568–578 (2021). 33560326 10.1001/jama.2020.22171 PMC8225295

[956] Yu, H. et al. Supramolecular self-assembly of EGCG-selenomethionine nanodrug for treating osteoarthritis. *Bioact. Mater.* **32**, 164–176 (2024). 37822916 10.1016/j.bioactmat.2023.09.020 PMC10563013

[957] Lv, Z. et al. Single cell RNA-seq analysis identifies ferroptotic chondrocyte cluster and reveals TRPV1 as an anti-ferroptotic target in osteoarthritis. *EBioMedicine* **84**, 104258 (2022). 36137413 10.1016/j.ebiom.2022.104258 PMC9494174

[958] Li, W. et al. Near infrared responsive gold nanorods attenuate osteoarthritis progression by targeting TRPV1. *Adv. Sci.* **11**, e2307683 (2024). 10.1002/advs.202307683 PMC11040380 38358041

[959] Li, Y. et al. A DNA tetrahedron-based ferroptosis-suppressing nanoparticle: superior delivery of curcumin and alleviation of diabetic osteoporosis. *Bone Res.* **12**, 14 (2024). 38424439 10.1038/s41413-024-00319-7 PMC10904802

[960] Rivella, S. Iron metabolism under conditions of ineffective erythropoiesis in beta-thalassemia. *Blood* **133**, 51–58 (2019). 30401707 10.1182/blood-2018-07-815928 PMC6318430

[961] Enns, C. A., Jue, S. & Zhang, A. S. The ectodomain of matriptase-2 plays an important nonproteolytic role in suppressing hepcidin expression in mice. *Blood* **136**, 989–1001 (2020). 32384154 10.1182/blood.2020005222 PMC7441170

[962] Nai, A. et al. Deletion of TMPRSS6 attenuates the phenotype in a mouse model of beta-thalassemia. *Blood* **119**, 5021–5029 (2012). 22490684 10.1182/blood-2012-01-401885 PMC3426375

[963] Schmidt, P. J. et al. An RNAi therapeutic targeting Tmprss6 decreases iron overload in Hfe(-/-) mice and ameliorates anemia and iron overload in murine beta-thalassemia intermedia. *Blood* **121**, 1200–1208 (2013). 23223430 10.1182/blood-2012-09-453977 PMC3655736

[964] Debacker, A. J. et al. Delivery of oligonucleotides to the liver with GalNAc: from research to registered therapeutic drug. *Mol. Ther.* **28**, 1759–1771 (2020). 32592692 10.1016/j.ymthe.2020.06.015 PMC7403466

[965] Vadolas, J. et al. SLN124, a GalNac-siRNA targeting transmembrane serine protease 6, in combination with deferiprone therapy reduces ineffective erythropoiesis and hepatic iron-overload in a mouse model of beta-thalassaemia. *Br. J. Haematol.* **194**, 200–210 (2021). 33942901 10.1111/bjh.17428 PMC8359948

[966] Ying, Y. et al. A shear-thinning, ROS-scavenging hydrogel combined with dental pulp stem cells promotes spinal cord repair by inhibiting ferroptosis. *Bioact. Mater.* **22**, 274–290 (2023). 36263097 10.1016/j.bioactmat.2022.09.019 PMC9556860

[967] Zandi, N. et al. Nanoengineered shear-thinning and bioprintable hydrogel as a versatile platform for biomedical applications. *Biomaterials* **267**, 120476 (2021). 33137603 10.1016/j.biomaterials.2020.120476 PMC7846391

[968] Zhao, T. et al. A triple-targeted rutin-based self-assembled delivery vector for treating ischemic stroke by vascular normalization and anti-inflammation via ACE2/Ang1-7 signaling. *ACS Cent. Sci.* **9**, 1180–1199 (2023). 37396868 10.1021/acscentsci.3c00377 PMC10311651

[969] Li, C. et al. Pleiotropic microenvironment remodeling micelles for cerebral ischemia-reperfusion injury therapy by inhibiting neuronal ferroptosis and glial overactivation. *ACS Nano* **17**, 18164–18177 (2023). 37703316 10.1021/acsnano.3c05038

[970] Sun, X. et al. Escin avoids hemorrhagic transformation in ischemic stroke by protecting BBB through the AMPK/Cav-1/MMP-9 pathway. *Phytomedicine* **120**, 155071 (2023). 37716034 10.1016/j.phymed.2023.155071

[971] Geng, Y. Q. et al. Alleviating recombinant tissue plasminogen activator-induced hemorrhagic transformation in ischemic stroke via targeted delivery of a ferroptosis inhibitor. *Adv. Sci.* **24**, e2309517 (2024). 10.1002/advs.202309517 PMC11199968 38647405

[972] Wang, Y. et al. Anti-ferroptosis exosomes engineered for targeting M2 microglia to improve neurological function in ischemic stroke. *J. Nanobiotechnol.* **22**, 291 (2024). 10.1186/s12951-024-02560-y PMC11129432 38802919

[973] Liu, H. T. et al. Effects of coenzyme Q10 supplementation on antioxidant capacity and inflammation in hepatocellular carcinoma patients after surgery: a randomized, placebo-controlled trial. *Nutr. J.* **15**, 85 (2016). 27716246 10.1186/s12937-016-0205-6 PMC5053088

[974] D’Amico, F. et al. Use of N-acetylcysteine during liver procurement: a prospective randomized controlled study. *Liver Transpl.* **19**, 135–144 (2013). 22859317 10.1002/lt.23527

[975] Lee, W. M. et al. Intravenous N-acetylcysteine improves transplant-free survival in early stage non-acetaminophen acute liver failure. *Gastroenterology* **137**, 856–64, (2009). 19524577 10.1053/j.gastro.2009.06.006 PMC3189485

[976] Berwanger, O. et al. Acetylcysteine for the prevention of renal outcomes in patients with diabetes mellitus undergoing coronary and peripheral vascular angiography: a substudy of the acetylcysteine for contrast-induced nephropathy trial. *Circ. Cardiovasc Inter.* **6**, 139–145 (2013). 10.1161/CIRCINTERVENTIONS.112.000149 23572490

[977] Li, N. & Zhou, H. SGLT2 inhibitors: a novel player in the treatment and prevention of diabetic cardiomyopathy. *Drug Des. Dev.Ther.* **14**, 4775–4788 (2020). 10.2147/DDDT.S269514 PMC7654518 33192053

[978] Elalfy, M. S. et al. Efficacy and safety of early-start deferiprone in infants and young children with transfusion-dependent beta thalassemia: evidence for iron shuttling to transferrin in a randomized, double-blind, placebo-controlled, clinical trial (START). *Am. J. Hematol.* **98**, 1415–1424 (2023). 37401738 10.1002/ajh.27010

[979] Hamdy, M. et al. Deferiprone versus deferoxamine for transfusional iron overload in sickle cell disease and other anemias: pediatric subgroup analysis of the randomized, open-label FIRST study. *Pediatr. Blood Cancer* **71**, e30711 (2024). 37807937 10.1002/pbc.30711

[980] Calvaruso, G. et al. Deferiprone versus deferoxamine in thalassemia intermedia: results from a 5-year long-term Italian multicenter randomized clinical trial. *Am. J. Hematol.* **90**, 634–638 (2015). 25809173 10.1002/ajh.24024

[981] Pennell, D. J. et al. A 1-year randomized controlled trial of deferasirox vs deferoxamine for myocardial iron removal in beta-thalassemia major (CORDELIA). *Blood* **123**, 1447–1454 (2014). 24385534 10.1182/blood-2013-04-497842 PMC3945858

[982] Maggio, A. et al. Evaluation of the efficacy and safety of deferiprone compared with deferasirox in paediatric patients with transfusion-dependent haemoglobinopathies (DEEP-2): a multicentre, randomised, open-label, non-inferiority, phase 3 trial. *Lancet Haematol.* **7**, e469–e478 (2020). 32470438 10.1016/S2352-3026(20)30100-9

[983] Sarocchi, M. et al. Cardiac effects of deferasirox in transfusion-dependent patients with myelodysplastic syndromes: TELESTO study. *Br. J. Haematol.* **204**, 2049–2056 (2024). 38343073 10.1111/bjh.19316

[984] Maggio, A. et al. Long-term use of deferiprone significantly enhances left-ventricular ejection function in thalassemia major patients. *Am. J. Hematol.* **87**, 732–733 (2012). 22622672 10.1002/ajh.23219

[985] Fernandes, J. L. et al. Amlodipine reduces cardiac iron overload in patients with thalassemia major: a pilot trial. *Am. J. Med.* **126**, 834–837 (2013). 23830536 10.1016/j.amjmed.2013.05.002

[986] Richard, F. et al. Oral ferroportin inhibitor VIT-2763: first-in-human, phase 1 study in healthy volunteers. *Am. J. Hematol.* **95**, 68–77 (2020). 31674058 10.1002/ajh.25670 PMC6916274

[987] Vidart, J. et al. N-acetylcysteine administration prevents nonthyroidal illness syndrome in patients with acute myocardial infarction: a randomized clinical trial. *J. Clin. Endocrinol. Metab.* **99**, 4537–4545 (2014). 25148231 10.1210/jc.2014-2192 PMC4255112

[988] Marian, A. J. et al. Hypertrophy regression with N-acetylcysteine in hypertrophic cardiomyopathy (HALT-HCM): a randomized, placebo-controlled, double-blind pilot study. *Circ. Res.* **122**, 1109–1118 (2018). 29540445 10.1161/CIRCRESAHA.117.312647 PMC5899034

[989] Lee, B. J., Tseng, Y. F., Yen, C. H. & Lin, P. T. Effects of coenzyme Q10 supplementation (300 mg/day) on antioxidation and anti-inflammation in coronary artery disease patients during statins therapy: a randomized, placebo-controlled trial. *Nutr. J.* **12**, 142 (2013). 24192015 10.1186/1475-2891-12-142 PMC4176102

[990] Foster, L. et al. Effect of deferoxamine on trajectory of recovery after intracerebral hemorrhage: a post hoc analysis of the i-DEF trial. *Stroke* **53**, 2204–2210 (2022). 35306827 10.1161/STROKEAHA.121.037298 PMC9246960

[991] Wei, C. et al. Effect of deferoxamine on outcome according to baseline hematoma volume: a post hoc analysis of the i-DEF trial. *Stroke* **53**, 1149–1156 (2022). 34789008 10.1161/STROKEAHA.121.035421 PMC8960321

[992] Selim, M. et al. Deferoxamine mesylate in patients with intracerebral haemorrhage (i-DEF): a multicentre, randomised, placebo-controlled, double-blind phase 2 trial. *Lancet Neurol.* **18**, 428–438 (2019). 30898550 10.1016/S1474-4422(19)30069-9 PMC6494117

[993] Sorond, F. A. et al. Deferoxamine, cerebrovascular hemodynamics, and vascular aging: potential role for hypoxia-inducible transcription factor-1-regulated pathways. *Stroke* **46**, 2576–2583 (2015). 26304864 10.1161/STROKEAHA.115.009906 PMC4551113

[994] Devos, D. et al. Trial of deferiprone in Parkinson’s disease. *N. Engl. J. Med.* **387**, 2045–2055 (2022). 36449420 10.1056/NEJMoa2209254

[995] Pilotto, F., Chellapandi, D. M. & Puccio, H. Omaveloxolone: a groundbreaking milestone as the first FDA-approved drug for Friedreich ataxia. *Trends Mol. Med.* **30**, 117–125 (2024). 38272714 10.1016/j.molmed.2023.12.002

[996] Wang, Y. et al. A membrane-targeting aggregation-induced emission probe for monitoring lipid droplet dynamics in ischemia/reperfusion-induced cardiomyocyte ferroptosis. *Adv. Sci.* **11**, e2309907 (2024). 10.1002/advs.202309907 PMC11234465 38696589

[997] Li, L. et al. Dual ratio and ultraprecision quantification of mitochondrial viscosity in ferroptosis enabled by a vibration-based triple-emission fluorescent probe. *Anal. Chem.* **95**, 17003–17010 (2023). 37942555 10.1021/acs.analchem.3c03541

[998] Tan, M. et al. Targeted mitochondrial fluorescence probe with large stokes shift for detecting viscosity changes in vivo and in ferroptosis process. *Spectrochim. Acta A Mol. Biomol. Spectrosc.* **315**, 124246 (2024). 38593540 10.1016/j.saa.2024.124246

[999] Yin, J. et al. Investigating the therapeutic effects of ferroptosis on myocardial ischemia-reperfusion injury using a dual-locking mitochondrial targeting strategy. *Angew. Chem. Int. Ed. Engl.* **63**, e202402537 (2024). 38509827 10.1002/anie.202402537

[1000] Luo, X. et al. A near-infrared light-activated nanoprobe for simultaneous detection of hydrogen polysulfide and sulfur dioxide in myocardial ischemia-reperfusion injury. *Chem. Sci.* **14**, 14290–14301 (2023). 38098706 10.1039/d3sc04937j PMC10718178

[1001] Yang, W. et al. Quantitative visualization of myocardial ischemia-reperfusion-induced cardiac lesions via ferroptosis magnetic particle imaging. *Theranostics* **14**, 1081–1097 (2024). 38250046 10.7150/thno.89190 PMC10797296

